# The synthesis and characterization of an iron(VII) nitrido complex

**DOI:** 10.1038/s41557-023-01418-4

**Published:** 2024-01-30

**Authors:** Martin Keilwerth, Weiqing Mao, Moritz Malischewski, Sergio A. V. Jannuzzi, Kevin Breitwieser, Frank W. Heinemann, Andreas Scheurer, Serena DeBeer, Dominik Munz, Eckhard Bill, Karsten Meyer

**Affiliations:** 1 Friedrich-Alexander-Universität Erlangen-Nürnberg (FAU), Department of Chemistry and Pharmacy, Inorganic Chemistry, Erlangen, Germany; 2 Freie Universität Berlin, Institute of Chemistry and Biochemistry, Inorganic Chemistry, Berlin, Germany; 3 Max Planck Institute for Chemical Energy Conversion, Mülheim an der Ruhr, Germany; 4 Saarland University, Inorganic Chemistry, Coordination Chemistry, Saarbrücken, Germany

**Keywords:** Chemical bonding, Synthetic chemistry methodology

## Abstract

Complexes of iron in high oxidation states are captivating research subjects due to their pivotal role as active intermediates in numerous catalytic processes. Structural and spectroscopic studies of well-defined model complexes often provide evidence of these intermediates. In addition to the fundamental molecular and electronic structure insights gained by these complexes, their reactivity also affects our understanding of catalytic reaction mechanisms for small molecule and bond-activation chemistry. Here, we report the synthesis, structural and spectroscopic characterization of a stable, octahedral Fe(VI) nitrido complex and an authenticated, unique Fe(VII) species, prepared by one-electron oxidation. The super-oxidized Fe(VII) nitride rearranges to an Fe(V) imide through an intramolecular amination mechanism and ligand exchange, which is characterized spectroscopically and computationally. This enables combined reactivity and stability studies on a single molecular system of a rare high-valent complex redox pair. Quantum chemical calculations complement the spectroscopic parameters and provide evidence for a diamagnetic (*S* = 0) *d* ^2^ Fe(VI) and a genuine *S* = 1/2, *d* ^1^ Fe(VII) configuration of these super-oxidized nitrido complexes.

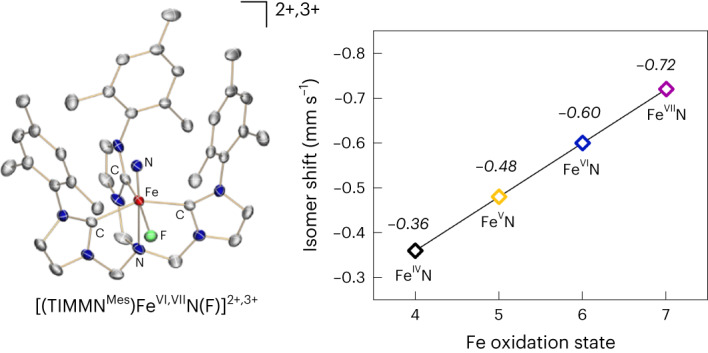

## Abstract (web summary)

Complexes of iron in high oxidation states play a pivotal role as active intermediates in numerous catalytic processes. Now, using a multimethod approach on a single molecular system, it has been shown that a stable high-valent Fe(VI) nitride can be converted to a reactive, superoxidized, heptavalent Fe(VII) nitride.

## Main

**Fig. 1:**
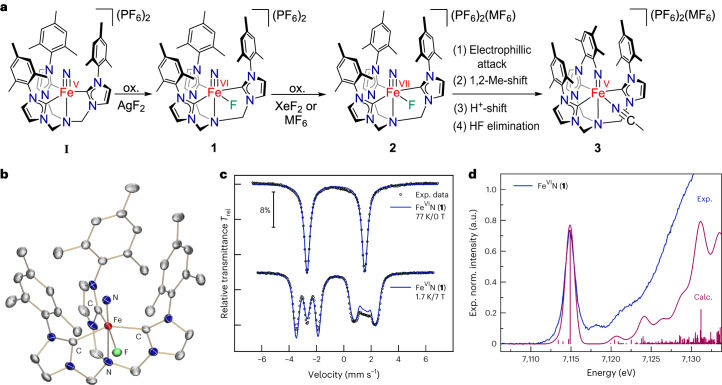
Synthetic scheme for the super-oxidized Fe(VI, VII) nitrides (1, 2), rearrangement to the cyclic Fe(V) imide (3), and molecular and electronic structure analysis of the stable Fe(VI) nitride (1). **a**, Oxidation of the Fe(VI) nitride (**1**) leads to a reactive Fe(VII) nitride (**2**) (MF_6_ with M = Mo, Re), which readily rearranges intramolecularly via C–C and C–H bond activation chemistry to a high-valent, unusual Fe(V) imide (**3**). **b**, Molecular structure of dication **1** in crystals of [(TIMMN^Mes^)Fe^VI^(N)(F)](PF_6_)_2_·CH_2_Cl_2_, depicting the coordination environment of the central hexavalent Fe ion, as well as the short Fe≡N bond length of 1.518(3) Å. Thermal ellipsoids are shown at 50% probability; counterions, hydrogen atoms and co-crystalized solvent molecules are omitted for clarity. **c**, Solid-state zero- and applied-field ^57^Fe Mössbauer spectra of **1** at 77 K (top) and 1.7 K (bottom). The coloured lines represent the best global fit for the experimental data (black circles) with a remarkably negative isomer shift of −0.60 mm s^−1^. **d**, Experimental Fe K-edge X-ray absorption spectrum of **1**, measured in K*β*_1,3_-HERFD mode, and the corresponding TDDFT-calculated spectrum (see Supplementary Information for details). Source data

Iron—the most abundant transition element—and its high oxidation state chemistry are of fundamental importance in various (bio)catalysts^1–4^. In this regard, the electronic structure and reactive nature of highly oxidized iron complexes are relevant for a general understanding of such catalytic processes^5–7^. In stark contrast to the notable stability of the permanganate ion [MnO_4_]^–^, the neighbouring ferrate(VII) anion [FeO_4_]^–^, generated by photolysis of the dioxoiron peroxide [(η^2^-O_2_)FeO_2_]^–^, is believed to be stable in helium matrices only at 4 K (refs. ^8,9^). With the exception of the shelf-stable, commercially available tetraoxoferrate(VI) dianion—which splits water into dioxygen^10,11^ and is also used for environmentally friendly water treatment^12^—only a handful of high-oxidation-state iron compounds are known. The hitherto accessible molecular compounds have been terminal Fe(IV,V) nitrido^13–19^, imido^20–23^ and oxo^24–29^ complexes, with their diverse reactivity attributed to the axial metal–ligand multiple bonds^30–32^. By contrast, only two Fe(VI) complexes have been reported before. These are a spectroscopically observed Fe(VI) nitride^33^, and a structurally authenticated, tetrahedral Fe(VI) *bis*(imide)^34^. Herein, we report the synthesis, isolation and full characterization of an octahedral Fe(VI) nitride (**1**). One-electron oxidation of **1** yields the unique Fe(VII) intermediate **2**, which is unambiguously spectroscopically characterized and rearranges to an Fe(V) imide product (**3**) (Fig. 1a). The calculated energy profile for the latter transformation provides further credence to a heptavalent iron complex (see below). Single-crystal X-ray diffraction (SC-XRD) studies of **1** and **3** revealed six-coordinate Fe centres in both compounds. The formation of the intramolecular amination product **3** emphasizes the high reactivity of the super-oxidized Fe(VII) intermediate **2**.

## Results and discussion

### Synthesis and characterization of the hexavalent iron nitride

Oxidation of the [(TIMMN^Mes^)Fe^V^(N)]^2+^ complex **I** (TIMMN^Mes^ = *tris*-[(3-mesityl-imidazol-2-ylidene)methyl]amine)^17^ with silver difluoride (Ag^II^F_2_) provides quantitative access to the hexavalent nitrido complex [(TIMMN^Mes^)Fe^VI^(N)(F)]^2+^ (**1**). The molecular structure of **1** (Fig. 1b), determined by SC-XRD analysis, reveals a six-coordinate iron centre best described as a *C*_s_-symmetric, distorted octahedron due to the additional, equatorially coordinated fluorido ligand. The iron centre is situated above the *tris*-carbene plane with an offset (*d*_oop_) of 0.419(3) Å and a short Fe≡N bond length of 1.518(3) Å. The average Fe−C_NHC_ distance is 1.976(4) Å with the shortest Fe−C bond length of 1.967(4) Å trans to the fluorido ligand, whereas the Fe−N_amine_ distance amounts to 2.351(3) Å.

Multinuclear ^1^H, ^13^C, ^15^N and ^19^F nuclear magnetic resonance (NMR) experiments also confirm that **1** is diamagnetic and *C*_s_-symmetric, unlike its trigonal-symmetric Fe(IV) and Fe(V) precursors and related imido complexes of the TIMMN^Mes^ ligand^17,20^. In solution, the fluorido ligand remains bound to the iron centre, as evidenced by an unusual ^19^F NMR chemical shift of −310 ppm (versus CFCl_3_; Supplementary Fig. 3), and couplings to the ^13^C and ^15^N nuclei in the respective NMR spectra. The zero-field ^57^Fe Mössbauer spectrum of **1** (Fig. 1c), recorded in the solid state at 77 K, shows a sharp quadrupole doublet with a remarkably negative isomer shift of −0.60 mm s^−1^ and a quadrupole splitting (Δ*E*_Q_) of 4.16 mm s^−1^, which is indicative of an Fe(VI) ion with a *d* ^2^, *S* = 0 electronic ground state (1.7 K, 7 T: *δ* = –0.58 mm s^−1^, Δ*E*_Q_, = +4.16 mm s^–1^, *η* = 0.54). Still, the negative isomer shift—indicative of a highly oxidized Fe ion—is much more negative than observed for the only other reported Fe(VI) complexes, namely, [(Me_3_cy-ac)Fe^VI^(N)](PF_6_)_2_ (cy-ac = 1,4,8,11-tetraazacyclotetradecane-1-acetate) (*δ* = −0.29 mm s^−1^, Fe−N distance (EXAFS) = 1.57(2) Å)^33^ and [H_2_B(^Mes^Im)_2_Fe^VI^(=N^Mes^)_2_]BF_4_ (H_2_B(^Mes^Im)_2_ = dihydro-*bis*[(1-mesityl)imidazol-2-ylidene]borato) (*δ* = −0.48 mm s^−1^, Fe−N distance = 1.634(3) Å)^34^. The markedly more negative isomer shift observed in hexavalent **1**, when compared with these other Fe(VI) complexes, can be attributed to the shorter, more covalent Fe−N bond in **1**. The effect of the ligand charge, first-shell ligand atoms, and coordination number require further in-depth analysis, as reported elsewhere^35^. X-ray absorption spectroscopy (XAS) measurements were conducted to elucidate further the electronic structure of hexavalent **1** and its related tetra- and pentavalent Fe(IV,V) nitrido complexes. The Fe K*β*_1,3_-HERFD XAS spectrum of [(TIMMN^Mes^)Fe^VI^(N)(F)]^2+^ (**1**) (Fig. 1d) shows a slightly asymmetric pre-edge peak at 7,114.9 eV and a minor feature on the onset of the rising edge at 7,119.2 eV. The inflection point at 7,126.9 eV of the rising edge follows the oxidation state trend of [Fe^IV,V^(N)]^1+/2+^ (**I′**, **I**), with the energies of 7,123.9 and 7,125.4 eV being consistent with a +VI oxidation state in **1** (Supplementary Fig. 17). These values are considerably higher than for other reported Fe(VI) complexes^33,34^ due to the 0.05 Å shorter Fe−N bond length in **1** compared with the experimentally determined EXAFS Fe−N data in [(Me_3_cy-ac)Fe^VI^(N)](PF_6_)_2_ (ref. ^33^). This trend is reflected within the more negative ^57^Fe Mössbauer isomer shift values of this Fe nitride series. The *C*_s_ symmetry of the complex stabilizes the 3*d*(xy) and 3*d*(x²–y²) orbitals relative to the parent *O*_h_ levels, causing the two *d* electrons to be paired in 3*d*(xy), thus, leading to the electronic configuration *a*″(xy)^2^, *a*′(xz)^0^, *a*″(yz)^0^, *a*′(x²–y²)^0^, *a*′(z²)^0^, as supported by quantum chemical calculations (Supplementary Fig. 48)^36–39^.

### Formation of the heptavalent iron nitride

**Fig. 2:**
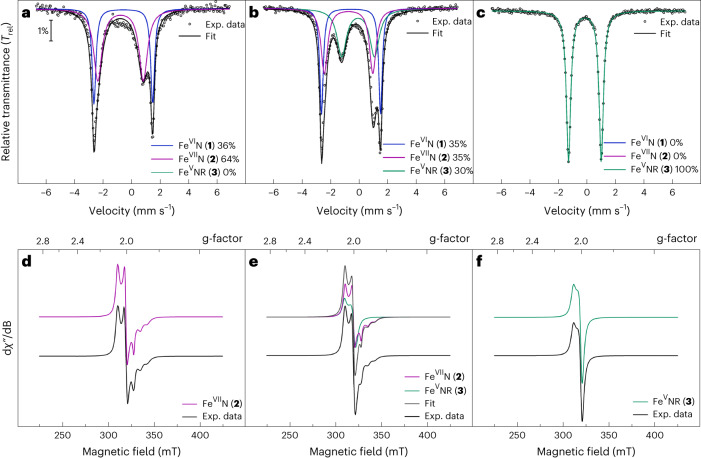
^57^Fe Mössbauer and EPR spectroscopic characterization of the super-oxidized Fe(VI, VII) nitrides (1, 2) and the rearranged Fe(V) imide (3). **a**, A mixture of Fe(VI)N (**1**) and Fe(VII)N (**2**) trapped in frozen solution and studied by zero-field ^57^Fe Mössbauer spectroscopy. While the Fe(VI) nitride possesses an isomer shift of –0.60 mm s^−1^, the Fe(VII) nitride reveals an exceptional negative isomer shift of −0.72 mm s^−1^ within the Fe nitride series. **b**, Rearrangement of the Fe(VII) nitride (**2**) to yield the Fe(V) imide (**3**). **c**, The zero-field ^57^Fe Mössbauer spectrum after complete conversion of **2** to **3**, with a less negative isomer shift of −0.16 mm s^−1^, typical for an Fe(V) imide. **d**–**f**, Formation, rearrangement and complete transformation of **2** to **3**, followed by X-band EPR spectroscopy. The Fe(VII) possesses an *S* = 1/2 signal (*g*_1_ = 2.058, *g*_2_ = 1.998, *g*_3_ = 1.908) with a characteristic three-line nitrogen hyperfine coupling (*A*_3_ = 6.8 mT) that vanishes following the rearrangement. ^57^Fe Mössbauer spectra **a–c** were recorded at 77 K and EPR spectra **d–f** at 15 K. Black circles (top) and lines (bottom) represent the experimental data, whereas the coloured lines represent the best fit obtained. Source data

**Fig. 3:**
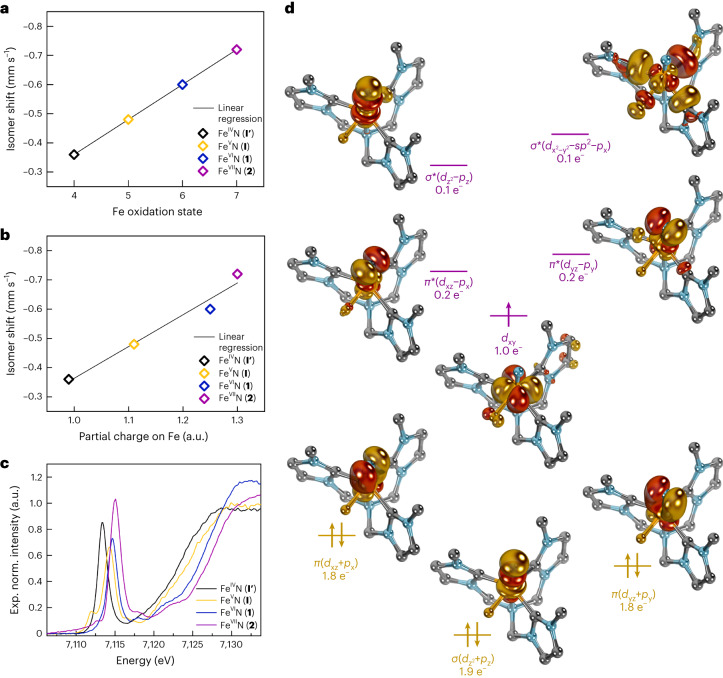
Characterization data of the Fe(IV–VII) nitride series (I′–2). **a**, Correlation diagram of the experimental (exp.) ^57^Fe Mössbauer isomer shift versus the Fe oxidation state of **1** and **2** compared with [(TIMMN^Mes^) Fe^IV,V^(N)]^1+,2+^ (**I**′, **I**). **b**, Correlation diagram of the exp. ^57^Fe Mössbauer isomer shift versus the calculated Bader atomic partial charge (quantum theory of atoms in molecules, Perdew–Burke-Ernzerhof functional) on Fe increasing within the **I′**–**2** series. **c**, Overlay of XAS spectra of **1**, deconvoluted **2** (see Supplementary Information for details) and [(TIMMN^Mes^)Fe^IV,V^(N)]^1+,2+^ (**I′,** **I**). The inflection points constantly shift to higher energies within the series (7,123.9 eV (**I′**), 7,125.4 eV (**I**), 7,126.9 eV (**1**) and 7,128.1 eV (**2**)), supporting the physical +VII oxidation state in **2**. **d**, Pertinent natural orbitals of the metal–nitrido moiety in **2** according to CASSCF(15,15) calculations, lead configuration: *c* = 0.66 (compare wth Supplementary Fig. 53), revealing three covalent bonds with the nitrido ligand (yellow) and an unpaired electron (purple) in the *d*(xy) orbital (singly occupied molecular orbital), corroborating a metal-centred spin for an Fe(VII), *d* ^1^ *S* = 1/2. Mesityl groups are truncated with methyl groups for clarity, yet have been included in the calculations. Source data

Unexpectedly, the particularly stable Fe(VI) complex **1** can be oxidized even further using powerful oxidizing agents, such as the metal hexafluorides (MF_6_ with M = Re, Mo) or salts containing the XeF^+^ cation (Supplementary Fig. 10). Treatment of **1** with either one of these reagents forms the one-electron-oxidized Fe(VII) nitride [(TIMMN^Mes^)Fe^VII^(N)(F)]^3+^ (**2**). This super-oxidized complex is highly reactive above −50 °C and rearranges (Fig. 2b,e) via an intramolecular amination mechanism^40,41^ to the cyclic Fe(V) imido complex [(TIMMN^Mes*^)Fe^V^(=N^*^)(NCMe)]^3+^ (**3**) (Fig. 2c,f). The freeze–quench ^57^Fe Mössbauer and X-band electron paramagnetic resonance (EPR) studies (Fig. 2), combined with XAS techniques (Supplementary Fig. 14), enabled the identification of **2**. The initial zero-field ^57^Fe Mössbauer spectrum (Fig. 2a) reveals **2** as the main component (64%) with an extraordinary negative isomer shift of −0.72 mm s^−1^ and Δ*E*_Q_ of 3.30 mm s^−1^. With the exception of ferrates (*δ* ≈ −0.80 mm s^−1^ at 78 K)^42^, the isomer shift in **2** represents the most negative value reported for any other known molecular iron coordination complex (see Supplementary Information for details), suggesting that the iron centre in **2** is the most strongly oxidized one. A linear correlation plot of the ^57^Fe Mössbauer isomer shift versus the iron oxidation state further supports an Fe(VII) physical oxidation state (Fig. 3a). A similar analysis was reported for Fe(VI)^33,34^. The remarkably linear relationship between the ^57^Fe Mössbauer isomer shift and the oxidation state is surprising, considering the complexes’ different structures. While **I**′ is trigonal four-coordinate, **I** is five-coordinate, and **1** and **2** are tetragonal six-coordinated with an extra fluorido ligand bound in the equatorial plane. The linear correlation reported between the *d*-electron count and the calculated electron density at the Fe nucleus^35^ indicates that the contraction of *s* orbitals—while the effective nuclear charge increases with the oxidation state—seems to be the dominant factor, overriding structural discrepancies across the Fe(IV–VII) series. The EPR spectroscopic data of **2** (Fig. 2d) reveal a doublet (*S* = 1/2) ground state with an anisotropic, rhombic ***g***-tensor with *g*_1_ = 2.058, *g*_2_ = 1.998, *g*_3_ = 1.908, and a super-hyperfine coupling to the nitrido nitrogen with *A*_3_ = 6.8 mT (^14^*N*, 99.63%, *I* = 1), which is indicative of a metal-centred spin. This characteristic three-line super-hyperfine-interaction with one coupled ^14^N nucleus vanishes upon thermally induced cyclization of **2** (Fig. 2d–f), and further matches the spectroscopic fingerprint (see below) of an independently synthesized sample of **3** (Supplementary Information). Owing to its diamagnetic (*d* ^2^, *S* = 0) ground state, unreacted **1** is EPR silent and, therefore, not seen in the spectrum of **2** (Fig. 2d).

The deconvoluted XAS data of [(TIMMN^Mes^)Fe^VII^(N)(F)]^3+^ (**2**) (Fig. 3c and Supplementary Fig. 14), from the reaction mixture evaporated at −50 °C, reveal a similar overall spectrum compared with **1**, but shifted to higher energies with an asymmetric pre-edge peak at 7,115.1 eV and a shoulder at 7,112.6 eV. Most importantly, the inflection point at 7,128.1 eV is 1.2 eV higher than for **1**, thus, following the oxidation state trend and supporting the physical +VII oxidation state assignment of **2** (Fig. 3c). Quantum chemical calculations at various levels of theory show a delocalization of the unpaired electron across the iron centre and to a minor extent to the NHC ligands as well as to the mesityl groups, supporting the metal as the main locus of oxidation. CASSCF(15,15) computations of **2**, which include the metal as well as the NHC- and mesityl substituents, identify three covalent bonds with the nitrido ligand and an unpaired electron in the *d*(xy) orbital (Fig. 3d; Supplementary Figs. 52 and 53; see Supplementary Fig. 48 for a comparison with **1**). While the computed spin density at the iron atom is method-dependent, we find that including the polar acetonitrile solvent and the hexafluorophosphate anions in the calculations corroborates metal-centred character. Density functional theory calculations reproduce the experimental Mössbauer data and support an increasing accumulation of positive charge on the Fe ion within each oxidation step going from +IV to +V, +VI, and +VII (Fig. 3b; see also Supplementary Tables 10–14 and Supplementary Figs. 31–34).

### Reactivity and mechanistic studies

**Fig. 4:**
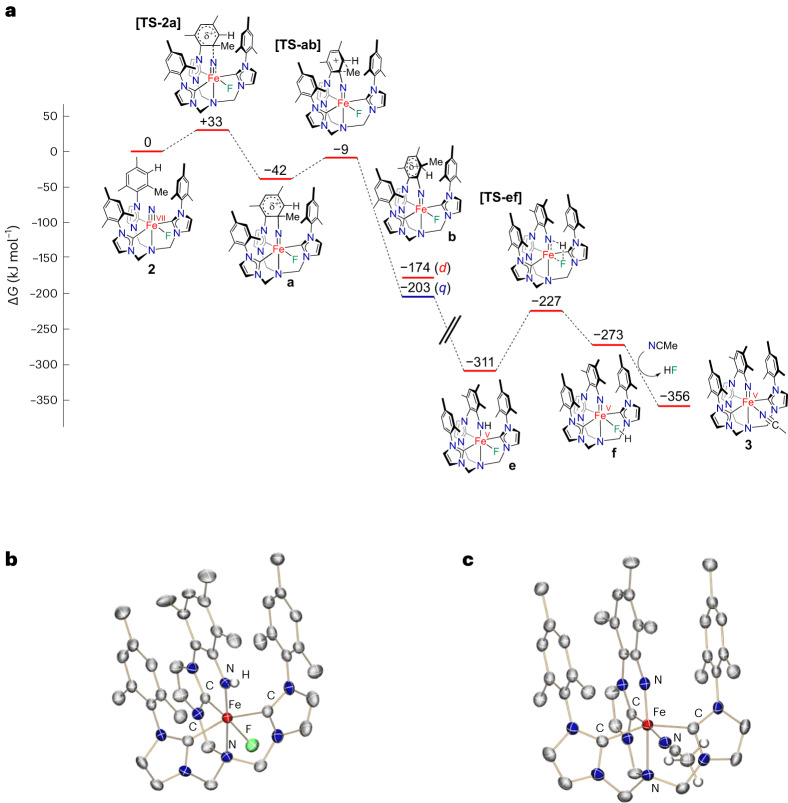
Calculated energy profile for the intramolecular transformation of the super-oxidized Fe(VII) nitride (2) via the intermediary Fe(V)amide (2e) to the cyclic Fe(V) imide (3). **a**, Computed reaction profile (ZORA-TPSSh-D3(CPCM)/def2-TZVPP//ZORA-PBE-D3/def2-SVP). Proton transfer sequences **c** and **d** are omitted for clarity (see Supplementary Fig. 27). **b**, Molecular structure of intermediary Fe(V)amide **2e** in crystals of [(TIMMN^Mes*^)Fe^V^(NH^*^)(F)](PF_6_)_3_, depicting the coordination environment of the Fe ion (*d*Fe–N_amido_ = 1.752(3) Å). **c**, Molecular structure of **3** in crystals of [(TIMMN^Mes*^)Fe^V^(=N^*^)(NCMe)](PF_6_)_2_(MoF_6_)·5 CH_3_CN depicting the coordination environment of the central pentavalent Fe ion with a typical Fe=N_imido_ bond length of 1.679(3) Å. All thermal ellipsoids for **b** and **c** are shown at 50% probability; counterions, hydrogen atoms and co-crystalized solvent molecules are omitted for clarity, except the amido proton in **b** and the acetonitrile hydrogens in **c**.

When leaving frozen-matrix conditions, a rapid intramolecular rearrangement reaction occurs (Fig. 4a), thus, thwarting the isolation of heptavalent **2**. Low-temperature crystallization attempts at −30 °C provided further mechanistic clarification, revealing that the Fe(V) imido complex [(TIMMN^Mes*^)Fe^V^(=N^*^)(NCMe)]^3+^ (**3**) (Fig. 4c) forms as the stable amination product. Following oxidization of **1** to **2**, complex **3** forms through subsequent HF−MeCN ligand exchange under ambient conditions. Single-crystal X-ray diffraction analysis corroborates the *C*_s_-symmetric nature of **3** and a retained six-coordinate Fe centre with a *d*_oop_ of 0.399(3) Å. Due to HF elimination^43^, the newly formed aryl-imido metallacycle possesses a typical^20,23^ Fe^V^=N_imido_ bond length of 1.679(3) Å and an N−C bond length of 1.340(4) Å. Consequently, the aryl substituents of the ligand framework in **3** align parallel to each other in a graphene-type fashion with an intramolecular aryl−aryl distance of 3.343 Å (Supplementary Fig. 24). The average Fe−C_NHC_ distance is 1.936(4) Å with a remarkably short Fe−C bond length of 1.855(4) Å *trans* to the acetonitrile ligand, and the Fe−N_amine_ distance amounts to 2.172(3) Å. The zero-field ^57^Fe Mössbauer spectrum of **3** (Fig. 2c) is characteristic for a *d* ^3^, *S* = 1/2 electronic ground state of an Fe(V) imide^20,34^ with an isomer shift of −0.16 mms^−1^ and Δ*E*_Q_ of 2.28 mms^−1^. The EPR spectroscopic data (Fig. 2f) confirm the low-spin (*S* = 1/2) state with an anisotropic ***g***-tensor (*g*_1_ = 2.065, *g*_2_ = 2.000, *g*_3_ = 1.984). The overall reaction sequence is evidenced by quantum chemical calculations (Fig. 4a). An electrophilic attack of the nitrido ligand on the chelate’s mesityl group in **2** causes an initial N−C bond formation (Fig. 4a, complex **a**). The arenium ion rearranges by a 1,2-methyl shift (Fig. 4a, complex **b**) and a subsequent proton migration via the mesityl rings (Supplementary Fig. 27, complexes **c** and **d**) to afford the Fe(V) amido complex (Fig. 4a, complex **e**). The following elimination of HF is caused by the coordination of an incoming acetonitrile solvent molecule, which renders Fe(V) imido complex **3** thermodynamically stable. Notably, the migration of the methyl group, involving a 1,2-methyl shift followed by proton migration, has an electronic and steric origin. First, the 1,2-methyl shift allows for better stabilization of positive charge in the *meta*-position of the mesityl substituent (Fig. 4a, complexes **a** versus **b**). Second, as the Fe-nitrido nitrogen atom forms the metallacycle through the electrophilic attack and N–C bond formation, the methyl group probably clashes with the neighbouring aryl groups. The following 1,2-methyl shift and subsequent H^+^ transfer release the steric pressure, rearomatize the arene and allow the three aryl rings to align in a graphene-type *π*-stacking. At first, this reaction sequence yields a unique but unstable Fe(V) amido species (Fig. 4b, complex **e**), which, coincidentally, has been crystallographically characterized from HF (Supplementary Fig. 25). Ultimately, the stable Fe(V) imide (**3**) is formed via H–F elimination. Computing the corresponding reaction for hexavalent **1** confirms that the nitrido ligand is not sufficiently electrophilic for a reaction with the mesityl group, which renders the addition step considerably endergonic (see Supplementary Fig. 28).

Together, these spectroscopic data corroborate the conversion of a high-valent Fe(VI) nitride (**1**) to a reactive, super-oxidized, heptavalent iron nitride (**2**) en route to an Fe(V) imide (**3**) via an intramolecular amination mechanism. This study, thus, establishes the full structural characterization of a molecular hexavalent Fe(VI) nitride and the spectroscopic identification of a genuine heptavalent Fe(VII) nitrido complex.

## Methods

### General considerations

All experiments were performed under a dry nitrogen atmosphere, using standard Schlenk techniques or an MBraun inert-gas glovebox, equipped with a –35 °C freezer. Solvents were purified using a two-column solid-state purification system (Glass Contour System) and stored over activated molecular sieves (3 Å) under an inert atmosphere for all halogenated, nitro- or nitrile-containing solvents (dichloromethane, chloroform, acetonitrile and nitromethane). Furthermore, potassium was added to alkanes, aromatic solvents and cyclic ethers (*n*-pentane, *n*-hexane, benzene, toluene and tetrahydrofuran). Sulfur dioxide (SO_2_) is a toxic gas (boiling point −10 °C) and was stored in a stainless-steel cylinder over CaH_2_. Anhydrous HF is a highly toxic and corrosive gas (boiling point = +19 °C), which was purified by distillation and stored in a stainless-steel cylinder. MoF_6_ and ReF_6_ (ABCR) are highly volatile liquids, corrosive and strongly oxidizing, and were also stored in stainless-steel cylinders. Reactions involving SO_2_, HF, MoF_6_ and ReF_6_ were performed in tetrafluoroethene-perfluoroalkoxyvinyl-copolymer (PFA) tubes connected to stainless-steel valves. Deuterated solvents were obtained, packaged under argon and stored over activated molecular sieves. The NMR spectra were recorded on a JEOL ECZ 400 S instrument operating at 399.79 MHz for ^1^H and 100.53 MHz for ^13^C. The solvent residual signals of incomplete deuterated solvent molecules were used as the internal reference for the ^1^H NMR spectra and the solvent signals for ^13^C NMR spectral data^44^. For ^15^N, ^19^F and ^31^P NMR data, chemical shifts were referenced by the Delta v.5.0.5 software provided by JEOL Ltd NMR. Multiplicities are abbreviated as follows: s = singlet, d = doublet, t = triplet, q = quartet, m = multiplet, b = broad. Coupling constants *J* are given in hertz. Zero-field ^57^Fe Mössbauer spectra were recorded on a WissEl Mössbauer spectrometer (MRG-500) at a temperature of 77 K in constant acceleration mode. ^57^Co/Rh was used as *γ*-radiation source (1.8 GBq). The temperature of the samples was controlled by a MBBC-HE0106 MÖSSBAUER He/N_2_ cryostat within an accuracy of ±0.3 K. Applied-field ^57^Fe Mössbauer spectra were recorded with alternating constant acceleration of the *γ*-source (lab-built spectrometer, MPI-CEC). The sample temperature was maintained constant in a cryogen-free, closed-cycle Mössbauer magnet cryostat from Cryogenic Ltd. The latter is a split-pair super-conducting magnet system for applied fields of up to 7 T. The temperature of the sample can be varied in the range 1.7 K to 300 K. Isomer shifts are reported relative to *α*-iron metal at 300 K. Electron paramagnetic resonance spectra were recorded under nitrogen atmosphere in air-tight J. Young quartz glass EPR tubes on a JEOL continuous wave spectrometer (JES-FA200) equipped with an X-band Gunn diode oscillator bridge, a cylindrical mode cavity, and a helium cryostat using the following parameters: microwave frequency = 8.959 GHz, modulation width = 1.0 mT, microwave power = 1.0 mW, modulation frequency = 100 kHz, and time constant = 0.1 s. Electron paramagnetic resonance line widths, *W*, are given in units of millitesla at the full-width at half-maximum. X-ray absorption spectroscopy in high-energy resolution fluorescence-detected mode (K*β*-HERFD) was performed at the European Synchrotron Radiation Facility (ESRF) beam line ID26 (6 GeV, 30 mA, 16-bunch mode) using a Si(311) liquid nitrogen cooled double crystal monochromator. The emission spectrometer was set to the maximum of K*β*1,3 line and the absorption spectrum was collected in continuous scan mode from 7,080 to 7,250 eV with integration set to give a 0.1 eV step size. Scans from 7,020 to 7,760 eV were collected for normalization. The final data were composed of an average of scans on 60–80 spots on the surface of a single sample. X-ray absorption spectroscopy in transmission mode was performed at the Stanford Synchrotron Radiation Lightsource beamline 9-3 (3 GeV, 500 mA, top-off mode) using a Si(220) liquid-nitrogen-cooled double crystal monochromator. All samples were measured at 10 K in a liquid helium flow cryostat. The XAS measurements were collected in step-scan mode from 6,830.8 to 7,095.8 in 5 eV steps, from 7,096.0 to 7,140.0 in 0.2 eV steps, and at constant steps in *k* space up to 7,988 eV. Electronic absorption spectra were recorded in solution on a Shimadzu double-beam UV-3600 UV/Vis/NIR spectrophotometer at room temperature. Electrochemical measurements were performed at room temperature under a dinitrogen atmosphere with an µAutolab Type III potentiostat using a rotating disk electrode with a glassy carbon or platinum tip (3 mm diameter) as working electrode and platinum wires as counter and pseudo-reference electrodes referenced to the Fc^+^/Fc couple. Elemental analyses were obtained using Euro EA 3000 (Euro Vector) and EA 1108 (Carlo-Erba) elemental analysers at the Friedrich-Alexander-Universität Erlangen-Nürnberg. See the Supplementary Information for in-depth details on the parameters of each spectroscopic set-up.

### Starting materials

Commercially available starting materials were purchased from commercial suppliers (Acros Organics, Alfa Aesar, Sigma-Aldrich, Merck, TCI, VWR) and were used without further purification. 1-Mesitylimidazole, *tris*-(chloromethyl)amine, *tris*-[(3-mesityl-imidazol-2-ylidene)-methyl]amine (TIMMN^Mes^) and [(TIMMN^Mes^)Fe^IV,V^(N)]^1+,2+^ (**I′**/**I**) were synthesized as reported in the literature^17,45–47^.

### Synthetic procedures

#### [(TIMMN^Mes^)Fe^VI^(N)(F)](PF_6_)_2_ (1)

Excess (3–4 equiv.) silver difluoride (AgF_2_) was added, under the exclusion of light, to a solution of [(TIMMN^Mes^)Fe^V^(N)](PF_6_)_2_ (**I**) (400 mg, 0.41 mmol, 1.0 equiv.) in dichloromethane (10 ml). The reaction mixture was vigorously stirred for 1 h at room temperature, whereupon a gradual colour change from intensively yellow–orange to lime-green occurred. This colour change is accompanied by the formation of a green precipitate of the crude [(TIMMN^Mes^)Fe^VI^(N)(F)](PF_6_)_2_ complex **1**. The resulting dark green solid was filtered off, washed with dichloromethane (4 × 2 ml), redissolved in acetonitrile, and extracted from the remaining AgF_2_ excess by filtration. Subsequent drying in vacuo yielded 350 mg (85%) of lime-green powdery complex **1** used for spectroscopic analysis. Single-crystals suitable for X-ray diffraction analysis were obtained by slow diffusion of diethyl ether into an acetonitrile/dichloromethane mixture at –35 °C overnight.

^1^H NMR (400 MHz, CD_3_NO_2_, +23 °C) *δ* 7.76 (*s*, 3 H, Im-*H*), 7.56 (*s*, 3 H, Im-*H*), 7.01 (*s*, 2 H, Ar-*H*), 6.99 (*s*, 2 H, Ar-*H*), 6.67 (*s*, 2 H, Ar-*H*), 5.86 (*s*, 2 H, N–C*H*_2_), 5.70 (*d*, |^2^*J*| = 11.9 Hz, 2 H, N–C*H*_2_), 5.38 (*d*, |^2^*J*| = 11.9 Hz, 2 H, N–C*H*_2_), 2.34 (*s*, 6 H, C*H*_3_), 2.32 (*s*, 6 H, C*H*_3_), 2.23 (*s*, 3 H, C*H*_3_), 1.97 (*s*, 6 H, C*H*_3_), 1.40 (*s*, 6 H, C*H*_3_). ^13^C NMR (101 MHz, CD_3_CN, +23 °C *δ* 167.82 (2C, Fe–*C*N_2_), 157.26 (*d*, ^2^*J*_C,F_ = 29.0 Hz, 1 C, Fe–*C*_*trans*_N_2_), 141.85, 141.64, 136.61, 136.48, 135.68, 134.86 and 133.02 (12C, Ar-*C*), 133.67, 130.82, 130.38, 130.18, 129.37, 124.17, and 123.74 (12C, Ar-*C*H and Im-*C*H), 73.36 (1C, N–*C*H_2_), 72.32 (2C, N–*C*H_2_), 21.11, 21.06, 19.08, 18.61 and 17.52 (9C, *C*H_3_). ^15^N NMR (41 MHz, CD_3_CN, +23 °C) *δ* 1057.5 (*d*, ^2^*J*_N,F_ = 22 Hz, Fe≡*N*). ^19^F NMR (376 MHz, CD_3_CN, +23 °C) *δ* –72.67 (*d*, ^1^*J*_F,P_ = 706.6 Hz, (P*F*_6_^–^)), –309.54 (*s*, Fe–*F*). ^31^P NMR (162 MHz, CD_3_CN, +23 °C) *δ* –141.55 (septet, ^1^*J*_P,F_ = 706.6 Hz, (*P*F_6_^–^)). Elemental analysis (calc., found for C_39_H_45_FeN_8_P_2_F_13_·1CH_2_Cl_2_): C (44.67, 44.74), H (4.40, 4.28), N (10.42, 10.45).

#### [(TIMMN^Mes^)Fe^VII^(N)(F)](PF_6_)_2_(PF_6_/MF_6_), M = Mo, Re (2)

The following procedures have been applied for freeze–quench spectroscopic experiments (^57^Fe Mössbauer, EPR and X-ray absorption spectroscopy). Route 1: excess (3–5 equiv.) XeF_2_ and sodium hexafluorophosphate (7 mg, 0.04 mmol, 1.0 equiv.) were added to a pre-cooled solution (−30 °C) of [(TIMMN^Mes^)Fe^VI^(N)(F)](PF_6_)_2_ (**1**) (40 mg, 0.04 mmol, 1.0 equiv.) in acetonitrile (1 ml). The reaction mixture was vigorously stirred for a few minutes at −30 °C, whereupon a gradual colour change from intensively lime-green to orange–red occurred. This mixture was immediately frozen and analysed spectroscopically. Note: a similar observation was made using XeF(SbF_6_) in chlorofluoromethane at −60 °C. Herein, an immediate colour change from lime-green to red occurred. Route 2: [(TIMMN^Mes^)Fe^VI^(N)(F)](PF_6_)_2_ (**1**) (20 mg, 0.02 mmol, 1.0 equiv) was placed inside a PFA tube (8 mm outer diameter) equipped with a stainless-steel valve and connected to a stainless-steel vacuum line. The PFA tube was cooled to −196 °C with liquid nitrogen, and ∼1.0 ml of SO_2_ (which is highly oxidation stable) was condensed onto **1**. The tube was placed in a −10 °C ethanol cooling bath to liquefy SO_2_. Afterwards, the PFA tube was again cooled to −196 °C, and an excess of MF_6_ (20–30 mg) was condensed into the reaction vessel. The PFA tube was then placed again in a cold ethanol bath. For ReF_6_, a temperature of −60 °C was already sufficient to observe a colour change to orange–red within minutes, whereas, in the case of MoF_6_, the reaction temperature was carefully raised successively to −10 °C. After approximately 10 min, all volatiles were removed as much as possible in vacuum in the cold (−60 °C, 10^–3^ mbar); however, at the end, warming to room temperature for 1–2 min was needed to ensure complete removal of all volatiles to give an orange–red solid. After removal of all volatiles, the PFA tubes were flame-sealed in vacuum and the samples were placed in a cryoshipper (liquid nitrogen temperature) and sent from Berlin to Erlangen/Mülheim for further spectroscopic analysis. Note: the first route is preferred for monitoring the formation, rearrangement and conversion of **2** by freeze–quench studies with ^57^Fe Mössbauer and X-band EPR spectroscopy, whereas the second route enables handling/removal of reactants at lower temperatures (−60 °C, 10^–3^ mbar) and, therefore, allows for a solid-state ^57^Fe Mössbauer and X-ray absorption spectroscopic analysis. Note: the intramolecular rearrangement of **2** to **3** occurred independently from the solvent used in each route, as a fraction of acetonitrile remains within **1** following extraction of [(TIMMN^Mes^)Fe^VI^(N)(F)](PF_6_)_2_ from AgF_2_ (see above).

#### [(TIMMN^Mes*^)Fe^V^(=N^*^)(NCMe)](PF_6_)_2_(MF_6_), M = Mo, Re (3)

Complex **3** was prepared in analogy to the freeze–quench experiments following the synthesis of **2**. Accordingly, [(TIMMN^Mes^)Fe^VI^(N)(F)](PF_6_)_2_ (**1**) (20 mg, 0.02 mmol, 1.0 equiv.) was placed inside of a PFA tube equipped with a stainless-steel valve and connected to a stainless-steel vacuum line, cooled to −196 °C with liquid nitrogen, and ∼1.0 ml of SO_2_ was condensed onto **1**. The tube was placed in a −10 °C ethanol cooling bath to liquefy SO_2_. The PFA tube was then cooled again to −196 °C and excess MF_6_ (20–30 mg) was condensed onto the reactant. The PFA tube was then placed again in a cold ethanol bath and stirred for 15 min. Removal of all volatiles at −10 °C in vacuo produces an orange–brown, room-temperature-stable solid in quantitative yield, used for spectroscopic analysis. Single-crystals suitable for X-ray diffraction analysis of [(TIMMN^Mes*^)Fe^V^(=N^*^)(NCMe)](PF_6_)_2_(MF_6_), M=Mo, Re (**3**) can be obtained from acetonitrile/diethyl ether or acetonitrile/toluene mixtures. Elemental analysis (calc., found for C_41_H_47_FeN_9_P_2_MoF_18_·1C_7_H_8_·1.5CH_3_CN): C (44.72, 44.65), H (4.38, 4.51), N (10.69, 10.56). See the main text and Supplementary Information for additional in-depth spectroscopic characterization data of each compound.

Note: the coincidentally obtained single-crystals suitable for X-ray diffraction analysis of [(TIMMN^Mes*^)Fe^V^(NH^*^)(F)](PF_6_)_3_ (**2e**) were obtained only once (analogusly to the reaction procedure of **3**) by changing the crystallization conditions to an SO_2_/HF mixture, thus, potentially hindering the HF elimination mechanism.

### Crystal structure determination

Suitable single-crystals of the investigated compounds were embedded in protective perfluoropolyalkyether oil on a microscope slide, and a single specimen was selected and subsequently transferred to the cold nitrogen gas stream of the diffractometer. Intensity data were collected using Mo*K*_α_ radiation (*λ* = 0.71073 Å) on a Bruker Kappa PHOTON 2 IμS Duo diffractometer equipped with QUAZAR focusing Montel optics. Data were corrected for Lorentz and polarization effects, semi-empirical absorption corrections were performed on the basis of multiple scans using SADABS^48^. The structures were solved by direct methods (SHELX XT 2014/5)^49^ and refined by full-matrix least-squares procedures on *F*^2^ using SHELXL 2018/3 (ref. ^50^). All non-hydrogen atoms were refined with anisotropic displacement parameters. All hydrogen atoms were placed in positions of optimized geometry, their isotropic displacement parameters were tied to those of the corresponding carrier atoms by a factor of either 1.2 or 1.5. Olex2 was used to prepare material for publication^51^ (see Supplementary Information for crystallographic data, data collection and structure refinement details).

### Computational details

All computations were performed with ORCA v.5.0.1‒4 (refs. ^37–39^^,52^). The NPA charges were calculated with NBO v.7.0 (ref. ^53^), whereas the Bader charges were calculated with AIMAll v.19.10.12 (ref. ^54^). Orbitals and spin densities were visualized with IboView v.20211019-RevA^55^ or ChemCraft^56^. See the Supplementary Information for further details on DFT and CASSCF calculations.

## Online content

Any methods, additional references, Nature Portfolio reporting summaries, source data, extended data, supplementary information, acknowledgements, peer review information; details of author contributions and competing interests; and statements of data and code availability are available at 10.1038/s41557-023-01418-4.

### Supplementary information

Supplementary Information Full Supplementary Information file (without *xyz* data, as requested).

Supplementary Data 1 Crystallographic data for compound 1; CCDC reference no. 2258913.

Supplementary Data 2 Crystallographic data for complex (intermediate) 2e; CCDC reference no. 2297600

Supplementary Data 3 Crystallographic data for complex 3; CCDC reference no. 2258914

Supplementary Data 4 The *xyz* coordinates for the optimized structures

### Source data

Source Data Fig. 1 Zero- and applied-field Moessbauer and XAS data shown in Fig. 1.

Source Data Fig. 2 Zero-field Moessbauer and X-band EPR data shown in Fig. 2.

Source Data Fig. 3 Plots of isomer shift versus oxidation state and partial charge and XAS data shown in Fig. 3.

## Data Availability

All data generated and analysed during this study are included in this article and its Supplementary Information, and are also available from the authors on reasonable request. Atomic coordinates and structure factors for the reported crystal structures have been deposited in the Cambridge Crystallographic Database under accession codes CCDC-2258913 for [(TIMMN^Mes^)Fe^VI^(N)(F)](PF_6_)_2_·CH_2_Cl_2_ (**1**·CH_2_Cl_2_); CCDC-2297600 for [(TIMMN^Mes*^)Fe^V^(NH^*^)(F)](PF_6_)_3_ (**2e**); and CCDC-2258914 for [(TIMMN^Mes*^)Fe^V^(=N^*^)(NCMe)](PF_6_)_2_MoF_6_·5 C_2_H_3_N (**3**·5 C_2_H_3_N). Source Data are provided with this paper.
