# Supplementary material for: The synthesis and characterization of an iron(VII) nitrido complex
Source: Nat Chem. 2024 Jan 30;16(4):514–20. doi: 10.1038/s41557-023-01418-4 (PMC10997499; doi:10.1038/s41557-023-01418-4)
Supplement: Supplementary file 1 — Full Supplementary Information file (without xyz data, as requested). [file 41557_2023_1418_MOESM1_ESM.pdf]

nature chemistry

Article https://doi.org/10.1038/s41557-023-01418-4

The synthesis and characterization of an iron(VII) nitrido complex

In the format provided by the authors and unedited

Table of Contents

General Methods 2
Synthesis and NMR Spectroscopic Characterization 5
UV/VIS/NIR Spectroscopy 9
EPR Spectroscopy 10
^{57}Fe Mössbauer Spectroscopy 11
Voltammetry 14
Fe K-edge X-Ray Absorption Spectroscopy 15
Crystallographic Details 24
Computational Details 32
References 62

General Methods

If not stated otherwise, all air- and moisture-sensitive experiments were performed under dry nitrogen atmosphere, using standard Schlenk techniques or an MBraun inert-gas glovebox, containing an atmosphere of purified dinitrogen. The glovebox was equipped with a –35 °C freezer.
Solvents were purified using a two-column solid-state purification system (Glass Contour System, Irvine, CA), transferred to the glovebox without exposure to air and moisture, and stored over activated molecular sieves (3 Å) for all halogenated-, nitro- or nitrile-containing solvents (dichloromethane, chloroform, acetonitrile, and nitromethane). Additionally, potassium was added to alkanes, aromatic solvents and cyclic ethers (*n*-pentane, *n*-hexane, benzene, toluene, and tetrahydrofuran). Commercially available starting materials were purchased from commercial suppliers (Acros Organics, Alfa Aesar, Sigma Aldrich, Merck, TCI, VWR) and were used without further purification. NMR solvents were obtained packaged under argon and stored over activated molecular sieves[1].
CAUTION: Sulfur dioxide (SO_2) is a toxic gas (boiling point −10 °C) and was stored in a stainless-steel cylinder over CaH_2. Anhydrous HF (boiling point +19 °C) is a highly toxic and corrosive gas. It was purified by distillation and stored in a stainless-steel cylinder. MoF_6 and ReF_6 (ABCR) are highly volatile liquids. They are corrosive and strongly oxidizing. They were also stored in stainless-steel cylinders. Liquid SO_2 is an aprotic, polar, and highly oxidation stable (>2 Volt *vs.* Cp_2Fe) solvent with low nucleophilicity[2]. Due to its liquid range between –75°C and –10°C, it is well suited for low-temperature reactions and can be removed conveniently in a vacuum. While the compounds and reagents used in this study have excellent solubility in SO_2, SO_2 does not react with MoF_6 or ReF_6; these reagents do, however, react with common solvents, such as CH_2Cl_2. Regarding safety precautions, reactions involving SO_2, HF, MoF_6 and ReF_6 are conducted with small amounts (1–2 ml of SO_2) in closed setups (steel vacuum line and reaction tubes made from the perfluorinated tetrafluoroethene-perfluoroalkoxyvinyl-copolymer (PFA)) in a regular fume hood. Attention has to be paid to the vapor pressure of SO_2 at room temperature (3 bar). Although working in glassware is possible, it is recommended to use metal/PFA setups instead.

Elemental Analyses were obtained using Euro EA 3000 (Euro Vector) and EA 1108 (Carlo-Erba) elemental analyzers in the Chair of Inorganic and General Chemistry at the Friedrich-Alexander-Universität Erlangen-Nürnberg (FAU). Commercial laboratories refuse samples that contain fluorine as it damages the modern elemental analyzers. Besides this practical problem, fluorine often causes substantial variations from the expected/calculated values.

Electronic absorption spectra were recorded from $\lambda = 200$ nm to $\lambda = 2200$ nm on a Shimadzu double-beam UV-3600 UV/Vis/NIR spectrophotometer in given solvents at room temperature. Cuvettes of the type 117.100-QS from Hellma® with 10 mm layer thickness were used. Data points between 1700 and 1750 nm are obscured due to the switch from the In/Ga/As detector to the PbS detector of the instrument and were removed from the graphical representation.

^{1}H and ^{13}C NMR spectra were recorded on a JEOL ECZ 400S instrument operating at 399.79 MHz for ^{1}H and at 100.53 MHz for ^{13}C at a probe temperature of 23 °C if not stated otherwise. The solvent residual signals of incomplete deuterated solvent molecules were used as internal reference for the ^{1}H NMR spectra and the solvent signals for ^{13}C NMR spectral data[3]. For **^{15}N, ^{19}F, and ^{31}P NMR data**, chemical shifts were referenced by the Delta v5.0.5 software provided by JEOL Ltd NMR. In detail, for ^{15}N NMR shifts the program considers liquid ammonia as a reference. NMR multiplicities are abbreviated as follows: s = singlet, d = doublet, t = triplet, q = quartet, m = multiplet, b = broad. Coupling constants *J* are given in Hz.

EPR spectra were recorded on a JEOL continuous wave spectrometer, JES-FA200, equipped with an X-band Gunn diode oscillator bridge, a cylindrical mode cavity, and a helium cryostat. If not stated otherwise, the samples were measured in the corresponding solvent in approx. 1 mM concentration under nitrogen atmosphere in air-tight J. Young quartz glass EPR tubes at 293, 95, and 7 K. Directly upon exiting the glovebox, the samples were frozen in liquid nitrogen. The spectra were recorded using the following parameters: microwave frequency = 8.959 GHz, modulation width 1.0 mT, microwave power 1.0 mW, modulation frequency 100 kHz, time constant of 0.1 s. Data analysis and simulation was performed using the software “eview” and “esim”, written by Dr. Eckhard Bill (MPI CEC, Mülheim/Ruhr), on the basis of a spin-Hamiltonian description of the electronic ground state:

$$\hat{H} = D\left(\hat{S}_z^2 - \frac{1}{3}S(S+1) + \frac{E}{D}\left(\hat{S}_x^2 - \hat{S}_y^2\right)\right) + \mu_B \underline{g} \vec{S}\ .$$

Here, S represents the total spin quantum number of the coupled system, D and E/D are the axial and rhombic zero-field parameters, respectively, and $\underline{g}$ is the g-matrix. Calculations are based on the $S = 5/2$ routines developed by Gaffney and Silverstone[4]. EPR line widths, W, are given in units of mT at full-width-half-maximum (FWHM).

Zero-field ^{57}Fe-Mössbauer spectra were recorded on a WissEl Mössbauer spectrometer (MRG-500) at a temperature of 77 K in constant acceleration mode. ^{57}Co/Rh was used as γ-radiation source. WinNormos for Igor Pro software was used for the quantitative evaluation of the spectral parameters (least-squares fitting to Lorentzian peaks). The minimum experimental line widths were 0.21 mm s^{-1} (full width at half maximum, FWHM). The temperature of the samples was controlled by a MBBC-HE0106 MÖSSBAUER He/N_2 cryostat within an accuracy of ±0.3 K. Least-square fitting of the Lorentzian signals was carried out with the Mfit software, developed by Dr. Eckhard Bill (MPI Mülheim/Ruhr). Isomer shifts are reported relative to α-iron at 300 K.

Applied-field ^{57}Fe-Mössbauer spectra were recorded with alternating constant acceleration of the γ-source (lab-built spectrometer, MPI-CEC). The minimum experimental line width was 0.24 mm s^{-1} (full width at half maximum, FWHM). The sample temperature was maintained constant in a cryogen-free, closed-cycle Mössbauer magnet cryostat from Cryogenic Ltd. The latter is a split-pair super-conducting magnet system for applied fields up to 7 T. The temperature of the sample can be varied in the range 1.7 K to 300 K. The field at the sample is perpendicular to the γ-beam. The ^{57}Co/Rh source (1.8 GBq) was positioned from outside at room temperature at a zero-field position inside the gap of the magnet, by using a re-entrant bore tube. The detector was a Si-Drift diode (150 mm^2 SDD CUBE) of an AXAS-M1 instrument from Ketek GmbH with vacuum-tight 200 mm stainless steel finger, which was inserted into the cryostat to position the diode also in the gap of the magnet. Isomer shifts are reported relative to a-iron metal at 300 K. The magnetic Mössbauer spectra were simulated with the program *mx.SL* (Dr. Eckhard Bill (MPI Mülheim/Ruhr)) by using the usual nuclear Hamiltonian for ^{57}Fe for $S = 0$, or by diagonalization of the corresponding electronic spin Hamiltonian for $S = 1/2$.

Electrochemical measurements were carried out at room temperature under dinitrogen atmosphere with an μAutolab Type III potentiostat. Samples were recorded in 1 mM solutions of the analyte in acetonitrile, tetrahydrofuran or dichloromethane containing 0.1 M $N(n\text{-Bu})_4PF_6$ ($TBAPF_6$, ≥99.0% for electrochemical analysis, purchased from Sigma Aldrich and used without further purification) using a rotating disk electrode with a glassy carbon, platinum or gold tip (3 mm diameter) as working electrode and platinum wires as counter and pseudo-reference electrodes. Ferrocene (Fc) was added as an internal standard and all measurements were referenced to the Fc^+/Fc couple.

Samples for X-ray absorption spectroscopy were prepared by grinding approximately 25–30 mg of pure crystalline compounds (**1**, **3**) with 50–60 mg of dry boron nitride (Sigma Aldrich) in a glovebox with an agate mortar and pestle to achieve 1.5–2.0% mass fraction of iron. The resulting powder was packed into 1-mm thick aluminium cells previously cleaned with aqueous EDTA (pH 7) overnight and sealed with 38-μm thick Kapton tape. Given the impossibility to isolate **2**, the sample was prepared by freeze drying an oxidation reaction of **1** with rhenium hexafluoride and subsequently grinding it at −50 °C. The resulting powder was packed under pre-cooled conditions, analogously to **1** and **3**, into a 1-mm thick aluminium cell with a fraction of it used for ^{57}Fe Mössbauer spectroscopy. This enabled XAS measurements and speciation by ^{57}Fe Mössbauer on the same sample in order to find the ratio between **2** and **3**. The samples were stored at cryogenic temperatures and mounted on the cryostat holder under liquid nitrogen.

X-ray absorption spectroscopy in high-energy resolution fluorescence-detected mode (Kβ-HERFD) was performed at the European Synchrotron Radiation Facility (ESRF) beam line ID26 (6 GeV, 30 mA, 16-bunch mode) using a Si(311) liquid nitrogen cooled double crystal monochromator calibrated versus the first inflection point of metallic Fe foil at 7111.2 eV. Silicon-coated mirrors were used to focus the beam to 200×100 μm (h×v), providing nominal flux of ~1×10^{13} photons/s. To avoid radiation-induced spectral changes, aluminium foils of 20 μm each were used to attenuate the incident flux when necessary. The measurements were conducted at 30 K in a liquid He cryostat, with the samples positioned at 45° relative to the incident beam. The emission spectrometer was equipped with five Si(531) spherically bent crystals (for Kβ at 7059 eV, Bragg angle: 73°) with 100 mm diameter and 1 m curvature radius in Rowland geometry. The flight path between the sample and the detector was filled with helium to mitigate the attenuation of emitted photons. The detector was a silicon avalanche photodiode with 200 μm thickness and 10×10 mm² active area, behind a slit with 1 mm opening. The emission spectrometer was set to the maximum of $K\beta_{1,3}$ line and the absorption spectrum was collected in continuous scan mode from 7080 to 7250 eV with integration set to give a 0.1 eV step size. Scans from 7020 to 7760 eV were collected for normalization.

In order to assess the radiation damage rate on each sample, quick near-edge XAS spectra were taken on the same spot until changes in consecutive spectra were observed. The changes overall were the K-edge shifting down in energy (denoting photoreduction) and intensity modulations in the pre-edge and rising edge regions. Multiple consecutive spectra without changes were used to determine the maximum dwell time on each spot of each sample, which in the sequence was set as the scan time. The final data was composed of an average of scans on 60–80 spots on the surface of a single sample.

X-ray absorption spectroscopy in transmission mode was performed at the Stanford Synchrotron Radiation Lightsource (SSRL) beam line 9-3 (3 GeV, 500 mA, top off mode) using a Si(220) liquid nitrogen cooled double crystal monochromator calibrated versus the first inflection point of metallic Fe foil at 7111.2 eV. A Rh-coated mirror in combination with 30% detuning of the monochromator was utilized for rejection of higher harmonics. N_2 filled ionization chambers were utilized as detectors before and after the sample and the metal reference foil. The spot size at the sample was ~1×4 mm with a flux of ~1×10^{12} photons/s. All samples were measured at 10 K in a liquid helium flow cryostat. Aluminium foil (0.25 mm thick) was used to attenuate the incoming flux whenever necessary to avoid photoreduction, and multiple scans on the same spot was collected to ensure that final data contained no radiation-induced spectral distortions. The XAS spectra were collected in step-scan mode from 6830.8 to 7095.8 in 5 eV step, from 7096.0 to 7140.0 in 0.2 eV steps and at constants steps in k space up to 7988 eV.

The spectral deconvolution to yield the K-edge X-ray absorption spectrum of **2** shown in Figure 3C and Supplementary Figure 14 (purple trace) followed the composition found by ^{57}Fe Mössbauer spectroscopy (Supplementary Figure 7, bottom) on an independently analyzed sample of the same batch. Therefore, 77% of the spectrum obtained from a pure sample of **3** (Supplementary Figure 16) were subtracted from the experimental spectrum, which contained a mixture of **2** and **3** (Supplementary Figure 15). The content of **2** (23%) is lower than the optimized sample preparations for Mössbauer spectroscopy shown in Figure 2A (64% of **2**), due to small periods of increased temperature to remove the solvent in the preparation of the solid XAS sample, leading to a rapid conversion of **2** to **3**. The resulting difference spectrum was renormalized to an edge jump of 1.

The procedures described above for XAS sample preparation, Fe K-edge $K\beta_{1,3}$-HERFD XAS, and Fe K-edge XAS in transmission mode are the same as reported previously[5].

K-edge X-ray absorption calculations were performed in ORCA 5.0.3[6-8] with time-dependent density functional theory (TDDFT) as previously described[9], employing the Tamm-Dancoff approximation[10], the PBE0[11-13] exchange-correlation functional, resolution of identity and approximation[14,15] and "chain-of-spheres" RIJCOSX for the Hartree-Fock exchange calculation[16-19]. Scalar relativistic effects were accounted by means of the zeroth-order regular relativistic approximation (ZORA)[20]. The all-electron recontracted triple-zeta basis set ZORA-def2-TZVPP[21] was employed together with the SARC/J[22] general-purpose auxiliary basis set for Coulomb fitting. The effects of implicit solvation by acetonitrile were included by the conductor-like continuum polarizable model (CPCM) with Gaussian charge scheme[23] and SMD solvation module[24]. Dispersion interactions were accounted with Becke-Johnson damping (D3BJ)[25,26]. The structural parameters of **1** and **3** relate to the ones obtained by SC-XRD after optimization of the H-atoms (see computational detail section). The structure for **2** was obtained by full optimization at this same level of theory of a model in which the mesityl groups were replaced by phenyl groups, and three PF_6^- counterions were explicitly included. The SCF convergence criteria were set to "TightSCF". The oscillator strength was taken as the sum of electric dipole, electric quadrupole and magnetic dipole contributions. The assignment of the electronic transitions were based on the compositions of the canonical receptor molecular orbitals and by inspecting the receptor natural transition orbitals[27], whose plots were produced by VMD[28]. The plot in Figure 1D was produced with orca_mapspc utility with 1.3 eV line width and Gaussian line shape. An energy shift of +17.0 eV and a scaling factor of 0.05 ware applied to match the pre-edge peak with the experimental data.

Fe(II)Cl_2×1.5THF, 1-Mesitylimidazole, *tris*-(chloromethyl)amine, ***tris***-[(3-mesityl-**im**idazol-2-ylidene)-**m**ethyl]-ami**n**e (TIMMN^{Mes}) and [(TIMMN^{Mes})$Fe^{IV,V}$(N)]$^{1+,2+}$ (**I'/I**) were synthesized as described in literature[29-32].

Synthesis and NMR Spectroscopic Characterization

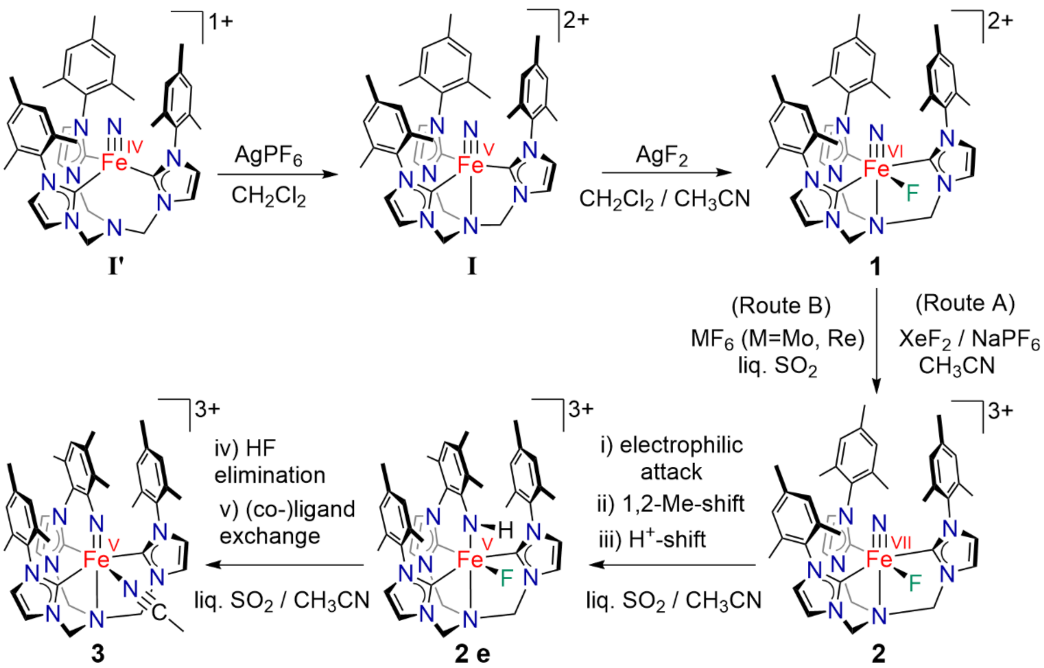

Supplementary Figure 1 | Synthesis of the nitrido complexes **1** and **2** and the cyclization product **3** *via* the Fe(V) amide intermediate **2e**.

H_3TIMMN^{Mes}(PF_6)$_3$: In a 250 ml Schlenk flask, solid white crystals of *tris*-(chloromethyl)amine[31] (3.8 g, 23.4 mmol, 1 equiv) were treated with a concentrated solution of 1-mesitylimidazole[29] (14.0 g, 75.2 mmol, 3.2 equiv) in dichloromethane (DCM). Immediate increase of temperature and bubbling indicated initiation of the S_N2 reaction, which is complete within minutes. The imidazolium chloride salt was found to be highly hygroscopic and sensitive to moisture. Therefore, the mixture was evaporated to dryness on the rotary evaporator as quickly as possible, and treated with a solution of ammonium hexafluorophosphate (15.3 g, 93.9 mmol, 4 equiv) in methanol (MeOH). The resulting suspension is vigorously stirred overnight. Finally, filtration, washing with MeOH (3×30 mL) and drying in high vacuum yielded the imidazolium salt, H_3TIMMN^{Mes}(PF_6)$_3$, as off-white solid (19.3 g, 18.4 mmol, 79 % over two steps)[32].

^{1}H NMR (270 MHz, DMSO-d_6) δ[ppm]: 9.51 (s, 3H), 8.11 (s, 3H), 8.04 (s, 3H), 7.19 (s, 6H), 5.84 (s, 6H), 2.35 (s, 9H), 2.02 (s, 18H)[32].

[(TIMMN^{Mes})Fe^{II}(N_3)](PF_6): To a thawing slurry of H_3TIMMN^{Mes}(PF_6)$_3$ (10 g, 9.6 mmol, 1.0 equiv) in 50 mL of THF, lithium *bis*(trimethylsilyl)amide (5.2 g, 31.1 mmol, 3.3 equiv) and $FeCl_2$·1.5 THF[30] (2.1 g, 9.0 mmol, 0.95 equiv) were added under vigorous stirring. Upon addition of base and warming to ambient temperature, the mixture cleared up and turned yellowish. This marks the instant generation of the highly unstable TIMMN^{Mes} free carbene, which requires the simultaneous presence of the iron precursor ($FeCl_2$·1.5 THF[30]). After approximately 5 minutes, the mixture turns more and more cloudy with fine white precipitate, while stirring was continued overnight. Finally, the white solid was filtered off and washed with THF, diethyl ether and pentane. Subsequent drying *in vacuo* yields 7.2 g (94 %) of [(TIMMN^{Mes})Fe^{II}Cl](PF_6) and [(TIMMN^{Mes})Fe^{II}](PF_6)$_2$. The crude product was purified by vapor diffusion of diethyl ether into a MeCN solution, yielding a white solid powder and a light-yellow solvent phase, which was separated from the product by filtration. Subsequently, [(TIMMN^{Mes})Fe^{II}Cl](PF_6) (450.4 mg, 0.53 mmol, 1 equiv) was dissolved in 15 mL of acetonitrile, and sodium azide (214.5 mg, 3.3 mmol, 6.2 eq.) was added, the vial was protected from light, and placed in the –30°C freezer for 48 h. After that, excess sodium azide and sodium chloride were separated from the reaction mixture by means of filtration, and the solution was evaporated to dryness. The remaining solid was slurried with diethyl ether and dried again, in order to extract residues of acetonitrile. Lastly, the product was extracted into THF and the colourless solution was evaporated to dryness, yielding 405 mg (90 %) of product as an off-white solid[32]. Single-crystals suitable for X-ray diffraction were obtained by slow diffusion of pentane into a saturated benzene solution of [(TIMMN^{Mes})Fe^{II}(N_3)](PF_6) at room temperature overnight[32].

^{1}H NMR (400 MHz, THF-d_8) δ[ppm]: 82.9 (bs, 6H), 37.6 (s, 3H), 32.5 (s, 3H), 9.2 (s, 6H), 6.5 (s, 9H), –11.8 (s, 18H)[32].

[(TIMMN^{Mes})Fe^{IV}(N)](PF$_6$) (I'): In a 300 ml borosilicate glass Schlenk flask, equipped with a magnetic stir bar, [(TIMMN^{Mes})Fe^{II}(N$_3$)](PF$_6$) (200 mg, 0.06 mmol, 1 eq.) was dissolved in 140 ml of THF. The colorless solution was taken out of the glovebox, onto a magnetic stir plate surrounded by four 365 nm DC UV light sources and irradiated under stirring. Irradiation was carried out for 40 hrs, within which the solution gradually changed its color from colorless to dark red. Lastly, the solution was evaporated to dryness *in vacuo* (2×10^{-3} mbar) and re-introduced into the glovebox. The dark red solid was dissolved in as little THF as possible and the dark red solution filtered through Celite®. Thereby, a brown precipitate was removed from the mixture. Purification was carried out using a hexane-toluene vapor diffusion setup at room temperature, yielding 170 mg (85 %) of the iron(IV) nitrido complex as dark red crystals[32]. Single-crystals suitable for X-ray diffraction analysis were obtained by diffusion of hexane into a saturated benzene solution of [(TIMMN^{Mes})Fe^{IV}(N)](PF$_6$) (**I'**) at room temperature over the course of several days. The corresponding ^{15}N-labelled compound for ^{15}N NMR spectroscopy was synthesized using labelled sodium azide in the same fashion, on a 50 mg scale[32].

^{1}H NMR (400 MHz, MeCN-d_3) δ [ppm]: 7.33 (s, 3H), 7.10 (s, 3H), 6.76 (s, 6H), 4.80 (s, 6H), 2.25 (s, 9H), 1.65 (s, 18H)[32].

^{13}C NMR (400 MHz, MeCN-d_3) δ [ppm]: 204.5, 138.9, 138.1, 136.0, 129.5, 127.1, 120.9, 65.0, 21.0, 18.0[32].

^{15}N NMR (400 MHz, MeCN-d_3) δ [ppm]: 1103.6 (*vs*. NH$_3$) / 723.1 (*vs*. CH$_3$NO$_2$)[32].

[(TIMMN^{Mes})Fe^{V}(N)](PF$_6$)$_2$ (I): [(TIMMN^{Mes})Fe^{IV}(N)](PF$_6$) (**I'**) (197.1 mg, 0.24 mmol, 1.0 equiv) was dissolved in 7 mL of cold DCM in a small vial. Under vigorous stirring, silver hexafluorophosphate (67.0 mg, 0.26 mmol, 1.1 equiv) was added, whereupon the mixture changed to a dark yellow colour immediately. Subsequently, stirring was continued for 10 minutes and the black precipitate was removed by filtration over Celite®. The solution was taken to dryness *in vacuo* and the solid washed with diethyl ether, yielding 213 mg (92%) of crude product as dark yellow to brownish solid[32]. For purification, the compound was recrystallized by diffusion of diethyl ether into a DCM solution of the complex, whereupon 189 mg (79 %) of product were obtained as thin brown needles. Single-crystals suitable for X-ray diffraction analysis were obtained by diffusion of diethyl ether into a pyridine solution of [(TIMMN^{Mes})Fe^{V}(N)](PF$_6$)$_2$ (**I**) at room temperature overnight[32].

^{1}H NMR (400 MHz, DCM-d_2) δ [ppm]: 25.9 (bs, 6H), 8.4 (s, 6H), 4.7 (s, 3H), 3.3 (s, 9H), 1.0 (s, 18H), –15.1 (s, 3H)[32].

[(TIMMN^{Mes})Fe^{VI}(N)(F)](PF$_6$)$_2$ (1): To a solution of [(TIMMN^{Mes})Fe^{V}(N)](PF$_6$)$_2$ (**I**) (400 mg, 0.41 mmol, 1.0 equiv) in dichloromethane (10 mL), excess (3–4 equiv) silver difluoride (AgF$_2$) was added under the exclusion of light. The reaction mixture was vigorously stirred for 1 h at room temperature, whereupon a gradual color change from intensively yellow-orange to lime-green occurred. This color change is accompanied by the formation of a green precipitate, which is the crude [(TIMMN^{Mes})Fe^{VI}(N)(F)](PF$_6$)$_2$ complex **1**. The resulting dark green solid was filtered off, washed with dichloromethane (4×2 mL), re-dissolved in acetonitrile, and extracted from the remaining AgF$_2$ excess by filtration. Subsequent drying *in vacuo* yields 350 mg (85%) of lime-green powdery complex **1** used for spectroscopic analysis. Single-crystals suitable for X-ray diffraction analysis were obtained by slow diffusion of diethyl ether into an acetonitrile/dichloromethane mixture at –35°C overnight.

^{1}H NMR (400 MHz, CD$_3$NO$_2$, +23 °C) δ [ppm]: 7.76 (s, 3 H, Im-*H*), 7.56 (s, 3 H, Im-*H*), 7.01 (s, 2 H, Ar-*H*), 6.99 (s, 2 H, Ar-*H*), 6.67 (s, 2 H, Ar-*H*), 5.86 (s, 2 H, N–C*H*$_2$), 5.70 (d, $|^2J|$ = 11.9 Hz, 2 H, N–C*H*$_2$), 5.38 (d, $|^2J|$ = 11.9 Hz, 2 H, N–C*H*$_2$), 2.34 (s, 6 H, C*H*$_3$), 2.32 (s, 6 H, C*H*$_3$), 2.23 (s, 3 H, C*H*$_3$), 1.97 (s, 6 H, C*H*$_3$), 1.40 (s, 6 H, C*H*$_3$).

^{13}C NMR (101 MHz, CD$_3$CN, +23 °C) δ [ppm]: 167.82 (2 C, Fe–*C*N$_2$), 157.26 (d, $^2J_{C,F}$ = 29.0 Hz, 1 C, Fe–C_{trans}N$_2$), 141.85, 141.64, 136.61, 136.48, 135.68, 134.86, and 133.02 (12 C, Ar-*C*), 133.67, 130.82, 130.38, 130.18, 129.37, 124.17, and 123.74 (12 C, Ar-*C*H and Im-*C*H), 73.36 (1 C, N–*C*H$_2$), 72.32 (2 C, N–*C*H$_2$), 21.11, 21.06, 19.08, 18.61, and 17.52 (9 C, *C*H$_3$).

^{15}N NMR (41 MHz, CD$_3$CN, +23 °C) δ [ppm]: 1057.5 (d, $^2J_{N,F}$ = 22 Hz, Fe≡*N*).

^{19}F NMR (376 MHz, CD$_3$CN, +23 °C) δ [ppm]: –72.67 (d, $^1J_{F,P}$ = 706.6 Hz, (PF_6^-)), –309.54 (s, Fe–*F*).

^{31}P NMR (162 MHz, CD$_3$CN, +23 °C) δ [ppm]: –141.55 (septet, $^1J_{P,F}$ = 706.6 Hz, (PF$_6^-$)).

EA (calc., found for C$_{39}$H$_{45}$FeN$_8$P$_2$F$_{13}$ •1CH$_2$Cl$_2$): C (44.67, 44.74), H (4.40, 4.28), N (10.42, 10.45).

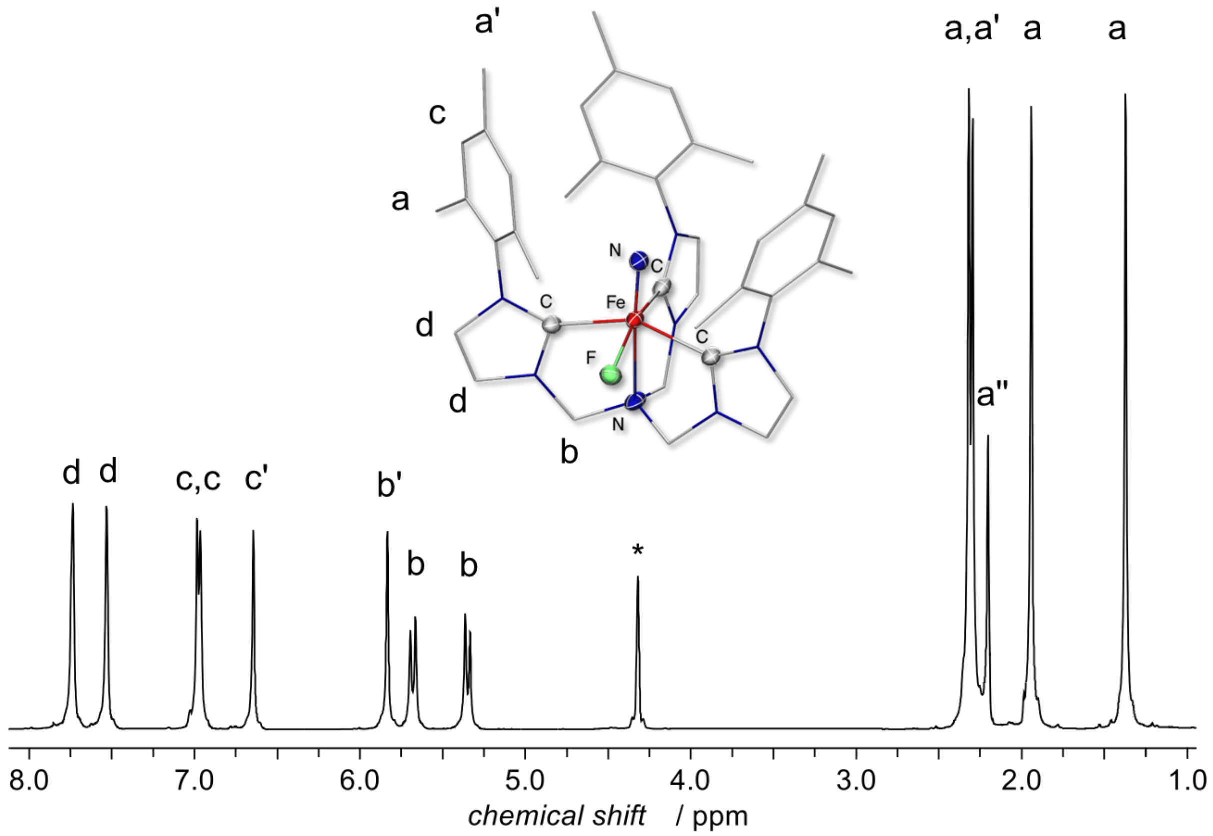

Supplementary Figure 2 | ^{1}H NMR spectrum of **1**, measured at 400 MHz in nitromethane-d_3 at room temperature. Solvent residual signal is marked with *.

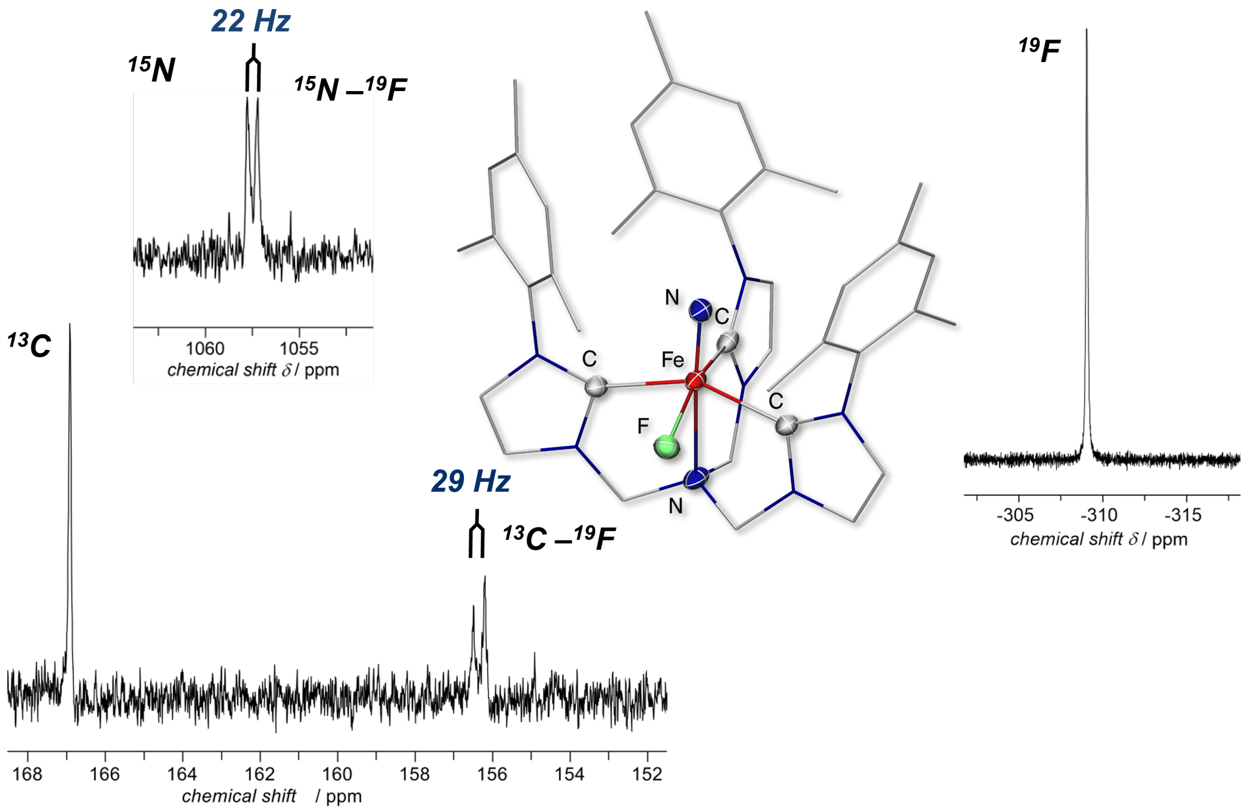

Supplementary Figure 3 | Multinuclear (^{13}C, ^{15}N, ^{19}F) NMR spectra of **1**, each measured at a different frequency (MHz) in acetonitrile-d_3 at room temperature.

[(TIMMN^{Mes})Fe^{VII}(N)(F)](PF$_6$)$_2$(PF$_6$/MF$_6$), M=Mo, Re (2): For freeze-quench spectroscopic experiments (^{57}Fe Mössbauer, EPR, and X-ray absorption spectroscopy), the following procedures have been applied. **A)** To a pre-cooled solution (−30 °C) of [(TIMMN^{Mes})Fe^{VI}(N)(F)](PF$_6$)$_2$ (**1**) (40 mg, 0.04 mmol, 1.0 equiv) in acetonitrile (1 mL), excess (3–5 equiv) xenon difluoride (XeF_2) and sodium hexafluorophosphate (7 mg, 0.04mmol, 1.0 equiv) were added. The reaction mixture was vigorously stirred for a few minutes at −30 °C, whereupon a gradual color change from intensively lime-green to orange-red occurred. This mixture was immediately frozen and analyzed spectroscopically. Note: A similar observation was made using XeF(SbF$_6$) in chlorofluoromethane at −60 °C. Herin, an immediate color change from lime green to red occurred. **B**) [(TIMMN^{Mes})Fe^{VI}(N)(F)](PF$_6$)$_2$ (**1**) (20 mg, 0.02 mmol, 1.0 equiv) was placed inside a PFA tube (8 mm outer diameter), equipped with a stainless-steel valve and connected to a stainless-steel vacuum line. The PFA tube was cooled to −196 °C with liquid nitrogen and ~1.0 ml of SO_2 was condensed onto **1**. The tube was placed in a −10 °C ethanol cooling bath to liquefy SO_2. Afterwards, the PFA tube was again cooled to −196 °C, and an excess of MF_6 (20–30 mg) was condensed into the reaction vessel. Subsequently, the PFA tube was placed again in a cold ethanol bath. In the case of ReF_6, a temperature of −60 °C was already sufficient to observe a color change to orange-red within minutes, whereas, in the case of MoF_6, the reaction temperature was carefully raised successively to −10 °C . After approximately 10 minutes, all volatiles were removed as far as possible in a vacuum in the cold (−60 °C, 10^{-3} mbar). However, in the end, warming to room temperature for 1–2 minutes was needed to ensure complete removal of all volatiles to give an orange-red solid. After the removal of all volatiles, the PFA tubes were flame-sealed in a vacuum, and the samples were placed in a cryo-shipper (liquid nitrogen temperature) and sent from Berlin to Erlangen/Mülheim for further spectroscopic analysis. Note: *Route A* is preferred to monitor the formation, rearrangement, and conversion of **2** by freeze-quench studies with ^{57}Fe Mössbauer and X-band EPR spectroscopy, whereas *route B* enables handling/removal of reactants at lower temperatures (−60 °C, 10^{-3} mbar), and therefore allows for a solid-state ^{57}Fe Mössbauer and X-ray absorption spectroscopic analysis. Note: The intramolecular rearrangement of **2** to **3** occurred independently from the solvent used in each route, as a fraction of acetonitrile remains within **1** upon extraction of [(TIMMN^{Mes})Fe^{VI}(N)(F)](PF$_6$)$_2$ from AgF_2 (*vide supra*).

[(TIMMN^{Mes*})Fe^{V}(=N*)(NCMe)](PF$_6$)$_2$(MF$_6$), M=Mo, Re (3): Complex **3** was prepared in analogy to the freeze-quench experiments upon the synthesis of **2**. Accordingly, [(TIMMN^{Mes})Fe^{VI}(N)(F)](PF$_6$)$_2$ (**1**) (20 mg, 0.02 mmol, 1.0 equiv) was placed inside a PFA tube equipped with a stainless-steel valve and connected to a stainless-steel vacuum line, cooled to −196 °C with liquid nitrogen and ~1.0 mL of SO_2 was condensed onto **1**. The tube was placed in a −10 °C ethanol cooling bath to liquefy SO_2. Afterwards, the PFA tube was again cooled to −196 °C, and excess of MF_6 (20–30 mg) was condensed onto the reactant. Subsequently, the PFA tube was placed again in a cold ethanol bath and stirred for 15 minutes. Removal of all volatiles at −10 °C *in vacuo* produces an orange-brown, room temperature stable solid in quantitative yield, used for spectroscopic analysis. Single-crystals suitable for X-ray diffraction analysis of [(TIMMN^{Mes*})Fe^{V}(=N*)-(NCMe)](PF$_6$)$_2$(MF$_6$), M=Mo, Re (**3**) can be obtained from acetonitrile/ diethyl ether or acetonitrile/toluene mixtures.

EA (calc., found for $C_{41}H_{47}FeN_9P_2MoF_{18}$ •1C_7H_8 •1.5CH_3CN): C (44.72, 44.65), H (4.38, 4.51), N (10.69, 10.56).

Note: Coincidentally obtained single-crystals suitable for sc-XRD analysis of [(TIMMN^{Mes*})Fe^{V}(NH*)(F)](PF$_6$)$_3$ (**intermediate 2e**) have been obtained only once by changing the crystallization conditions to an SO_2/HF mixture, thus potentially hindering the HF-elimination mechanism. These crystallization conditions could not be reproduced. Accordingly, we refrained from a discussion of bond metrics.

UV/VIS/NIR Spectroscopy

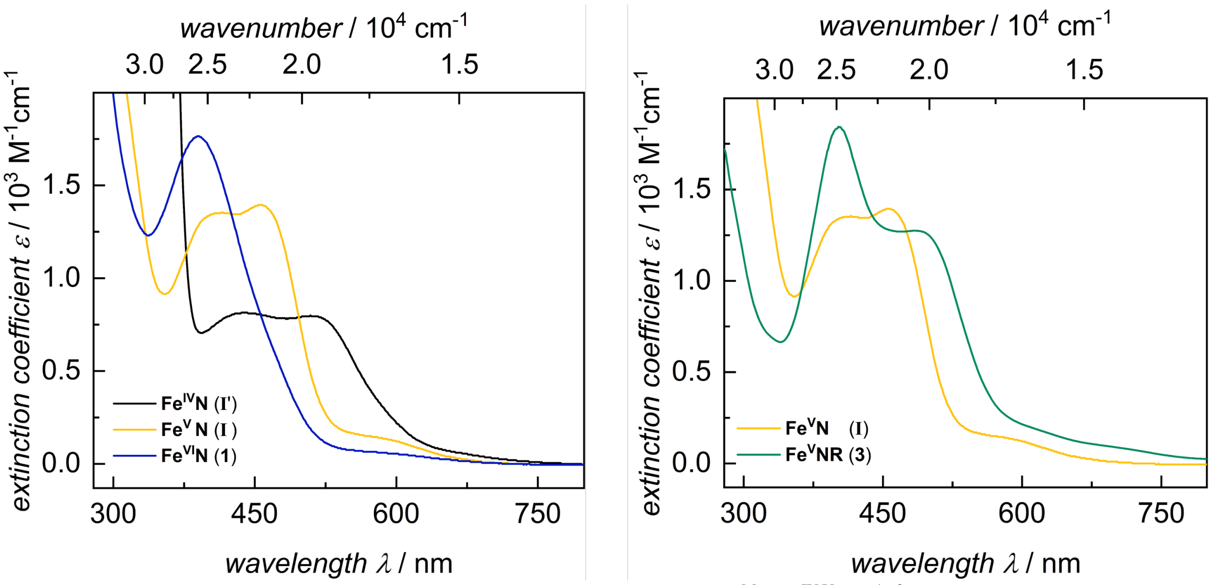

Supplementary Figure 4 | *Left*: Electronic absorption spectra of $[(TIMMN^{Mes})Fe^{IV,V}(N)]^{1+,2+}$ (**I'**/**I**) (black/yellow trace) and its oxidized congener $[(TIMMN^{Mes})Fe^{VI}(N)(F)]^{2+}$ (**1**) (blue trace). *Right*: Comparison of the electronic absorption spectra of $[(TIMMN^{Mes})Fe^{V}(N)]^{2+}$ (**I**) (yellow trace) and $[(TIMMN^{Mes*})Fe^{V}(=N^{*})(NCMe)]^{3+}$(**3**)

(**I'**, CH_3CN, nm ($M^{-1}cm^{-1}$): λ (ε)= 230 (9.383), 260 (4681), 340 (9956), 440 (819), 510 (800), 580 (321).

(**I**, CH_3CN, nm ($M^{-1}cm^{-1}$): λ (ε)= 230 (11.936), 260 (14553), 408 (1342), 455 (1406), 585 (131).

(**1**, CH_3CN, nm ($M^{-1}cm^{-1}$): λ (ε)= 230 (14.874), 260 (5796), 390 (1784), 590 (68).

(**3**, CH_3CN, nm ($M^{-1}cm^{-1}$): λ (ε)= 230 (11.839), 280 (11780), 400 (1840), 487 (1280), 620 (176), 711 (87).

EPR Spectroscopy

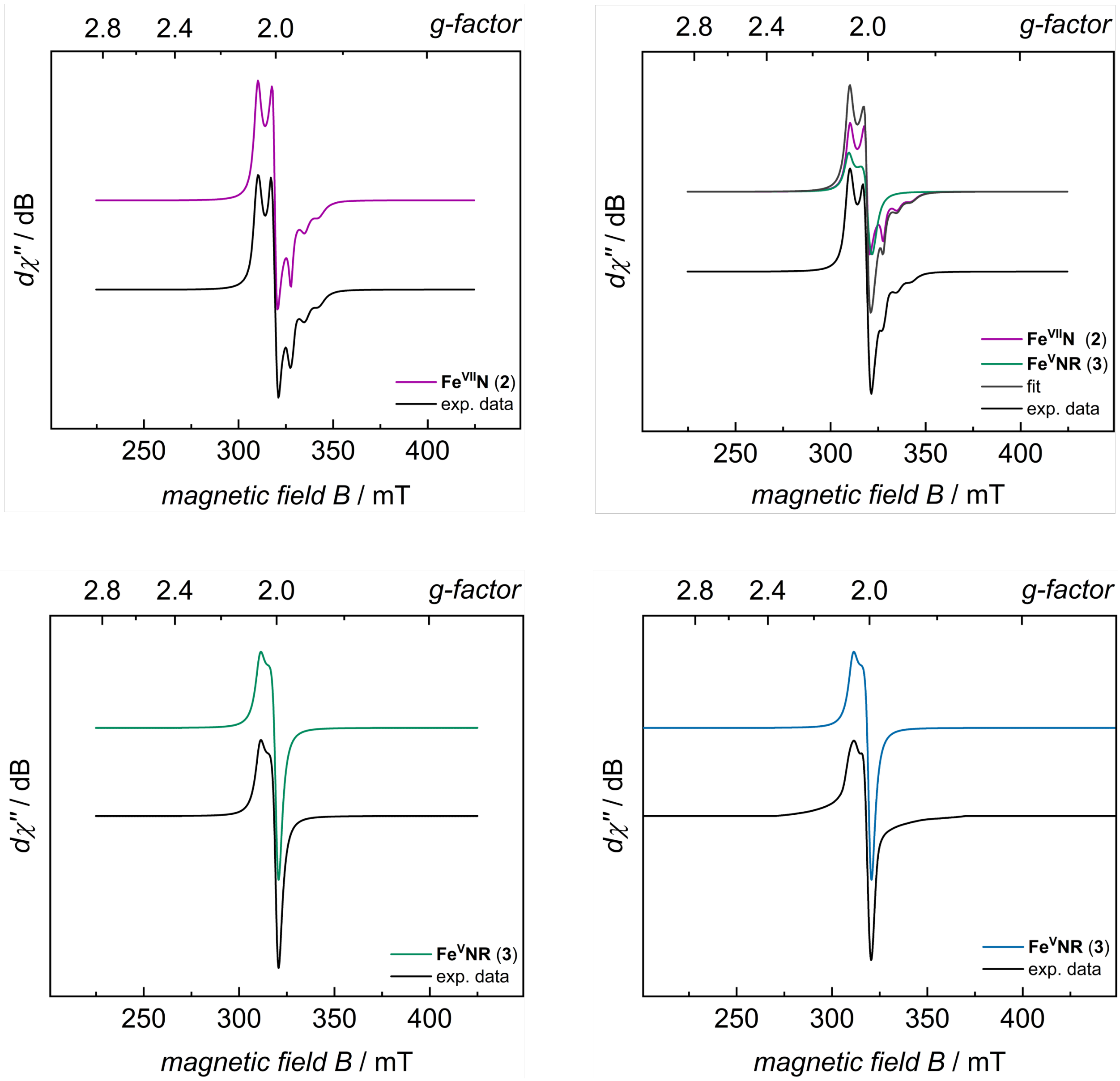

Supplementary Figure 5 | *Top and bottom (left):* Time-resolved X-band EPR formation, rearrangement, and complete transformation of **2**, measured at 15 K in a 1 mM frozen acetonitrile solution with XeF^{+} as the oxidant. Black lines represent the experimental data, and colored lines are the best fit with the parameters given below. Simulations show **a)** the rhombic $S = 1/2$ signal for initially formed **2** (purple trace, *top (left)*) with super-hyperfine coupling to the nitrido nitrogen, **b)** the rearrangement of **2** (purple trace, *top (right)*) to the slightly rhombic $S = 1/2$ signal of **3** (green trace, *top (right)*) with decreasing nitrogen super-hyperfine coupling, due to formation of the aryl-imido-metallacycle and **c)** the slightly rhombic $S = 1/2$ signal of **3** (green trace, *bottom (left)*) after complete transformation. *Bottom (right):* X-band EPR spectrum of **3** (blue trace), measured at 9 K in a 1 mM frozen acetonitrile solution, independently synthesized from **1** with MF_6 (M=Mo, Re) in liquid SO_2. The black line represents the experimental data, and the colored line are the best fit for the slightly rhombic $S = 1/2$ signal of **3**.

(**2** *(top, left)*, 15 K 8.944 GHz, CH_3CN): $g_1 = 2.058$, $g_2 = 1.998$; $g_3 = 1.908$; $W_1 = 2.7$ mT, $W_2 = 2.6$ mT, $W_3 = 2.2$ mT, $A_1 = 6.8$ mT (^{14}N, 99.63%, $I = 1$).

Note: The anisotropic g-values of **2** differ from values expected for an organic radical and further support a metal-centered oxidation.

(**2**/**3** *(top, right)*, 15 K 8.944 GHz, CH_3CN): (**2**, 65%) g_1 = 2.058, g_2 = 1.998; g_3 = 1.908; W_1 = 2.7 mT, W_2 = 2.6 mT, W_3 = 2.2 mT, A_1 = 6.8 mT (^{14}N, 99.63%, I = 1) and (**3**, 35%) g_1 = 2.065, g_2 = 2.000; g_3 = 1.984; W_1 = 3.8 mT, W_2 = 3.5 mT, W_3 = 3.5 mT.

(**3** *(bottom, left)*, 15 K 8.944 GHz, CH_3CN): g_1 = 2.065, g_2 = 2.000; g_3 = 1.984; W_1 = 3.5 mT, W_2 = 3.2 mT, W_3 = 3.2 mT.

(**3** *(bottom, right)*, 9 K 8.943 GHz, CH_3CN): g_1 = 2.065, g_2 = 2.000; g_3 = 1.984; W_1 = 3.5 mT, W_2 = 3.2 mT, W_3 = 3.2 mT.

^{57}Fe Mössbauer Spectroscopy

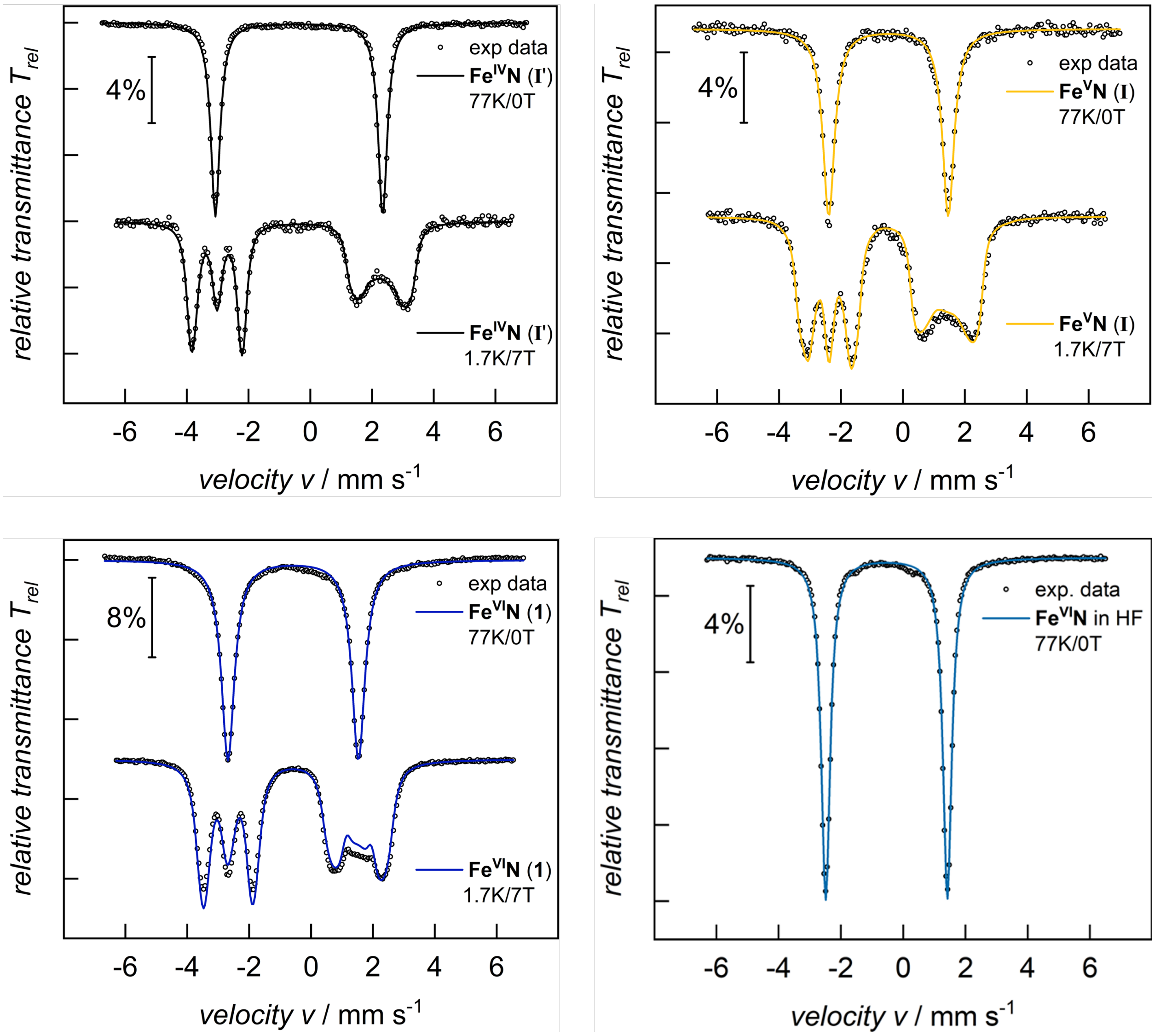

Supplementary Figure 6 | Solid-state zero-/applied-field ^{57}Fe Mössbauer spectra of $[(TIMMN^{Mes})Fe^{IV,V}(N)]^{1+,2+}$ (**I'**/**I**) (black/yellow trace, *top*) and its oxidized congener $[(TIMMN^{Mes})Fe^{VI}(N)(F)]^{2+}$ (**1**) (blue trace, (*bottom, left*)), measured at 77 K/1.7 K, respectively. *Bottom, right*: Solid-state zero-field ^{57}Fe Mössbauer spectrum of **1** (light blue trace), after dilution in HF and evaporation, measured at 77 K.

(**I'** *(top, left)*, solid state, 77 K/0T): $\delta = -0.36$ mm s^{-1}, $|\Delta E_Q|$ = 5.42 mm s^{-1}, Γ_{FWHM} = 0.30 mm s^{-1}.

(**I'** *(top, left)*, solid state, 1.7 K/7T): $\delta = -0.34$ mm s^{-1}, $\Delta E_Q = +5.42$ mm s^{-1}, $\Gamma_{FWHM} = 0.30$ mm s^{-1}, $\eta = 0.20$.

(**I** *(top, right)*, solid state, 77 K/0T): $\delta = -0.48$ mm s^{-1}, $|\Delta E_Q| = 3.85$ mm s^{-1}, $\Gamma_{FWHM} = 0.46$ mm s^{-1}.

(**I** *(top, right)*, solid state, 1.7 K/7T): $\delta = -0.46$ mm s^{-1}, $\Delta E_Q = +3.85$ mm s^{-1}, $\Gamma_{FWHM} = 0.46$ mm s^{-1}, $\eta = 0.15$.

(**1** *(bottom, left)*, solid state, 77 K/0T): $\delta = -0.60$ mm s^{-1}, $|\Delta E_Q| = 4.16$ mm s^{-1}, $\Gamma_{FWHM} = 0.36$ mm s^{-1}.

(**1** *(bottom, left)*, solid state, 1.7 K/7T): $\delta = -0.58$ mm s^{-1}, $\Delta E_Q = +4.16$ mm s^{-1}, $\Gamma_{FWHM} = 0.36$ mm s^{-1}, $\eta = 0.54$.

(**1** *(bottom, right)*, solid state, 77 K/0T): $\delta = -0.60$ mm s^{-1}, $|\Delta E_Q| = 4.16$ mm s^{-1}, $\Gamma_{FWHM} = 0.36$ mm s^{-1}.

Note: The ^{57}Fe Mössbauer isomer shift at 1.7 K is less negative compared to the data collected at 77 K due to the second-order Doppler effect[33].

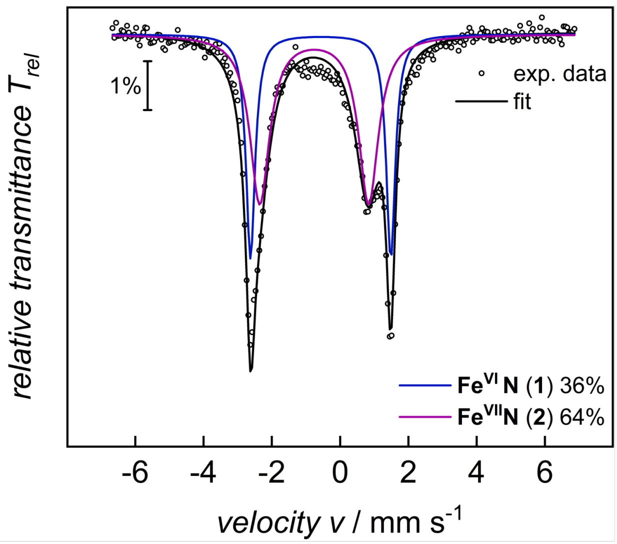

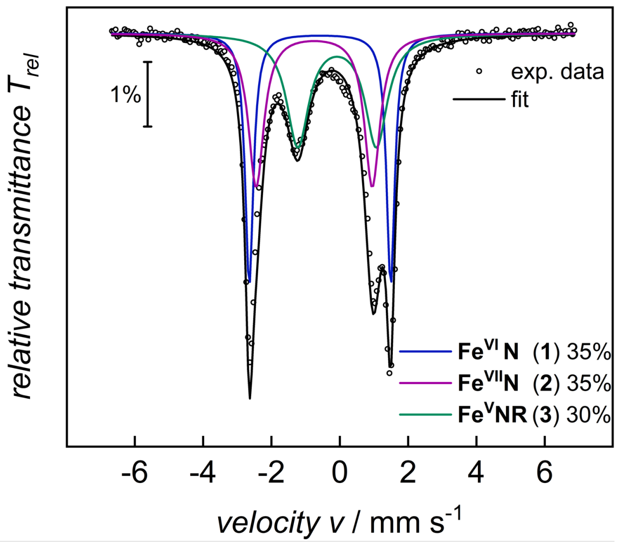

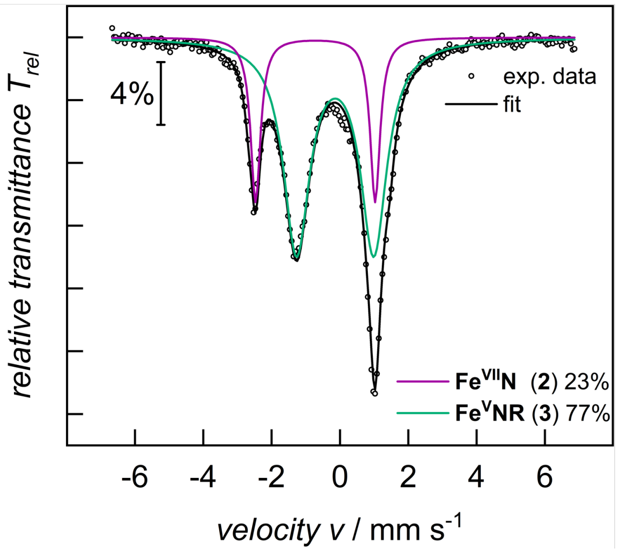

Supplementary Figure 7 | Zero-field ^{57}Fe Mössbauer spectroscopy of the formation (*top*, *left*) and rearrangement (*top, right*) of **2**, trapped in frozen acetonitrile solution (1 mM, 20% ^{57}Fe enriched sample). *Bottom*: Solid-state zero-field ^{57}Fe Mössbauer spectrum after further rearrangement of **2**.

(**1/2** *(top, left)*, frozen solution (CH_3CN), 77 K/0T): (**1**, 36%) $\delta = -0.60$ mm s^{-1}, $|\Delta E_Q| = 4.16$ mm s^{-1}, $\Gamma_{FWHM} = 0.36$ mm s^{-1} and (**2**, 64%) $\delta = -0.72$ mm s^{-1}, $|\Delta E_Q| = 3.30$ mm s^{-1}, $\Gamma_{FWHM} = 0.48$ mm s^{-1}.

(**1-3** *(top, right)*, frozen solution (CH_3CN), 77 K/0T): (**1**, 36%) $\delta = -0.60$ mm s^{-1}, $|\Delta E_Q| = 4.16$ mm s^{-1}, $\Gamma_{FWHM} = 0.36$ mm s^{-1}, (**2**, 35%) $\delta = -0.72$ mm s^{-1}, $|\Delta E_Q| = 3.30$ mm s^{-1}, $\Gamma_{FWHM} = 0.40$ mm s^{-1} and (**3**, 30%) $\delta = -0.16$ mm s^{-1}, $|\Delta E_Q| = 2.35$ mm s^{-1}, $\Gamma_{FWHM} = 0.65$ mm s^{-1}.

(**2/3** *(bottom)*, solid state, 77 K/0T): (**2**, 23%) $\delta = -0.72$ mm s^{-1}, $|\Delta E_Q| = 3.30$ mm s^{-1}, $\Gamma_{FWHM} = 0.40$ mm s^{-1} and (**3**, 77%) $\delta = -0.16$ mm s^{-1}, $|\Delta E_Q| = 2.30$ mm s^{-1}, $\Gamma_{FWHM} = 0.65$ mm s^{-1}.

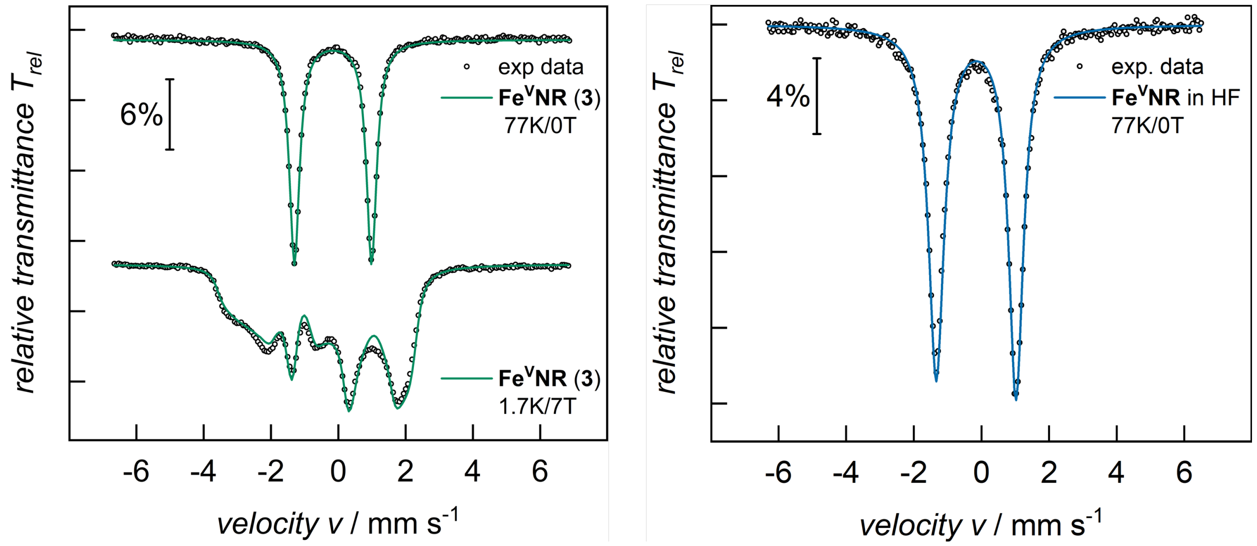

Supplementary Figure 8 | *Left*: Solid-state zero-/applied -field ^{57}Fe Mössbauer spectra of $[(TIMMN^{Mes*})Fe^{V}(=N^{*})(NCMe)]^{3+}$(**3**) (green trace) measured at 77 K/1.7 K, respectively. *Right*: Solid-state zero-field ^{57}Fe Mössbauer spectrum of **3** (light blue trace), after dilution in HF and evaporation, measured at 77 K.

(**3** *(left, top)*, solid state, 77 K/0T): $\delta = -0.16$ mm s^{-1}, $|\Delta E_Q| = 2.30$ mm s^{-1}, $\Gamma_{FWHM} = 0.60$ mm s^{-1}.

(**3** *(left, bottom)*, solid state, 1.7 K/7T): $\delta = -0.14$ mm s^{-1}, $\Delta E_Q = +2.30$ mm s^{-1}, $\Gamma_{FWHM} = 0.60$ mm s^{-1}, $\eta = 0.35$.

(**3** *(right)*, solid state, 77 K/0T): $\delta = -0.16$ mm s^{-1}, $|\Delta E_Q| = 2.30$ mm s^{-1}, $\Gamma_{FWHM} = 0.60$ mm s^{-1}.

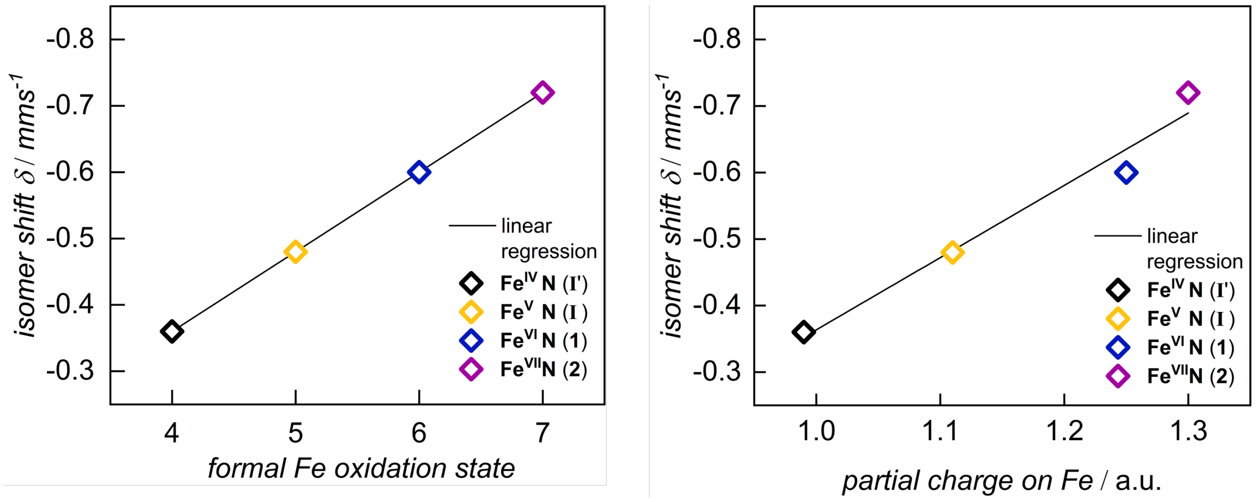

Supplementary Figure 9 | *Left*: Linear correlation diagram of the experimentally determined ^{57}Fe Mössbauer isomer shift *vs.* Fe oxidation state of $[(TIMMN^{Mes})Fe^{IV,V}(N)]^{1+,2+}$ (**I'/I**) (black/yellow symbol) and $[(TIMMN^{Mes})Fe^{VI,VII}(N)(F)]^{2+,3+}$ (**1/2**) (blue/purple symbol). *Right*: Correlation diagram of the exp. ^{57}Fe Mössbauer isomer shift of $[(TIMMN^{Mes})\text{-}Fe^{IV,V}(N)]^{1+,2+}$ (**I'/I**) (black / yellow symbol) and $[(TIMMN^{Mes})Fe^{VI,VII}(N)(F)]^{2+,3+}$ (**1/2**) (blue/purple symbol) *vs.* the calculated Bader atomic partial charge (QTAIM, PBE functional) on Fe.

Isomer shift of $[(TIMMN^{Mes})Fe^{VI,VII}(N)(F)]^{2+,3+}$ (1/2) *vs.* $[FeO_4]^{2-}$

Despite the same +VI oxidation state, the isomer shift, δ, of **1** (at 77 K) is –0.60 mm s^{-1}, while the values for tetraoxoferrate(VI) complexes range from –0.79 to –0.85 mm s^{-1}, depending on the cation[34,35]. The more negative isomer shift for the oxoferrates can be rationalized by a combined effect of Fe–ligand bond length and the Fe(4*s*) contribution to the bonding molecular orbitals.

Bond Length

While **1** only has one exceptionally short bond of 1.518(3) Å with the nitrido ligand, $K_2[FeO_4]$ has four short Fe–O bonds at 1.645(2) Å and 1.653(1) Å[36,37]. As a consequence, the shorter bonds compress the 3*s* and 4*s* iron orbitals, which, *i.e.*, shifts the maximum electronic density probability towards the nucleus. This increases the electronic density at the ^{57}Fe nucleus, thus leading to more negative isomer shifts ([33], p. 162-164).

Contribution of Fe(4*s*) to the Bonding Orbitals

Further, the increased covalency of the $[FeO_4]^{2-}$ dianion relative to **1** should lead to a more pronounced mixing of 4*s* with the ligand orbitals. Additionally, the tetrahedral geometry of the $[FeO_4]^{2-}$ anion (O–Fe–O angles of 108.4-109.9°)[36,37] imparts an additional, particular effect. Usually, the 4*s* contribution on the valence molecular orbitals does not correlate with the electronic density at the ^{57}Fe nucleus[38]. However, in T_d point group, the totally symmetric bonding molecular orbital contains only Fe(*s*) contributions, as the *s* orbitals transform to an a_1 irreducible representation. Due to the orthogonality of *p* (t_2) and *d* (*e*, t_2) functions, the totally symmetric molecular orbital has no shielding imparted by *p* and *d* electrons, preventing *s* electrons from relaxing and allowing them to induce a larger electronic density at the nucleus.

Consequently, these effects contribute to the exceptionally negative isomer shift of $[FeO_4]^{2-}$ relative to **1**, where the lower symmetry (C_s) allows *s-p* and *s-d* mixing. Even the higher oxidation state of **2**, and further contraction of 3*s* and 4*s* orbitals, causes its isomer shift (δ = –0.72 mm s^{-1}) to be less negative than that of $[FeO_4]^{2-}$, thus evidencing the importance of the 4*s* participation in the bonding orbitals. The influence of "4*s* electron inflow" was discussed previously[39].

Finally, the same symmetry argument has been suggested to explain the more negative isomer shift of tetrahedral tetraalkylferrate(IV) complexes – despite their longer Fe–ligand bonds – relative to trigonal bipyramidal alkynylferrate(IV) species[40].

Voltammetry

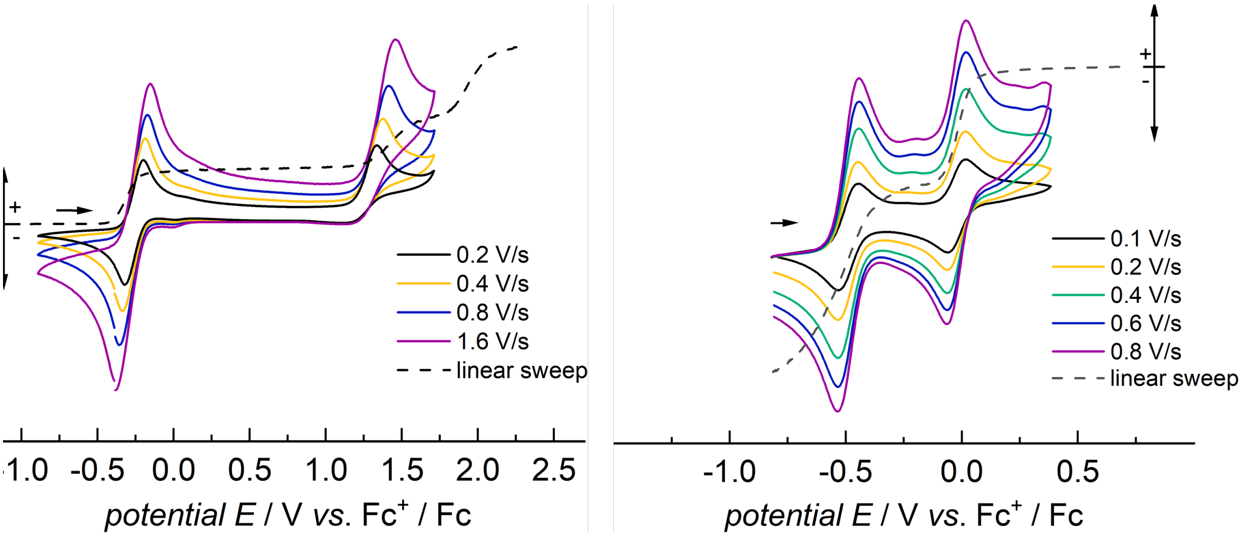

Supplementary Figure 10 | *Left*: Cyclic/ linear sweep voltammograms of $[(TIMMN^{Mes})Fe^{IV}(N)]^{1+}$ (**I'**) in dichloromethane solution, measured at room temperature with 0.1 M N(n-Bu)$_4$PF$_6$ electrolyte and varying scan rates (colored lines, 0.2–1.6 V/s), referenced *vs*. $Fe(Cp)_2^+/Fe(Cp)_2$. *Right*: Cyclic/ linear sweep voltammograms of $[(TIMMN^{Mes*})Fe^{V}(=N^*)(NCMe)]^{3+}$(**3**) in acetonitrile solution, measured at room temperature with 0.1 M N(*n*-Bu)$_4$PF$_6$ electrolyte and varying scan rates (0.1–0.8 V/s), referenced *vs*. $Fe(Cp)_2^+/Fe(Cp)_2$.

(**I'** *(left)*, 0.02–1.60 V/s, CH_2Cl_2): $E_{1/2}$ = −0.35 V (**I'/I**), $E_{1/2}$ = +1.25 V (**I/1**), $E_{1/2}$ = +1.75 V (**1/2**).

(**3** *(right)*, 0.02–1.60 V/s, CH_3CN): $E_{1/2}$ = −0.05 V (**3/3red$_1$**), $E_{1/2}$ = −0.50 V (**3red$_1$/3red$_2$**).

Note: The voltammograms of **3** further corroborate the clean and full conversion of **2** to **3** as they show no additional redox events of different intensity of the remaining starting material or decomposition products.

Fe K-edge X-Ray Absorption Spectroscopy

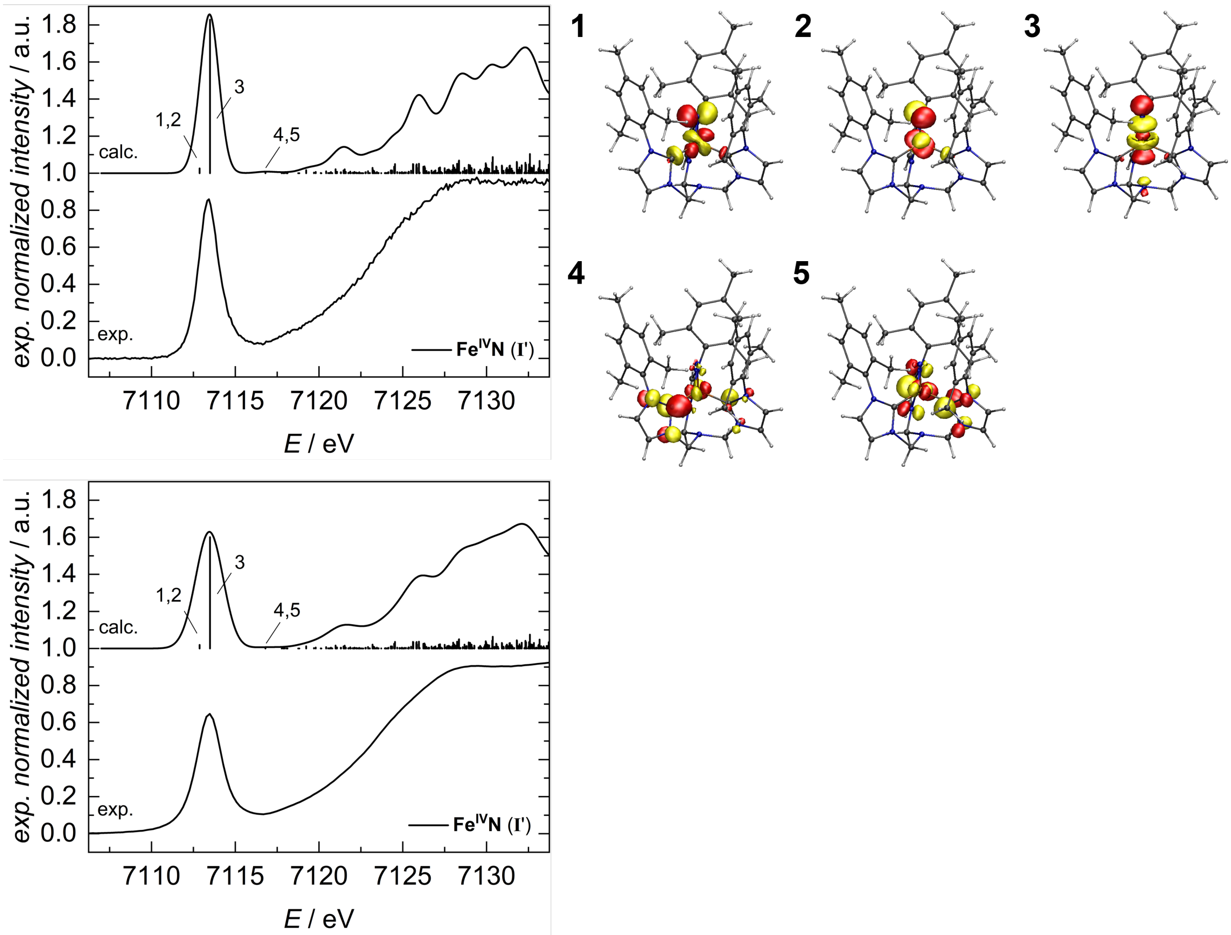

Supplementary Figure 11 | Experimental Fe K-edge X-ray absorption spectra of [(TIMMN^{Mes})Fe^{IV}(N)]$^{1+}$ (**I'**) measured in K$\beta_{1,3}$-HERFD (*top, left*) and transmission mode (*bottom, left*) combined with the corresponding TDDFT calculated spectra of **I'** with an offset of 1.0 a.u. (black traces, *left top*: line width = 1.3 eV, energy shift = +17.0 eV, *left bottom*: linewidth = 1.9 eV, energy shift = +16.5 eV. Line shape: Gaussian). Main excited states calculated by TDDFT/PBE0/def2-TZVPP/ZORA level on H-only optimized structure of **I'** are numbered and the respective acceptor natural transition orbitals are shown (*right*). Transitions 1*s* to 3*d*: states 1–3; MLCT: states 4, 5.

Supplementary Table 1 | Assignment of Fe K-edge XAS spectrum calculated by TDDFT at PBE0/def2-TZVPP/ZORA level on H-only optimized structure **I'**.

**Stick numbers / States**	**Energy without shift correction (eV)**	**Energy with shift correction in HERFD / transmission mode(+17.0 / 16.5 eV)**	**Assignment of the Natural Transition Orbitals (NTOs)**
**1**	**7095.87**	**7112.87 / 7112.37**	*dxz*
**2**	**7095.87**	**7112.87 / 7112.37**	*dyz*
**3**	**7096.49**	**7113.49 / 7112.99**	dz^2 *+ 4pz (7.5%)*
**4**	**7099.80**	**7116.80 / 7116.30**	*dxy + NHC* π*
**5**	**7099.80**	**7116.80 / 7116.30**	dx^2-y^2*+ NHC* π*

The ground state electronic configuration in C_3 point group of $[(TIMMN^{Mes})Fe^{IV}(N)]^{1+}$ (**I'**) is $e(x^2\text{-}y^2, xy)^4$, $e(xz, yz)^0$, $a(z^2)^0$. The pre-edge peak is dominated by the 1*s* to 3$d(z^2)$ transition due to the significant covalency with the nitrido ligand and the large 4*p* participation (7.2%). The transitions to *d*(xz) and *d*(yz) are weak and appear at lower energy with respect to $d(z^2)$. The weak feature at the rising edge is composed of a transition to a doubly degenerate NHC π^* set combined with *d*(xy) and $d(x^2\text{-}y^2)$.

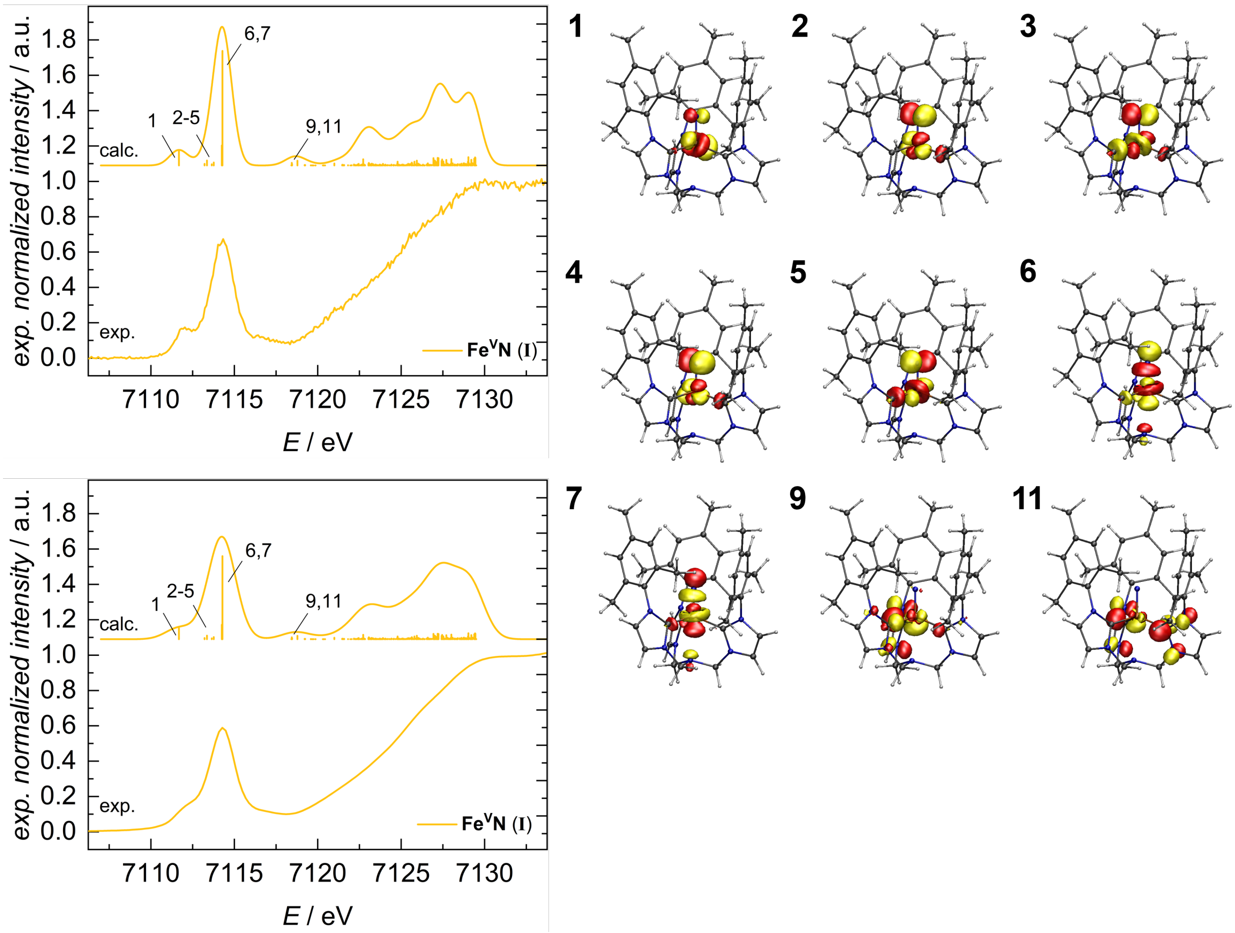

Supplementary Figure 12 | Experimental Fe K-edge X-ray absorption spectra of $[(TIMMN^{Mes})Fe^{V}(N)]^{2+}$ (**I**) measured in $K\beta_{1,3}$-HERFD (*top, left*) and transmission mode (*bottom*, *left*) combined with the corresponding TDDFT calculated spectra of **I** with an offset of 1.1 a.u. (yellow traces, *left top*: line width = 1.3 eV, energy shift = +17.0 eV, *left bottom*: line width = 1.9 eV, energy shift = +16.5 eV. Line shape: Gaussian). Main excited states calculated by TDDFT/PBE0/def2-TZVPP/ZORA level on H-only optimized structure of **I** are numbered, and the respective acceptor natural transition orbitals are shown (*right*). Transitions 1*s* to 3*d*: states 1–7; MLCT: states 9, 11.

Supplementary Table 2 | Assignment of Fe K-edge XAS spectrum calculated by TDDFT at PBE0/def2-TZVPP/ZORA level on H-only optimized structure **I**.

Stick numbers / States	Energy without shift correction (eV)	Energy with shift correction in HERFD / transmission mode(+17.0 / 16.5 eV)	Assignment of the Natural Transition Orbitals (NTOs)
**1**	**7094.68**	**7111.68/ 7111.18**	$dx^2\text{-}y^2$ (*β*)
**2**	**7096.22**	**7113.22 / 7112.72**	*dxz (β)*
**3**	**7096.36**	**7113.36 / 7112.86**	*dyz (α)*
**4**	**7096.65**	**7113.65 / 7113.15**	*dxz (α)*
**5**	**7096.77**	**7113.77 / 7113.27**	*dyz (β)*
**6**	**7097.25**	**7114.25 / 7113.75**	dz^2 (*α*)
**7**	**7097.29**	**7114.29 / 7113.79**	dz^2 (*β*)
**9**	**7101.44**	**7118.44 / 7117.94**	*NHC π* − dxy (α)*
**11**	**7101.79**	**7118.79 / 7118.29**	*NHC π* − dxy (β)*

The ground state electronic configuration in C_3 point group of $[(\text{TIMMN}^{\text{Mes}})\text{Fe}^{\text{V}}(\text{N})]^{2+}$ (**I**) is $e(x^2\text{-}y^2, xy)^3$, $e(xz, yz)^0$, $a(z^2)^0$. The pre-edge peak is again dominated by the 1*s* to 3$d(z^2)$ transition, with a small peak at the low-energy side of the main peak, attributed to a transition from the 1*s* to the singly occupied $d(x^2\text{-}y^2)$ orbital. The weak transitions to *d*(xz) and *d*(yz) appear between these two peaks. The feature at the rising edge is composed by transitions to the NHC *π** set with *d*(xy).

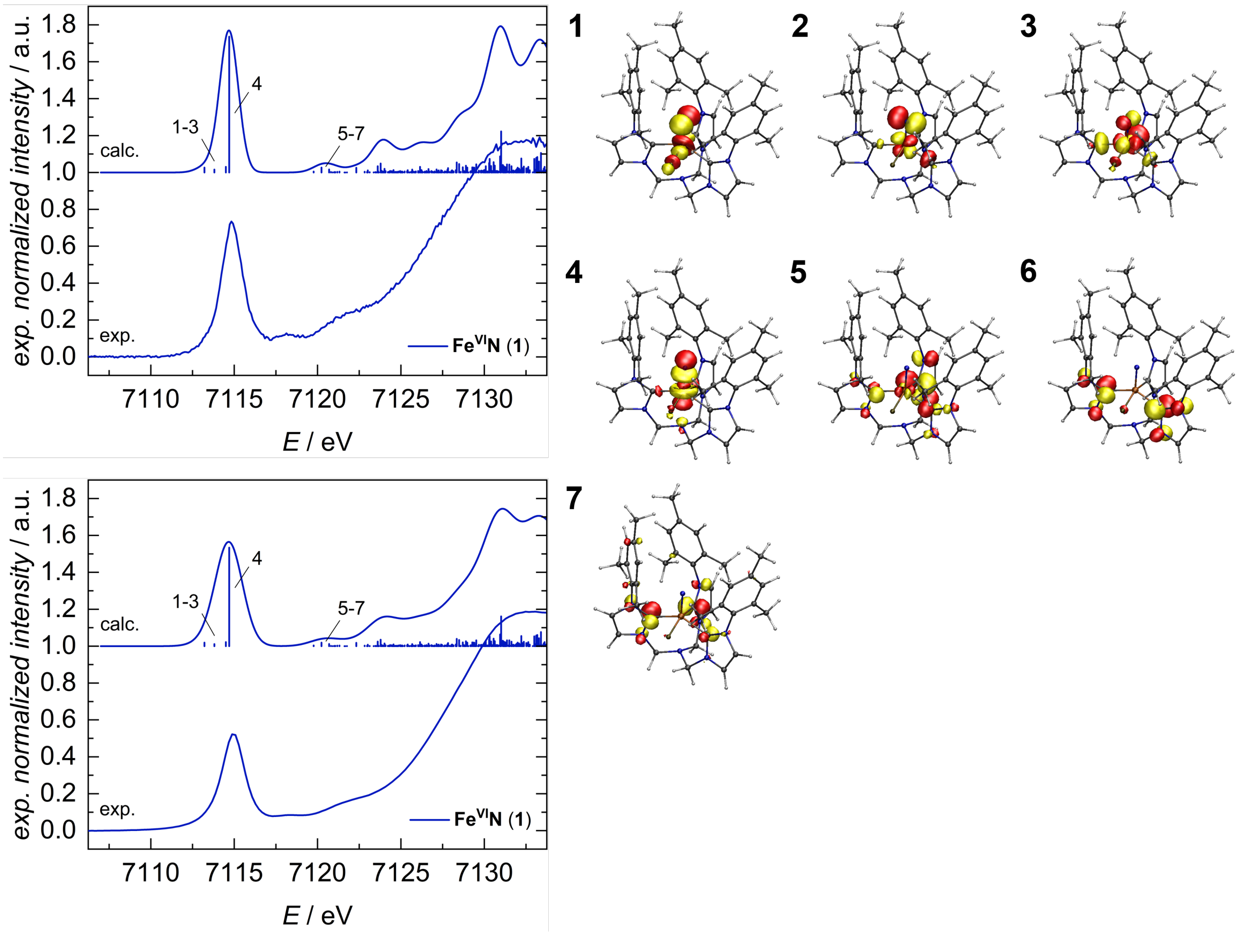

Supplementary Figure 13 | Experimental Fe K-edge X-ray absorption spectrum of $[(TIMMN^{Mes})Fe^{VI}(N)(F)]^{2+}$ (**1**) measured in K$\beta_{1,3}$-HERFD (*top*, *left*) and transmission mode (*bottom*, *left*) combined with the corresponding TDDFT calculated spectra of **1** with an offset of 1.0 a.u. (blue traces, *left top*: line width = 1.3 eV, energy shift = +17.0 eV, *left bottom*: line width = 1.9 eV, energy shift = +16.5 eV. Line shape: Gaussian). Main excited states calculated by TDDFT/PBE0/def2-TZVPP/ZORA level on H-only optimized structure of **1** are numbered and the respective acceptor natural transition orbitals are shown (*right*). Transitions 1*s* to 3*d*: states 1–4; MLCT: states 5–7.

Supplementary Table 3 | Assignment of Fe K-edge XAS spectrum calculated by TDDFT at PBE0/def2-TZVPP/ZORA level on H-only optimized structure **1**.

Stick numbers / States	Energy without shift correction (eV)	Energy with shift correction in HERFD / transmission mode(+17.0 / 16.5 eV)	Assignment of the Natural Transition Orbitals (NTOs)
**1**	**7096.22**	**7113.22/ 7112.72**	*dxz*
**2**	**7096.81**	**7113.81 / 7113.31**	*dyz*
**3**	**7097.50**	**7114.50 / 7114.00**	dx^2-y^2
**4**	**7097.70**	**7114.70 / 7114.20**	dz^2
**5**	**7102.77**	**7119.77 / 7119.27**	*NHC* π^* − *dxy*
**6**	**7103.24**	**7120.24 / 7119.74**	*NHC* π^*
**7**	**7103.70**	**7120.70 / 7120.20**	*NHC* π^*

The ground state electronic configuration in C_s point group of $[(TIMMN^{Mes})Fe^{VI}(N)(F)]^{2+}$ (**1**) is $a''(xy)^2$, $a'(xz)^0$, $a''(yz)^0$, $a'(z^2)^0$, $a'(x^2\text{-}y^2)^0$ with the mirror plane along the F-Fe-N nuclei, thus leaving the two d electrons paired in the $3d(xy)$ orbital. The asymmetry at the low-energy side of the pre-edge peak is attributed to (i) $1s$ to $3d(xz)$ transition along the Fe–F bond on the x-axis, with some F contribution, (ii) $1s$ to $3d(yz)$, (iii) $1s$ to $3d(x^2\text{-}y^2)$. The pre-edge peak is again dominated by the most intense feature identified as the $1s$ to $3d(z^2)$ transition.

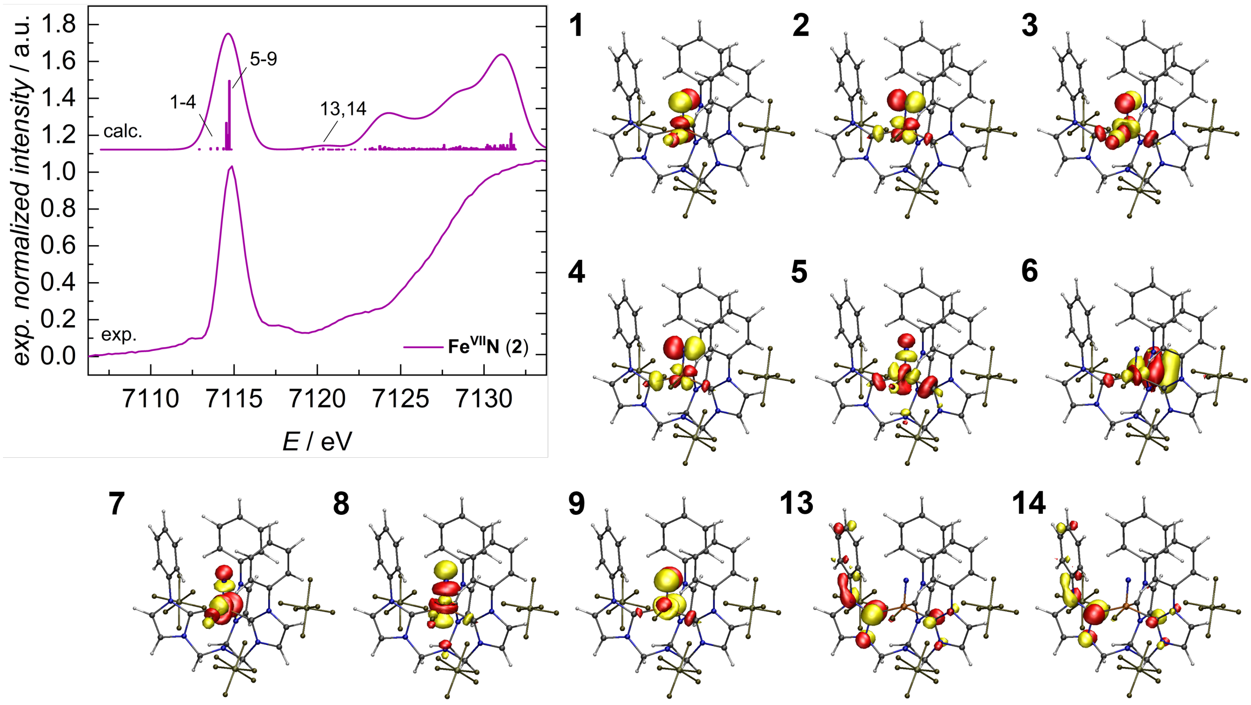

Supplementary Figure 14 | Experimental Fe K-edge X-ray absorption spectrum of $[(TIMMN^{Mes})Fe^{VII}(N)(F)]^{3+}$ (**2**) measured in transmission mode and the corresponding TDDFT calculated spectrum of **2** with an offset of 1.0 a.u. (purple traces, *left*: line width = 1.9 eV, energy shift = +16.5 eV. Line shape: Gaussian). The data have been deconvoluted from a mixture of **2** and **3** (see Supplementary Figure15) with their relative ratios obtained by ^{57}Fe Mössbauer spectroscopy of the same batch (see Supplementary Figure 7, *bottom*). Main excited states calculated by TDDFT/PBE0/def2-TZVPP/ZORA level on fully optimized structure of $[(TIMMN^{Mes})Fe^{VII}(N)(F)](PF_6)_3$ (**2**) at ZORA-PBE0-D3/def2-SVP/SMD(MeCN) level are numbered, and the respective acceptor natural transition orbitals are shown (*right*). Transitions $1s$ to $3d$: states 1–9; MLCT: states 13, 14.

Supplementary Table 4 | Assignment of Fe K-edge XAS spectrum calculated by TDDFT at PBE0/def2-TZVPP/ZORA level on fully optimized structure of $[(TIMMN^{Mes})Fe^{VII}(N)(F)](PF_6)_3$ (**2**).

Stick numbers / States	Energy without shift correction (eV)	Energy with shift correction in transmission mode(+16.5 eV)	Assignment of the Natural Transition Orbitals (NTOs)
**1**	**7095.92**	**7112.42**	*dxz (β)*
**2**	**7096.60**	**7113.10**	*dyz (β)*
**3**	**7097.00**	**7113.50**	*dx^2-y^2 (α)*
**4**	**7097.37**	**7113.87**	*dyz (α)*
**5**	**7097.54**	**7114.04**	*dz^2 + 4pz (10%) (β)*
**6**	**7097.56**	**7114.06**	*dxy + NHC π* (β)*
**7**	**7097.63**	**7114.13**	*dx^2-y^2 (β)*
**8**	**7097.72**	**7114.22**	*dz^2 (α)*

**9**	**7097.84**	**7114.34**	*dxz (α)*
**13**	**7103.34**	**7119.84**	*NHC π* (β)*
**14**	**7103.36**	**7119.86**	*NHC π* (α)*

The ground state electronic configuration in *C*s point group of $[(TIMMN^{Mes})Fe^{VII}(N)(F)]^{2+}$ (**2**) is $a''(xy)^1$, $a'(xz)^0$, $a''(yz)^0$, $a'(z^2)^0$, $a'(x^2-y^2)^0$ with the unpaired electron located in the 3*d*(xy) orbital. The asymmetry at the low-energy side of the pre-edge peak is again attributed to (i) 1*s* to 3*d*(xz) transition, along the Fe–F bond on the *x*-axis, with some F contribution, (ii) 1*s* to 3*d*(yz), (iii) 1*s* to 3*d*(x^2-y^2). The pre-edge peak is dominated by the most intense feature identified as the 1*s* to 3*d*(z^2) transition. The first rising-edge feature is a MLCT transition to the carbene *π** orbitals.

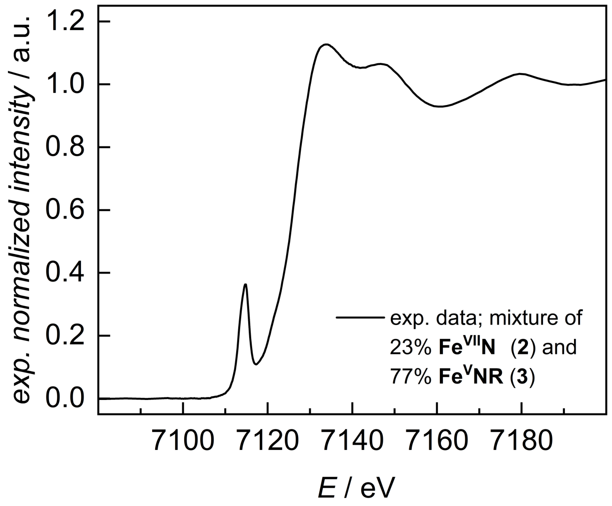

Supplementary Figure 15 | Experimental Fe K-edge X-ray absorption spectrum of a mixture of $[(TIMMN^{Mes})Fe^{VII}(N)(F)]^{3+}$ (**2**) and $[(TIMMN^{Mes*})Fe^{V}(=N^{*})(NCMe)]^{3+}$(**3**) measured in transmission mode.

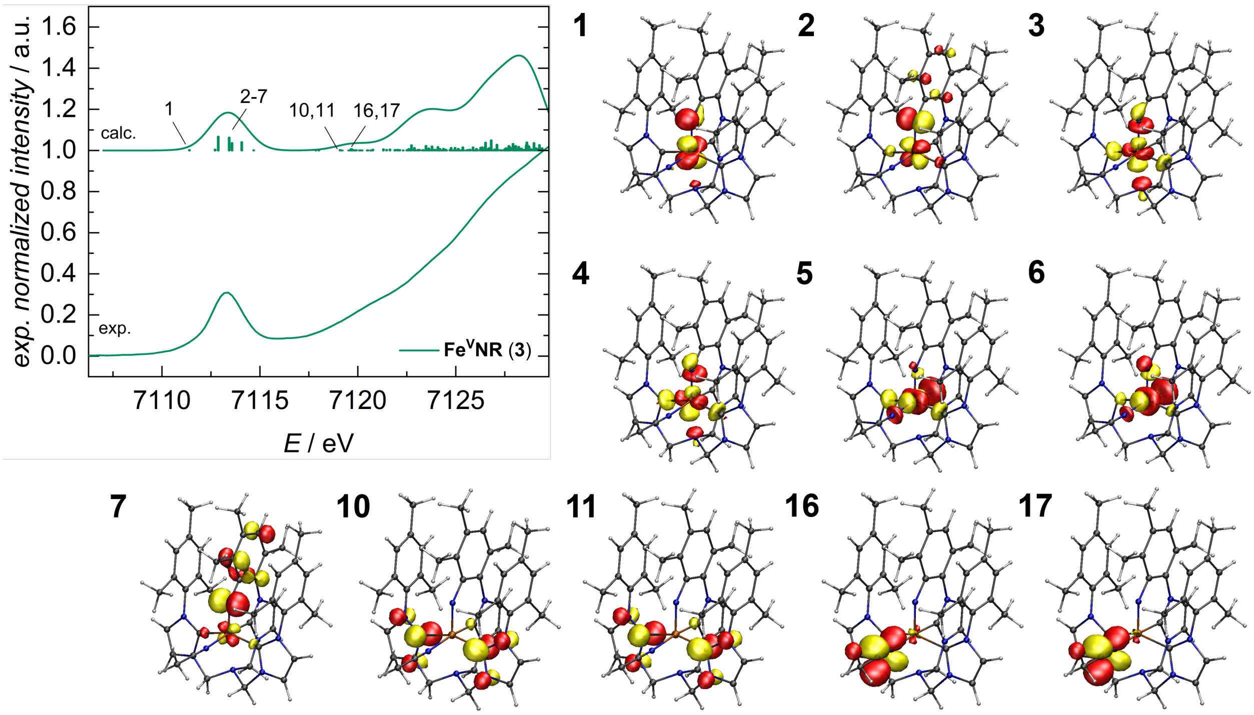

Supplementary Figure 16 | Experimental Fe K-edge X-ray absorption spectrum of $[(TIMMN^{Mes*})Fe^{V}(=N^{*})(NCMe)]^{3+}$(**3**) measured in transmission mode and the corresponding TDDFT calculated spectrum of **3** with an offset of 1.0 a.u. (green traces, *left*: linewidth = 1.9 eV, energy shift = +16.5 eV. Line shape: Gaussian). Main excited states calculated by TDDFT/PBE0/def2-TZVPP/ZORA level on H only optimized structure of **3** are numbered, and the respective acceptor natural transition orbitals are shown (*right*). Transitions 1*s* to 3*d*: states 1–7; MLCT: states 10, 11, 16, and 17.

Supplementary Table 5 | Assignment of Fe K-edge XAS spectrum calculated by TDDFT at PBE0/def2-TZVPP/ZORA level on H-only optimized structure **3**.

Stick numbers / States	Energy without shift correction (eV)	Energy with shift correction in transmission mode(+16.5 eV)	Assignment of the Natural Transition Orbitals (NTOs)
**1**	**7094.40**	**7110.90**	*dxz (β)*
**2**	**7095.71**	**7112.21**	*dyz (β)*
**3**	**7095.87**	**7112.37**	*dz^2 (α)*
**4**	**7096.43**	**7112.93**	*dz^2 (β)*
**5**	**7096.57**	**7113.07**	*dx^2-y^2 (α)*
**6**	**7097.07**	**7113.57**	*dx^2-y^2 (β)*
**7**	**7097.68**	**7114.18**	*dyz (α)*
**8**	**7102.09**	**7118.59**	*NHC π* (β)*
**9**	**7102.11**	**7118.61**	*NHC π* (α)*
**10**	**7102.65**	**7119.15**	*CH_3CN π* (α)*
**11**	**7102.68**	**7119.18**	*CH_3CN π* (β)*

The ground state electronic configuration in C_S point group of *low-spin* $[(TIMMN^{Mes*})Fe^V(=N^*)(NCMe)]^{3+}$ (**3**) is $a''(xy)^2$, $a'(xz)^1$, $a'(z^2)^0$, $a''(yz)^0$, $a'(x^2\text{-}y^2)^0$ considering the mirror plane along the N_{MeCN}–Fe–N_{imide} nuclei. The 3*d*(xz) orbital (SOMO) is along the axis with the weakest field ligand (MeCN), while the 3*d*(yz) is destabilized even above the 3*d*(z^2) due to the strongest field induced by the NHC relative to the amine-imide pair. The rising-edge feature is dominated by MLCT transitions to the acetonitrile *π** orbitals and less intense MLCT to NHC *π** transitions appearing at lower energies.

Note: The calculated XAS spectrum of the final, completely rearranged Fe(V)imido, acetonitrile adduct complex, $[(TIMMN^{Mes*})Fe^V(=N^*)(NCMe)]^{3+}$ (**3**) agrees better with the experimental data than the calculations for the Fe(V) amido, fluoride adduct complex, $[(TIMMN^{Mes*})Fe^V(NH^*)(F)]^{3+}$ (intermediate **e**) before HF elimination.

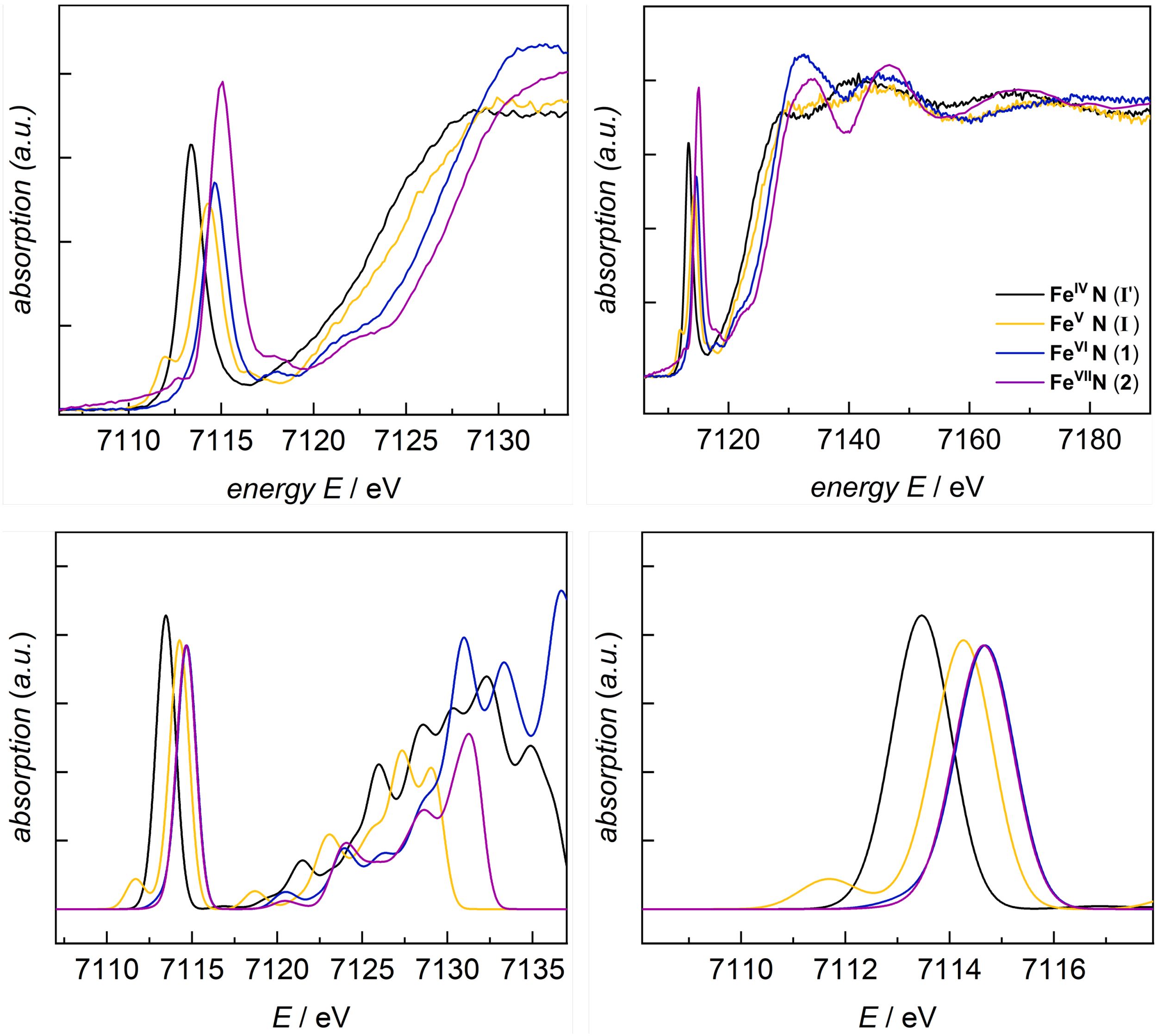

Supplementary Figure 17 | *Top*; overlay of the experimental Fe K-edge X-ray absorption spectra of **I'** (black), **I** (yellow) and **1**(blue) measured in K$\beta_{1,3}$-HERFD mode and of deconvoluted **2** in transmission mode. *Bottom*; overlay of the corresponding TDDFT calculations for [(TIMMN^{Mes})Fe^{IV,V}(N)]$^{1+,2+}$ (**I'**/**I**) (black/yellow trace) and [(TIMMN^{Mes})Fe^{VI,VII}(N)(F)]$^{2+,3+}$ (**1**/**2**) (blue/purple trace).

Supplementary Table 6 | Comparison of measured and calculated (in parentheses) Fe K-edge X-ray absorption spectra parameters of **I'**,**I** ,**1** and **2**. Calculated energies are shifted by 17.0 eV.

	**[Fe^{IV}(N)]$^{1+}$ I'**	**[Fe^{V}(N)]$^{2+}$ I**	**[Fe^{VI}(N)(F)]$^{2+}$ 1**	**[Fe^{VII}(N)(F)]$^{3+}$ 2**
**Pre-edge peaks [eV]**	**7113.4 (7113.5)**	**7114.3 (7114.3)**	**7114.9 (7114.8)**	**7115.0 (7114.8)**
**1st inflection point at rising edge [eV]**	**7123.9**	**7125.4**	**7126.9**	**7128.1**

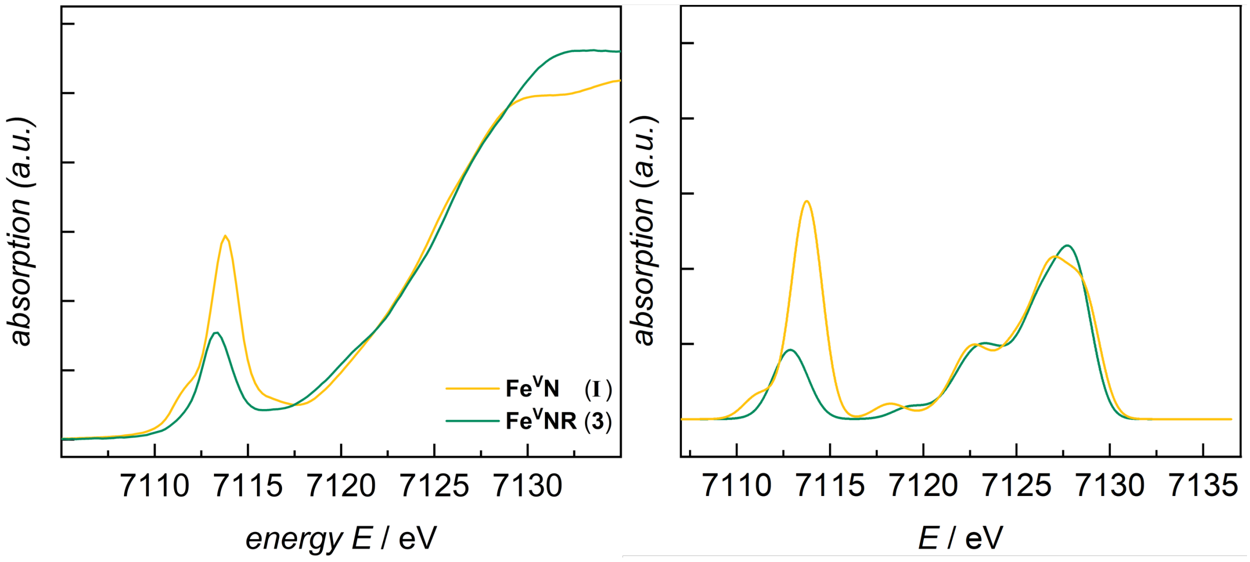

Supplementary Figure 18 | Overlay of the experimental Fe K-edge X-ray absorption spectra (*left*) and the corresponding TDDFT calculations (*right*) of $[(TIMMN^{Mes})Fe^{V}(N)]^{2+}$ (**I**) (yellow trace) and $[(TIMMN^{Mes*})Fe^{V}(=N^{*})(NCMe)]^{3+}$(**3**) (green trace).

Supplementary Table 7 | Comparison of measured and calculated (in parentheses) Fe K-edge X-ray absorption spectra parameters of **I** and **3**. Calculated energies are shifted by 16.5 eV.

	$[Fe^{V}(N)]^{2+}$ **I**	$[Fe^{V}(NR)(NCMe)]^{3+}$ **3**
**Pre-edge peaks [eV]**	**7113.8 (7113.8)**	**7113.3 (7113.0)**
**1st inflection point at rising edge [eV]**	**7125.0**	**7125.5**

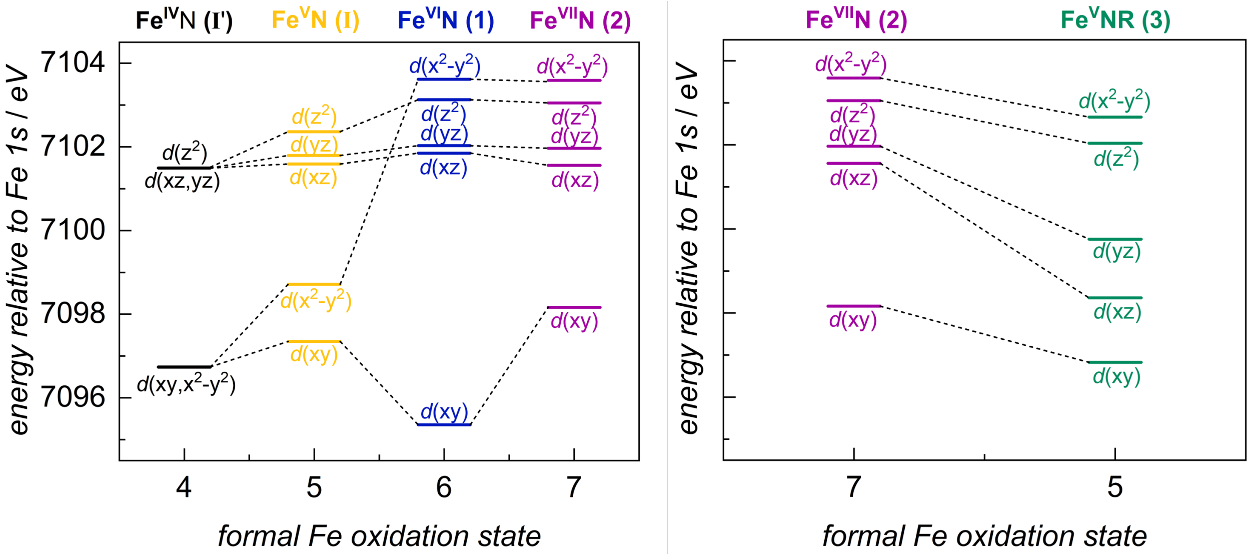

Supplementary Figure 19 | Ligand field splitting obtained by TD-DFT (PBE0/ZORA-def2-TZVPP), illustrating the energy difference between the 1*s*-orbital and the quasi-restricted orbitals (QROs) associated with the 3*d* shell. The orbitals' main *d* character is indicated.

The energy of the pre-edge peak is dominated by the MO with a predominant $d(z^2)$ character and with an antibonding combination with the $N_{nitrido}$ $2p(z)$. The energy of the $d(z^2)$ increases going from Fe(IV) to Fe(VI), which is consistent with the experimental trend. The experimental data for the Fe(VII) complex shows the peak ~0.1 eV at higher energy relative to Fe(VI). This is not captured by the best model of **2**, which resulted in the $d(z^2)$ being slightly lower (~0.1 eV) than the Fe(VI). This finding might be attributed to minor errors associated with the deconvolution of the XAS spectrum of **2** from the reaction mixture and to the DFT geometry optimization for the $[(TIMMN^{Mes})Fe^{VII}(N)(F)](PF_6)_3$ model of **2**, in which the Fe≡N distance is slightly longer (1.519 Å) than the crystallographically determined Fe≡N distance of **1** (1.518 Å). The energy of the $d(x^2\text{-}y^2)$ increases steeply and surpasses the $d(z^2)$ when the fluorido ligand coordinates in the Fe(VI). This indicates that, although the Fe-nitrido bond is short, the Fe–N_{amine} is long, making the field strength on the *xy*-plane larger than along the *z*-axis due to the fluorido ligand and the three carbenes.

Note: The cyclic rearrangement to the Fe(V) imido complex **3** leads to a stabilization of all orbitals, especially in *d*(xz) and *d*(yz), with respect to the Fe(VII) nitride **2**.

Crystallographic Details

Crystallographic data for the structures reported in this Article have been deposited at the Cambridge Crystallographic Data Centre, under deposition numbers **CCDC-2258913** for $[(TIMMN^{Mes})Fe^{VI}(N)(F)]$-$(PF_6)_2 \cdot CH_2Cl_2$ (**1** • CH_2Cl_2), **CCDC-2297600** for $[(TIMMN^{Mes*})Fe^{V}(NH^*)(F)](PF_6)_3$ (**2e**), and **CCDC-2258914** for $[(TIMMN^{Mes*})$-$Fe^{V}(=N^*)(NCMe)](PF_6)_2MoF_6 \cdot 5\ C_2H_3N$ (**3** • 5 C_2H_3N). Copies of the data can be obtained free of charge via https://www.ccdc.cam.ac.uk/structures/.

Suitable single crystals of the investigated compounds were embedded in protective perfluoropolyalkyether oil on a microscope slide and a single specimen was selected and subsequently transferred to the cold nitrogen gas stream of the diffractometer. Intensity data were collected using MoK_α radiation (λ = 0.71073 Å) on a Bruker Kappa PHOTON 2 *IμS* Duo diffractometer equipped with QUAZAR focusing Montel optics. Data were corrected for Lorentz and polarization effects, semiempirical absorption corrections were performed on the basis of multiple scans using *SADABS*[40]. The structures were solved by direct methods (*SHELX XT* 2014/5)[41] and refined by full-matrix least-squares procedures on F^2 using *SHELXL* 2018/3[42]. All non-hydrogen atoms were refined with anisotropic displacement parameters. All hydrogen atoms were placed in positions of optimized geometry, their isotropic displacement parameters were tied to those of the corresponding carrier atoms by a factor of either 1.2 or 1.5. Olex2 was used to prepare material for publication[43]. Crystallographic data, data collection, and structure refinement details are given in Supplementary Supplementary Table **8**.
In the crystal structure of $[(TIMMN^{Mes})Fe^{VI}(N)(F)](PF_6)_2 \cdot CH_2Cl_2$ one of the ligand's arms was disordered. Two alternative orientations were refined and resulted in site occupancies of 81.7(8) and 18.3(8) for the atoms C22 – C30 and C22A – C30A, respectively. Same anisotropic displacement parameter restraints (EADP) were applied to the corresponding pairs of disordered atoms. Both of the PF_6^- anions were disordered. Two alternative orientations each were refined and resulted in site occupancies of 81.0(10) and 19.0(10) % for the atoms F11 – F16 and F11A – F16A, and of 53.8(6) and 46.2(6) % for the atoms F21 – F26 and F21A – F26A, respectively. Similarity restraints were applied in the refinement of the anisotropic displacement parameters of the disordered fluorine atoms. The compound crystallized with one molecule of CH_2Cl_2 per formula unit. This solvent molecule was also disordered. Two alternative orientations were refined and resulted in site occupancies of 91.0(2) and 9.0(2) for the atoms C100 – Cl12 and C110 – Cl14, respectively. Here, again same anisotropic displacement parameter constraints (EADP) were applied to the corresponding pairs of disordered atoms.

In the crystal structure of $[(TIMMN^{Mes*})Fe^{V}(=N^*)(NCMe)](PF_6)_2MoF_6 \cdot 5\ C_2H_3N$ the complex molecule was situated on a crystallographic mirror plane (Wyckoff position 4*c*) and possesses crystallographically imposed mirror symmetry. The anions turned out to consist of a mixture of PF_6^- and MoF_6^-. Both PF_6^- anions shared their site with a MoF_6^- anion. The site occupancies were refined and resulted in the following values, 67.5(2) % for P1 – F16 and 32.5(2) % for Mo1A – F16A, and 62.9(3) % for P2 – F25 and 37.1(3) % for Mo2A – F25A with the latter anion being located again on a crystallographic mirror plane (Wyckoff position 4*c*). Same anisotropic displacement parameter constraints (EADP) were applied to the atoms P2 and Mo2A. Similarity restraints were applied to the anisotropic displacement parameters of the fluorine atoms of this disordered anion. The compound crystallized with five molecules of acetonitrile per formula unit (four independent molecules, three of which were again located on a crystallographic mirror plane).

Crystals of the intermediate compound $[(TIMMNMes^*)Fe^{V}(\ NH^*)(F)](PF6)_3$ (**2e**) could be isolated only once. A suitable single crystal was mounted on a Bruker D8 Venture Photon 2 diffractometer equipped with an Incoatec *IμS* 2.0 microsource and Montel optics using MoK_α radiation (λ = 0.71073 Å). For technical reasons intensity data could only be collected at room temperature and suffered from loss of incorporated solvent molecules (SO_2 and

HF were used for crystallization). Data were corrected for Lorentz and polarization effects, semiempirical absorption corrections were performed on the basis of multiple scans using *SADABS*[40]. The structures were solved by direct methods (*SHELX XT* 2014/5)[41] and refined by full-matrix least-squares procedures on F^2 using *SHELXL* 2018/3[42]. All non-hydrogen atoms were refined with anisotropic displacement parameters. All hydrogen atoms were placed in positions of optimized geometry, their isotropic displacement parameters were tied to those of the corresponding carrier atoms by a factor of either 1.2 or 1.5. The Squeeze procedure was applied to account for the volatile solvent molecules.[72] Olex2 was used to prepare material for publication[43]. Crystallographic data, data collection, and structure refinement details are given in Supplementary Table **8**. Two of the three PF6 anions were disordered. Two alternative orientations each were refined and resulted in site occupancies of 59.9(9) and 40.1(9) % for the atoms P2 – F26 and P2A – F26A, and of 59.2(6) and 40.8(6) % for the atoms P3 – F36 and P3A – F36A, respectively. Enhanced rigid bond restraints, as well as similarity and some pseudo-isotropic restraints were applied in the refinement of the anisotropic displacement parameters of the disordered fluorine atoms. For the atom pairs F12/F14 and P3/P3A same anisotropic displacement parameter constraints (EADP) were applied. CheckCIF reported two level B alerts that has been commented on as follows:

PLAT414_ALERT_2_B Short Intra D-H..H-X H8 ..H28A . 1.86 Ang. x,y,z = 1_555 Check

Author Response: The position of H8 was taken from a difference Fourier synthesis and the coordinates were allowed to ride on the carrier atom N8. H28A belongs to the methyl group at C28 and these H atoms were added in positions of optimized geometry and maximized electron density.

PLAT434_ALERT_2_B Short Inter HL..HL Contact F1 ..F1 . 2.25 Ang. 1/2-x,3/2-y,1-z = 7_566 Check

Author Response: This short F1 ... F1 contact can be traced back to the arrangement of the molecules observed in the crystallographic packing.

Supplementary Table 8 | Crystallographic data, data collection, and refinement details for $[(TIMMN^{Mes})Fe^{VI}(N)(F)]$-$(PF_6)_2$ • CH_2Cl_2 and $[(TIMMN^{Mes*})Fe^{V}(=N^*)(NCMe)](PF_6)_2MoF_6$ • 5 C_2H_3N and $[(TIMMN^{Mes*})Fe^{V}(NH^*)(F)](PF_6)_3$.

	$[(TIMMN^{Mes})Fe^{VI}(N)(F)]$ $(PF_6)_2$ • CH_2Cl_2 (**1** • CH_2Cl_2)<br><br>**WM2116A**<br>**CCDC-2258913**	$[(TIMMN^{Mes*})Fe^{V}(=N^*)(NCMe)]$ $(PF_6)_2MoF_6$ • 5 C_2H_3N (**3** • 5 C_2H_3N)<br><br>**MK2206**<br>**CCDC-2258914**	$[(TIMMN^{Mes*})Fe^{V}(NH^*)(F)]$ $(PF_6)_3$ (**2e**)<br><br>**MM489Fe6**<br>**CCDC-2297600**
Empirical formula	$C_{40}H_{47}Cl_2F_{13}FeN_8P_2$	$C_{51}H_{62}F_{18}FeMo_{1.02}N_{14}P_{1.98}$	$C_{39}H_{45}F_{19}FeN_8P_3$
Mol. Weight	1075.54	1428.17	1135.59
Crystal shape, color	block, orange	needle, orange	needle, orange
Crystal size [mm]	0.18 x 0.08 x 0.07	0.38 x 0.11 x 0.06	0.40 x 0.03 x 0.02
Temperature [K]	100	100	298
Crystal system	monoclinic	orthorhombic	monoclinic
Space group	$P2_1/n$ (no. 14)	*Pnma* (no. 62)	*C*2/*c* (no. 15)
a [Å]	11.6631(10)	25.4988(15)	28.425(2)
b [Å]	18.1351(16)	13.5933(8)	19.8259(14)
c [Å]	20.9895(19)	17.9406(11)	21.743(3)
α [°]	90	90	90
β [°]	96.206(3)	90	120.899(2)
γ [°]	90	90	90
V [Å^3]	4413.5(7)	6218.4(6)	10514(2)
Z	4	4	8
ρ [g cm^{-3}] (calc.)	1.619	1.525	1.435
μ [mm^{-1}]	0.633	0.591	0.482
F (000)	2200	2906	4616
T_{min}; T_{max}	0.707; 0.746	0.666; 0.746	0.647; 0.746
2Θ interval [°]	$3.8 \leq 2\ \Theta \leq 54.2$	$3.7 \leq 2\ \Theta \leq 57.4$	$3.9 \leq 2\ \Theta \leq 50.9$
Coll. Refl.	116135	125939	236540
Indep. Refl.; R_{int}	9734	8362	9699
Obs. refl. $F_0 \geq 4\sigma(F)$	8645	6714	7033

No. ref. parameters / restraints	755 / 243	555 / 174	756 / 822
wR_2 (all data)	0.1775	0.1346	0.2236
R_1 ($F_0 \geq 4\sigma(F)$)	0.0689	0.0549	0.0732
GooF on F^2	1.153	1.169	1.124
$\Delta\rho_{max/min}$ [e Å^{-3}]	1.025; –1.891	0.986; –0.862	0.697; –0.638

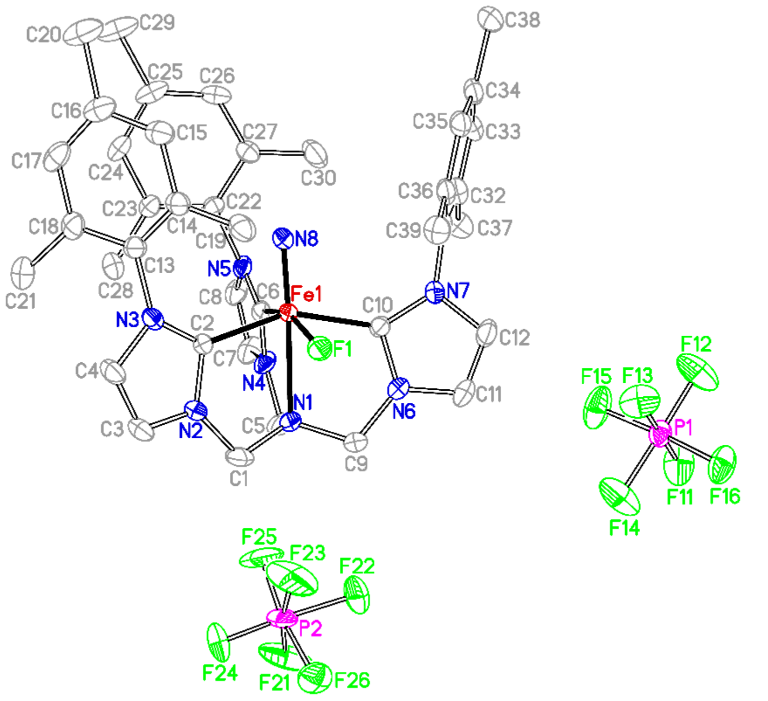

Supplementary Figure 20 | Solid-state molecular structure of **1** (CCDC-2258913) with the applied numbering scheme of [(TIMMN^{Mes})Fe^{VI}N(F)](PF$_6$)$_2$ in crystals of [(TIMMN^{Mes})Fe^{VI}(N)(F)](PF$_6$)$_2$ • CH$_2$Cl$_2$ (disorder, hydrogen atoms and co-crystalized solvent molecules omitted for clarity, thermal ellipsoids drawn at the 50 % probability level).

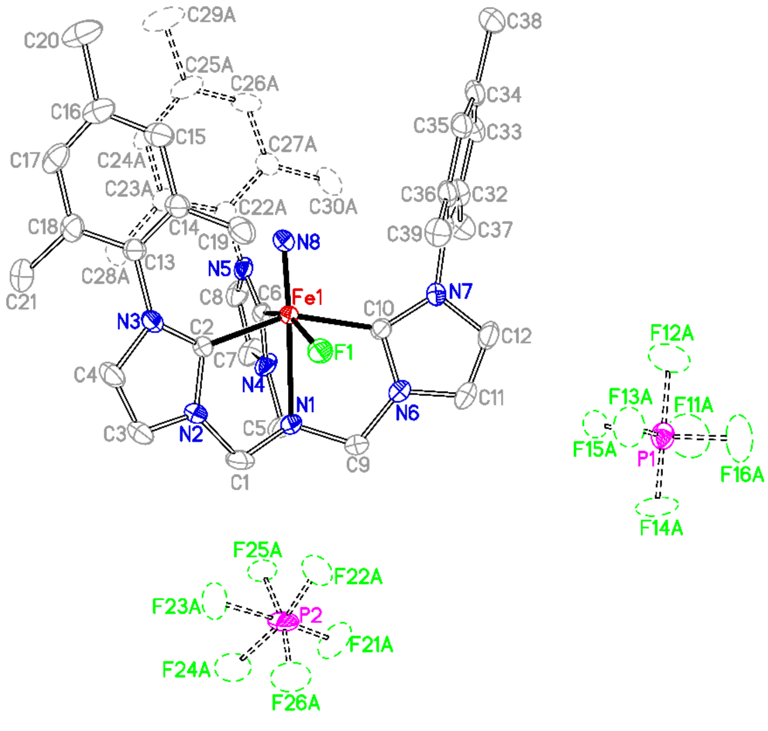

Supplementary Figure 21 | Solid-state molecular structure of **1** (CCDC-2258913) with the applied numbering scheme of [(TIMMN^{Mes})Fe^{VI}N(F)](PF$_6$)$_2$ in crystals of [(TIMMN^{Mes})Fe^{VI}(N)(F)](PF$_6$)$_2$ • CH$_2$Cl$_2$ showing only the minor component of the disordered moieties (hydrogen atoms and co-crystalized solvent molecules omitted for clarity, thermal ellipsoids drawn at the 50 % probability level).

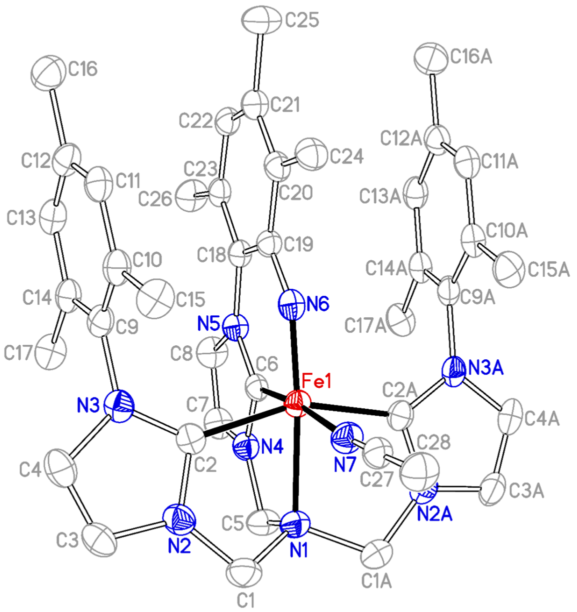

Supplementary Figure 22 | Solid-state molecular structure of **3** (CCDC-2258914) with the applied numbering scheme of [(TIMMN^{Mes*})Fe^{V}(=N*)(NCMe)]$^{3+}$ in crystals of [(TIMMN^{Mes*})Fe^{V}(=N*)(NCMe)](PF$_6$)$_2$MoF$_6$ • 5 C_2H_3N (anions, hydrogen atoms and co-crystalized solvent molecules omitted for clarity, thermal ellipsoids drawn at the 50 % probability level).

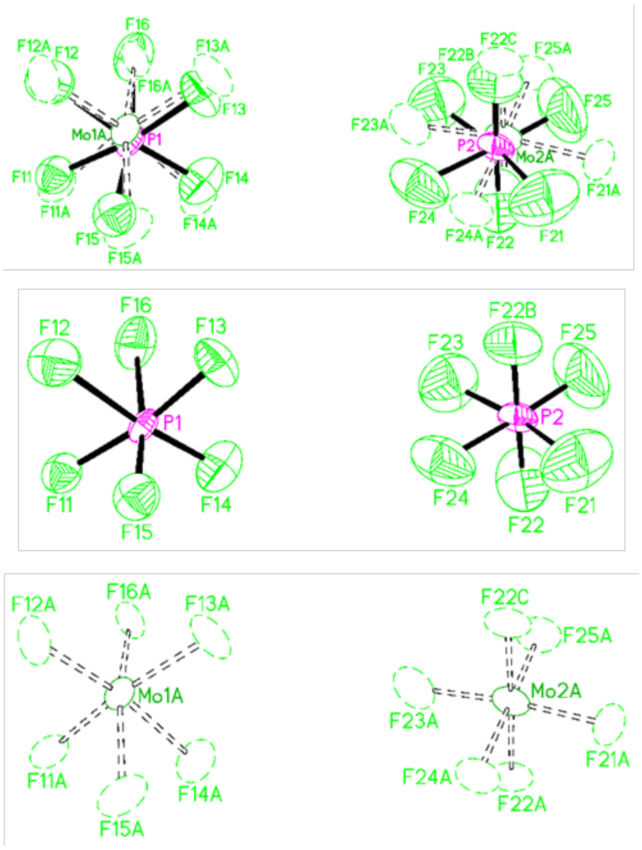

Supplementary Figure 23 | Solid-state molecular structure with the applied numbering scheme of the disordered PF$_6^-$ and MoF$_6^-$ anions in crystals of [(TIMMN^{Mes*})Fe^{V}(=N*)(NCMe)](PF$_6$)$_2$MoF$_6$ • 5 C_2H_3N (**3**, CCDC-2258914) (thermal ellipsoids drawn at the 50 % probability level). *Top*: overlay of the disordered PF$_6^-$ and MoF$_6^-$ anions; center: molecular structures of the PF$_6^-$ anions only; *bottom*: molecular structures of the MoF$_6^-$ anions only.

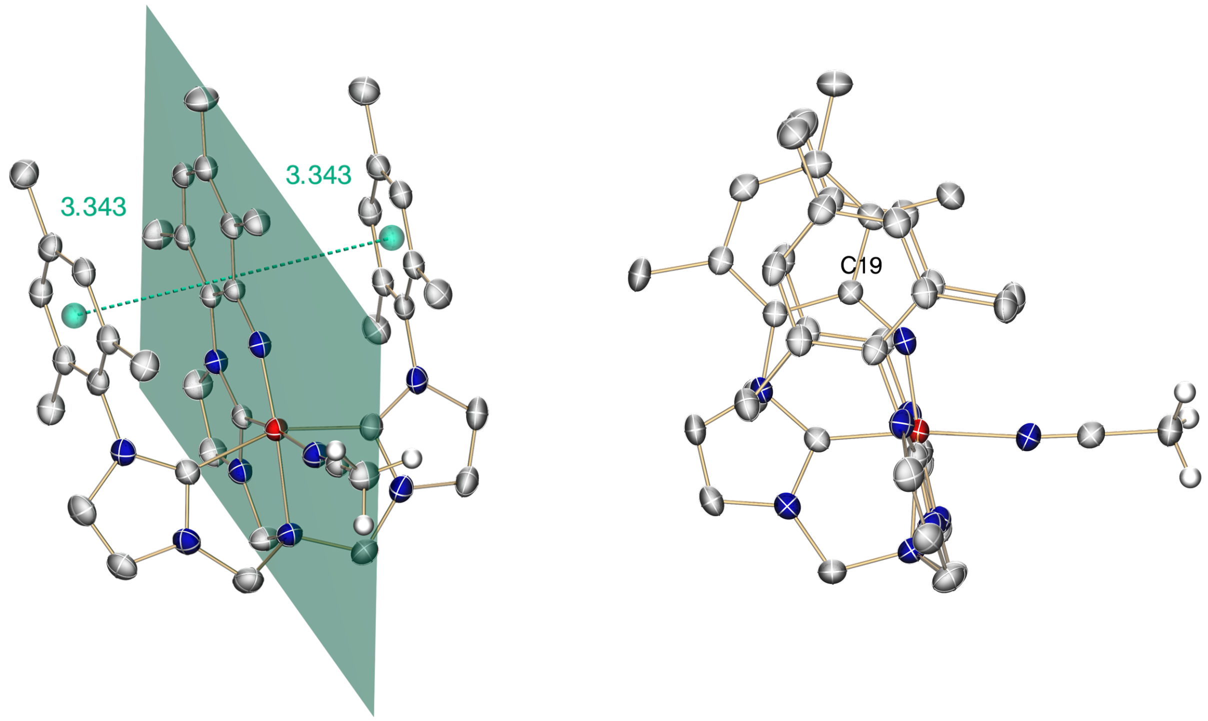

Supplementary Figure 24 | Solid-state molecular structure representations of $[(TIMMN^{Mes*})Fe^{V}(=N^{*})(NCMe)]^{3+}$ in crystals of $[(TIMMN^{Mes*})Fe^{V}(=N^{*})(NCMe)](PF_6)_2MoF_6 \cdot 5\ C_2H_3N$ (**3**, CCDC-2258914) (anions, hydrogen atoms and co-crystalized solvent molecules omitted for clarity, thermal ellipsoids drawn at the 50 % probability level) highlighting the similarity of a graphene-type fashion with an intramolecular aryl–aryl distance of 3.343 Å.

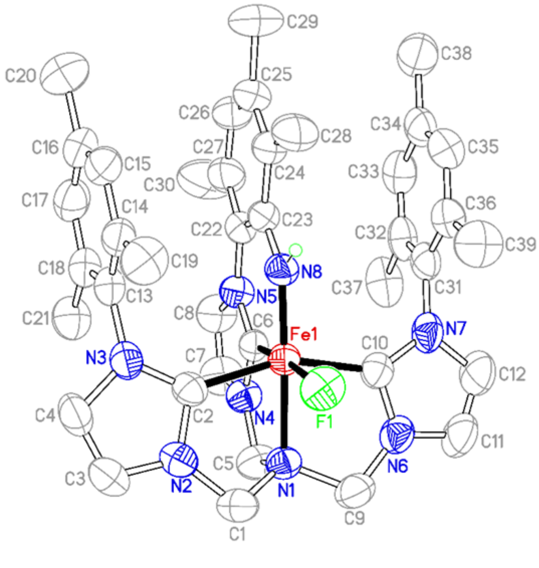

Supplementary Figure 25 | Solid-state molecular structure with the applied numbering scheme of $[(TIMMN^{Mes*})$-$Fe^{V}(NH^{*})(F)]^{3+}$ (**2e**, CCDC-2297600) in crystals of $[(TIMMN^{Mes*})Fe^{V}(NH^{*})(F)](PF_6)_3$ (anions and hydrogen atoms, with the exception of the N8-bound hydrogen, omitted for clarity, thermal ellipsoids drawn at the 50 % probability level).

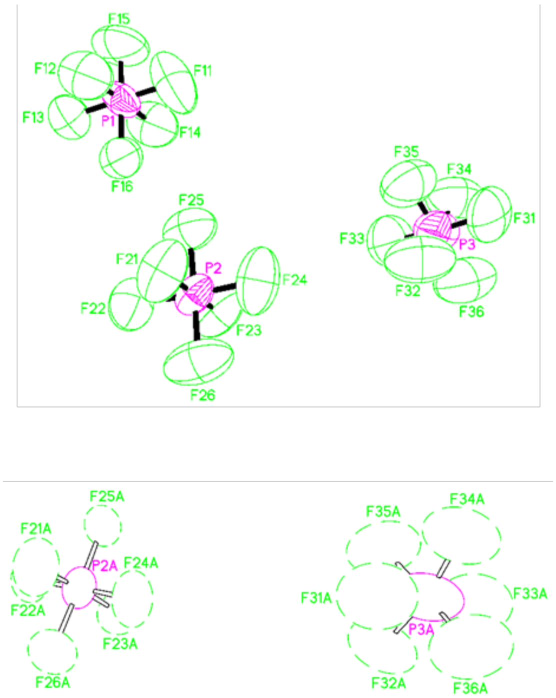

Supplementary Figure 26 | Solid-state molecular structure with the applied numbering scheme of the PF_6^- anions in crystals of $[(TIMMN^{Mes*})Fe^{V}(NH^{*})(F)](PF_6)_3$ (**2e**, CCDC-2297600) (thermal ellipsoids drawn at the 50 % probability level). *Top*: molecular structures of the first orientation of the PF_6^- anions only; *bottom*: molecular structures of the second orientation of the PF_6^- anions only (at P2A and P3A only).

Computational Details

All computations were performed with ORCA v5.0.1–4[7]. The NPA charges were calculated with NBO 7.0[46], Bader charges with AIMAll v.19.10.12[47]. Orbitals and spin densities were visualized with IboView v20211019-RevA[48] or ChemCraft[49].

DFT Calculations: The geometric parameters of all compounds were optimized using the PBE functional[12] and D3-dispersion correction with Becke-Johnson (BJ) damping[25,26]. Scalar relativistic effects were modeled within the "Zeroth Order Regular Approximation" (ZORA)[20]. The ZORA-def2-SVP basis set was used for all light elements, whereas the ZORA-def2-TZVP basis set was used for iron[50]. The RI approximation in combination with the auxiliary basis set SARC/J was used to speed up these calculations[22]. Tighter-than-default scf ("*tightscf*") and optimization criteria ("*tightopt*") were applied. All calculated structures were verified as true minima by the absence ($N^{\text{imag}} = 0$) of imaginary eigen¬values in the harmonic vibrational frequency analysis. Molecular properties (TD-DFT, CASSCF/NEVPT2[51,52], Mössbauer) and single point energies were calculated at the triple-ζ level of theory (ZORA-def2-TZVPP)[50]. In addition to the PBE functional, the PBE0[13,53],TPPSh[54] and ωB97X-D4[55,56] hybrid functionals were used for comparison. Energies, Bader charges, IBOs[57], Mössbauer spectra, Hirshfeld-, Mulliken-[58], Löwdin-[59], ChElPG-[60] (*LARGE* gridsize) and NPA[61] charges (atomic spin densities, respectively) were computed/refined by single-point calculations with the def2-TZVPP basis set for all atoms. The TD-DFT absorption spectra were computed with the def2-TZVPP basis set (150 roots, *maxdim* = 5, Tamm-Dancoff approximation). For the hybrid functionals the RIJCOSX approximation in combination with the related auxiliary basis set SARC/J was used[22]. For the computation of Mössbauer parameters, the SC-XRD structures were used, with the position of all hydrogen atoms optimized ("*optimizehydrogens* true"; PBE-D3/def2-SVP). Where SC-XRD structures were not available (**2**), structures were fully optimized as described before (ZORA-PBE-D3/def2-SVP) with and without truncating the methyl groups at the mesityl substituents. Truncation of the mesityl-methyl groups by hydrogen atoms was found to shift spin-density to the ligand in case of **2** (*vide infra*), whereas no substantial influence of methyl-group truncation was found for **I'**, **I, 1** and **e**. For the population analyses of PF_6^- adducts, structures were both optimized with and without the SMD implicit solvent model[24] in acetonitrile and charges are given as such. Figure 3B in the manuscript shows the values obtained with explicit (PF_6^-) and implicit (SMD=MeCN) solvation (Supplementary Tables 11 and 13). The computation of the Mössbauer spectra was carried out using the Douglas-Kroll-Hess method truncated at second order (DKH2)[62,63] in combination with the BP86[64-66], B3LYP[67] and TPSSh[54] functionals as delineated in the literature (calibration parameters from full test-set)[68]. For the light atoms, the DKH-def2-TZVPP[50,69] basis set was used, while for iron the core property basis set CP(PPP) was used[38]. Effects of solvation were investigated in much detail (*vide infra*) for **2**, using either (i) the implicit solvent model CPCM[70] with the default acetonitrile parameters, tailored parameters for SO_2 at −60 °C (ε = 21.73[71], and n = 1.361[72]) for the mechanism depicted in Supplementary Figs. 27–30, the SMD model[24], and a combination with explicit solvation by one, two, or three hexafluorophosphate anions (see Supplementary Fig. 36, 38 and 54).

Overall, the DFT computational analyses (atomic partial charges Supplementary Tables 10–13 and Supplementary Figures 31–34; spin densities, Supplementary Figures 35–38; localized orbitals Supplementary Tables 15–20; solvation, Supplementary Figures 31–38) suggest delocalization of the unpaired electron in 2, with the main locus being the iron center. The analysis of atomic partial charges (Hirshfeld, ChElPG, Bader, and to a minor extent Löwdin, Mulliken, NPA) with all investigated computational methods suggests a correlation between the Mössbauer isomer shift and the oxidation of the metal (Supplementary Tables 10, 11 and Figures 31–34). Corresponding trends are obtained for the nitrido- and carbene atoms.

CASSCF calculations: ZORA and the related def2-TZVPP basis set were applied. The structural parameters obtained in the solid state were used for the complexes in compounds **I'**, **I**, **1** and **e**, whereas for **2** the structural parameters obtained by ZORA-PBE-D3/def-SVP were used. Test calculations using the structural parameters obtained with PBE0 or TPSSh gave consistent results. Using the structural parameters obtained by optimization with implicit solvation have smaller deviation from the solid-state structures (Supplementary Table 9), yet the effect on the computed electronic structure is negligible (Supplementary Table 22). For these calculations, the three methyl groups of each mesityl substituent were truncated by hydrogen atoms and the position of all hydrogen atoms optimized ("*optimizehydrogens true*"). In the case of complex **2**, it was found in the DFT calculations that the methyl groups have a considerable influence on the distribution of the spin density (Supplementary Figure 37). Thus, the methyl groups of the mesityl substituents were NOT truncated by hydrogen atoms. The *autoaux* procedure was used to generate the Aux/C auxiliary basis set[73]. For the NEVPT2 computations, no core electrons were frozen ("*nofrozencore*") and the state-averaging roots (if applicable) are given in the captions of Supplementary Figures 40, 41 and 43, 44. Several active spaces were investigated (Supplementary Tables 21, 22). These comprise "minimal" CASSCF(9,9), including all orbitals involving the iron ion (Supplementary Figure 49),

complemented by a solvation study (Supplementary Figure 54). Further, CASSCF(11,10) was computed, which additionally includes one NHC-based π-orbital (Supplementary Figure 50). Additionally, CASSCF(15,14) with the inclusion of five π/π^* orbitals on the mesityl rings (Supplementary Figure 51), CASSCF(15,15) with the inclusion of three π/π^* orbital pairs on the mesityl rings (ΔE = −0.033 Eh; Supplementary Figure 52)), and CASSCF(15,15) including the three π/π^* NHC orbitals pairs (ΔE = −0.066 Eh; Supplementary Figure 53 were calculated. **All active spaces except CASSCF(15,14), which predicts a mesityl-based radical, suggest a *bona-fide* Fe(VII) ion.** Higher levels of theories, albeit desirable, proved computationally too demanding.

Supplementary Table 9 | Benchmark of structural parameters obtained with PBE-D3/def2-SVP, TPSSh-D3/def2-SVP and PBE0-D3/def2-SVP with these found in the solid state.

	Bonds/Angles	XRD	PBE		PBE (CPCM=MeCN)		TPSSh		TPSSh (CPCM=MeCN)		PBE0		PBE0 (CPCM=MeCN)	
		d/ Å or α/ °	d/ Å or α/ °	Δ	d/ Å or α/ °	Δ	d/ Å or α/ °	Δ	d/ Å or α/ °	Δ	d/ Å or α/ °	Δ	d/ Å or α/ °	Δ
**I'**	0Fe–12C	1.936	1.904	−0.033	1.907	−0.029	1.910	−0.026	1.915	−0.021	1.916	−0.021	1.921	−0.016
	0Fe–20C	1.932	1.902	−0.030	1.906	−0.026	1.909	−0.023	1.914	−0.018	1.911	−0.021	1.919	−0.013
	0Fe–28C	1.944	1.902	−0.042	1.906	−0.039	1.908	−0.037	1.913	−0.031	1.909	−0.035	1.915	−0.030
	0Fe–1N	2.422	2.571	+0.149	2.501	+0.079	2.529	+0.107	2.461	+0.039	2.597	+0.175	2.513	+0.091
	0Fe–8N	1.526	1.511	−0.015	1.522	−0.004	1.500	−0.026	1.513	−0.013	1.479	−0.047	1.494	−0.032
	8N-0Fe-12C	105.746	106.784	+1.038	106.464	+0.718	106.807	+1.061	106.516	+0.770	108.762	+3.016	107.542	+1.796
	8N-0Fe-20C	107.953	106.949	−1.004	106.500	−1.453	107.305	−0.648	106.773	−1.180	108.665	+0.712	108.106	+0.153
	8N-0Fe-28C	106.363	107.358	+0.995	106.733	+0.370	106.795	+0.432	106.193	−0.170	106.206	−0.157	106.027	−0.336
	1N-0Fe-12C	73.754	73.016	−0.738	73.472	−0.282	73.134	−0.620	73.550	−0.204	72.234	−1.520	72.807	−0.947
	1N-0Fe-20C	73.054	72.978	−0.076	73.418	+0.364	73.041	−0.013	73.524	+0.470	72.197	−0.857	72.851	−0.203
	1N-0Fe-28C	73.190	72.916	−0.274	73.413	+0.223	72.919	−0.271	73.446	+0.256	71.973	−1.217	72.684	−0.506
	12C-0Fe-20C	110.235	112.014	+1.779	112.054	+1.819	111.687	+1.452	111.867	+1.632	109.577	−0.658	110.623	+0.388
	20C-0Fe-28C	110.259	111.712	+1.453	112.205	+1.946	111.756	+1.497	112.433	+2.174	112.067	+1.808	112.079	+1.820
	28C-0Fe-12C	115.843	111.668	−4.175	112.382	−3.461	112.117	−3.726	112.533	−3.310	111.427	−4.416	112.171	−3.672
	**rmsd (distances)**		**0.072**		**0.043**		**0.054**		**0.026**		**0.084**		**0.046**	
	**rmsd (angles)**		**1.713**		**1.567**		**2.022**		**1.516**		**2.037**		**1.546**	
**I**	0Fe–12C	1.958	1.922	−0.036	1.922	−0.036	1.934	−0.025	1.934	−0.025	1.957	−0.001	1.939	−0.019
	0Fe–20C	1.975	1.934	−0.041	1.934	−0.041	1.949	−0.027	1.949	−0.026	1.957	−0.019	1.957	−0.018
	0Fe–C28	1.944	1.908	−0.036	1.908	−0.036	1.916	−0.028	1.917	−0.027	1.941	−0.003	1.921	−0.023
	0Fe–1N	2.329	2.394	+0.065	2.394	+0.065	2.350	+0.022	2.350	+0.021	2.468	+0.139	2.361	+0.032
	0Fe–8N	1.529	1.520	−0.009	1.520	−0.009	1.507	−0.022	1.507	−0.022	1.475	−0.054	1.486	−0.043
	8N-0Fe-12C	104.794	104.982	+0.188	105.013	+0.219	104.819	+0.025	104.775	−0.019	110.065	+5.271	104.422	−0.372
	8N-0Fe-20C	106.654	107.205	+0.551	107.210	+0.556	106.802	+0.148	106.806	+0.152	107.980	+1.326	107.073	+0.419
	8N-0Fe-28C	99.917	100.746	+0.829	100.756	+0.839	100.771	+0.854	100.782	+0.865	99.490	−0.427	101.398	+1.481
	1N-0Fe-12C	76.133	75.757	−0.376	75.754	−0.379	76.009	−0.124	76.033	−0.100	73.940	−2.193	75.898	−0.235
	1N-0Fe-20C	75.296	74.693	−0.603	74.665	−0.631	74.770	−0.526	74.773	−0.523	73.372	−1.924	74.595	−0.701
	1N-0Fe-28C	76.444	75.608	−0.836	75.596	−0.848	75.801	−0.643	75.807	−0.637	73.917	−2.527	75.749	−0.695
	12C-0Fe-20C	125.107	127.881	+2.774	127.825	+2.718	129.294	+4.187	129.334	+4.227	121.011	−4.096	129.426	+4.319
	20C-0Fe-28C	107.808	106.814	−0.994	106.794	−1.014	106.291	−1.517	106.229	−1.579	108.279	+0.471	105.643	−2.165
	28C-0Fe-12C	109.597	106.072	−3.525	106.117	−3.480	105.456	−4.14	105.505	−4.092	107.865	−1.732	105.647	−3.950
	**rmsd (distances)**		**0.042**		**0.042**		**0.024**		**0.024**		**0.067**		**0.029**	
	**rmsd (angles)**		**1.610**		**1.593**		**2.067**		**2.070**		**2.680**		**2.173**	
**1**	0Fe–13C	1.976	1.942	−0.034	1.935	−0.042	1.936	−0.040	1.937	−0.040	1.932	−0.044	1.934	−0.043
	0Fe–21C	1.967	1.948	−0.020	1.946	−0.021	1.950	−0.018	1.952	−0.015	1.948	−0.019	1.949	−0.018
	0Fe–29C	1.984	1.997	+0.013	1.973	−0.011	1.980	−0.004	1.971	−0.013	1.972	−0.012	1.966	−0.018
	0Fe–2N	2.351	2.580	+0.229	2.421	+0.070	2.408	+0.057	2.360	+0.009	2.433	+0.082	2.381	+0.030
	0Fe–9N	1.519	1.504	−0.015	1.507	−0.012	1.489	−0.029	1.491	−0.027	1.465	−0.054	1.468	−0.050
	0Fe-1F	1.868	1.846	−0.022	1.852	−0.016	1.820	−0.047	1.828	−0.039	1.809	−0.058	1.819	−0.049
	9N-0Fe-13C	102.981	102.453	−0.528	102.557	−0.424	102.331	−0.650	102.956	−0.025	103.071	+0.090	103.479	+0.498
	9N-0Fe-21C	92.754	95.387	+2.633	94.096	+1.342	94.919	+2.165	94.118	+1.364	95.022	+2.268	94.015	+1.261
	9N-0Fe-29C	103.639	110.565	+6.926	107.772	+4.133	106.800	+3.161	106.028	+2.389	106.460	+2.821	106.138	+2.499
	9N-0Fe-1F	108.649	109.836	+1.187	107.63	−1.019	108.346	−0.303	107.509	−1.140	108.501	−0.148	107.372	−1.277
	2N-0Fe-13C	78.691	76.192	−2.499	77.903	−0.788	78.205	−0.486	78.568	−0.123	77.948	−0.743	78.232	−0.459
	2N-0Fe-21C	76.815	73.467	−3.348	75.261	−1.554	75.450	−1.365	75.829	−0.986	75.196	−1.619	75.533	−1.282
	2N-0Fe-29C	77.126	73.764	−3.362	74.670	−2.456	75.184	−1.942	75.039	−2.087	75.004	−2.122	74.830	−2.296
	2N-0Fe-1F	81.775	81.319	−0.456	83.012	+1.237	81.277	−0.498	82.548	+0.773	81.280	−0.495	83.082	+1.307
	13C-0Fe-21C	96.045	94.769	−1.280	95.248	−0.797	94.240	−1.810	94.437	−1.608	93.907	−2.140	94.326	−1.719
	21C-0Fe-29C	93.422	93.747	+0.325	95.014	+1.592	95.445	+2.023	95.351	+1.929	95.158	+1.736	95.226	+1.804
	29C-0Fe-1F	80.553	78.156	−2.400	78.828	−1.725	78.998	−1.550	79.407	−1.146	79.084	−1.470	79.337	−1.216
	1F-0Fe-13C	80.759	79.746	−1.010	80.142	−0.617	80.238	−0.520	80.543	−0.216	80.480	−0.280	80.726	−0.033
	**rmsd (distances)**		**0.096**		**0.035**		**0.037**		**0.027**		**0.051**		**0.041**	
	**rmsd (angles)**		**2.800**		**1.761**		**1.616**		**1.369**		**1.604**		**1.707**	
**e**	0Fe–11C	1.959	1.957	−0.002	1.965	+0.006	1.960	+0.001	1.969	+0.010	1.964	+0.005	1.973	+0.014
	0Fe–12C	1.842	1.842	−0.000	1.834	−0.008	1.852	+0.009	1.845	+0.003	1.850	+0.008	1.846	+0.003
	0Fe–15C	1.960	1.961	+0.001	1.964	+0.004	1.963	+0.003	1.969	+0.009	1.965	+0.005	1.972	+0.012
	0Fe–2N	1.772	1.771	−0.001	1.763	−0.009	1.788	+0.016	1.780	+0.008	1.781	+0.009	1.780	+0.008
	0Fe–3N	2.206	2.200	−0.006	2.181	−0.025	2.155	−0.051	2.133	−0.073	2.156	−0.050	2.126	−0.080
	0Fe-1F	1.818	1.818	+0.000	1.828	+0.010	1.793	−0.025	1.806	−0.012	1.783	−0.035	1.798	−0.020
	2N-0Fe-11C	101.172	101.214	+0.042	101.249	+0.077	100.597	−0.575	100.499	−0.673	100.471	−0.701	100.756	−0.416
	2N-0Fe-12C	89.125	89.070	−0.055	89.006	−0.119	88.458	−0.667	88.519	−0.606	88.699	−0.426	88.721	−0.404
	2N-0Fe-15C	102.622	102.454	−0.168	103.246	+0.624	102.164	−0.458	102.61	−0.012	102.230	−0.392	102.041	−0.581
	2N-0Fe-1F	91.355	91.225	−0.130	90.762	−0.593	92.617	+1.262	91.954	+0.599	92.938	+1.583	92.210	+0.855
	3N-0Fe-11C	77.800	77.717	−0.083	77.599	−0.201	78.033	+0.233	78.137	+0.337	78.161	+0.361	78.312	+0.512
	3N-0Fe-12C	82.591	82.739	+0.148	83.144	+0.553	83.343	+0.752	83.621	+1.030	82.961	+0.370	83.361	+0.770
	3N-0Fe-15C	78.148	78.332	+0.184	77.362	−0.786	78.726	+0.578	77.990	−0.158	78.594	+0.446	78.043	−0.105
	3N-0Fe-1F	96.926	96.967	+0.041	97.081	+0.155	95.575	−1.351	95.901	−1.025	95.397	−1.529	95.706	−1.220
	11C-0Fe-12C	88.481	88.547	+0.066	87.235	−1.246	88.076	−0.405	86.674	−1.807	87.761	−0.720	86.308	−2.173
	12C-0Fe-15C	88.419	88.207	−0.212	87.625	−0.794	87.618	−0.801	86.872	−1.547	87.432	−0.987	86.597	−1.822
	15C-0Fe-1F	91.623	91.719	+0.096	92.874	+1.251	92.482	+0.859	93.362	+1.739	91.688	+0.065	93.386	+1.763
	1F-0Fe-11C	91.277	91.406	+0.129	92.361	+1.084	91.394	+0.117	92.898	+1.621	91.688	+0.411	93.336	+2.059
	**rmsd (distances)**		**0.003**		**0.012**		**0.024**		**0.031**		**0.026**		**0.035**	
	**rmsd (angles)**		**0.126**		**0.746**		**0.759**		**1.108**		**0.806**		**1.263**	

**3**	0Fe–11C	1.976	1.959	−0.017	1.958	−0.018	1.960	−0.016	1.958	−0.018	1.956	−0.019	1.954	−0.022
	0Fe–17C	1.855	1.850	−0.005	1.847	−0.008	1.852	−0.003	1.849	−0.007	1.848	−0.008	1.846	−0.009
	0Fe–57C	1.976	1.956	−0.020	1.957	−0.019	1.955	−0.020	1.956	−0.019	1.955	−0.021	1.953	−0.022
	0Fe–1N	2.025	2.238	+0.213	2.214	+0.189	2.195	+0.170	2.176	+0.150	2.208	+0.183	2.186	+0.161
	0Fe–6N	1.680	1.673	−0.006	1.668	−0.011	1.673	−0.006	1.667	−0.013	1.662	−0.017	1.657	−0.022
	0Fe–7N	2.025	2.016	−0.009	1.983	−0.042	2.013	−0.012	1.983	−0.042	2.026	+0.000	1.991	−0.034
	6N-0Fe-11C	101.783	101.613	−0.170	102.0	+0.259	101.213	−0.570	101.589	−0.194	101.331	−0.452	101.554	−0.229
	6N-0Fe-17C	85.924	86.573	+0.649	86.793	+0.869	86.401	+0.477	86.636	+0.712	86.737	+0.813	87.006	+1.082
	6N-0Fe-57C	101.783	101.587	−0.196	101.710	−0.073	101.198	−0.585	101.349	−0.434	101.126	−0.657	101.343	−0.440
	6N-0Fe-7N	100.989	100.950	−0.039	100.504	−0.485	101.259	+0.270	100.725	−0.264	101.409	+0.429	100.825	−0.164
	1N-0Fe-11C	78.198	78.668	+0.470	78.089	−0.109	79.099	+0.901	78.538	+0.340	78.863	+0.665	78.461	+0.263
	1N-0Fe-17C	82.643	81.280	−1.363	81.651	−0.992	81.706	−0.937	82.007	−0.636	81.322	−1.321	81.579	−1.064
	1N-0Fe-57C	78.198	78.239	+0.041	78.152	−0.046	78.481	+0.283	78.475	+0.277	78.671	+0.473	78.590	+0.392
	1N-0Fe-7N	90.444	91.199	+0.755	91.052	+0.608	90.636	+0.192	90.633	+0.189	90.532	+0.088	90.591	+0.147
	11C-0Fe-17C	89.567	89.375	−0.192	89.416	−0.151	88.960	−0.607	89.284	−0.283	89.305	−0.262	89.437	−0.130
	17C-0Fe-57C	89.567	90.048	+0.481	89.490	−0.077	90.081	+0.514	89.551	−0.016	89.597	+0.030	89.217	−0.350
	57C-0Fe-7N	89.014	88.747	−0.267	89.103	+0.089	88.835	−0.179	89.164	+0.150	88.873	−0.141	89.096	+0.082
	7N-0Fe-11C	89.014	88.798	−0.216	88.974	−0.040	89.133	+0.119	89.059	+0.045	89.038	+0.024	89.125	+0.111
	**rmsd (distances)**		**0.088**		**0.080**		**0.070**		**0.065**		**0.076**		**0.069**	
	**rmsd (angles)**		**0.542**		**0.454**		**0.536**		**0.358**		**0.576**		**0.498**	

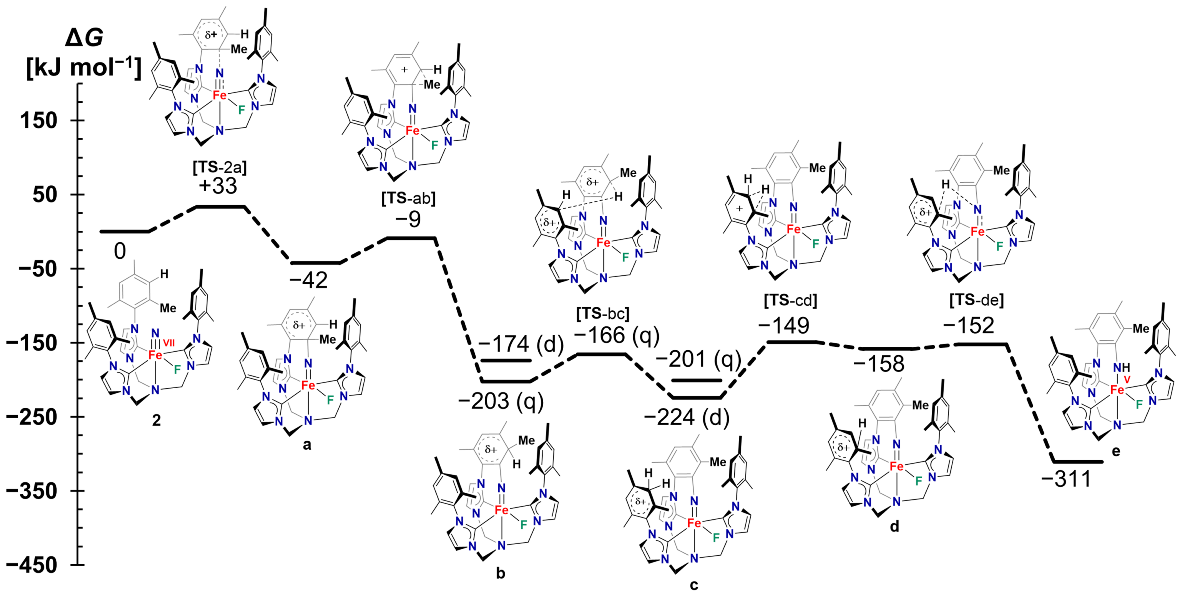

Supplementary Figure 27 | Full mechanism for the rearrangement of **2** to **e** with d, q representing the doublet and quartet states respectively. TPSSh-D3(CPCM=SO_2)/def2-TZVPP//PBE-D3/def2-SVP).

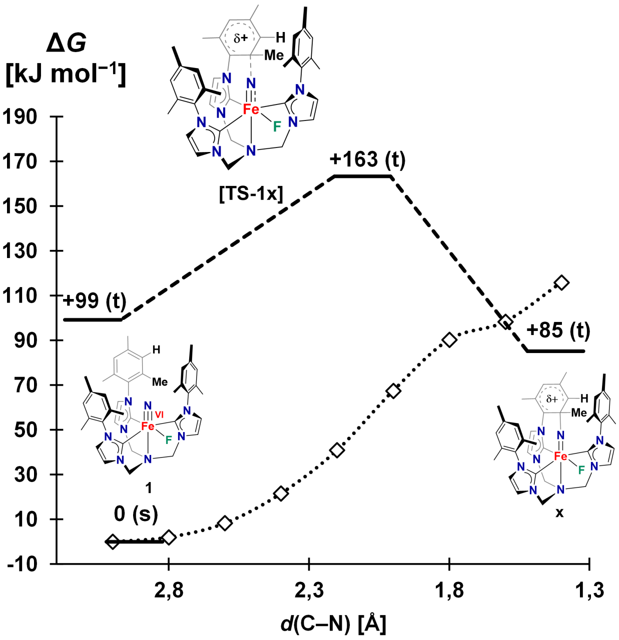

Supplementary Figure 28 | Reaction energy profile for the reaction of the nitride in **1** with a mesityl group (ZORA-TPSSh-D3(CPCM)/def2-TZVPP//ZORA-PBE-D3/def2-SVP). The calculations reveal that this transformation is endergonic by ΔG = +85 kJ mol^{-1} and associated with intersystem crossing to the triplet state, whereas the addition product with a singlet ground state is predicted to be unstable; hence, a reaction coordinate scan is shown instead.

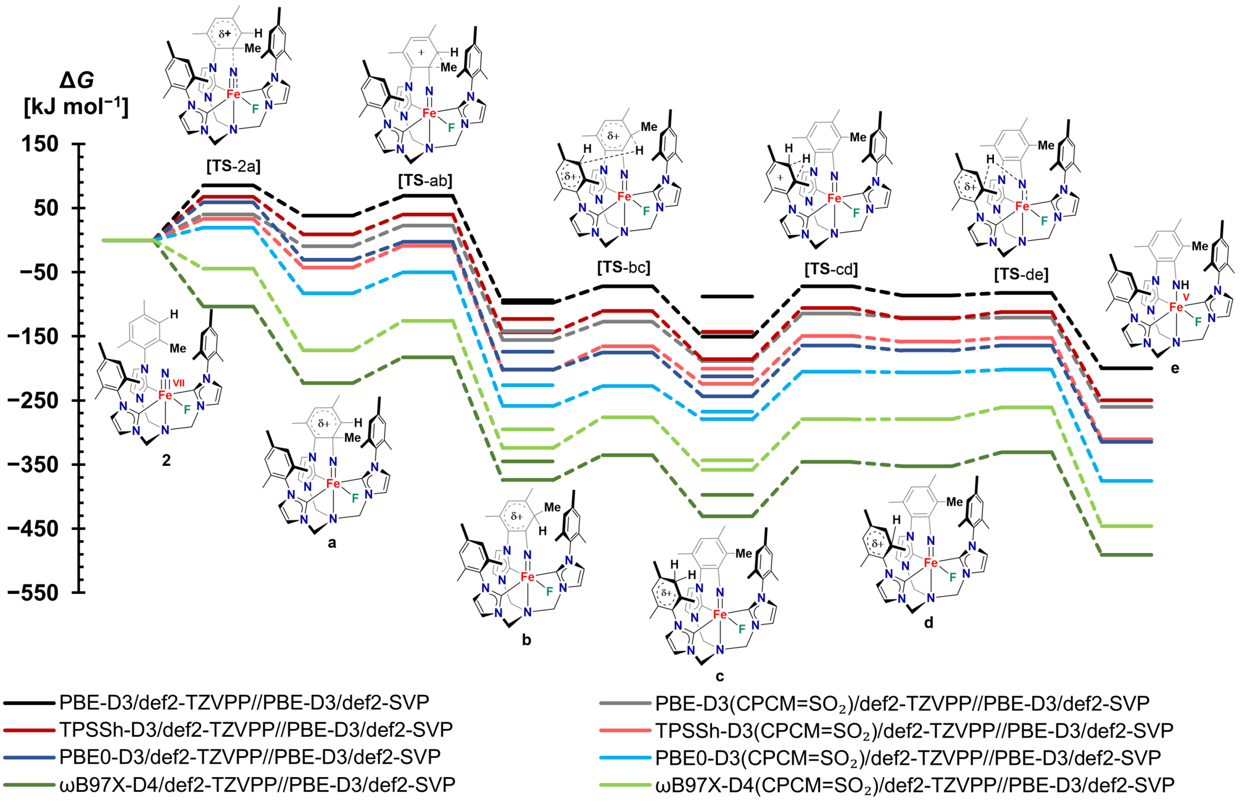

Supplementary Figure 29 | Comparison of the computational strategies for the full mechanism for the rearrangement of **2** to **e** with d, q representing the doublet and quartet states, respectively. PBE-D3/def2-TZVPP//PBE-D3/def2-SVP (black), PBE-D3 (CPCM=SO_2)/def2-TZVPP//PBE-D3/def2-SVP (grey), TPSSh-D3/def2-TZVPP//PBE-D3/def2-SVP (dark red), TPSSh-D3 (CPCM=SO_2)/def2-TZVPP//PBE-D3/def2-SVP (light red) , PBE0-D3/def2-TZVPP//PBE-D3/def2-SVP (dark blue), PBE0-D3 (CPCM=SO_2)/def2-TZVPP//PBE-D3/def2-SVP (light blue), ωB97X-D4/def2-TZVPP//PBE-D3/def2-SVP (dark green), ωB97X-D4 (CPCM=SO_2)/def2-TZVPP//PBE-D3/def2-SVP (light green).

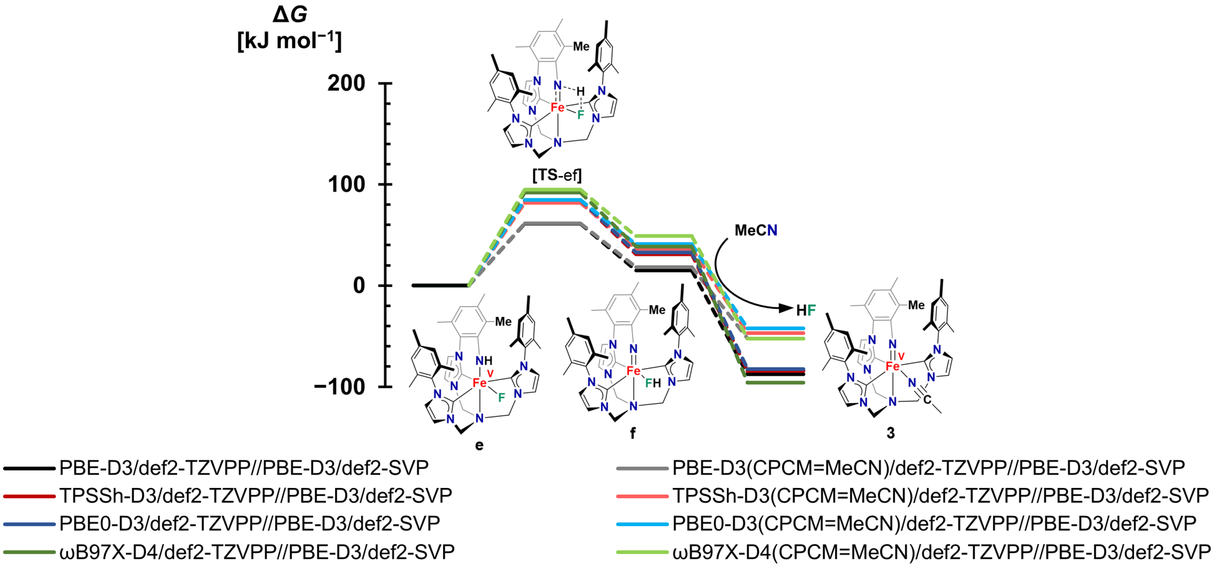

Supplementary Figure 30 | Comparison of the computational strategies for the full mechanism for the formation of **e** to **3**. PBE-D3/def2-TZVPP//PBE-D3/def2-SVP (black), PBE-D3 (CPCM=SO_2)/def2-TZVPP//PBE-D3/def2-SVP (grey), TPSSh-D3/def2-TZVPP//PBE-D3/def2-SVP (dark red), TPSSh-D3 (CPCM=SO_2)/def2-TZVPP//PBE-D3/def2-SVP (light red), PBE0-D3/def2-TZVPP//PBE-D3/def2-SVP (dark blue), PBE0-D3 (CPCM=SO_2)/def2-TZVPP//PBE-D3/def2-SVP (light blue), ωB97X-D4/def2-TZVPP//PBE-D3/def2-SVP (dark green), ωB97X-D4 (CPCM=SO_2)/def2-TZVPP//PBE-D3/def2-SVP (light green).

Supplementary Table 10 | Partial atomic charges for the iron atom as obtained by CASSCF and DFT (PBE-D3/def2-TZVPP, TPSSh-D3/def2-TZVPP, PBE0-D3/def2-TZVPP) wave functions (gas phase). For **2**, CASSCF(15,15) including the NHC's π -orbitals was used. For structures obtained from optimization with SMD=MeCN, the implicit solvent model was also used for the calculation of charges.

	**Method**	**I'**	**I**	**1**	**2**
Mulliken atomic charge on Fe [a.u.]	CASSCF	+1.086	+1.097	+1.136	+0.991
	PBE	+0.368	+0.292	+0.320	+0.258
	TPSSh	+0.362	+0.324	+0.388	+0.331
	PBE0	+0.378	+0.369	+0.426	+0.371
Löwdin atomic charge on Fe [a.u.]	CASSCF	−0.602	−0.280	−0.335	−0.100
	PBE	−0.628	−0.484	−0.570	−0.526
	TPSSh	−0.624	−0.450	−0.537	−0.516
	PBE0	−0.652	−0.447	−0.547	−0.526
Hirshfeld atomic charge on Fe [a.u.]	PBE	−0.004	+0.104	+0.157	+0.184
	TPSSh	+0.005	+0.133	+0.186	+0.197
	PBE0	−0.017	+0.130	+0.177	+0.188
ChElPG atomic charge on Fe [a.u.]	CASSCF	−0.336	+0.224	+0.909	+0.998
	PBE	−0.451	−0.109	+0.562	+0.614
	TPSSh	−0.463	−0.051	+0.603	+0.677
	PBE0	−0.516	−0.066	+0.604	+0.683
Bader atomic charge on Fe [a.u.]	CASSCF	+1.36	+1.70	+1.92	+2.02
	PBE	+0.62	+1.095	+1.27	+1.29
	TPSSh	+0.58	+1.17	+1.35	+1.35
	PBE0	+1.00	+1.17	+1.33	+1.34
NPA atomic charge on Fe [a.u.]	PBE	+0.25	+0.44	+0.40	+0.44
	TPSSh	+0.29	+0.60	+0.46	+0.48
	PBE0	+0.30	+0.53	+0.47	+0.49

Supplementary Table 11 | Partial atomic charges for the iron atom as obtained by DFT (PBE-D3/def2-TZVPP, TPSSh-D3/def2-TZVPP, PBE0-D3/def2-TZVPP) wave functions from neutral structures through explicit solvation with PF_6^- ions. For structures obtained from optimization with SMD=MeCN, the implicit solvent model was also used for the calculation of charges, while otherwise the computations were carried out in gas phase.

	**Method**	**I'·PF$_6$**	**I'·PF$_6$ (SMD=MeCN)**	**I·2PF$_6$**	**I·2PF$_6$ (SMD=MeCN)**	**1·2PF$_6$**	**1·2PF$_6$ (SMD=MeCN)**	**2·3PF$_6$**	**2·3PF$_6$ (SMD=MeCN)**
Mulliken atomic charge on Fe [a.u.]	PBE	+0.460	+0.578	+0.288	+0.311	+0.420	+0.455	+0.445	+0.491
	TPSSh	+0.492	+0.639	+0.381	+0.388	+0.518	+0.540	+0.532	+0.576
	PBE0	+0.469	+0.592	+0.366	+0.348	+0.511	+0.542	+0.471	+0.530
Löwdin atomic charge on Fe [a.u.]	PBE	−0.644	−0.672	−0.552	−0.513	−0.570	−0.570	−0.526	−0.520
	TPSSh	−0.642	−0.667	−0.527	−0.481	−0.540	−0.540	−0.513	−0.508
	PBE0	−0.665	−0.687	−0.533	−0.482	−0.544	−0.543	−0.528	−0.523
Hirshfeld atomic charge on Fe [a.u.]	PBE	+0.014	−0.014	+0.110	+0.110	+0.161	+0.160	+0.192	+0.191
	TPSSh	+0.022	−0.004	+0.135	+0.139	+0.188	+0.188	+0.207	+0.206
	PBE0	+0.002	−0.022	+0.127	+0.135	+0.182	+0.183	+0.195	+0.193
ChElPG atomic charge on Fe [a.u.]	PBE	+0.027	+0.370	+0.496	+0.703	+1.265	+1.478	+0.551	+0.938
	TPSSh	+0.025	+0.362	+0.494	+0.753	+1.288	+1.484	+0.568	+0.945
	PBE0	+0.074	+0.417	+0.535	+0.791	+1.345	+1.540	+0.588	+0.969
Bader atomic charge on Fe [a.u.]	PBE	+0.99	+0.99	+1.14	+1.11	+1.26	+1.26	+1.295	+1.30
	TPSSh	+1.04	+1.04	+1.21	+1.18	+1.33	+1.33	+1.35	+1.35
	PBE0	+1.01	+1.02	+1.20	+1.18	+1.31	+1.32	+1.33	+1.33
NPA atomic charge on Fe [a.u.]	PBE	+0.27	+0.27	+0.42	+0.43	+0.41	+0.41	+0.45	+0.46
	TPSSh	+0.31	+0.32	+0.50	+0.52	+0.47	+0.47	+0.48	+0.48
	PBE0	+0.32	+0.34	+0.53	+0.57	+0.48	+0.48	+0.48	+0.49

Supplementary Table 12 | Partial atomic charges for the nitrido atom as obtained by CASSCF and DFT (PBE-D3/def2-TZVPP, TPSSh-D3/def2-TZVPP, PBE0-D3/def2-TZVPP) wave functions (gas phase). For **2**, CASSCF(15,15) including the NHC's π-orbitals was used. For structures obtained from optimization with SMD=MeCN, the implicit solvent model was also used for the calculation of atomic partial charges.

	Method	**I'**	**I**	**1**	**2**
Mulliken atomic charge on N [a.u.]	CASSCF	−0.502	−0.202	−0.063	+0.048
	PBE	−0.380	−0.256	−0.130	−0.156
	TPSSh	−0.457	−0.296	−0.167	−0.229
	PBE0	−0.405	−0.232	−0.094	−0.162
Löwdin atomic charge on N [a.u.]	CASSCF	+0.064	+0.287	+0.398	+0.529
	PBE	+0.119	+0.206	+0.285	+0.279
	TPSSh	+0.094	+0.198	+0.292	+0.262
	PBE0	+0.089	+0.212	+0.312	+0.277
Hirshfeld atomic charge on N [a.u.]	PBE	−0.224	−0.133	−0.065	−0.069
	TPSSh	−0.248	−0.138	−0.056	−0.083
	PBE0	−0.257	−0.125	−0.039	−0.070
ChElPG atomic charge on N [a.u.]	CASSCF	−0.144	−0.077	−0.112	+0.040
	PBE	−0.272	−0.197	−0.268	−0.238
	TPSSh	−0.290	−0.202	−0.253	−0.232
	PBE0	−0.304	−0.197	−0.241	−0.238
Bader atomic charge on N [a.u.]	CASSCF	−0.48	−0.09	+0.07	+0.29
	PBE	−0.54	−0.42	−0.34	−0.31
	TPSSh	−0.58	−0.43	−0.30	−0.63
	PBE0	−0.60	−0.41	−0.28	−0.32
NPA atomic charge on N [a.u.]	PBE	−0.16	−0.03	+0.17	+0.15
	TPSSh	−0.19	−0.04	+0.18	+0.13
	PBE0	−0.21	−0.04	+0.21	+0.15

Supplementary Table 13 | Partial atomic charges for the nitrido atom as obtained by DFT (PBE-D3/def2-TZVPP, TPSSh-D3/def2-TZVPP, PBE0-D3/def2-TZVPP) wave functions from neutral structures through explicit solvation with PF_6^- ions. For structures obtained from optimization with SMD=MeCN, the implicit solvent model was also used for the calculation of charges, while otherwise the computations were carried out in gas phase.

	Method	**I'·PF_6**	**I'·PF_6 (SMD=MeCN)**	**I·2PF_6**	**I·2PF_6 (SMD=MeCN)**	**1·2PF_6**	**1·2PF_6 (SMD=MeCN)**	**2·3PF_6**	**2·3PF_6 (SMD=MeCN)**
Mulliken atomic charge on N [a.u.]	PBE	−0.552	−0.612	−0.443	−0.511	−0.263	−0.289	−0.293	−0.333
	TPSSh	−0.610	−0.688	−0.514	−0.562	−0.313	−0.340	−0.336	−0.381
	PBE0	−0.547	−0.616	−0.423	−0.479	−0.202	−0.228	−0.224	−0.271
Löwdin atomic charge on N [a.u.]	PBE	+0.105	+0.043	+0.172	+0.145	+0.266	+0.248	+0.274	+0.267
	TPSSh	+0.082	+0.013	+0.161	+0.135	+0.272	+0.257	+0.282	+0.274
	PBE0	+0.075	+0.001	+0.167	+0.142	+0.293	+0.277	+0.300	+0.290
Hirshfeld atomic charge on N [a.u.]	PBE	−0.221	−0.286	−0.159	−0.186	−0.091	−0.107	−0.070	−0.076
	TPSSh	−0.244	−0.316	−0.167	−0.194	−0.083	−0.098	−0.063	−0.070
	PBE0	−0.252	−0.329	−0.162	−0.187	−0.065	−0.080	−0.047	−0.056
ChElPG atomic charge on N [a.u.]	PBE	−0.228	−0.397	−0.208	−0.329	−0.253	−0.361	−0.147	−0.231
	TPSSh	−0.244	−0.430	−0.211	−0.341	−0.244	−0.341	−0.133	−0.221
	PBE0	−0.265	−0.468	−0.213	−0.347	−0.237	−0.336	−0.120	−0.213
Bader atomic charge on N [a.u.]	PBE	−0.57	−0.65	−0.47	−0.50	−0.35	−0.37	−0.32	-0.33
	TPSSh	−0.61	−0.70	−0.49	−0.52	−0.34	−0.36	−0.32	-0.33
	PBE0	−0.62	−0.72	−0.48	−0.51	−0.32	−0.34	−0.30	-0.29
NPA atomic charge on N [a.u.]	PBE	−0.21	−0.30	−0.10	−0.14	+0.11	+0.09	+0.11	+0.10
	TPSSh	−0.25	−0.35	−0.12	−0.16	+0.13	+0.11	+0.14	+0.12
	PBE0	−0.27	−0.38	−0.12	−0.16	+0.16	+0.14	+0.16	+0.16

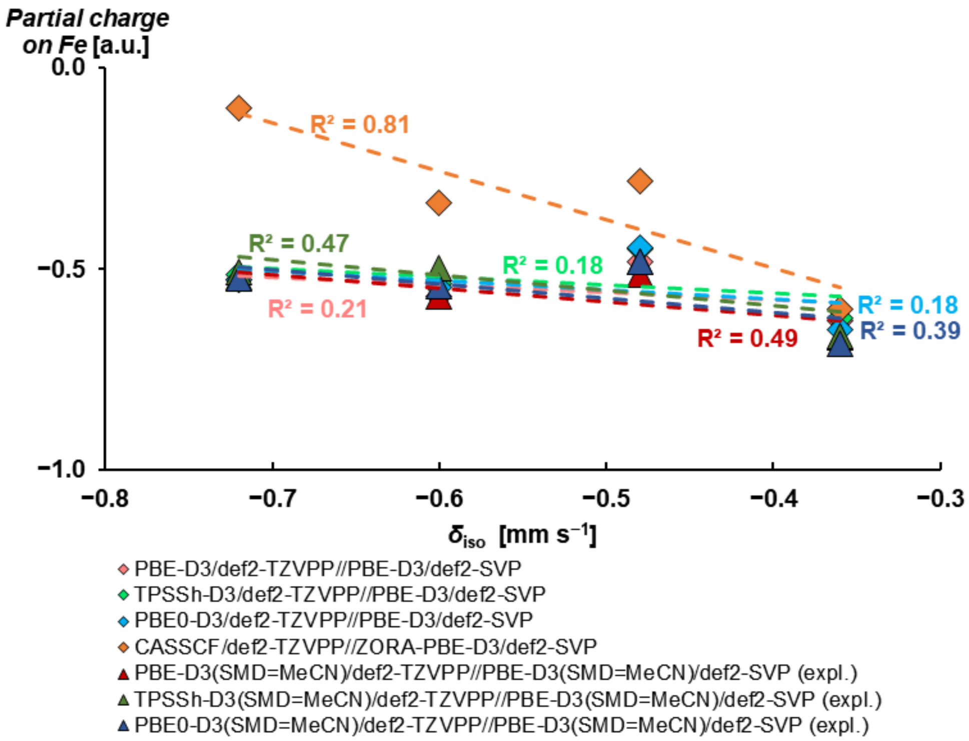

Supplementary Figure 31 | Correlation between the experimental ^{57}Fe Mössbauer isomer shifts with the computed Löwdin atomic charges on Fe (**I'**, **I**, **1**, **2**). Data points without solvation are marked with diamonds and lighter color and data points with explicit and implicit solvation (SMD=MeCN) are marked with triangles and darker color.

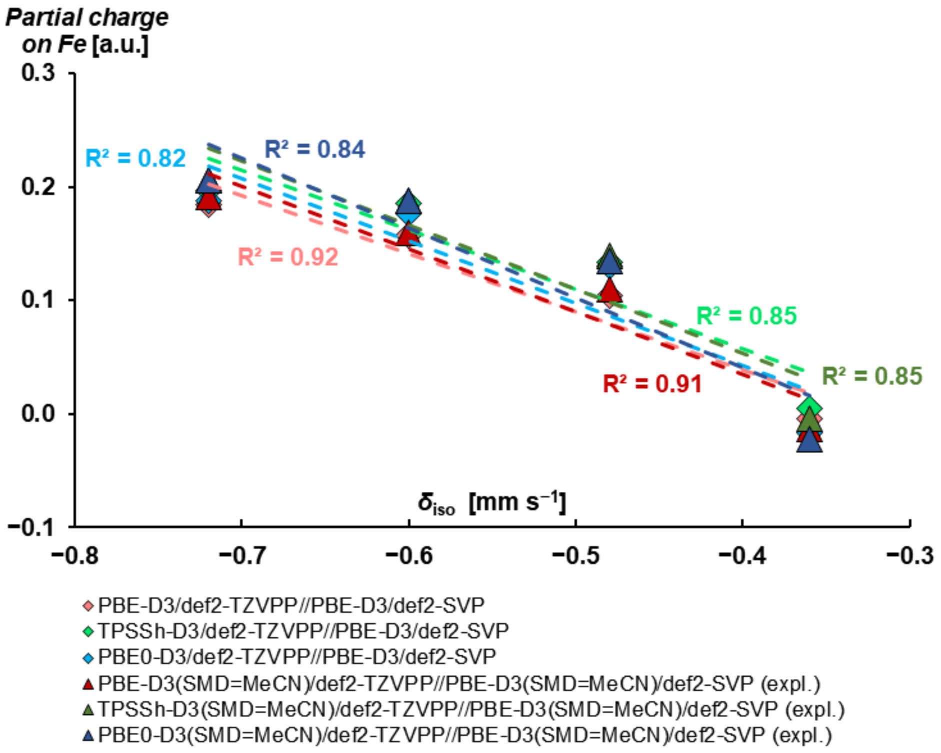

Supplementary Figure 32 | Correlation between the experimental ^{57}Fe Mössbauer isomer shifts with the computed Hirshfeld atomic charges on Fe (**I'**, **I**, **1**, **2**). Data points without solvation are marked with diamonds and lighter color and data points with explicit and implicit solvation (SMD=MeCN) are marked with triangles and darker color.

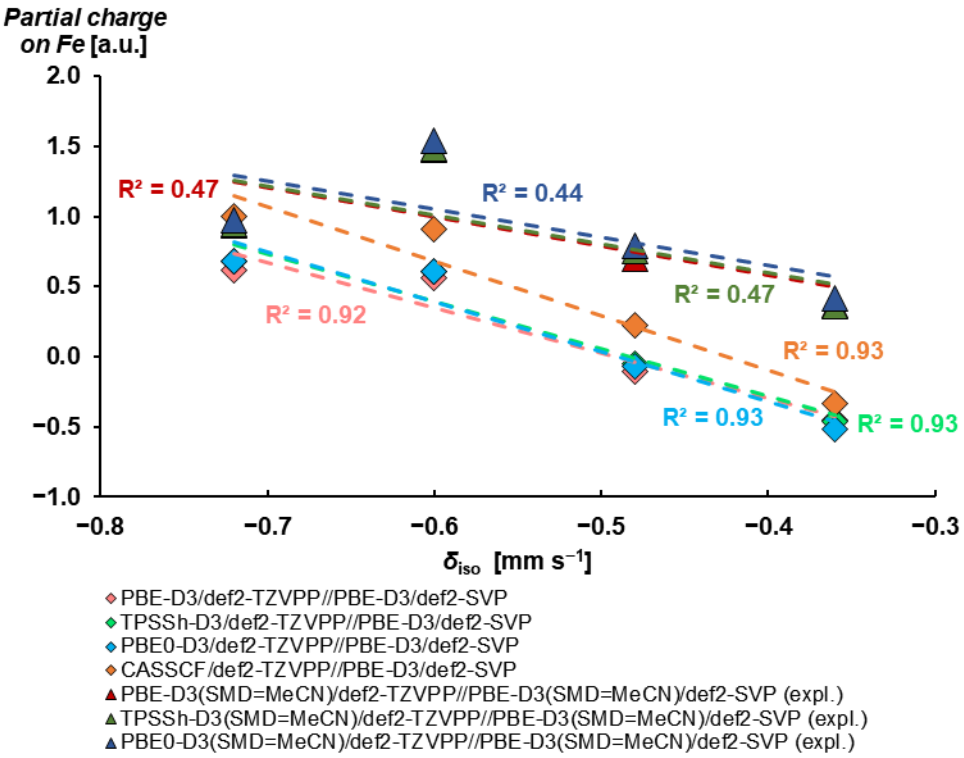

Supplementary Figure 33 | Correlation between the experimental ^{57}Fe Mössbauer isomer shifts with the computed ChElPG atomic charges on Fe (**I'**, **I**, **1**, **2**). Data points without solvation are marked with diamonds and lighter color and data points with explicit and implicit solvation (SMD=MeCN) are marked with triangles and darker color.

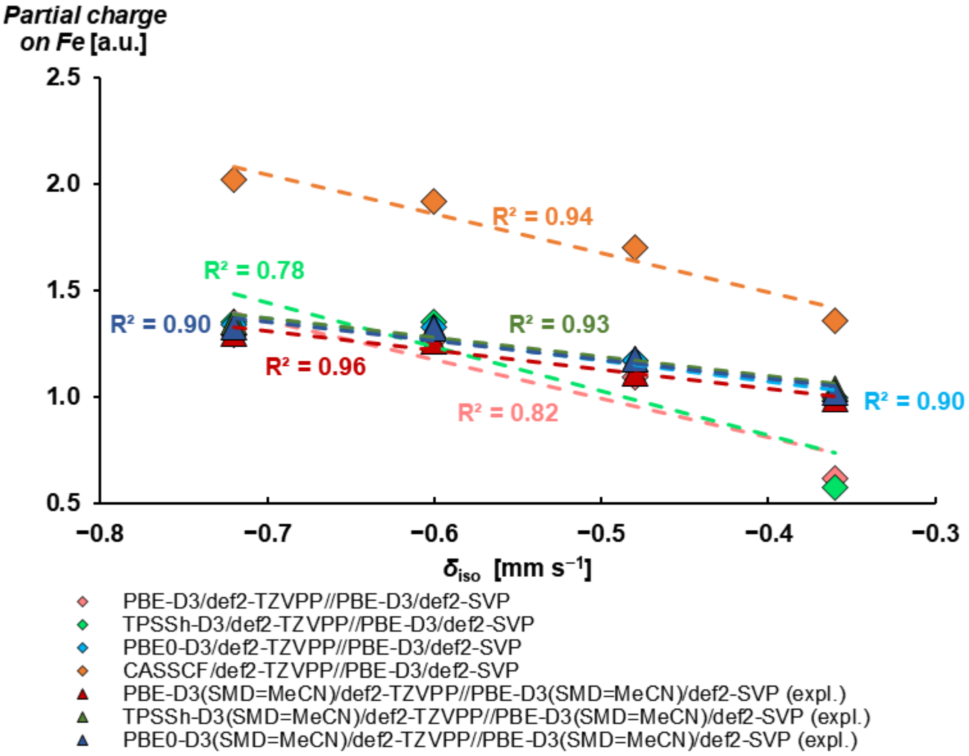

Supplementary Figure 34 | Correlation between the experimental ^{57}Fe Mössbauer isomer shifts with the computed Bader atomic charges on Fe (**I'**, **I**, **1**, **2**). Data points without solvation are marked with diamonds and lighter color and data points with explicit and implicit solvation (SMD=MeCN) are marked with triangles and darker color.

Supplementary Table 14 | Computed ^{57}Fe Mössbauer parameters (DKH2-BP86-D3/def2-TZVPP(Fe: CP(PPP)), DKH2-TPSSh-D3/def2-TZVPP(Fe: CP(PPP)), DKH2-B3LYP-D3/def2-TZVPP(Fe: CP(PPP)).

		I'	I	1	2	2·3PF$_6$	2·3PF$_6$ (CPCM= MeCN)	3
δ^{iso} [mm s^{-1}]	Measured	−0.36	−0.48	−0.60		−0.72		−0.16
	BP86	−0.45	−0.51	−0.54	−0.60	−0.59	−0.58	−0.11
	TPSSh	−0.37	−0.44	−0.50	−0.56	−0.07	−0.04	−0.10
	B3LYP	−0.37	−0.45	−0.52	−0.58	−0.57	−0.56	−0.10
ΔE_Q [mm s^{-1}]	Measured	5.28	3.85	4.12		2.9−3.2		2.30
	BP86	4.45	2.95	3.40	3.88	2.59	2.61	−2.00
	TPSSh	4.79	3.28	3.66	4.18	3.07	3.10	1.85
	B3LYP	5.18	3.61	3.93	4.49	3.58	3.76	1.84

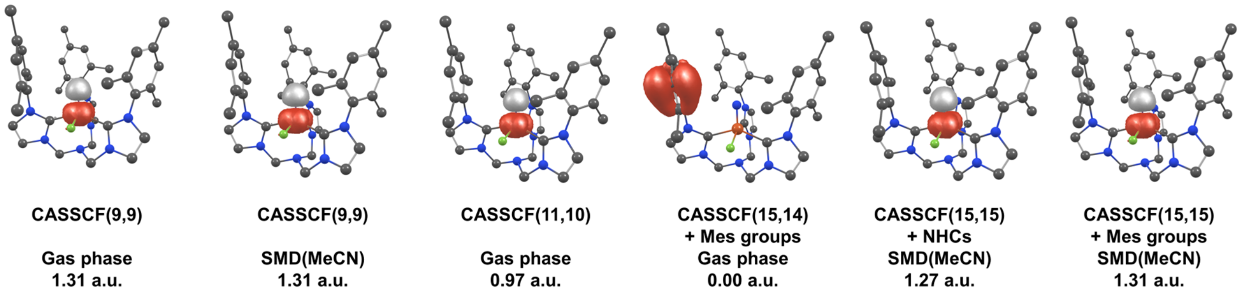

Supplementary Figure 35 | Spin density plots for **2** according to CASSCF(9,9), CASSCF(11,10), CASSCF(15,14), and CASSCF(15,15) including NHC or mesityl π-orbitals with and without implicit solvation (SMD=MeCN). Given values relate to atomic spin densities (Löwdin) at the iron atom.

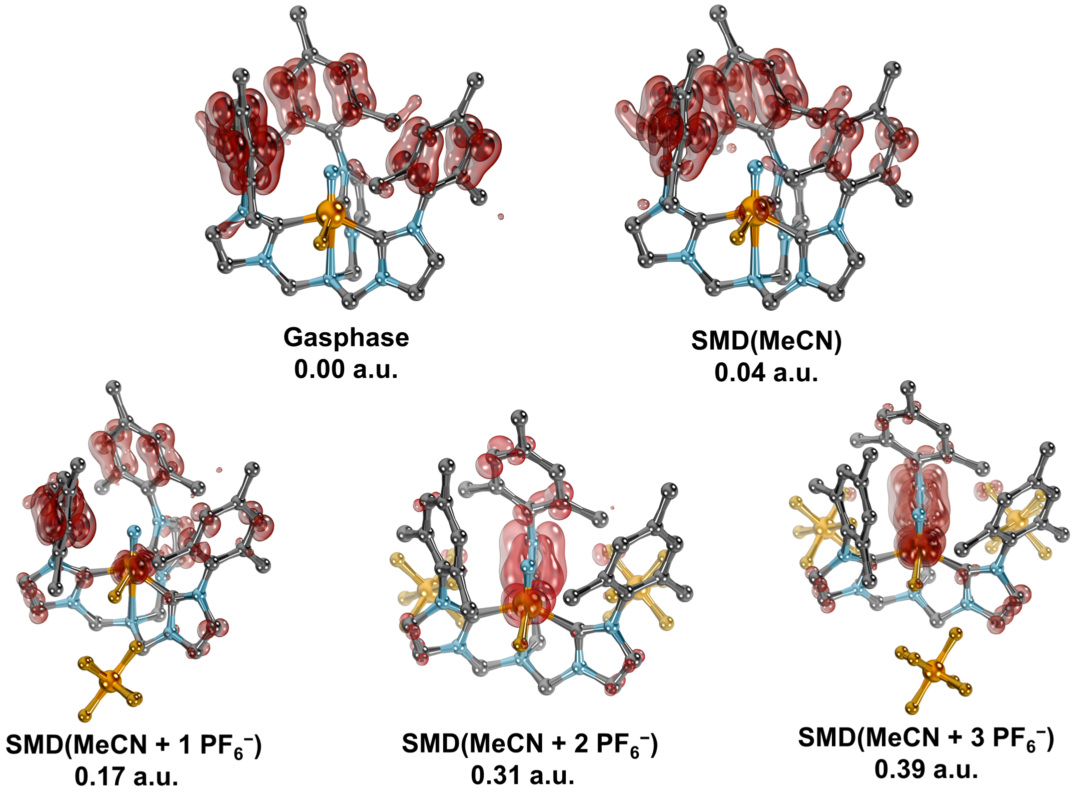

Supplementary Figure 36 | Effects of implicit (SMD=MeCN; *top*) and explicit solvation (one, two and three hexafluorophosphate anions; *bottom*) on the spin density in **2** (PBE-D3(SMD=MeCN)/def2-TZVPP//PBE-D3(SMD=MeCN)/def2-SVP). Given values relate to atomic spin densities (Hirshfeld) at the iron atom.

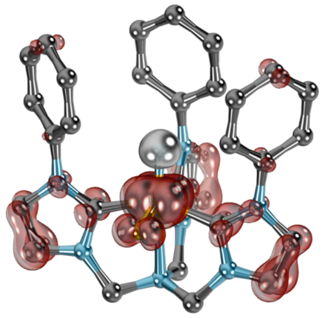

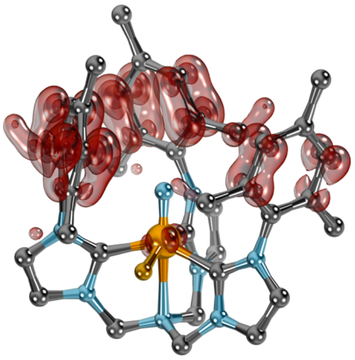

0.60 a.u. **0.04 a.u.**

Supplementary Figure 37 | Effects of methyl groups in mesityl substituents on spin density in **2** (PBE-D3(CPCM=MeCN)/def2-TZVPP//PBE-D3(CPCM=MeCN)/def2-SVP). Given values relate to atomic spin densities (Hirshfeld) at the iron atom.

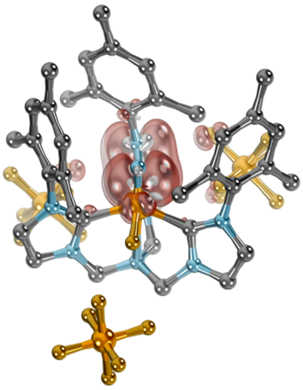

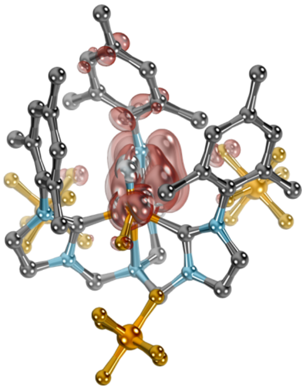

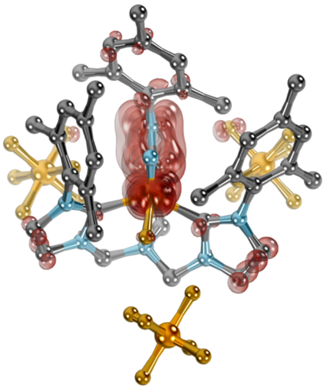

PBE0
0.08 a.u.

TPSSh
0.24 a.u.

PBE
0.39 a.u.

Supplementary Figure 38. | Effects of functional (PBE0, 0.25 HF exchange; TPSSh, 0.1 HF exchange; PBE, GGA) on spin density in **2** (functional-D3(SMD=MeCN)/def2-TZVPP//functional-D3(SMD=MeCN)/def2-TZVPP). Given values relate to atomic spin densities (Hirshfeld) at the iron atom.

Supplementary Table 15 | IBOs relating to the five 3d orbitals in **I** (mesityl-methyl groups truncated by hydrogen atoms) computed with PBE-D3/def2-TZVPP, TPSSh-D3/def2-TZVPP and PBE0-D3/def2-TZVPP. If a *d*-orbital partakes in several bonds, the MO with the highest contribution of iron is shown. The contribution of atoms is assessed based on the default Mulliken population analysis. The *α*-orbitals are shown and the populations for the *α* and *β* orbitals are noted in the form "*α*/ *β*". For SOMOs, the missing β electron is symbolized by "–".

	**PBE**	**TPSSh**	**PBE0**
$d_{x^2-y^2}$	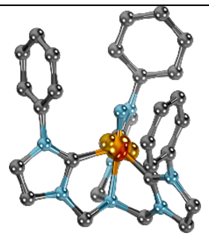	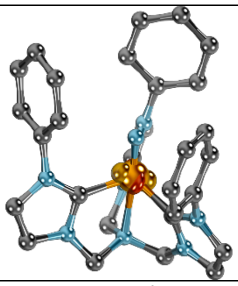	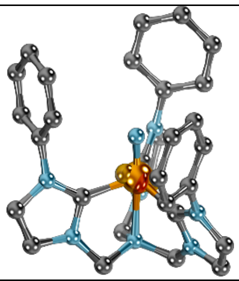
	Fe 0.95/ –	Fe 0.96/ –	Fe 0.97/ –
d_{xy}	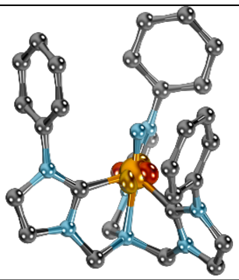	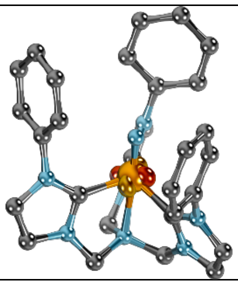	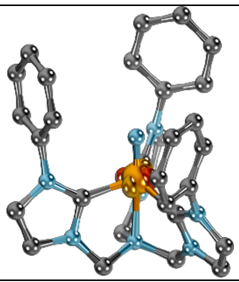
	Fe 0.91/Fe 0.89	Fe 0.94/ Fe 0.92	Fe 0.95/ Fe 0.93
d_{xz}	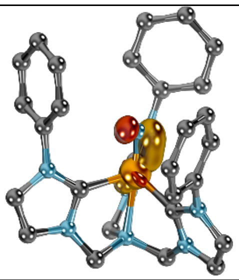	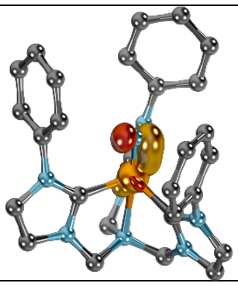	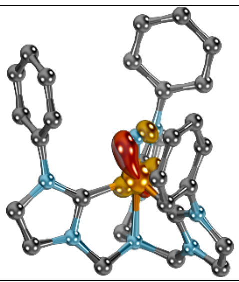
	Fe 0.47, N 0.52/ Fe 0.54, N 0.45	Fe 0.48, N 0.53/ Fe 0.56, N 0.44	Fe 0.62, N 0.37/ Fe 0.46, N 0.56
d_{yz}	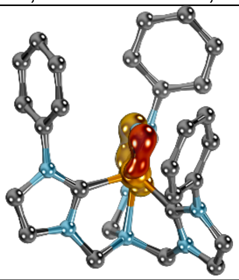	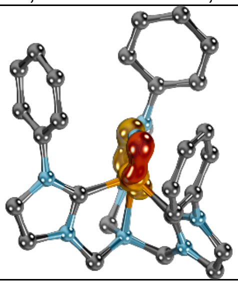	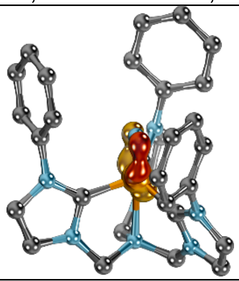
	Fe 0.55, N 0.42/ Fe 0.49, N 0.49	Fe 0.57, N 0.40/ Fe 0.48, N 0.50	Fe 0.70, N 0.26/ Fe 0.35, N 0.63
d_{z^2}	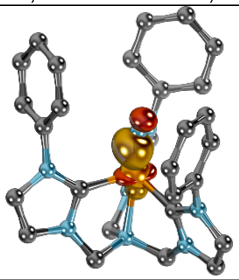	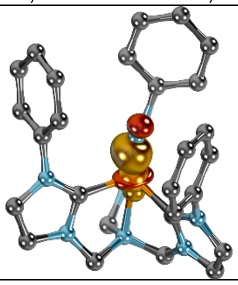	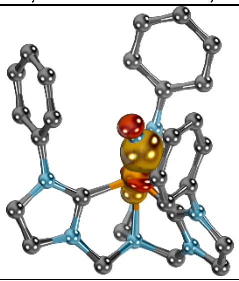
	Fe 0.52, N 0.50/ Fe 0.45, N 0.57	Fe 0.52, N 0.50/ Fe 0.44, N 0.58	Fe 0.54, N 0.47/ Fe 0.38, N 0.63

Supplementary Table 16 | IBOs relating to the five 3*d* orbitals in **1** (mesityl-methyl groups truncated by hydrogen atoms) computed with PBE-D3/def2-TZVPP, TPSSh-D3/def2-TZVPP and PBE0-D3/def2-TZVPP. If a *d*-orbital partakes in several bonds, the MO with the highest contribution of iron is shown. The contribution of atoms is assessed based on the default Mulliken population analysis.

	**PBE**	**TPSSh**	**PBE0**
$\boldsymbol{d_{x^2-y^2}}$	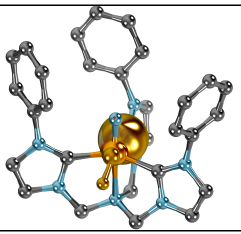	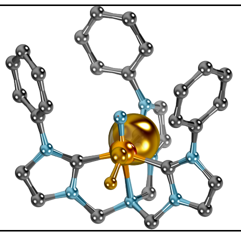	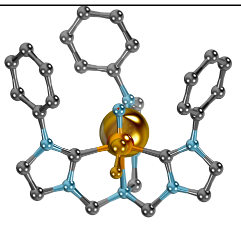
	Fe 0.35, C 0.65	Fe 0.33, C 0.67	Fe 0.33, C 0.67
$\boldsymbol{d_{xy}}$	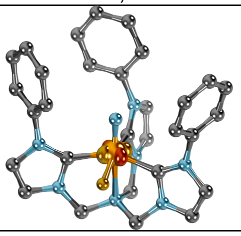	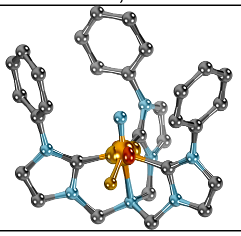	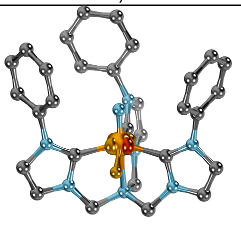
	Fe 0.94	Fe 0.96	Fe 0.98
$\boldsymbol{d_{xz}}$	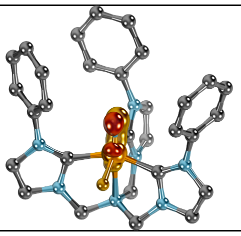	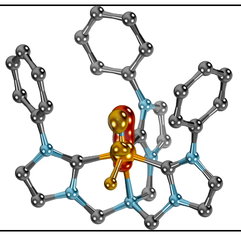	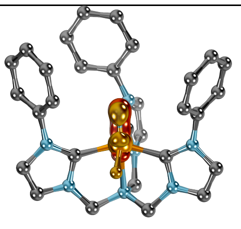
	Fe 0.57, N 0.43	Fe 0.57, N 0.43	Fe 0.58, N 0.42
$\boldsymbol{d_{yz}}$	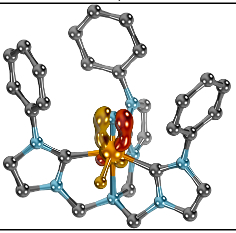	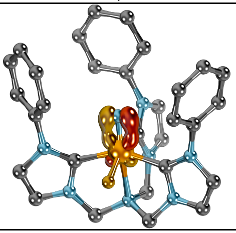	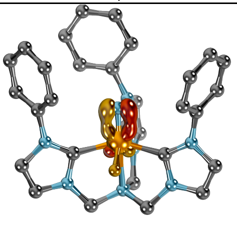
	Fe 0.57, N 0.41	Fe 0.58, N 0.40	Fe 0.59, N 0.40
$\boldsymbol{d_{z^2}}$	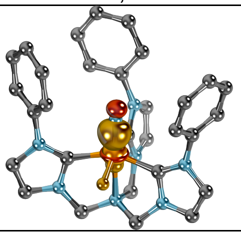	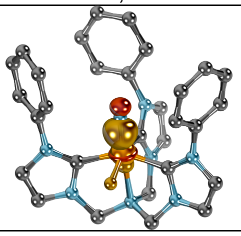	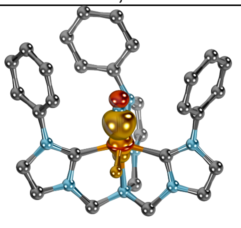
	Fe 0.47, N 0.54	Fe 0.46, N 0.55	Fe 0.45, N 0.57

Supplementary Table 17 | IBOs relating to the five 3*d* orbitals in **2** computed with PBE-D3/def2-TZVPP, TPSSh-D3/def2-TZVPP and PBE0-D3/def2-TZVPP with SMD=MeCN. If a *d*-orbital partakes in several bonds, the MO with the highest contribution of iron is shown. The contribution of atoms is assessed based on the default Mulliken population analysis. The α-orbitals are shown and the populations for the α and β orbitals are noted in the form "α/ β".

	PBE	TPSSh	PBE0
$d_{x^2-y^2}$	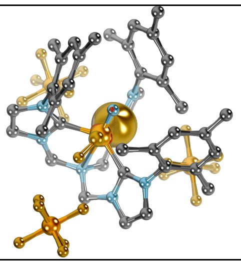<br>Fe 0.39, C 0.65/ Fe 0.37, C 0.68	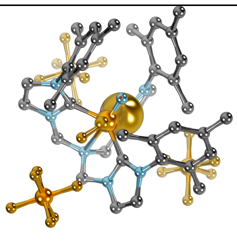<br>Fe 0.35, C 0.68/ Fe 0.34, C 0.70	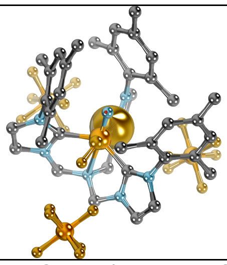<br>Fe 0.35, C 0.69/ Fe 0.33, C 0.73
d_{xy}	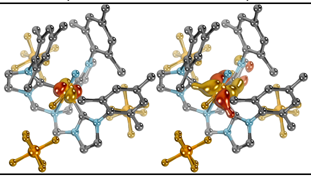<br>Fe 0.93/ Fe 0.66	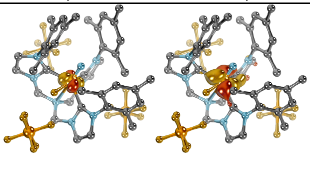<br>Fe 0.95/ Fe 0.77	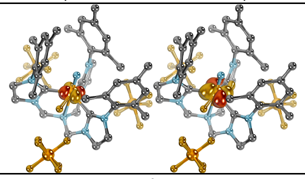<br>Fe 0.95/ Fe 0.85
d_{xz}	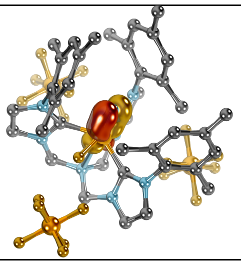<br>Fe 0.62, N 0.34/ Fe 0.55, N 0.40	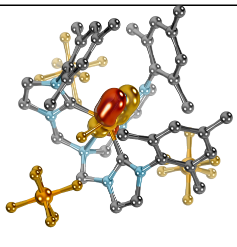<br>Fe 0.64, N 0.33/ Fe 0.46, N 0.54	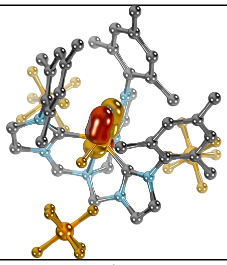<br>Fe 0.71, N 0.25/ Fe 0.39, N 0.59
d_{yz}	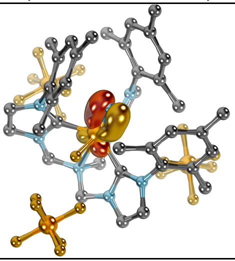<br>Fe 0.57, N 0.39/ Fe 0.52, N 0.46	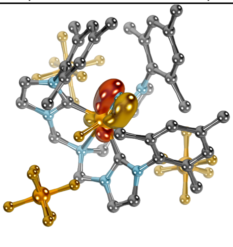<br>Fe 0.58, N 0.39/ Fe 0.51, N 0.48	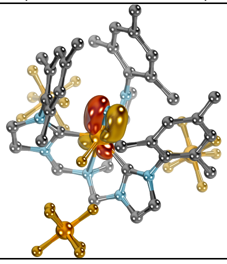<br>Fe 0.63, N 0.33/ Fe 0.45, N 0.57
d_{z^2}	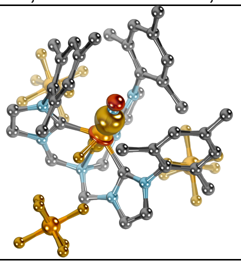<br>Fe 0.51, N 0.50/ Fe 0.48, N 0.53	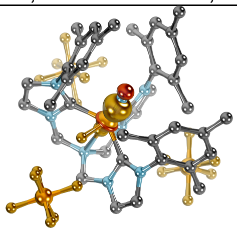<br>Fe 0.50, N 0.51/ Fe 0.54, N 0.42	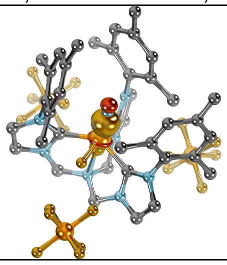<br>Fe 0.51, N 0.51/ Fe 0.45, N 0.57

Supplementary Table 18 | Number of Fe-centered and "covalent" valence electrons for **I** (cutoff Fe-centered: 0.7; cutoff "covalent": 0.3).

CASSCF(9,8), NOs		IBOs(PBE)		IBOs(TPSSh)		IBOs(PBE0)	
Fe-centered	covalent	Fe-centered	covalent	Fe-centered	covalent	Fe-centered	covalent
3	6	3	6	3	6	3	6

Supplementary Table 19 | Number of Fe-centered and covalent valence electrons for **1** (cutoff Fe-centered: 0.7; cutoff "covalent": 0.3).

CASSCF(10,9), NOs		IBOs(PBE)		IBOs(TPSSh)		IBOs(PBE0)	
Fe-centered	covalent	Fe-centered	covalent	Fe-centered	covalent	Fe-centered	covalent
2	8	2	8	2	8	2	8

Supplementary Table 20 | Number of Fe-centered and covalent valence electrons for **2** (cutoff Fe-centered: 0.7; cutoff "covalent": 0.3).

CASSCF(9,9), NOs		IBOs(PBE)		IBOs(TPSSh)		IBOs(PBE0)	
Fe-centered	covalent	Fe-centered	covalent	Fe-centered	covalent	Fe-centered	covalent
1	8	1	9	2	8	3	7

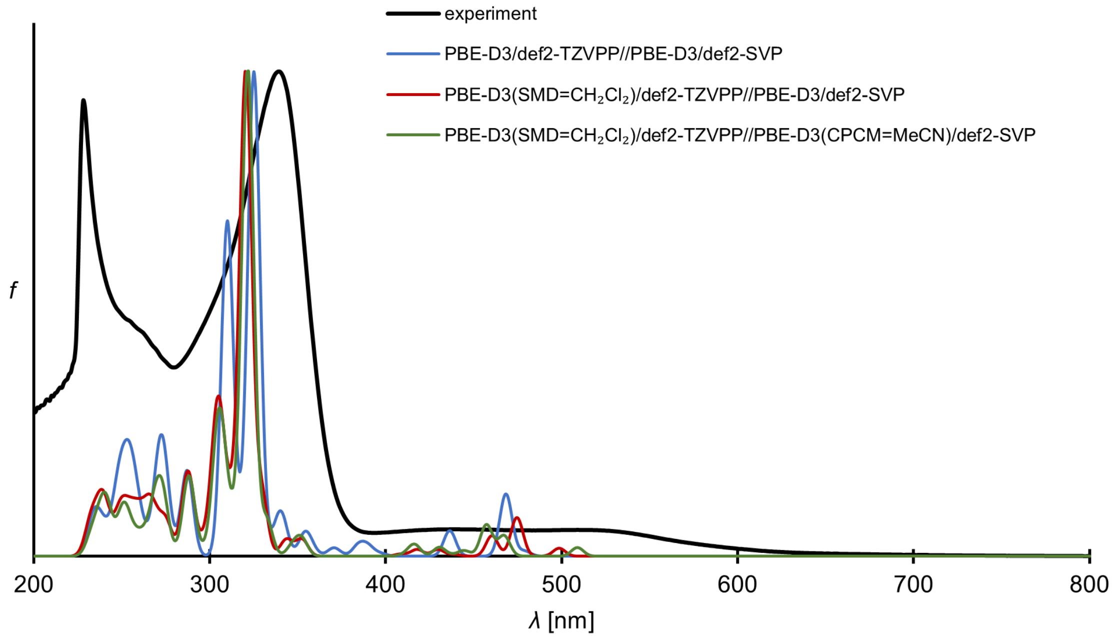

Supplementary Figure 39 | Comparison of TD-DFT absorption spectra of **I'** compared to the experimental spectrum in the experimental range of 200–800 nm. All spectra are scaled to the same maximum intensity. Gaussian broadening with a band width of 8 nm at 1/2 height. There is no notable difference when using implicit solvent models for structure optimization or TD-DFT computation.

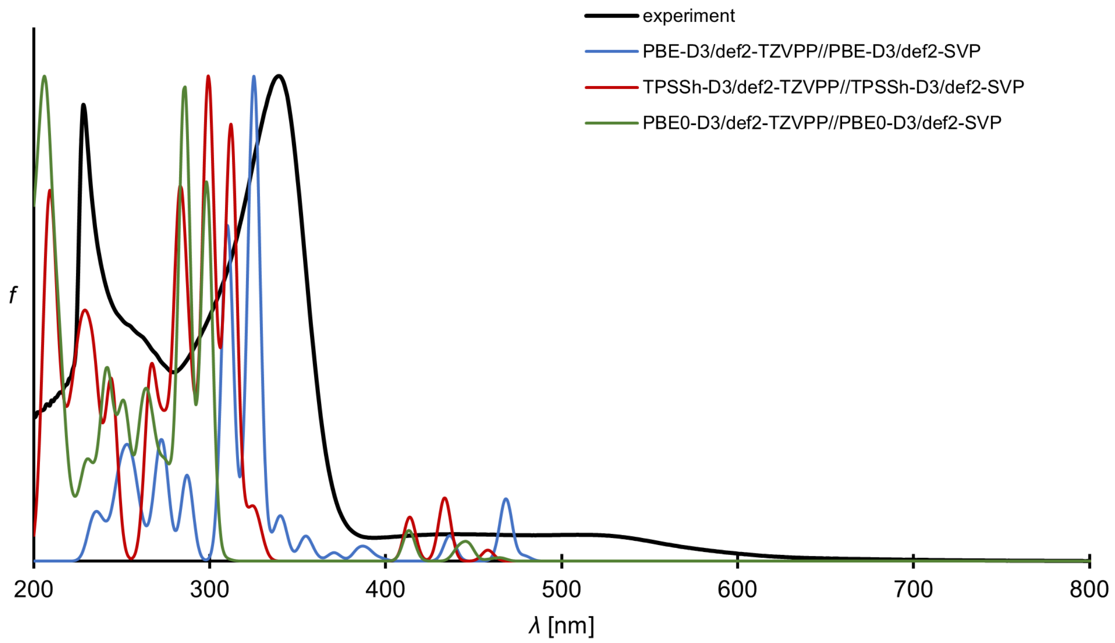

Supplementary Figure 40 | TD-DFT absorption spectra of **I'** compared to the experimental spectrum in the experimental range of 200–800 nm. All spectra are scaled to the same maximum intensity. Gaussian broadening with a band width of 8 nm at 1/2 height.

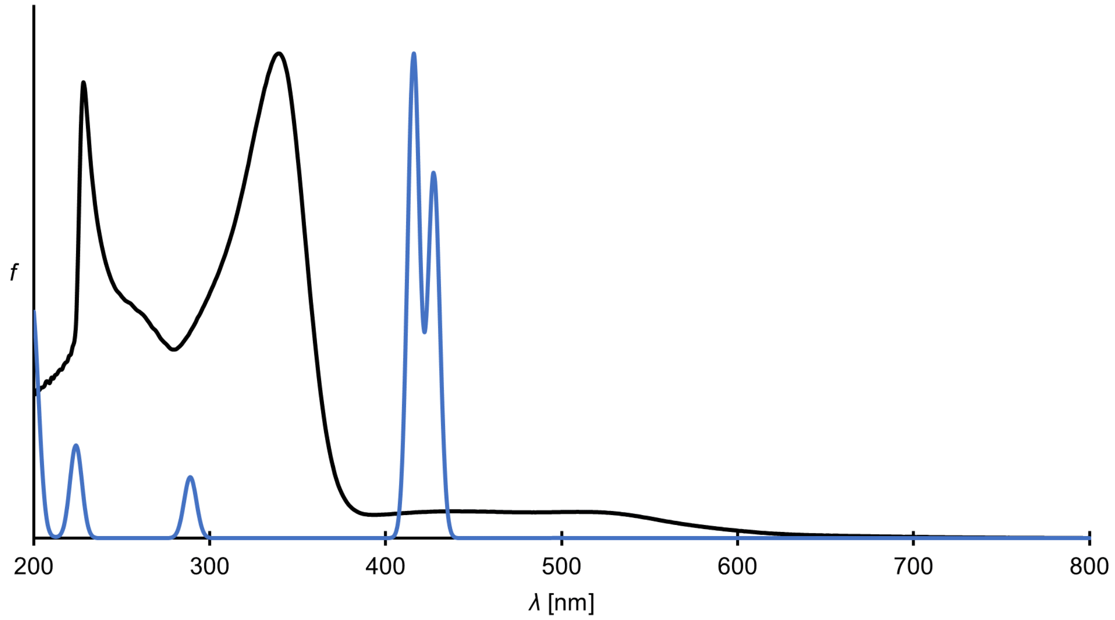

Supplementary Figure 41 | NEVPT2 absorption spectrum (5 quintets, 45 triplets, 50 singlets; blue) based on CASSCF(10,8) $S = 0$ ground state of **I'** compared to the experimental spectrum (black). All spectra are scaled to the same maximum intensity. Gaussian broadening with a band width of 8 nm at 1/2 height.

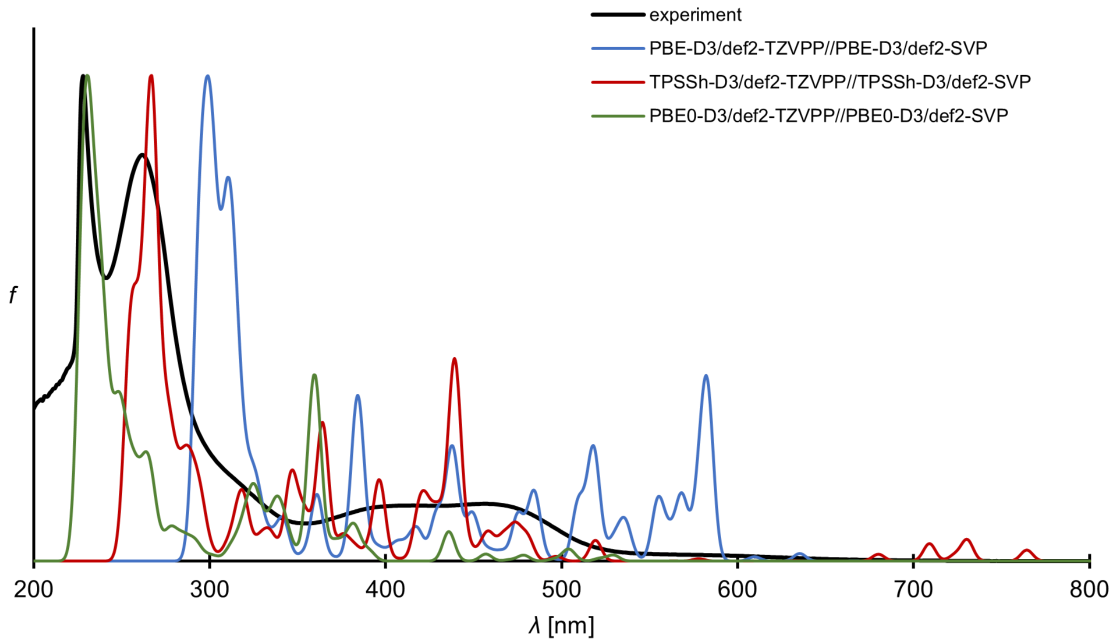

Supplementary Figure 42 | TD-DFT absorption spectra of **I** compared to the experimental spectrum in the experimental range of 200–800 nm. All spectra are scaled to the same maximum intensity. Gaussian broadening with a band width of 8 nm at 1/2 height.

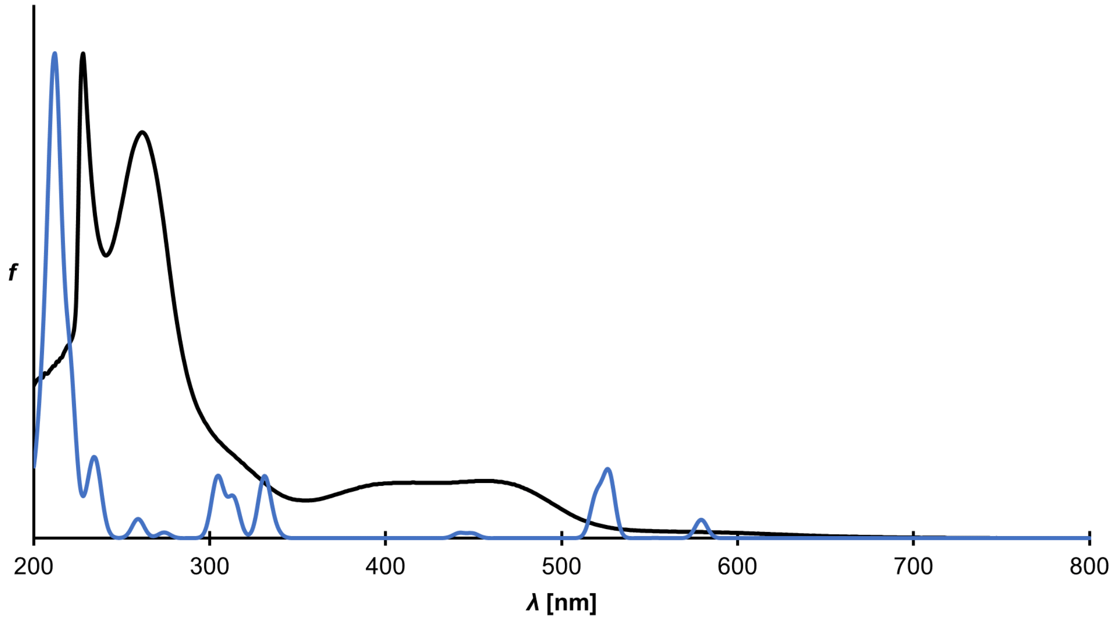

Supplementary Figure 43 | NEVPT2 absorption spectrum (1 sextet, 24 quartets, 75 doublets; blue) based on CASSCF(9,8) $S = 1/2$ ground state of **I** compared to the experimental spectrum (black). All spectra are scaled to the same maximum intensity. Gaussian broadening with a band width of 8 nm at 1/2 height.

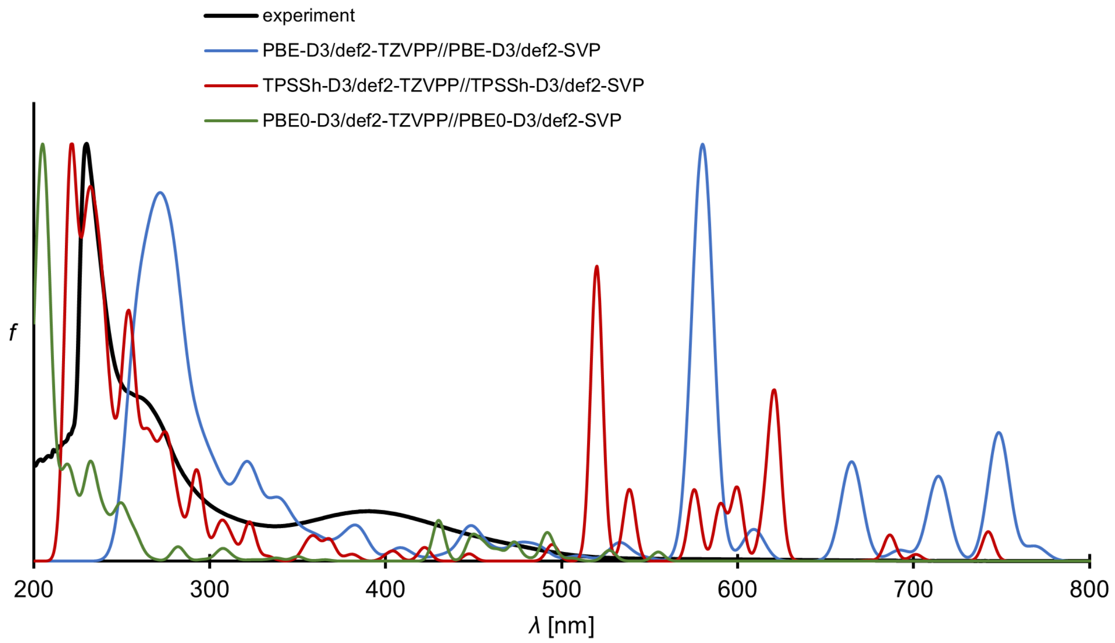

Supplementary Figure 44 | TD-DFT absorption spectra of **1** compared to the experimental spectrum in the experimental range of 200–800 nm. All spectra are scaled to the same maximum intensity. Gaussian broadening with a band width of 8 nm at 1/2 height.

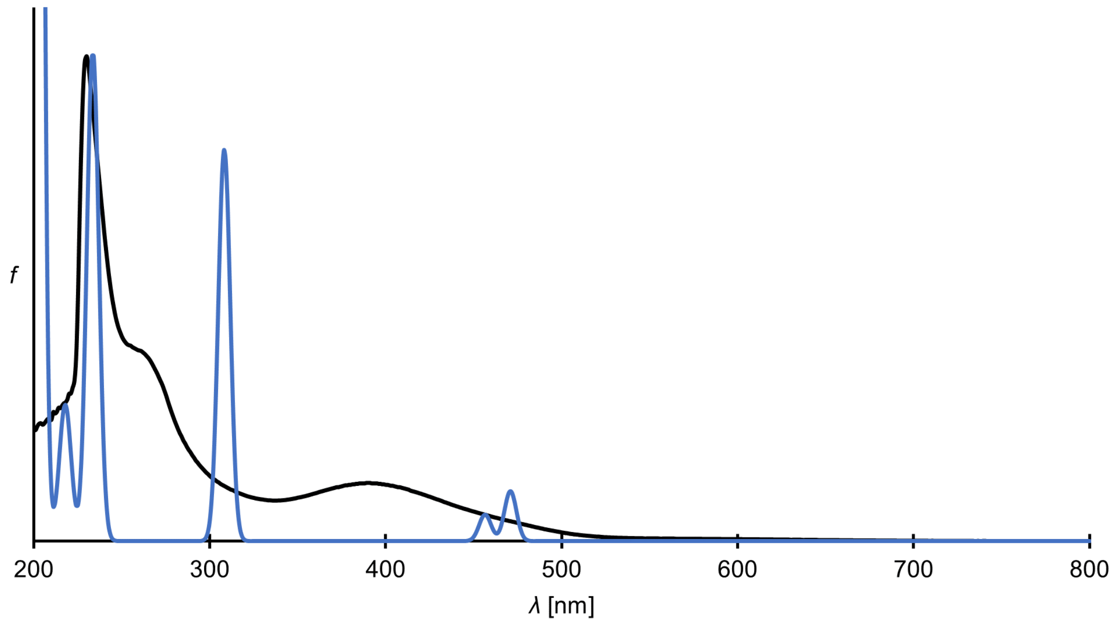

Supplementary Figure 45 | NEVPT2 absorption spectrum (5 quintets, 45 triplets, 50 singlets) based on CASSCF(10,9) $S = 0$ ground state of **1** compared to the experimental spectrum in the experimental range of 200 – 800 nm. All spectra are scaled to the same maximum intensity. Gaussian broadening with a band width of 8 nm at 1/2 height.

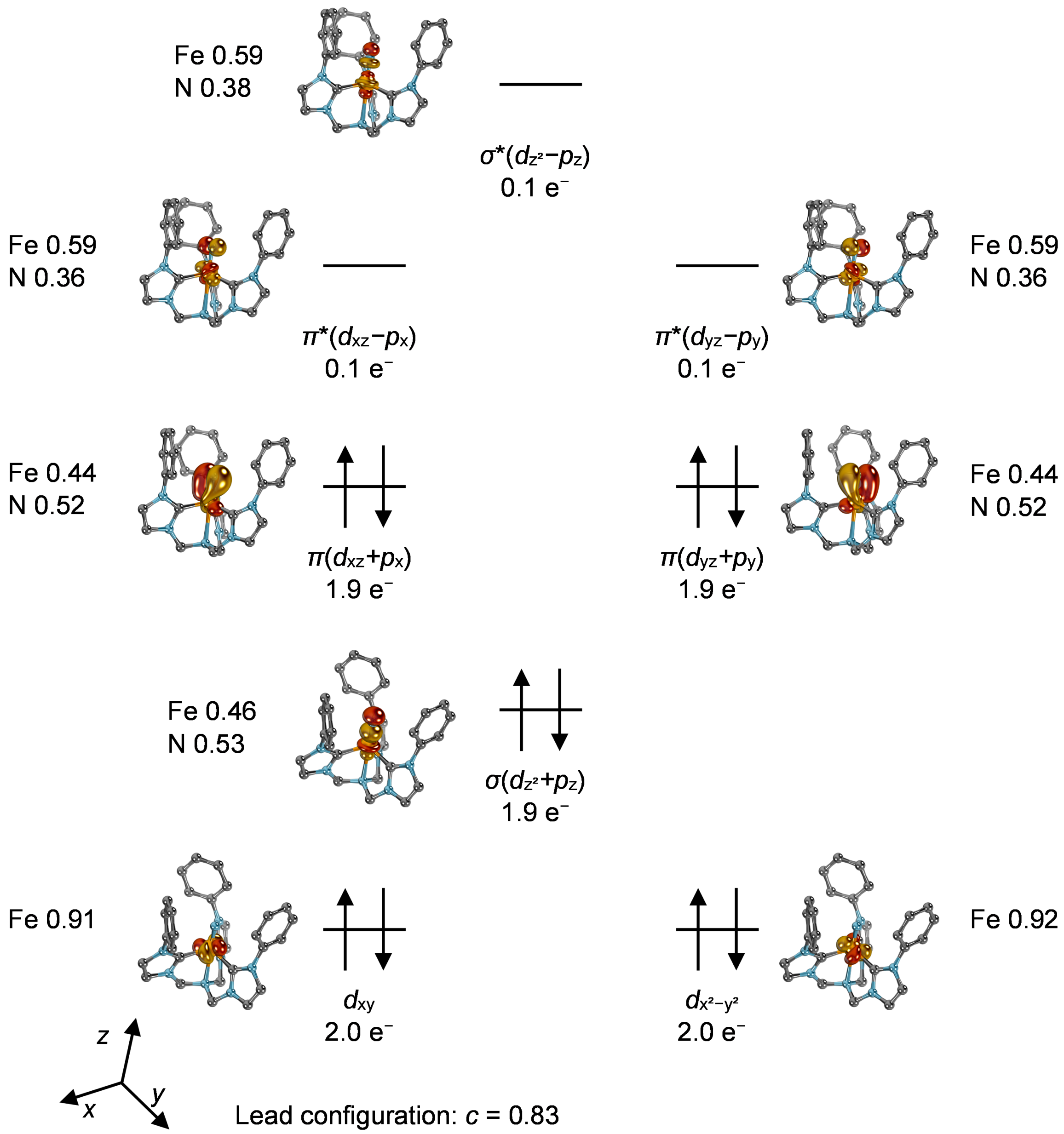

Supplementary Figure 46 | Active space (*top*) and configurations (hereafter) of **I'** for CASSCF(10,8). Orbitals ordered by occupancy.

0.83249 [783]: 22222000	0.02238 [754]: 22202020
0.02395 [733]: 22121101	0.01604 [682]: 22022002
0.02382 [769]: 22211110	0.00319 [708]: 22110211
0.02369 [718]: 22112011	0.00311 [693]: 22101121
0.02293 [779]: 22220200	0.00297 [657]: 22011112

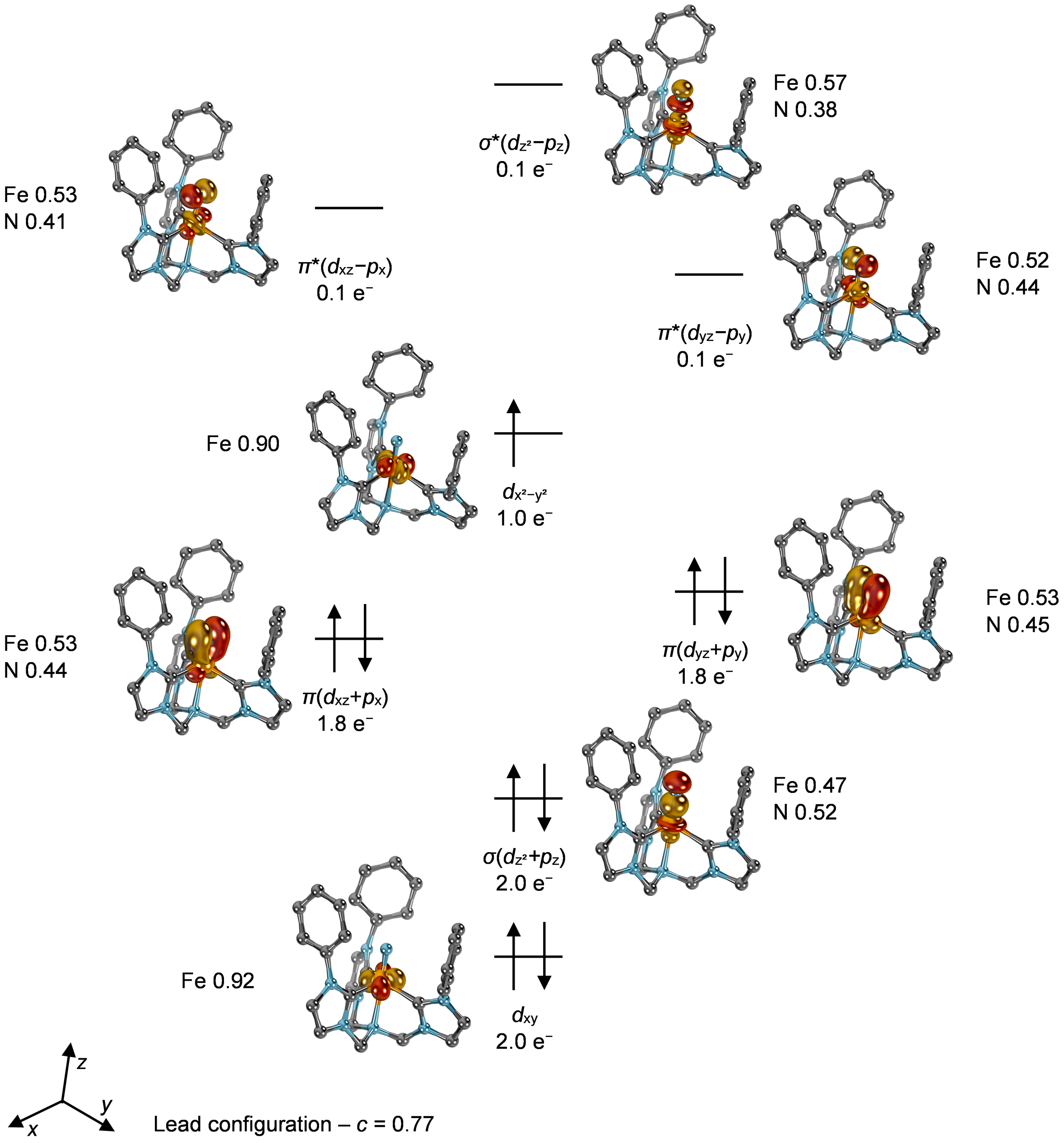

Supplementary Figure 47 | Active space (*top*) and configurations (hereafter) of **I** for CASSCF(9,8). Orbitals ordered by occupancy.

0.77472 [1015]: 22221000
0.03280 [998]: 22201200
0.02810 [934]: 22021020
0.02700 [971]: 22111110
0.02421 [874]: 21211101
0.02256 [837]: 21121011
0.01565 [740]: 20221002
0.01131 [1010]: 22211100

0.00782 [983]: 22121010
0.00590 [886]: 21221001
0.00464 [801]: 21101211
0.00419 [764]: 21011121
0.00383 [898]: 22001220
0.00301 [667]: 20111112
0.00261 [704]: 20201202

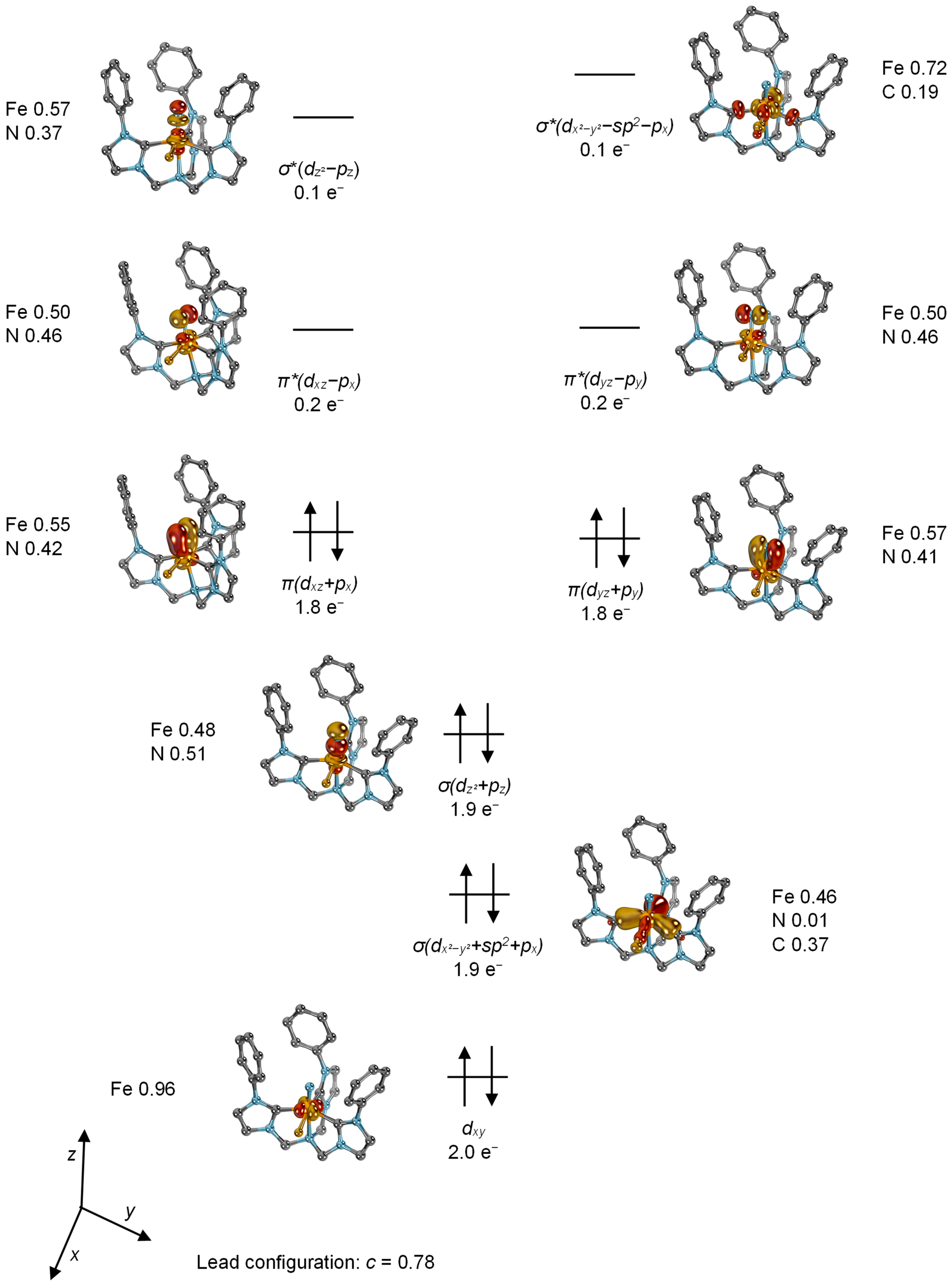

Supplementary Figure 48 | Active space (*top*) and configurations (hereafter) of **1** for CASSCF(10,9). Orbitals ordered by occupancy.

0.78128 [2906]: 222220000
0.03325 [2901]: 222202000
0.02907 [2857]: 222020200
0.02779 [2886]: 222111100
0.02239 [2810]: 221211010
0.02162 [2781]: 221120110
0.01329 [2683]: 220220020
0.01058 [2147]: 202220002

0.00579 [2542]: 212211001
0.00578 [2513]: 212120101
0.00453 [2759]: 221102110
0.00411 [2719]: 221011210
0.00408 [2835]: 222002200
0.00354 [2415]: 211220011
0.00264 [2621]: 220111120

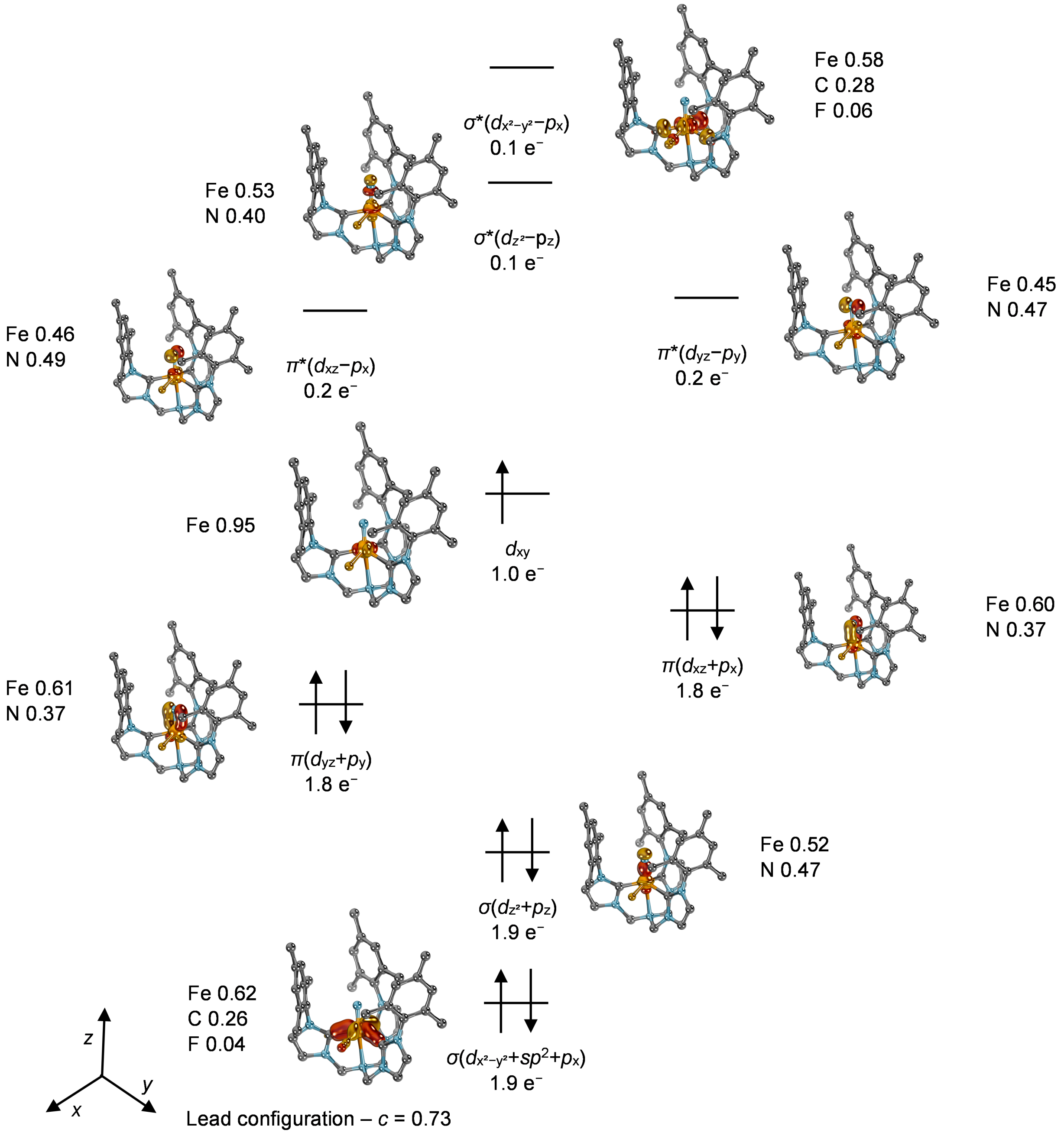

Supplementary Figure 49 | Active space (*top*) and configurations (hereafter) of **2** for CASSCF(9,9). Orbitals ordered by occupancy.

0.72774 [0]: 222210000
0.03007 [24]: 222012000
0.02436 [148]: 220210200
0.02407 [70]: 221111100
0.02183 [2136]: 022210002
0.02165 [287]: 212111010
0.01985 [365]: 211210110
0.01848 [6]: 222111000
0.01483 [52]: 221210100
0.01441 [634]: 202210020

0.00810 [269]: 212210010
0.00704 [1116]: 121210101
0.00682 [1038]: 122111001
0.00473 [1385]: 112210011
0.00466 [1020]: 122210001
0.00382 [448]: 211012110
0.00333 [549]: 210111210
0.00301 [231]: 220012200
0.00260 [818]: 201111120
0.00251 [717]: 202012020

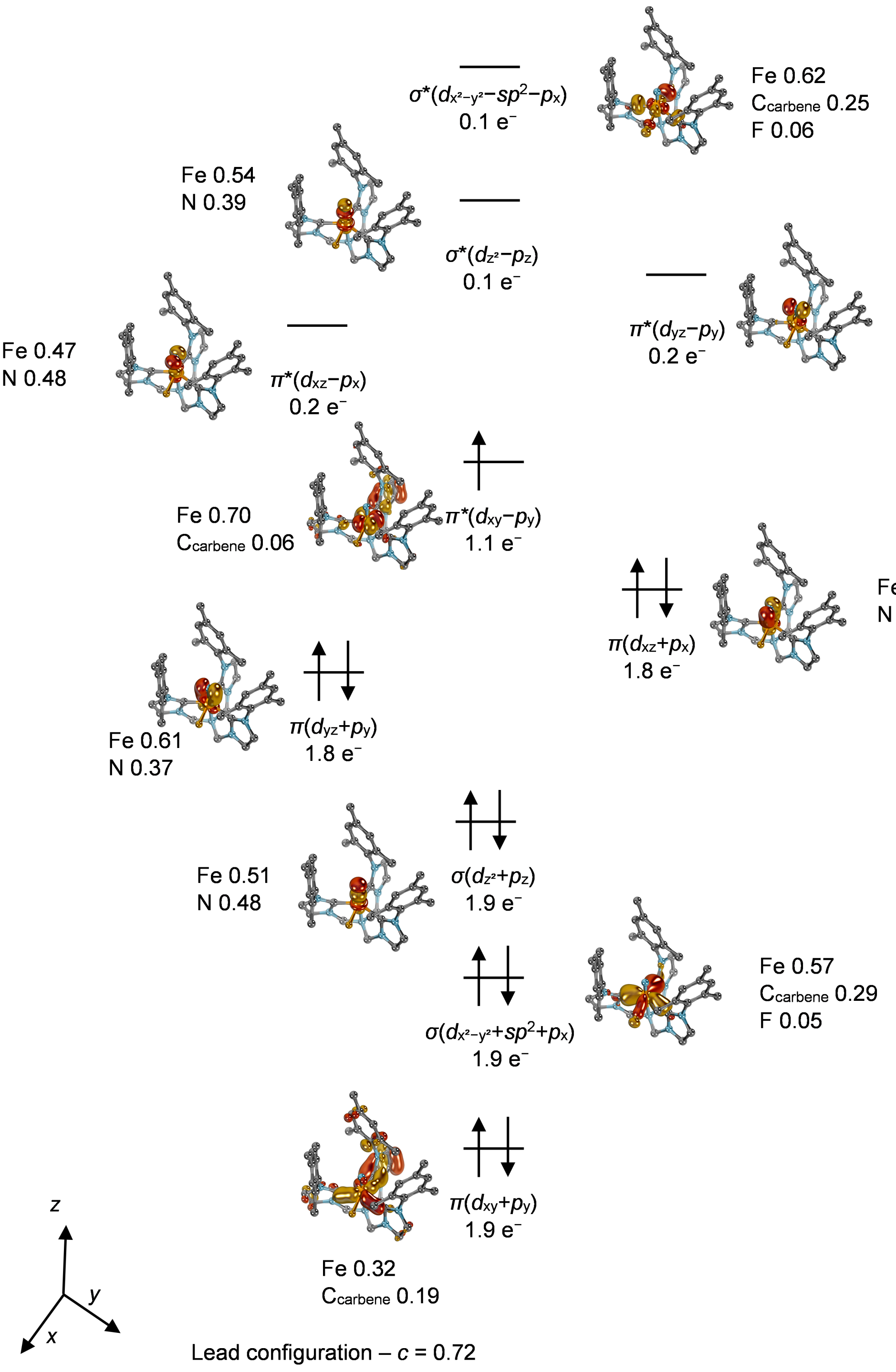

Supplementary Figure 50 | Active space (*top*) and configurations (hereafter) of **2** for CASSCF(11,10). Orbitals ordered by occupancy.

0.72370 [8349]: 2222210000	0.00741 [8297]: 2221210100
0.02750 [8325]: 2222012000	0.00528 [7311]: 2122111001
0.02260 [4100]: 1122220001	0.00521 [7233]: 2121210101
0.02256 [8201]: 2220210200	0.00454 [5195]: 1222121000
0.02166 [8279]: 2221111100	0.00426 [8080]: 2212210010
0.02051 [8062]: 2212111010	0.00400 [5119]: 1221220100
0.01865 [7984]: 2211210110	0.00368 [6964]: 2112210011
0.01396 [6213]: 2022210002	0.00340 [7901]: 2211012110
0.01378 [7715]: 2202210020	0.00298 [7800]: 2210111210
0.00929 [8343]: 2222111000	0.00271 [8118]: 2220012200

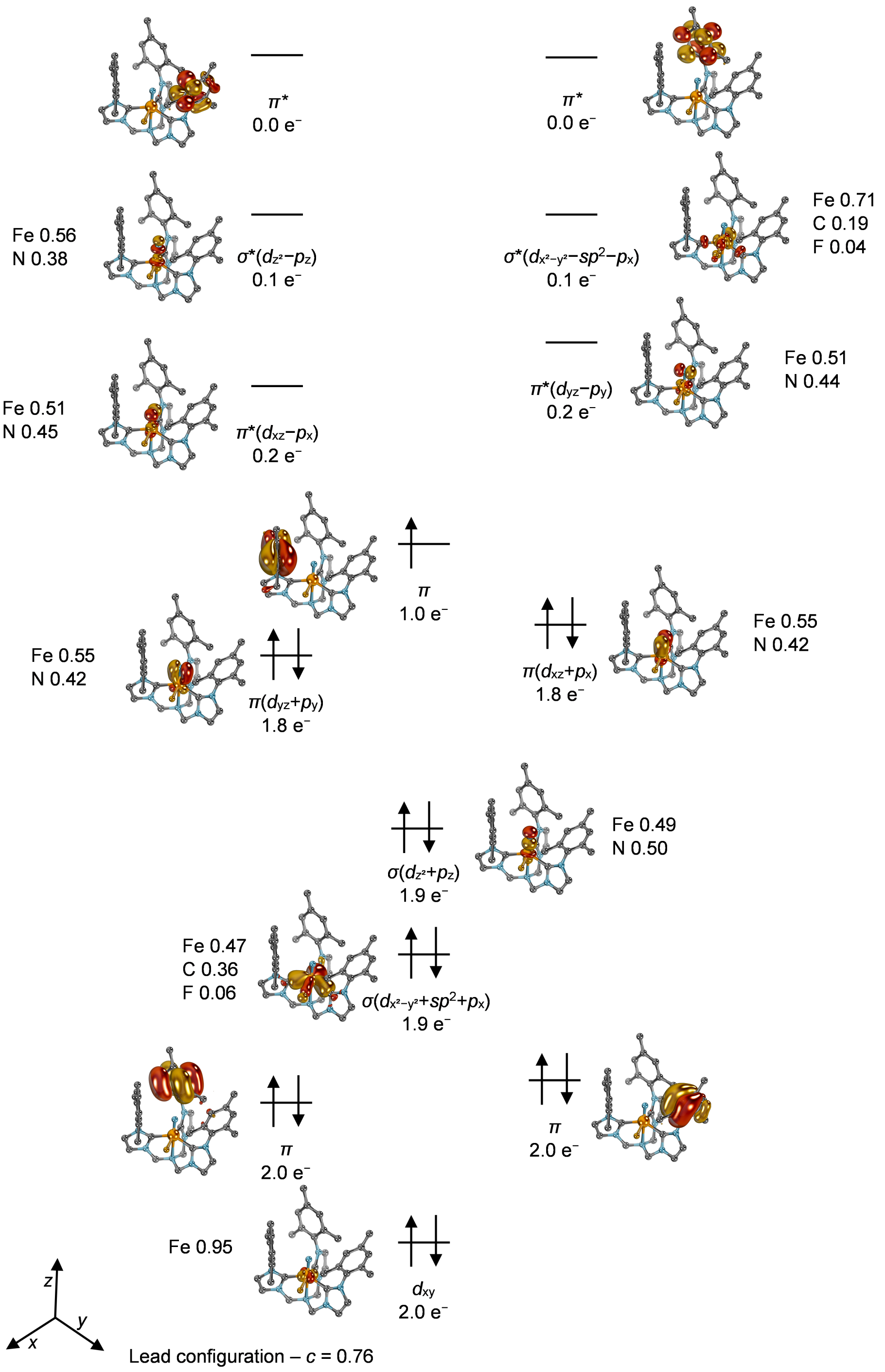

Supplementary Figure 51 | Active space (*top*) and configurations (hereafter) of **2** for CASSCF(15,14). Orbitals ordered by occupancy.

0.75665 [585689]: 22222221000000
0.02976 [585648]: 22222201200000
0.02514 [585299]: 22222021020000
0.02509 [585542]: 22222111110000
0.02146 [584771]: 22221211101000
0.02061 [584528]: 22221121011000
0.01532 [541742]: 22022221000020
0.01409 [442298]: 20222221000002
0.01320 [583236]: 22220221002000

0.01119 [574163]: 22202221000200
0.00566 [580912]: 22212211100100
0.00539 [580669]: 22212121010100
0.00407 [584252]: 22221101211000
0.00361 [583811]: 22221011121000
0.00340 [579377]: 22211221001100
0.00335 [585023]: 22222001220000
0.00255 [582519]: 22220111112000

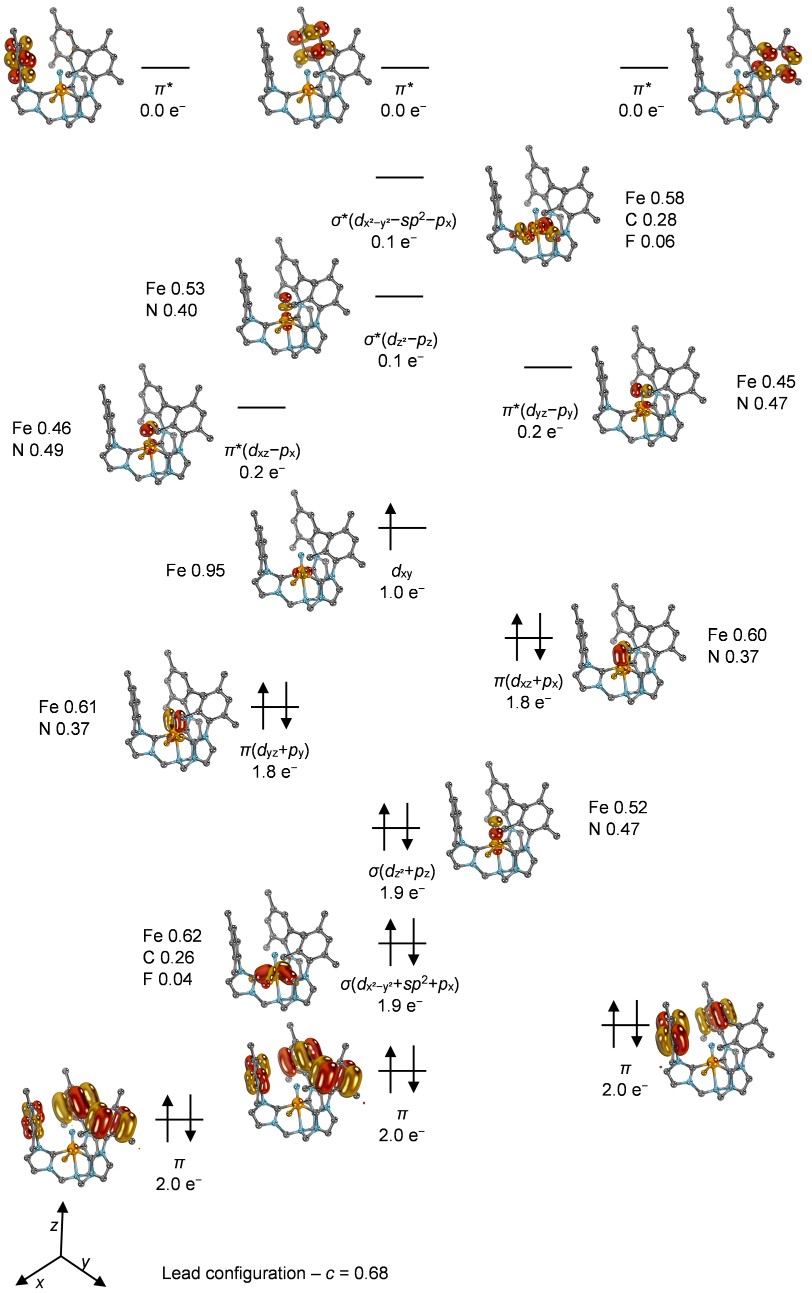

Supplementary Figure 52 | Active space (*top*) and configurations (hereafter) of **2** for CASSCF(15,15), including mesityl π-electrons. Orbitals ordered by occupancy.

0.68366 [0]: 222222210000000
0.02813 [51]: 222222012000000
0.02286 [584]: 222220210200000
0.02254 [200]: 222221111100000
0.02028 [1497]: 222212111010000
0.02025 [23422]: 222022210002000
0.01861 [1881]: 222211210110000
0.01724 [9]: 222222111000000
0.01382 [158]: 222221210100000
0.01351 [4322]: 222202210020000
0.00754 [1455]: 222212210010000
0.00711 [101559]: 220222210000200
0.00679 [787367]: 112222210000002
0.00660 [9472]: 222121210101000
0.00643 [9088]: 222122111001000
0.00444 [11913]: 222112210011000
0.00435 [9046]: 222122210001000
0.00423 [372783]: 202222210000002
0.00356 [2322]: 222211012110000
0.00323 [787365]: 112222210000020
0.00311 [3112]: 222210111210000
0.00281 [1025]: 222220012200000
0.00274 [644000]: 121222210000020
0.00273 [1201951]: 022222210000002
0.00267 [229413]: 211222210000200

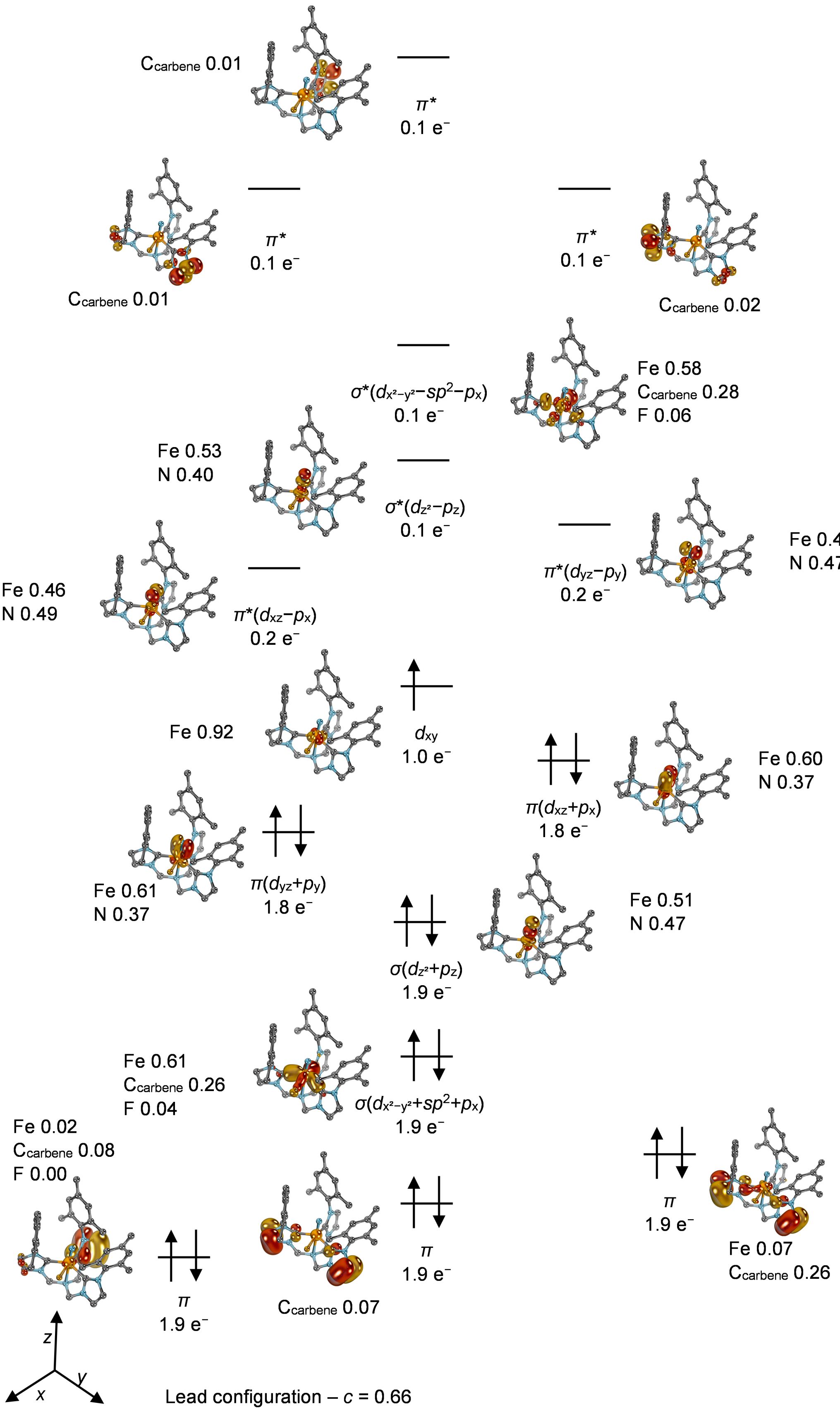

Supplementary Figure 53 | Active space (*top*) and configurations (hereafter) of **2** for CASSCF(15,15), including NHC π-electrons. Orbitals ordered by occupancy.

0.66233 [0]: 222222210000000
0.02706 [51]: 222222012000000
0.02205 [584]: 222220210200000
0.02171 [200]: 222221111100000
0.01959 [1497]: 222212111010000
0.01884 [23422]: 222022210002000
0.01867 [1201951]: 022222210000002
0.01798 [1881]: 222211210110000
0.01580 [9]: 222222111000000
0.01306 [4322]: 222202210020000
0.01273 [158]: 222221210100000
0.00731 [229414]: 211222210000110
0.00695 [1455]: 222212210010000
0.00659 [229413]: 211222210000200
0.00654 [372778]: 202222210000200
0.00614 [9472]: 222121210101000
0.00611 [229416]: 211222210000020
0.00600 [9088]: 222122111001000
0.00562 [101562]: 220222210000020
0.00427 [372781]: 202222210000020
0.00415 [11913]: 222112210011000
0.00407 [101559]: 220222210000200
0.00402 [9046]: 222122210001000
0.00342 [2322]: 222211012110000
0.00299 [3112]: 222210111210000
0.00269 [1025]: 222220012200000

Supplementary Table 21 | Single point energies of active spaces for **2** in gas phase.

	CASSCF(9,9)	CASSCF(11,10)	CASSCF(15,15) NHC	CASSCF(15,14) Mesityl	CASSCF(15,15) Mesityl
***E* [Eh]**	−3315.79870	−3315.80977	−3315.86431	−3315.91365	−3315.83161
	**NEVPT2/ CASSCF(9,9)**	**NEVPT2/ CASSCF(11,10)**	**NEVPT2/ CASSCF(15,15) NHC**	**NEVPT2/ CASSCF(15,14) Mesityl**	**NEVPT2/ CASSCF(15,15) Mesityl**
***E* [Eh]**	−3325.645107	−3325.66854	-3325.62985	−3325.68620	-3325.65527

Supplementary Table 22 | Single point energies of active spaces for **2** with correction for implicit solvation in acetonitrile.

	CASSCF(9,9)	CASSCF(11,10)	CASSCF(15,15) NHC	CASSCF(15,14) Mesityl	CASSCF(15,15) Mesityl
***E* [Eh]**	-3316.28856	-3316.29984	-3316.35405	-3316.38590	-3316.32088
	**NEVPT2/ CASSCF(9,9)**	**NEVPT2/ CASSCF(11,10)**	**NEVPT2/ CASSCF(15,15) NHC**	**NEVPT2/ CASSCF(15,14) Mesityl**	**NEVPT2/ CASSCF(15,15) Mesityl**
***E* [Eh]**	-3326.10831	-3326.12577	-3326.09311	-3326.13696	-3326.11857

Supplementary Table 23 | Energies and occupancies of CASSCF(9,9) for **2** using structures using different optimization routines.

	PBE-D3/def2-SVP		PBE-D3(CPCM=MeCN)/def2-SVP		TPSSh-D3/def2-SVP	
**MO**	***E* [Eh]**	**Occupancy**	***E* [Eh]**	**Occupancy**	***E* [Eh]**	**Occupancy**
$\sigma(d_{x^2-y^2}+sp^2+p_x)$	−0.9659	1.90	−0.9637	1.90	−0.9653	1.90
$\sigma(d_{z^2}+p_z)$	−0.9974	1.87	−0.9943	1.87	−1.0051	1.87
$\pi(d_{yz}+p_y)$	−0.9461	1.83	−0.9442	1.83	−0.9538	1.94
$\pi(d_{xz}+p_x)$	−0.9319	1.81	−0.9307	1.81	−0.9394	1.82
d_{xy}	−0.6731	1.00	−0.6707	1.00	−0.6736	1.00
$\pi^*(d_{xz}-p_x)$	−0.3679	0.19	−0.3661	0.19	−0.3591	0.18
$\pi^*(d_{yz}-p_y)$	−0.3543	0.17	−0.3522	0.17	−0.3461	0.16
$\sigma^*(d_{z^2}-p_z)$	−0.2220	0.13	−0.2214	0.13	−0.2097	0.13
$\sigma^*(d_{x^2-y^2}-sp^2-p_x)$	−0.2796	0.10	−0.2724	0.10	−0.2795	0.10

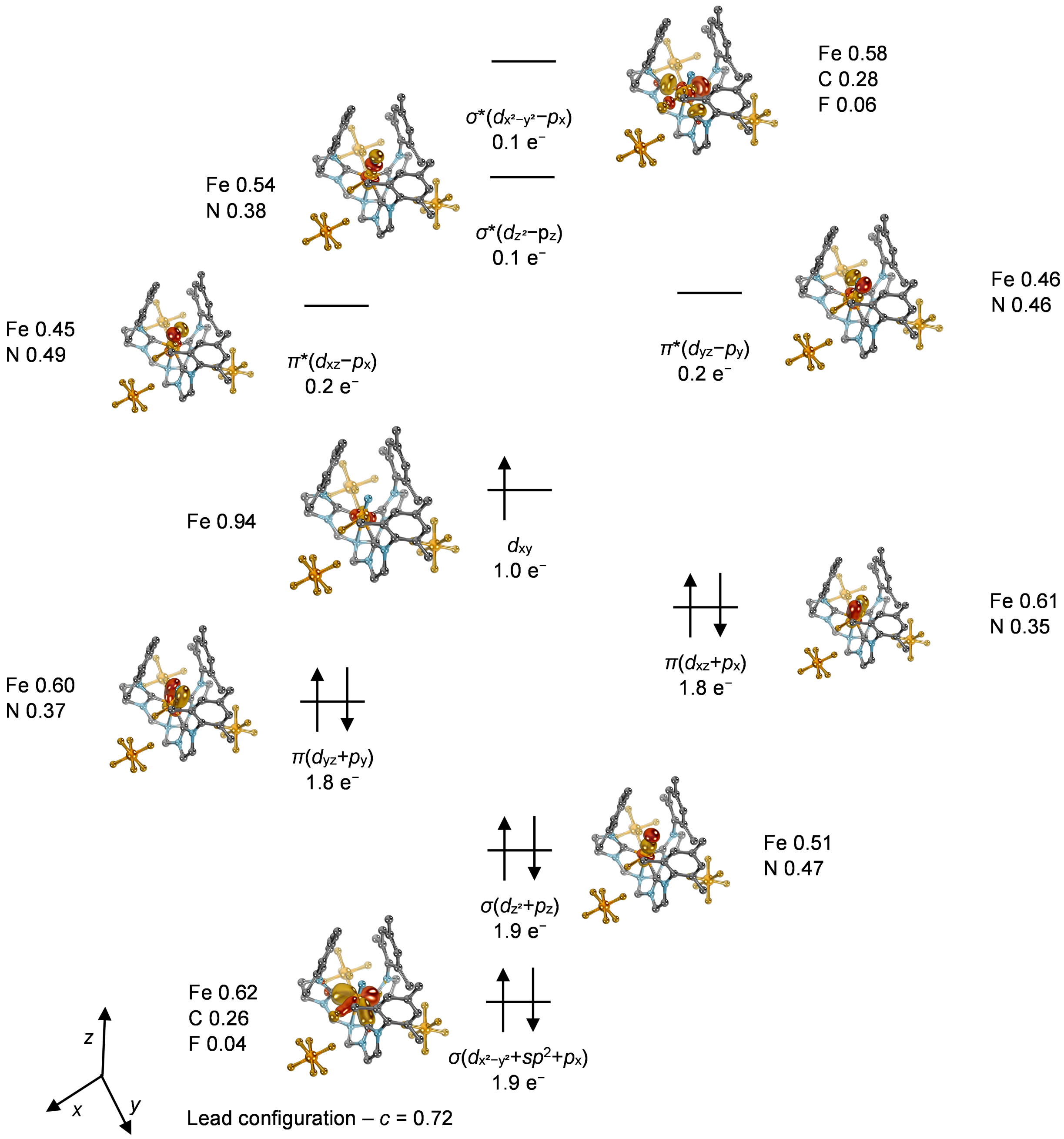

Supplementary Figure 54 | Active space (*top*) and configurations (hereafter) of **2·3PF$_6$** for CASSCF(9,9); SMD=MeCN. Orbitals ordered by occupancy.

0.71955 [	0]:	222210000			
0.03039 [	24]:	222012000	0.00832 [	269]:	212210010
0.02624 [	148]:	220210200	0.00715 [	1038]:	122111001
0.02484 [	70]:	221111100	0.00678 [	1116]:	121210101
0.02136 [	2136]:	022210002	0.00487 [	1385]:	112210011
0.02115 [	287]:	212111010	0.00459 [	1020]:	122210001
0.02059 [	365]:	211210110	0.00384 [	448]:	211012110
0.01934 [	6]:	222111000	0.00361 [	549]:	210111210
0.01618 [	52]:	221210100	0.00325 [	231]:	220012200
0.01432 [	634]:	202210020	0.00264 [	818]:	201111120

Supplementary Table 24 | Energies of ground states involved in the computed reaction mechanisms. All optimizations *via* PBE-D3/def2-SVP. For implicit solvation models, the solvent is given in parenthesis.

Name	*E*(PBE-D3/def2-SVP) [Eh]	*G*(PBE-D3/def2-SVP) [Eh]	*E*(PBE-D3/def2-TZVPP) [Eh]	*E*(PBE-D3/def2-TZVPP, CPCM) [Eh]	*E*(TPSSh-D3/def2-TZVPP) [Eh]	*E*(TPSSh-D3/def2-TZVPP, CPCM) [Eh]	*E*(PBE0-D3/def2-TZVPP) [Eh]	*E*(PBE0-D3/def2-TZVPP, CPCM) [Eh]	*E*(ωB97X-D4/def2-TZVPP) [Eh]	*E*(ωB97X-D4/def2-TZVPP, CPCM) [Eh]
**HF**	−100.21302	−100.22028	−100.53842	−100.54609 (MeCN)	−100.62774	−100.63561 (MeCN)	−100.53833	−100.54636 (MeCN)	−100.64207	−100.65009 (MeCN)
**MeCN**	−132.59184	−132.57069	−132.74131	−132.75183 (MeCN)	−132.91531	−132.92592 (MeCN)	−132.74473	−132.75565 (MeCN)	−132.95694	−132.96789 (MeCN)
**2**	−3325.41611	−3324.73056	−3327.72011	−3328.12532 (SO_2)	−3330.61871	−3331.02359 (SO_2)	−3327.74432	−3328.15153 (SO_2)	−3331.07856	−3331.52890 (SO_2)
**a**	−3325.41402	−3324.72047	−3327.71356	−3328.13693 (SO_2)	−3330.62323	−3331.04777 (SO_2)	−3327.76400	−3328.19101 (SO_2)	−3331.17145	−3331.60236 (SO_2)
**b (d)**	−3325.46066	−3324.77126	−3327.75957	−3328.18319 (SO_2)	−3330.66932	−3331.09373 (SO_2)	−3327.81457	−3328.24155 (SO_2)	−3331.21378	−3331.64511 (SO_2)
**b (q)**	−3325.46674	−3324.77596	−3327.76263	−3328.18991 (SO_2)	−3330.67877	−3331.10596 (SO_2)	−3327.82647	−3328.25532 (SO_2)	−3331.22630	−3331.65764 (SO_2)
**c (d)**	−3325.48357	−3324.79285	−3327.78276	−3328.20238 (SO_2)	−3330.69467	−3331.11419 (SO_2)	−3327.84221	−3328.26312 (SO_2)	−3331.24783	−3331.67060 (SO_2)
**c (q)**	−3325.46105	−3324.77223	−3327.75691	−3328.18358 (SO_2)	−3330.67647	−3331.10331 (SO_2)	−3327.82854	−3328.25682 (SO_2)	−3331.23325	−3331.66290 (SO_2)
**d**	−3325.45881	−3324.76734	−3327.75884	−3328.17729 (SO_2)	−3330.67115	−3331.08983 (SO_2)	−3327.81581	−3328.23594 (SO_2)	−3331.21877	−3331.64113 (SO_2)
**e**	−3325.51123	−3324.816256	−3327.80575	−3328.23381 (SO_2) −3328.24251 (MeCN)	−3330.72331	−3331.15140 (SO_2) −3331.16009 (MeCN)	−3327.87362	−3328.30406 (SO2) −3328.31280 (MeCN)	−3331.27498	−3331.70819 (SO_2) −3331.71698 (MeCN)
**f**	−3325.49875	−3324.80552	−3327.79834	−3328.23384 (MeCN)	−3330.70974	−3331.14449 (MeCN)	−3327.85937	−3328.29546 (MeCN)	−3331.25857	−3331.69660 (MeCN)
**3**	−3357.89515	−3357.17288	−3360.04092	−3360.46716 (MeCN)	−3363.04188	−3363.46714 (MeCN)	−3360.11038	−3360.53712 (MeCN)	−3363.62532	−3364.05364 (MeCN)

Supplementary Table 25 | Energies of transition states involved in the computed reaction mechanisms. All optimizations *via* PBE-D3/def2-SVP. For implicit solvent models, the solvent is given in parenthesis.

Name	*Imag.* [cm^{-1}]	*E*(PBE-D3/def2-SVP) [Eh]	*G*(PBE-D3/def2-SVP) [Eh]	*E*(PBE-D3/def2-TZVPP) [Eh]	*E*(PBE-D3/def2-TZVPP, CPCM) [Eh]	*E*(TPSSh-D3/def2-TZVPP) [Eh]	*E*(TPSSh-D3/def2-TZVPP, CPCM) [Eh]	*E*(PBE0-D3/def2-TZVPP) [Eh]	*E*(PBE0-D3/def2-TZVPP, CPCM) [Eh]	*E*(ωB97X-D4/def2-TZVPP) [Eh]	*E*(ωB97X-D4/def2-TZVPP, CPCM) [Eh]
**[TS**-2a**]**	*i*414	−3325.39838	−3324.70721	−3327.69311	−3328.11560 (SO_2)	−3330.59869	−3331.01650 (SO_2)	−3327.72748	−3328.14975 (SO_2)	−3331.12357	−3331.55138 (SO_2)
**[TS**-ab**]**	*i*431	−3325.39756	−3324.70707	−3327.69880	−3328.12151 (SO_2)	−3330.60844	−3331.03190 (SO_2)	−3327.75030	−3328.17564 (SO_2)	−3331.15314	−3331.58176 (SO_2)
**[TS**-bc**] (q)**	*i*1205	−3325.45433	−3324.76778	−3327.74848	−3328.17482 (SO_2)	−3330.66187	−3331.08766 (SO_2)	−3327.81210	−3328.23913 (SO_2)	−3331.20730	−3331.63519 (SO_2)
**[TS**-cd**]**	*i*690	−3325.45047	−3324.76189	−3327.75049	−3328.17202 (SO_2)	−3330.66197	−3331.08350 (SO_2)	−3327.80987	−3328.23274 (SO_2)	−3331.21342	−3331.63831 (SO_2)
**[TS**-de**]**	*i*207	−3325.45770	−3324.76776	−3327.75585	−3328.17581 (SO_2)	−3330.66589	−3331.08594 (SO_2)	−3327.81135	−3328.23285 (SO_2)	−3331.20893	−3331.63261 (SO_2)
**[TS**-ef**]**	*i*777	−3325.48091	−3324.79333	−3327.77503	−3328.21171 (MeCN)	−3330.68407	−3331.12160 (MeCN)	−3327.83404	−3328.27316 (MeCN)	−3331.23255	−3331.67345 (MeCN)

Supplementary Table 26 | Energies of ground states for optimization at the PBE-D3/def2-SVP, TPSSh-D3/def2-SVP and PBE0-D3/def2-SVP level of theory with and without CPCM=MeCN.

**Optimization method**	**PBE-D3/def2-SVP**					
**Compound**	**I'**	**I**	**1**	**2**	**e**	**3**
***E*(optimization method) [Eh]**	−3226.49958	−3226.41433	−3325.85615	−3325.41611	−3325.51123	−3357.89515
***G*(optimization method) [Eh]**	−3225.81622	−3225.72878	−3325.16387	−3324.73056	−3324.816256	−3357.17288
***E*(PBE-D3/def2-TZVPP) [Eh]**	−3228.51245	−3228.21184	−3328.16145	−3327.72011	−3327.80575	−3360.04092
***E*(PBE-D3(CPCM=MeCN)/def2-TZVPP) [Eh]**	−3228.58514	−3228.42637	−3328.36987	−3328.13352	−3328.24251	−3360.46716
***E*(TPSSh-D3/def2-TZVPP) [Eh]**	−3231.33318	−3231.03700	−3331.06688	−3330.61871	−3330.72331	−3363.04188
***E*(TPSSh-D3(CPCM=MeCN)/def2-TZVPP) [Eh]**	−3231.40647	−3231.25138	−3331.27508	−3331.03178	−3331.16009	−3363.46714
***E*(PBE0-D3/def2-TZVPP) [Eh]**	−3228.56853	−3228.27173	−3328.20204	−3327.74432	−3327.87362	−3360.11038
***E*(PBE0-D3(CPCM=MeCN)/def2-TZVPP) [Eh]**	−3228.64476	−3228.48827	−3328.41240	−3328.15981	−3328.31280	−3360.53712
**Optimization method**	**PBE-D3/def2-SVP(CPCM=MeCN)**					
**Compound**	**I'**	**I**	**1**	**2**	**e**	**3**

***E*(optimization method) [Eh]**	−3226.57380	−3226.41429	−3326.06233	−3325.83762	−3325.94989	−3358.32329
***G*(optimization method) [Eh]**	−3225.89156	−3225.72785	−3325.37045	−3325.14744	−3325.25214	−3357.59875
***E*(PBE-D3/def2-TZVPP) [Eh]**	−3228.51130	−3228.21185	−3328.15726	−3327.71952	−3327.80258	−3360.03907
***E*(PBE-D3(CPCM=MeCN)/def2-TZVPP) [Eh]**	−3228.58720	−3228.42632	−3328.36909	−3328.14368	−3328.24657	−3360.46960
***E*(TPSSh-D3/def2-TZVPP) [Eh]**	−3231.33189	−3231.03699	−3331.06320	−3330.62074	−3330.72177	−3363.04175
***E*(TPSSh-D3(CPCM=MeCN)/def2-TZVPP) [Eh]**	−3231.40869	−3231.25132	−3331.27490	−3331.04554	−3331.16587	−3363.47128
***E*(PBE0-D3/def2-TZVPP) [Eh]**	−3228.56632	−3228.27175	−3328.20000	−3327.74804	−3327.87242	−3360.11049
***E*(PBE0-D3(CPCM=MeCN)/def2-TZVPP) [Eh]**	−3228.64646	−3228.48824	−3328.41402	−3328.17610	−3328.31905	−3360.54164
**Optimization method**	**TPSSh-D3/def2-SVP**					
**Compound**	**I'**	**I**	**1**	**2**	**e**	**3**
***E*(optimization method) [Eh]**	−3229.31942	−3229.23868	−3328.76375	−3328.31559	−3328.43325	−3360.89317
***G*(optimization method) [Eh]**	−3228.61608	−3228.53247	−3328.05087	−3327.61165	−3327.71716	−3360.14800
***E*(PBE-D3/def2-TZVPP) [Eh]**	−3228.51428	−3228.21310	−3328.15921	−3327.72132	−3327.80608	−3360.04229
***E*(PBE-D3(CPCM=MeCN)/def2-TZVPP) [Eh]**	−3228.58693	−3228.42813	−3328.36906	−3328.13619	−3328.24456	−3360.47036
***E*(TPSSh-D3/def2-TZVPP) [Eh]**	−3231.34176	−3231.04542	−3331.07231	−3330.62748	−3330.73176	−3363.05088
***E*(TPSSh-D3(CPCM=MeCN)/def2-TZVPP) [Eh]**	−3231.41496	−3231.26031	−3331.28195	−3331.0421	−3331.17019	−3363.47792
***E*(PBE0-D3/def2-TZVPP) [Eh]**	−3228.57963	−3228.28285	−3328.21187	−3327.75591	−3327.88427	−3360.12142
***E*(PBE0-D3(CPCM=MeCN)/def2-TZVPP) [Eh]**	−3228.65565	−3228.49985	−3328.42363	−3328.17302	−3328.32511	−3360.54994
**Optimization method**	**TPSSh-D3/def2-SVP(CPCM=MeCN)**					
**Compound**	**I'**	**I**	**1**	**2**	**e**	**3**
***E*(optimization method) [Eh]**	−3229.39527	−3229.23863	−3328.97468	−3328.73744	−3328.87428	−3361.32268
***G*(optimization method) [Eh]**	−3228.69169	−3228.53233	−3328.26133	−3328.02837	−3328.15607	−3360.57541
***E*(PBE-D3/def2-TZVPP) [Eh]**	−3228.51314	−3228.21313	−3328.15776	−3327.71600	−3327.80177	−3360.03915
***E*(PBE-D3(CPCM=MeCN)/def2-TZVPP) [Eh]**	−3228.58929	−3228.42809	−3328.37023	−3328.13837	3328.24707	−3363.48014
***E*(TPSSh-D3/def2-TZVPP) [Eh]**	−3231.34039	−3231.04543	−3331.07152	−3330.62390	−3330.72884	−3363.04922
***E*(TPSSh-D3(CPCM=MeCN)/def2-TZVPP) [Eh]**	−3231.41748	−3231.26025	−3331.28384	−3331.04657	−3331.17421	−3363.48014
***E*(PBE0-D3/def2-TZVPP) [Eh]**	−3228.57730	−3228.28288	−3328.21119	−3327.75353	−3327.88191	−3360.11999
***E*(PBE0-D3(CPCM=MeCN)/def2-TZVPP) [Eh]**	−3228.65767	−3228.49982	−3328.42573	−3328.17858	−3328.32977	−3360.55251
**Optimization method**	**PBE0-D3/def2-SVP**					
**Compound**	**I'**	**I**	**1**	**2**	**e**	**3**
***E*(optimization method) [Eh]**	−3226.56799	−3226.27321	−3325.91525	−3325.45517	−3325.59729	−3357.97430
***G*(optimization method) [Eh]**	−3225.85607	−3225.56155	−3325.19537	−3324.74430	−3324.87479	−3357.22261
***E*(PBE-D3/def2-TZVPP) [Eh]**	−3228.51229	−3228.21276	-3328.15649	−3327.71856	−3327.80481	−3360.04142
***E*(PBE-D3(CPCM=MeCN)/def2-TZVPP) [Eh]**	−3228.58426	−3228.42366	−3328.36632	−3328.13448	−3328.24353	−3360.46965
***E*(TPSSh-D3/def2-TZVPP) [Eh]**	−3231.34183	−3231.04689	−3331.07218	−3330.62727	−3330.73226	−3363.05174

***E*(TPSSh-D3(CPCM=MeCN)/def2-TZVPP) [Eh]**	−3231.41431	−3231.25742	−3331.28173	−3331.04319	−3331.17097	−3363.47894
***E*(PBE0-D3/def2-TZVPP) [Eh]**	−3228.58516	−3228.29079	−3328.21746	−3327.76155	−3327.88965	−3360.12717
***E*(PBE0-D3(CPCM=MeCN)/def2-TZVPP) [Eh]**	−3228.66045	−3228.5030	−3328.42905	−3328.18016	−3328.33071	−3360.55575
**Optimization method**	**PBE0-D3/def2-SVP(CPCM=MeCN)**					
**Compound**	**I'**	**I**	**1**	**2**	**e**	**3**
***E*(optimization method) [Eh]**	−3226.64610	−3226.48840	−3326.128305	−3325.88086	−3326.04134	−3358.40536
***G*(optimization method) [Eh]**	−3225.93540	−3225.77523	−3325.40816	−3325.16587	−3325.31560	−3357.65159
***E*(PBE-D3/def2-TZVPP) [Eh]**	−3228.51196	−3228.21117	−3328.15531	−3327.71236	−3327.80035	−3360.03793
***E*(PBE-D3(CPCM=MeCN)/def2-TZVPP) [Eh]**	−3228.58739	−3228.42609	−3328.36791	−3328.13555	−3328.24577	−3360.47025
***E*(TPSSh-D3/def2-TZVPP) [Eh]**	−3231.34136	−3231.04587	−3331.07152	−3330.62262	−3330.72936	−3363.04971
***E*(TPSSh-D3(CPCM=MeCN)/def2-TZVPP) [Eh]**	−3231.41763	−3231.26058	−3331.28390	−3331.04649	−3331.17490	−3363.48100
***E*(PBE0-D3/def2-TZVPP) [Eh]**	−3228.58352	−3228.28846	−3328.21674	−3327.75840	−3327.88723	−3360.12525
***E*(PBE0-D3(CPCM=MeCN)/def2-TZVPP) [Eh]**	−3228.66303	−3228.50524	−3328.43127	−3328.18482	−3328.33526	−3360.55809

Supplementary Table 27 | Energies of ground states for optimization at the PBE-D3/def2-SVP level of theory with and without CPCM=MeCN for **I'·PF$_6$**, **I·2PF$_6$**, **1·2PF$_6$** and **2·3PF$_6$**.

**Optimization method**	**PBE-D3/def2-SVP**			
**Compound**	**I'·PF$_6$**	**I·2PF$_6$**	**1·2PF$_6$**	**2·3PF$_6$**
***E*(optimization method) [Eh]**	−4167.11497	−5107.56176	−5207.21388	−6147.56502
***G*(optimization method) [Eh]**	−4166.41595	−5106.84482	−5206.49093	−6146.82979
***E*(PBE-D3/def2-TZVPP) [Eh]**	−4171.09138	−5113.48553	−5213.43869	−6155.74654
***E*(PBE-D3(CPCM=MeCN)/def2-TZVPP) [Eh]**	−4171.16065	−5113.56328	−5213.51576	−6155.84090
***E*(TPSSh-D3/def2-TZVPP) [Eh]**	−4174.60904	−5117.70679	−5217.73847	−6160.73336
***E*(TPSSh-D3(CPCM=MeCN)/def2-TZVPP) [Eh]**	−4174.67929	−5117.78501	−5217.81585	−6160.82949
***E*(PBE0-D3/def2-TZVPP) [Eh]**	−4171.16593	−5113.58488	−5213.51758	−6155.82165
***E*(PBE0-D3(CPCM=MeCN)/def2-TZVPP) [Eh]**	−4171.24000	−5113.66634	−5213.59777	−6155.92228
**Optimization method**	**PBE-D3/def2-SVP(CPCM=MeCN)**			
**Compound**	**I'·PF$_6$**	**I·2PF$_6$**	**1·2PF$_6$**	**2·3PF$_6$**
***E*(optimization method) [Eh]**	−4167.18922	−5107.63633	−5207.29063	−6147.65676
***G*(optimization method) [Eh]**	−4166.48936	−5106.92058	−5206.56740	−6146.92284
***E*(PBE-D3/def2-TZVPP) [Eh]**	−4171.09081	−5113.48944	−5213.44240	−6155.75234
***E*(PBE-D3(CPCM=MeCN)/def2-TZVPP) [Eh]**	−4171.17271	−5113.58466	−5213.53198	−6155.86516
***E*(TPSSh-D3/def2-TZVPP) [Eh]**	−4174.60774	−5117.70777	−5217.74149	−6160.73730
***E*(TPSSh-D3(CPCM=MeCN)/def2-TZVPP) [Eh]**	−4174.69088	−5117.80369	−5217.83151	−6160.85193
***E*(PBE0-D3/def2-TZVPP) [Eh]**	−4171.16383	−5113.58630	−5213.52057	−6155.82374
***E*(PBE0-D3(CPCM=MeCN)/def2-TZVPP) [Eh]**	−4171.25132	−5113.68547	−5213.61349	−6155.94295

References

1 Marcó, A., Compañó, R., Rubio, R. & Casals, I. Assessment of Additives for Nitrogen, Carbon, Hydrogen and Sulfur Determination by Organic Elemental Analysis. *Microchim. Acta* **142**, 13-19, doi:10.1007/s00604-002-0956-y (2003).

2 Tinker, L. A. & Bard, A. J. Electrochemistry in Liquid Sulfur Dioxide. 1. Oxidation of Thianthrene, Phenothiazine, and 9,10-Diphenylanthracene. *J. Am. Chem. Soc.* **101**, 2316-2319, doi:10.1021/ja00503a012 (1979).

3 Fulmer, G. R. *et al.* NMR Chemical Shifts of Trace Impurities: Common Laboratory Solvents, Organics, and Gases in Deuterated Solvents Relevant to the Organometallic Chemist. *Organometallics* **29**, 2176-2179, doi:10.1021/om100106e (2010).

4 Gaffney, B. J. & J., S. J. *Simulation of the EMR Spectra of High-Spin Iron in Proteins*. (Plenum Press, 1993).

5 Keilwerth, M. *et al.* From Divalent to Pentavalent Iron Imido Complexes and an Fe(V) Nitride via N-C Bond Cleavage. *J. Am. Chem. Soc.* **145**, 873-887, doi:10.1021/jacs.2c09072 (2023).

6 Neese, F., Wennmohs, F., Becker, U. & Riplinger, C. The ORCA quantum chemistry program package. *J. Chem. Phys.* **152**, 224108, doi:10.1063/5.0004608 (2020).

7 Neese, F. Software update: The ORCA program system—Version 5.0. *Wiley Interdiscip. Rev. Comput. Mol. Sci.* **12**, e1606, doi:10.1002/wcms.1606 (2022).

8 Neese, F. The SHARK integral generation and digestion system. *J. Comput. Chem.* **44**, 381-396, doi:10.1002/jcc.26942 (2023).

9 DeBeer George, S., Petrenko, T. & Neese, F. Prediction of Iron K-Edge Absorption Spectra Using Time-Dependent Density Functional Theory. *J. Phys. Chem. A* **112**, 12936-12943, doi:10.1021/jp803174m (2008).

10 Hirata, S. & Head-Gordon, M. Time-dependent density functional theory within the Tamm–Dancoff approximation. *Chem. Phys. Lett.* **314**, 291-299, doi:10.1016/S0009-2614(99)01149-5 (1999).

11 Perdew, J. P. & Wang, Y. Accurate and simple analytic representation of the electron-gas correlation energy. *Phys. Rev. B.* **45**, 13244-13249, doi:10.1103/physrevb.45.13244 (1992).

12 Perdew, J. P., Burke, K. & Ernzerhof, M. Generalized Gradient Approximation Made Simple. *Phys. Rev. Lett.* **77**, 3865-3868, doi:10.1103/PhysRevLett.77.3865 (1996).

13 Perdew, J. P., Ernzerhof, M. & Burke, K. Rationale for mixing exact exchange with density functional approximations. *J. Chem. Phys.* **105**, 9982-9985, doi:10.1063/1.472933 (1996).

14 Neese, F. An Improvement of the Resolution of the Identity Approximation for the Formation of the Coulomb Matrix. *J. Comput. Chem.* **24**, 1740-1747, doi:10.1002/jcc.10318 (2003).

15 Helmich-Paris, B., de Souza, B., Neese, F. & Izsak, R. An improved chain of spheres for exchange algorithm. *J. Chem. Phys.* **155**, 104109, doi:10.1063/5.0058766 (2021).

16 Neese, F., Wennmohs, F., Hansen, A. & Becker, U. Efficient, approximate and parallel Hartree–Fock and hybrid DFT calculations. A ‘chain-of-spheres’ algorithm for the Hartree–Fock exchange. *Chem. Phys.* **356**, 98-109, doi:10.1016/j.chemphys.2008.10.036 (2009).

17 Izsak, R. & Neese, F. An overlap fitted chain of spheres exchange method. *J. Chem. Phys.* **135**, 144105, doi:10.1063/1.3646921 (2011).

18 Izsák, R., Hansen, A. & Neese, F. The resolution of identity and chain of spheres approximations for the LPNO-CCSD singles Fock term. *Mol. Phys.* **110**, 2413-2417, doi:10.1080/00268976.2012.687466 (2012).

19 Izsak, R., Neese, F. & Klopper, W. Robust fitting techniques in the chain of spheres approximation to the Fock exchange: The role of the complementary space. *J. Chem. Phys.* **139**, 094111, doi:10.1063/1.4819264 (2013).

20 van Wüllen, C. Molecular density functional calculations in the regular relativistic approximation: Method, application to coinage metal diatomics, hydrides, fluorides and chlorides, and comparison with first-order relativistic calculations. *J. Chem. Phys.* **109**, 392-399, doi:10.1063/1.476576 (1998).

21 Weigend, F. & Ahlrichs, R. Balanced basis sets of split valence, triple zeta valence and quadruple zeta valence quality for H to Rn: Design and assessment of accuracy. *Phys. Chem. Chem. Phys.* **7**, doi:10.1039/b508541a (2005).

22 Weigend, F. Accurate Coulomb-fitting basis sets for H to Rn. *Phys. Chem. Chem. Phys.* **8**, 1057-1065, doi:10.1039/B515623H (2006).

23 Garcia-Rates, M. & Neese, F. Effect of the Solute Cavity on the Solvation Energy and its Derivatives within the Framework of the Gaussian Charge Scheme. *J. Comput. Chem.* **41**, 922-939, doi:10.1002/jcc.26139 (2020).

24 Marenich, A. V., Cramer, C. J. & Truhlar, D. G. Universal Solvation Model Based on Solute Electron Density and on a Continuum Model of the Solvent Defined by the Bulk Dielectric Constant and Atomic Surface Tensions. *J. Phys. Chem. B* **113**, 6378-6396, doi:10.1021/jp810292n (2009).

25 Grimme, S., Antony, J., Ehrlich, S. & Krieg, H. A consistent and accurate ab initio parametrization of density functional dispersion correction (DFT-D) for the 94 elements H-Pu. *J. Chem. Phys.* **132**, 154104, doi:10.1063/1.3382344 (2010).
26 Grimme, S., Ehrlich, S. & Goerigk, L. Effect of the Damping Function in Dispersion Corrected Density Functional Theory. *J. Comput. Chem.* **32**, 1456-1465, doi:10.1002/Jcc.21759 (2011).
27 Plasser, F., Wormit, M. & Dreuw, A. New tools for the systematic analysis and visualization of electronic excitations. I. Formalism. *J. Chem. Phys.* **141**, 024106, doi:10.1063/1.4885819 (2014).
28 Humphrey, W., Dalke, A. & Schulten, K. VMD: Visual Molecular Dynamics. *J. Mol. Graph.* **14**, 33-38, doi:10.1016/0263-7855(96)00018-5 (1996).
29 Ahrens, S., Peritz, A. & Strassner, T. Tunable Aryl Alkyl Ionic Liquids (TAAILs): The Next Generation of Ionic Liquids. *Angew. Chem. Int. Ed.* **48**, 7908-7910, doi:10.1002/anie.200903399 (2009).
30 Liu, H. Y., Scharbert, B. & Holm, R. H. An initial approach to biologically related bridged assemblies: pyridinethiolate-linked iron Fe_4S_4-Fe complex systems. *J. Am. Chem. Soc.* **113**, 9529-9539, doi:10.1021/ja00025a018 (1991).
31 Fluck, E. & Meiser, P. Tris(chlormethyl) amin und Bis(chlormethyl)methylamin. Darstellung und chemische Reaktionen. *Chem. Ber.* **106**, 69-77, doi:10.1002/cber.19731060111 (1973).
32 Keilwerth, M. *et al.* Ligand Tailoring Toward an Air-Stable Iron(V) Nitrido Complex. *J. Am. Chem. Soc.* **143**, 1458-1465, doi:10.1021/jacs.0c11141 (2021).
33 Gütlich, P., Bill, E. & Trautwein, A. X. *Mössbauer Spectroscopy and Transition Metal Chemistry*. (Springer-Verlag, 2011).
34 Herber, R. H. & Johnson, D. Lattice Dynamics and Hyperfine Interactions in M_2FeO_4 (M = K^+, Rb^+, Cs^+) and M'FeO_4 (M = Sr^{2+}, Ba^{2+}). *Inorg. Chem.* **18**, 2786-1790, doi:10.1021/ic50200a030 (1979).
35 Shinjo, T., Ichida, T. & Takada, T. Fe^{57} Mössbauer Effect and Magnetic Susceptibility of Hexavalent Iron Compounds; K_2FeO_4, $SrFeO_4$ and $BaFeO_4$. *J. Phys. Soc. Jpn.* **29**, 111-116, doi:10.1143/JPSJ.29.111 (1970).
36 Hoppe, M. L., Schlemper, E. O. & Murmann, R. K. Structure of Dipotassium Ferrate(VI). *Acta Crystallogr. B.* **38**, 2237-2239, doi:10.1107/S0567740882008395 (1982).
37 Schmidbaur, H. The History and the Current Revival of the Oxo Chemistry of Iron in its Highest Oxidation States: Fe^{VI} - Fe^{VIII}. *Z. Anorg. Allg. Chem.* **644**, 536-559, doi:10.1002/zaac.201800036 (2018).
38 Neese, F. Prediction and interpretation of the ^{57}Fe isomer shift in Mössbauer spectra by density functional theory. *Inorg. Chim. Acta* **337**, 181-192, doi:10.1016/S0020-1693(02)01031-9 (2002).
39 Guenzburger, D., Esquivel, D. M. S. & Danon, J. Electronic structure and hyperfine interactions in the FeO_4^{2-} ion. *Phys. Rev. B* **18**, 4561-4569, doi:10.1103/PhysRevB.18.4561 (1978).
40 Souilah, C. *et al.* Synthesis and Characterization of Alkynylferrates(III) and Iron(IV) Alkynylide Complexes. (under revision).
41 Krause, L., Herbst-Irmer, R., Sheldrick, G. M. & Stalke, D. Comparison of silver and molybdenum microfocus X-ray sources for single-crystal structure determination. *J. Appl. Cryst.* **48**, 3-10, doi:10.1107/S1600576714022985 (2015).
42 Sheldrick, G. M. A short history of SHELX. *Acta Crystallogr. A* **64**, 112-122, doi:10.1107/S0108767307043930 (2008).
43 Sheldrick, G. M. Crystal structure refinement with SHELXL. *Acta Crystallogr. C* **71**, 3-8, doi:10.1107/S2053229614024218 (2015).
44 Dolomanov, O. V., Bourhis, L. J., Gildea, R. J., Howard, J. A. K. & Puschmann, H. OLEX2: a complete structure solution, refinement and analysis program. *J. Appl. Cryst.* **42**, 339-341, doi:10.1107/s0021889808042726 (2009).
45 Spek, A. L. PLATON SQUEEZE: a tool for the calculation of the disordered solvent contribution to the calculated structure factors. *Acta. Cryst. C* **71**, 9-18, doi:10.1107/S2053229614024929 (2015).
46 Glendening, E. D. *et al.* NBO 7.0, Theoretical Chemistry Institute, University of Wisconsin, Madison (2018).
47 Keith, T. A. AIMAll (Version 19.10.1), TK Gristmill Software, Overland Park KS, USA, (aim.tkgristmill.com) (2019).
48 Knizia, G. & Klein, J. E. Electron flow in reaction mechanisms – revealed from first principles. *Angew. Chem. Int. Ed.* **54**, 5518-5522, doi:10.1002/anie.201410637 (2015).
49 Zhurko, G. A. Chemcraft graphical program for visualization of quantum chemistry computations, Ivanovo, Russia (2005).
50 Weigend, F. & Ahlrichs, R. Balanced basis sets of split valence, triple zeta valence and quadruple zeta valence quality for H to Rn: Design and assessment of accuracy. *Phys. Chem. Chem. Phys.* **7**, 3297-3305, doi:10.1039/b508541a (2005).
51 Roos, B. O., Taylor, P. R. & Siegbahn, P. E. M. A complete active space SCF method (CASSCF) using a density matrix formulated super-CI approach. *Chem. Phys.* **48**, 157-173, doi:10.1016/0301-0104(80)80045-0 (1980).

52 Angeli, C., Cimiraglia, R., Evangelisti, S., Leininger, T. & Malrieu, J. P. Introduction of n-electron valence states for multireference perturbation theory. *J. Chem. Phys.* **114**, 10252-10264, doi:10.1063/1.1361246 (2001).

53 Adamo, C. & Barone, V. Toward reliable density functional methods without adjustable parameters: the PBE0 model. *J. Chem. Phys.* **110**, 6158-6170 (1999).

54 Staroverov, V. N., Scuseria, G. E., Tao, J. & Perdew, J. P. Comparative assessment of a new nonempirical density functional: Molecules and hydrogen-bonded complexes. *J. Chem. Phys.* **119**, 12129-12137, doi:10.1063/1.1626543 (2003).

55 Chai, J. D. & Head-Gordon, M. Systematic optimization of long-range corrected hybrid density functionals. *J. Chem. Phys.* **128**, 084106, doi:10.1063/1.2834918 (2008).

56 Caldeweyher, E. *et al.* A generally applicable atomic-charge dependent London dispersion correction. *J. Chem. Phys.* **150**, 154122, doi:10.1063/1.5090222 (2019).

57 Knizia, G. Intrinsic Atomic Orbitals: An Unbiased Bridge between Quantum Theory and Chemical Concepts. *J. Chem Theory Comput.* **9**, 4834-4843, doi:10.1021/ct400687b (2013).

58 Mulliken, R. S. Electronic Population Analysis on LCAO–MO Molecular Wave Functions. I. *J. Chem. Phys.* **23**, 1833-1840, doi:10.1063/1.1740588 (1955).

59 Löwdin, P. O. On the Non-Orthogonality Problem Connected with the Use of Atomic Wave Functions in the Theory of Molecules and Crystals. *J. Chem. Phys.* **18**, 365-375, doi:10.1063/1.1747632 (1950).

60 Breneman, C. M. & Wiberg, K. B. Determining atom-centered monopoles from molecular electrostatic potentials. The need for high sampling density in formamide conformational analysis. *J. Comput. Chem.* **11**, 361-373, doi:10.1002/jcc.540110311 (1990).

61 Reed, A. E., Weinstock, R. B. & Weinhold, F. Natural population analysis. *J. Chem. Phys.* **83**, 735-746, doi:10.1063/1.449486 (1985).

62 Nakajima, T. & Hirao, K. The Douglas-Kroll-Hess approach. *Chem. Rev.* **112**, 385-402, doi:10.1021/cr200040s (2012).

63 Reiher, M. Relativistic Douglas–Kroll–Hess theory. *Wiley Interdiscip. Rev. Comput. Mol. Sci.* **2**, 139-149, doi:10.1002/wcms.67 (2011).

64 Becke, A. D. Density-functional exchange-energy approximation with correct asymptotic behavior. *Phys. Rev. A: Gen. Phys.* **38**, 3098-3100 (1988).

65 Perdew, J. P. Density-functional approximation for the correlation energy of the inhomogeneous electron gas. *Phys. Rev. B* **33**, 8822-8824 (1986).

66 Perdew, J. P. Erratum: Density-functional approximation for the correlation energy of the inhomogeneous electron gas. *Phys. Rev. B: Condens. Matter* **34**, 7406-7406, doi:10.1103/PhysRevB.34.7406 (1986).

67 Becke, A. D. Density-Functional Thermochemistry. 3. The Role of Exact Exchange. *J. Chem. Phys.* **98**, 5648-5652, doi:10.1063/1.464913 (1993).

68 Bjornsson, R., Neese, F. & DeBeer, S. Revisiting the Mössbauer Isomer Shifts of the FeMoco Cluster of Nitrogenase and the Cofactor Charge. *Inorg. Chem.* **56**, 1470-1477, doi:10.1021/acs.inorgchem.6b02540 (2017).

69 Pantazis, D. A. & Neese, F. All-electron basis sets for heavy elements. *Wiley Interdiscip. Rev. Comput. Mol. Sci.* **4**, 363-374, doi:10.1002/wcms.1177 (2014).

70 Barone, V. & Cossi, M. Quantum Calculation of Molecular Energies and Energy Gradients in Solution by a Conductor Solvent Model. *J. Phys. Chem. A* **102**, 1995-2001 (1998).

71 Vierk, A.-L. Messung der Dielektrizitätskonstanten von flüssigem SO_2 unter normalem Druck zwischen −16,5 und −68,8 °C. *ZAAC* **261**, 279-282, doi:10.1002/zaac.19502610503 (1950).

72 Musso, M., Aschauer, R., Asenbaum, A., Vasi, C. & Wilhelm, E. Interferometric determination of the refractive index of liquid sulphur dioxide. *Meas. Sci. Technol.* **11**, 1714-1720, doi:10.1088/0957-0233/11/12/310 (2000).

73 Stoychev, G. L., Auer, A. A. & Neese, F. Automatic Generation of Auxiliary Basis Sets. *J. Chem. Theory Comput.* **13**, 554-562, doi:10.1021/acs.jctc.6b01041 (2017).
