# Supplementary material for: The synthesis and characterization of an iron(VII) nitrido complex
Source: Nat Chem. 2024 Jan 30;16(4):514–20. doi: 10.1038/s41557-023-01418-4 (PMC10997499; doi:10.1038/s41557-023-01418-4)
Supplement: Supplementary file 5 — The xyz coordinates for the optimized structures [file 41557_2023_1418_MOESM5_ESM.docx]

**XYZ Coordinates**

2

**HF**

F -5.743716 1.264582 -0.133525

H -4.817522 1.264582 -0.133525

6

**MeCN**

C -6.377377 1.251809 -0.133506

C -4.919283 1.261258 -0.133526

H -4.529096 0.628576 0.682628

H -4.539860 2.288153 0.008490

H -4.530692 0.874550 -1.091696

N -7.547905 1.244137 -0.133538

93

**I' (Hopt)**

Fe 19.829745 4.180861 12.245614

N 20.972297 4.052024 14.377363

N 19.212732 2.537305 14.527676

N 18.434159 1.497817 12.832061

N 22.561400 3.423972 12.802014

N 22.486248 4.015348 10.757531

N 20.378187 6.291045 14.146692

N 18.768131 6.967229 12.929836

N 19.086493 4.264943 10.915357

C 19.935935 3.544631 15.280372

H 20.332067 3.095489 16.214579

H 19.273847 4.392152 15.540302

C 19.087587 2.647602 13.166195

C 18.659168 1.376065 15.008460

H 18.664274 1.117607 16.068389

C 18.172427 0.712212 13.959991

H 17.667335 -0.249799 13.880252

C 22.082038 3.129621 14.151691

H 21.691821 2.096248 14.206511

H 22.916415 3.238263 14.874423

C 21.701502 3.890074 11.864688

C 23.837387 3.251053 12.297893

H 24.666144 2.880220 12.901811

C 23.784450 3.616781 11.029902

H 24.563520 3.665596 10.268746

C 21.397585 5.410344 14.703860

H 21.499325 5.599936 15.792125

H 22.373959 5.594778 14.217254

C 19.579962 5.898422 13.122186

C 20.076282 7.566749 14.584281

H 20.612460 8.051404 15.401121

C 19.073212 7.999866 13.823228

H 18.506499 8.930487 13.832614

C 18.219332 0.977354 11.509822

C 16.923775 0.948560 11.000583

C 16.722385 0.286674 9.789100

H 15.700832 0.237973 9.387668

C 17.755264 -0.299179 9.078962

C 19.034572 -0.223112 9.609122

H 19.869316 -0.697274 9.074689

C 19.299091 0.394796 10.835933

C 15.782877 1.589748 11.736272

H 15.602290 1.121758 12.721685

H 14.848926 1.517281 11.156488

H 15.988054 2.660678 11.922446

C 17.518059 -1.014885 7.775462

H 16.478257 -1.372764 7.689747

H 18.191478 -1.881418 7.661840

H 17.704085 -0.341915 6.916772

C 20.671783 0.369815 11.431582

H 21.067881 1.394417 11.555256

H 21.371234 -0.199146 10.798361

H 20.667123 -0.099292 12.434170

C 22.089475 4.639874 9.525804

C 21.877733 3.837690 8.405096

C 21.565345 4.495062 7.217999

H 21.404209 3.888590 6.315740

C 21.443519 5.861644 7.135776

C 21.644660 6.616515 8.277605

H 21.564541 7.710753 8.229865

C 21.998806 6.021151 9.478670

C 21.977257 2.352025 8.510966

H 21.139530 1.952637 9.112346

H 22.911744 2.018463 8.994600

H 21.925312 1.878431 7.516752

C 21.074950 6.569554 5.854142

H 21.058759 5.881615 4.992413

H 21.787539 7.382419 5.627732

H 20.072421 7.028631 5.934894

C 22.279219 6.865770 10.693256

H 21.516773 6.690832 11.475084

H 22.277688 7.937800 10.439391

H 23.262351 6.624290 11.138763

C 17.562472 6.989505 12.148382

C 17.501761 7.788989 11.009242

C 16.273669 7.872842 10.359054

H 16.196297 8.500997 9.461346

C 15.150982 7.194777 10.809467

C 15.257797 6.419802 11.946070

H 14.370464 5.897166 12.329027

C 16.446157 6.309800 12.644094

C 18.720899 8.524248 10.502189

H 19.498959 7.805227 10.185526

H 19.172631 9.174785 11.271901

H 18.465641 9.153452 9.634464

C 13.832417 7.296506 10.093063

H 13.820800 8.127697 9.368563

H 12.999436 7.448626 10.801771

H 13.614589 6.367093 9.533717

C 16.520357 5.520059 13.934234

H 17.168148 4.632120 13.807319

H 15.518791 5.181239 14.244073

H 16.945742 6.126119 14.755688

66

**I' (Hopt, trunc)**

Fe 0.000000 0.000000 0.000000

N -0.002887 -0.037541 -2.428499

N -1.542840 1.598085 -1.829379

N -2.533874 1.677702 0.070330

N -0.650567 -2.180171 -1.773551

N -0.228230 -3.023934 0.153750

N 2.196419 0.470161 -1.806332

N 2.740742 1.317905 0.084781

N 0.000000 0.000000 1.500243

C -0.544413 1.256020 -2.831223

H 0.280789 1.993384 -2.826355

H -1.012408 1.257570 -3.838001

C -1.444578 1.134913 -0.556129

C -2.646068 2.416640 -2.002085

H -2.890444 2.892211 -2.952883

C -3.279879 2.455204 -0.817958

H -4.188121 2.971442 -0.506930

C -0.843338 -1.183420 -2.790194

H -1.896658 -0.840742 -2.809082

H -0.604055 -1.610929 -3.786951

C -0.269986 -1.840404 -0.516543

C -0.866729 -3.539122 -1.883852

H -1.190159 -4.011767 -2.812716

C -0.609344 -4.075384 -0.690855

H -0.640839 -5.108193 -0.344298

C 1.405186 -0.226507 -2.802402

H 1.622841 -1.311544 -2.781325

H 1.653001 0.160061 -3.813247

C 1.724154 0.673683 -0.546570

C 3.500488 0.935063 -1.939354

H 4.067282 0.846247 -2.867344

C 3.843361 1.461943 -0.761152

H 4.760889 1.950424 -0.432954

C -2.994787 1.334821 1.390440

C -2.482914 1.993881 2.490854

C -3.060463 1.699094 3.733773

H -2.695747 2.226442 4.624919

C -4.079030 0.759321 3.861970

C -4.501269 0.086990 2.737565

H -5.290818 -0.671794 2.827426

C -3.977124 0.358703 1.478280

C 0.295733 -3.232449 1.471162

C -0.552292 -3.112859 2.568173

C -0.034668 -3.375027 3.814564

H -0.697402 -3.290803 4.686789

C 1.281542 -3.745072 4.005909

C 2.104891 -3.828685 2.890835

H 3.158371 -4.113464 3.013447

C 1.621517 -3.573770 1.616750

C 2.596696 1.963108 1.373991

C 2.788874 1.214993 2.521210

C 2.537403 1.878389 3.740226

H 2.663411 1.305943 4.669492

C 2.136419 3.174692 3.803487

C 1.999809 3.886334 2.632989

H 1.698384 4.941913 2.666023

C 2.220592 3.289897 1.393376

H -1.597504 -2.818538 2.427709

H 2.272537 -3.650354 0.738022

H 1.668618 -3.958607 5.008608

H 2.083974 3.845261 0.458901

H 1.945318 3.654081 4.770992

H 3.077275 0.161154 2.478937

H -1.684233 2.734034 2.383101

H -4.334847 -0.156609 0.580472

H -4.521717 0.549778 4.842502

93

**I' (PBE)**

Fe 19.964015 4.137597 12.354428

N 21.202120 4.069757 14.606960

N 19.494713 2.444272 14.664170

N 18.565667 1.533467 12.932485

N 22.759969 3.592140 12.902569

N 22.555267 4.156145 10.820492

N 20.298682 6.230844 14.336817

N 18.624846 6.739112 13.058689

N 19.237640 4.170108 11.030185

C 20.196752 3.442556 15.450647

H 20.614302 2.952925 16.355946

H 19.498239 4.236065 15.777958

C 19.297048 2.632644 13.310424

C 18.895745 1.271737 15.119203

H 18.932783 0.950379 16.160421

C 18.308263 0.699878 14.024107

H 17.741169 -0.224491 13.915779

C 22.358316 3.259506 14.258196

H 22.051527 2.198018 14.320385

H 23.228469 3.408443 14.932196

C 21.818654 3.970627 11.965655

C 24.040435 3.529403 12.357556

H 24.919090 3.240529 12.934882

C 23.907812 3.883441 11.042870

H 24.647865 3.977615 10.248324

C 21.444573 5.486604 14.828830

H 21.609258 5.750884 15.894961

H 22.353965 5.760785 14.260701

C 19.577577 5.768644 13.253887

C 19.822110 7.457604 14.793764

H 20.264515 7.991756 15.635061

C 18.767301 7.777104 13.983364

H 18.099067 8.638109 13.984248

C 18.243411 1.178930 11.576124

C 17.040652 1.639405 11.002161

C 16.761129 1.249646 9.681121

H 15.829411 1.598716 9.216674

C 17.634289 0.428971 8.942350

C 18.822124 -0.007373 9.558759

H 19.516189 -0.647202 8.997803

C 19.153555 0.359356 10.874661

C 16.116570 2.548167 11.765354

H 15.924685 2.180802 12.789234

H 15.149731 2.657917 11.248791

H 16.565112 3.553948 11.854465

C 17.318856 0.054256 7.515605

H 16.237774 -0.112028 7.369147

H 17.853154 -0.859698 7.206488

H 17.619393 0.863856 6.823308

C 20.454329 -0.066548 11.507595

H 21.068672 0.819768 11.758294

H 21.036391 -0.704942 10.823861

H 20.296058 -0.628292 12.446470

C 22.060583 4.731663 9.597761

C 21.508544 3.897698 8.604326

C 21.052851 4.508770 7.422858

H 20.611967 3.875942 6.641280

C 21.143353 5.897475 7.214737

C 21.701947 6.689834 8.235740

H 21.769962 7.777115 8.097784

C 22.162779 6.131691 9.440050

C 21.369362 2.416183 8.822150

H 20.549176 2.214335 9.534321

H 22.287842 1.969065 9.241933

H 21.130517 1.896803 7.880205

C 20.677697 6.518433 5.921352

H 21.498354 6.540502 5.179262

H 20.342918 7.559671 6.066999

H 19.847592 5.947046 5.472379

C 22.702841 6.997655 10.550511

H 22.075650 6.899569 11.457446

H 22.712703 8.058920 10.254674

H 23.732444 6.714530 10.836313

C 17.508296 6.635060 12.157077

C 17.644476 7.069747 10.822662

C 16.520971 6.957404 9.985712

H 16.610276 7.282049 8.940579

C 15.294077 6.441373 10.444512

C 15.207671 6.016861 11.783735

H 14.263164 5.598767 12.156497

C 16.303683 6.097453 12.660090

C 18.959928 7.588814 10.310294

H 19.699421 6.769285 10.270392

H 19.375271 8.378006 10.962730

H 18.857568 7.998581 9.292964

C 14.097620 6.365149 9.529232

H 14.394992 6.132393 8.492301

H 13.559928 7.332000 9.504379

H 13.377790 5.599000 9.863520

C 16.218893 5.580100 14.074211

H 16.926531 4.741345 14.221939

H 15.204524 5.215533 14.302186

H 16.477568 6.355922 14.817650

93

**I' (PBE, CPCM=MeCN)**

Fe 19.961448 4.143896 12.360827

N 21.163608 4.075023 14.552713

N 19.436811 2.486530 14.675274

N 18.507288 1.561392 12.954281

N 22.737533 3.523931 12.897126

N 22.552460 4.072244 10.811766

N 20.344424 6.262643 14.295442

N 18.677225 6.795285 13.022632

N 19.231252 4.182371 11.025715

C 20.172554 3.478054 15.442638

H 20.615621 2.990049 16.332752

H 19.492475 4.279652 15.783760

C 19.251154 2.657864 13.322821

C 18.829396 1.327930 15.143916

H 18.869644 1.025450 16.191101

C 18.238076 0.742445 14.056810

H 17.665413 -0.180222 13.959665

C 22.322947 3.238488 14.261633

H 22.015502 2.181106 14.354976

H 23.177586 3.416659 14.943438

C 21.810948 3.918863 11.959948

C 24.015136 3.424806 12.359448

H 24.880513 3.125262 12.951947

C 23.899825 3.768871 11.039332

H 24.645939 3.841217 10.247590

C 21.460618 5.481441 14.803878

H 21.608169 5.716845 15.876071

H 22.386236 5.738348 14.257368

C 19.609107 5.804345 13.226928

C 19.897945 7.499248 14.745350

H 20.358341 8.020425 15.585662

C 18.844996 7.839633 13.939331

H 18.192481 8.713115 13.940586

C 18.200969 1.197059 11.600065

C 16.968771 1.584349 11.034308

C 16.714940 1.213962 9.699955

H 15.761676 1.512610 9.242798

C 17.644941 0.476828 8.941699

C 18.854284 0.092617 9.555149

H 19.587918 -0.490744 8.982406

C 19.157403 0.443447 10.883054

C 15.976724 2.392465 11.826904

H 15.752878 1.926357 12.803122

H 15.032459 2.515905 11.272136

H 16.381411 3.398500 12.035625

C 17.371093 0.129898 7.498515

H 16.290052 0.133977 7.278155

H 17.780608 -0.861267 7.236749

H 17.848955 0.866359 6.823789

C 20.465756 0.056329 11.523928

H 21.037429 0.958155 11.815275

H 21.083319 -0.540117 10.833183

H 20.307259 -0.531497 12.446833

C 22.066343 4.653675 9.592618

C 21.612428 3.818920 8.551746

C 21.143302 4.434367 7.374895

H 20.779369 3.798129 6.556627

C 21.124877 5.833659 7.222157

C 21.603438 6.629189 8.283347

H 21.601377 7.723009 8.181752

C 22.077202 6.062890 9.479664

C 21.599059 2.322780 8.714505

H 20.885930 2.030747 9.505438

H 22.588551 1.931639 9.012113

H 21.292488 1.824884 7.780274

C 20.587494 6.475540 5.966251

H 20.420661 5.730795 5.170100

H 21.277244 7.248453 5.582049

H 19.622108 6.978590 6.165724

C 22.557287 6.919475 10.623140

H 21.934099 6.750045 11.522135

H 22.510990 7.989188 10.362516

H 23.597315 6.673735 10.906497

C 17.553827 6.693597 12.134665

C 17.634465 7.238205 10.836954

C 16.507619 7.110983 10.001709

H 16.555798 7.524650 8.985153

C 15.329250 6.471862 10.431587

C 15.286271 5.960375 11.744529

H 14.372772 5.465884 12.101840

C 16.386964 6.055408 12.614192

C 18.894690 7.905557 10.355718

H 19.709820 7.166581 10.259183

H 19.245179 8.681632 11.059564

H 18.743298 8.371609 9.368405

C 14.145575 6.314178 9.508562

H 14.236385 6.960351 8.619384

H 13.198250 6.556219 10.022486

H 14.061825 5.268142 9.156377

C 16.345104 5.476060 14.004902

H 17.077938 4.652239 14.106146

H 15.343704 5.079152 14.237416

H 16.609235 6.230415 14.768370

93

**I' (TPSSh)**

Fe 19.954294 4.150083 12.355340

N 21.171344 4.072960 14.571311

N 19.477210 2.449212 14.647830

N 18.578552 1.527370 12.921559

N 22.743029 3.589129 12.895470

N 22.551233 4.169205 10.829804

N 20.296355 6.238844 14.333698

N 18.625336 6.750172 13.074295

N 19.225036 4.199959 11.045731

C 20.167696 3.459237 15.427901

H 20.585655 2.987528 16.329946

H 19.469886 4.248865 15.731933

C 19.290155 2.628081 13.299798

C 18.885027 1.275305 15.103020

H 18.915241 0.963831 16.138567

C 18.317562 0.696618 14.011100

H 17.762183 -0.224601 13.900842

C 22.325657 3.250408 14.244137

H 22.008588 2.201618 14.293180

H 23.177515 3.395147 14.925490

C 21.814687 3.980335 11.963181

C 24.023474 3.519604 12.355211

H 24.889713 3.220432 12.929988

C 23.898432 3.884605 11.051123

H 24.634086 3.977289 10.263994

C 21.439739 5.482658 14.811025

H 21.608910 5.724986 15.870987

H 22.337605 5.749050 14.239991

C 19.569160 5.783072 13.263263

C 19.831098 7.468281 14.790787

H 20.280666 7.996382 15.621057

C 18.777364 7.788408 13.992822

H 18.118226 8.645639 13.994464

C 18.263540 1.177925 11.561908

C 17.091578 1.677403 10.973388

C 16.821069 1.300994 9.652693

H 15.917674 1.680436 9.176165

C 17.674608 0.452258 8.932165

C 18.832250 -0.023837 9.563347

H 19.508958 -0.679905 9.016831

C 19.153615 0.333648 10.878891

C 16.188223 2.616668 11.724347

H 15.974448 2.253165 12.736572

H 15.240077 2.752205 11.196393

H 16.669449 3.597403 11.815588

C 17.366763 0.087474 7.500527

H 16.299853 -0.121787 7.363797

H 17.933582 -0.793059 7.181339

H 17.626961 0.915934 6.827672

C 20.434627 -0.126188 11.528944

H 21.053596 0.739571 11.802177

H 21.010359 -0.757906 10.846484

H 20.247363 -0.698424 12.446418

C 22.046940 4.736848 9.607245

C 21.461848 3.900177 8.645075

C 20.988761 4.497189 7.470068

H 20.526778 3.864799 6.712626

C 21.094375 5.876774 7.243246

C 21.694828 6.671742 8.230196

H 21.784179 7.746065 8.071990

C 22.171130 6.124751 9.427641

C 21.311457 2.424107 8.890190

H 20.528855 2.251376 9.637965

H 22.239130 1.976950 9.266720

H 21.022597 1.903529 7.972716

C 20.541919 6.496650 5.983177

H 20.622852 5.811932 5.132187

H 21.065728 7.424578 5.730732

H 19.477826 6.739646 6.110314

C 22.755239 6.996870 10.510931

H 22.154989 6.920078 11.427892

H 22.769035 8.044920 10.198170

H 23.781604 6.703865 10.765145

C 17.515249 6.645630 12.164871

C 17.668949 7.062534 10.833851

C 16.559783 6.945311 9.988218

H 16.659431 7.258790 8.949483

C 15.331972 6.437728 10.439130

C 15.226004 6.036496 11.778151

H 14.281603 5.634852 12.144096

C 16.310416 6.121774 12.660309

C 18.993438 7.570894 10.333841

H 19.720376 6.750583 10.317139

H 19.394194 8.362702 10.978584

H 18.902547 7.964474 9.317677

C 14.165241 6.293635 9.493021

H 14.094004 7.151225 8.814877

H 13.218731 6.203891 10.035862

H 14.282396 5.393954 8.873442

C 16.211907 5.617902 14.078575

H 16.912320 4.785504 14.233651

H 15.201162 5.259831 14.294084

H 16.461951 6.396965 14.809483

93

**I' (TPSSh, CPCM=MeCN)**

Fe 19.955875 4.152659 12.357712

N 21.138027 4.080249 14.514539

N 19.421033 2.497615 14.662807

N 18.511022 1.561916 12.953007

N 22.723413 3.517128 12.887375

N 22.555392 4.073571 10.816125

N 20.345436 6.270448 14.283213

N 18.675511 6.803533 13.035866

N 19.222881 4.204528 11.034981

C 20.150880 3.500311 15.420861

H 20.596205 3.028919 16.306123

H 19.473645 4.299471 15.741105

C 19.242752 2.656574 13.316986

C 18.816109 1.341703 15.136399

H 18.852092 1.050544 16.178002

C 18.238364 0.749263 14.055760

H 17.674243 -0.168752 13.960362

C 22.293309 3.229744 14.246319

H 21.973974 2.185270 14.328914

H 23.131133 3.404453 14.933282

C 21.811687 3.920273 11.951986

C 24.002165 3.411621 12.358116

H 24.854148 3.105532 12.950831

C 23.897322 3.761840 11.047109

H 24.640654 3.832204 10.264367

C 21.461162 5.479415 14.776426

H 21.615878 5.698692 15.840437

H 22.373222 5.726515 14.222299

C 19.602222 5.818398 13.229293

C 19.907714 7.506725 14.737825

H 20.376030 8.021818 15.566292

C 18.852176 7.846166 13.948531

H 18.205952 8.713617 13.954205

C 18.205100 1.207612 11.596281

C 16.985683 1.613290 11.033560

C 16.736330 1.267676 9.697396

H 15.797415 1.581758 9.241299

C 17.661802 0.535307 8.938459

C 18.859753 0.131238 9.549661

H 19.586620 -0.444209 8.976749

C 19.155934 0.460975 10.878871

C 15.998722 2.417216 11.836774

H 15.719892 1.900999 12.763636

H 15.090920 2.609442 11.257377

H 16.437279 3.380803 12.117784

C 17.394412 0.214094 7.487485

H 16.323518 0.254914 7.261506

H 17.771471 -0.780976 7.225090

H 17.901405 0.939028 6.835348

C 20.457414 0.056956 11.524390

H 21.013888 0.944792 11.853346

H 21.080261 -0.506278 10.823237

H 20.284818 -0.563418 12.413210

C 22.063402 4.641641 9.593556

C 21.617766 3.796016 8.566895

C 21.133478 4.392265 7.393393

H 20.775886 3.750470 6.588158

C 21.094655 5.785838 7.234289

C 21.567272 6.593381 8.281472

H 21.549452 7.677703 8.172954

C 22.053817 6.042316 9.474135

C 21.633514 2.301270 8.744049

H 20.987368 2.016279 9.581329

H 22.642112 1.932405 8.967755

H 21.270256 1.798405 7.842975

C 20.528502 6.408382 5.980532

H 20.521806 5.694320 5.150154

H 21.105040 7.290896 5.680089

H 19.493043 6.735650 6.149095

C 22.530301 6.910291 10.610967

H 21.927153 6.725666 11.510113

H 22.453404 7.969685 10.349050

H 23.573633 6.689774 10.870509

C 17.554480 6.699504 12.145704

C 17.642446 7.234381 10.851209

C 16.527894 7.097971 10.011704

H 16.581025 7.501674 9.000495

C 15.354478 6.456492 10.438480

C 15.304060 5.954590 11.748204

H 14.398354 5.460252 12.098947

C 16.396595 6.059577 12.619863

C 18.907267 7.899549 10.378726

H 19.720768 7.167249 10.333027

H 19.224003 8.698128 11.060413

H 18.773691 8.324471 9.379461

C 14.184828 6.279201 9.500314

H 14.145173 7.083224 8.757296

H 13.235913 6.258187 10.047557

H 14.272776 5.328634 8.955573

C 16.354688 5.484012 14.012537

H 17.097435 4.681285 14.117935

H 15.365045 5.072737 14.232545

H 16.594005 6.244256 14.766687

93

**I' (PBE0)**

Fe 19.921183 4.202316 12.324084

N 21.179320 4.085710 14.593061

N 19.462491 2.487027 14.609669

N 18.578912 1.568979 12.888962

N 22.702099 3.596285 12.876104

N 22.510378 4.153751 10.817320

N 20.293915 6.242331 14.348159

N 18.620964 6.776492 13.122776

N 19.189996 4.312119 11.042698

C 20.164365 3.466350 15.406654

H 20.561229 2.963984 16.303860

H 19.473688 4.254046 15.735172

C 19.277509 2.666570 13.271127

C 18.879399 1.315294 15.059227

H 18.908490 0.999098 16.094624

C 18.317884 0.738471 13.968939

H 17.766627 -0.186495 13.856313

C 22.312902 3.275236 14.230740

H 22.009585 2.222172 14.298343

H 23.186678 3.419531 14.886913

C 21.783249 3.989715 11.949504

C 23.972541 3.503187 12.334196

H 24.839812 3.200303 12.907720

C 23.847469 3.854384 11.030827

H 24.582631 3.929169 10.239533

C 21.429739 5.485769 14.821471

H 21.600582 5.738023 15.880808

H 22.330429 5.760848 14.256707

C 19.561790 5.813953 13.282455

C 19.833794 7.453436 14.835799

H 20.287632 7.964286 15.676011

C 18.776162 7.789575 14.056735

H 18.115236 8.646601 14.084423

C 18.288696 1.194635 11.538485

C 17.125738 1.664367 10.919380

C 16.873812 1.239372 9.613401

H 15.974144 1.597122 9.112562

C 17.733815 0.370260 8.936905

C 18.882521 -0.075498 9.597081

H 19.567175 -0.750809 9.083418

C 19.186266 0.330536 10.896791

C 16.207019 2.615636 11.620662

H 15.976830 2.285719 12.640939

H 15.265938 2.726495 11.074370

H 16.675695 3.604817 11.688682

C 17.450980 -0.048829 7.523395

H 16.375135 -0.083799 7.321819

H 17.872620 -1.035687 7.304832

H 17.894088 0.662973 6.813544

C 20.454653 -0.103289 11.572837

H 21.069166 0.770180 11.831171

H 21.044260 -0.749989 10.916531

H 20.262397 -0.653703 12.502556

C 22.012232 4.689803 9.588285

C 21.491956 3.825285 8.619814

C 21.032425 4.390412 7.429490

H 20.617560 3.733296 6.664958

C 21.084799 5.767055 7.194395

C 21.606802 6.592882 8.194322

H 21.646121 7.670277 8.032593

C 22.074693 6.076309 9.403420

C 21.383872 2.355047 8.878774

H 20.592349 2.164344 9.613503

H 22.315718 1.935820 9.276881

H 21.126826 1.812336 7.964446

C 20.615402 6.339598 5.888529

H 19.765453 5.776879 5.487389

H 21.416632 6.300549 5.138197

H 20.315432 7.387651 5.992660

C 22.593595 6.971377 10.490527

H 21.986777 6.860837 11.399648

H 22.560628 8.020751 10.183410

H 23.630247 6.730437 10.758338

C 17.513856 6.703482 12.219742

C 17.639789 7.215573 10.924102

C 16.526859 7.130649 10.085976

H 16.607436 7.516883 9.069687

C 15.322054 6.562197 10.508242

C 15.244856 6.058830 11.809792

H 14.316669 5.601153 12.152659

C 16.331197 6.113461 12.683663

C 18.938235 7.776987 10.433949

H 19.622815 6.957382 10.181738

H 19.430963 8.401400 11.187872

H 18.789203 8.379067 9.532618

C 14.134753 6.511604 9.591271

H 13.593064 7.466885 9.604131

H 13.428762 5.730107 9.891112

H 14.437202 6.322185 8.555405

C 16.259589 5.512931 14.057057

H 16.958168 4.668594 14.141369

H 15.252676 5.142197 14.269960

H 16.525763 6.236551 14.837710

93

**I' (PBE0, CPCM=MeCN)**

Fe 19.924400 4.172844 12.338237

N 21.143062 4.078256 14.533926

N 19.424450 2.501077 14.638857

N 18.522400 1.566717 12.940175

N 22.691197 3.531245 12.871208

N 22.524129 4.086788 10.812676

N 20.331207 6.255508 14.298439

N 18.668009 6.804858 13.070313

N 19.181382 4.256099 11.044737

C 20.157656 3.482183 15.407845

H 20.594265 2.988132 16.287629

H 19.481178 4.272353 15.755605

C 19.237896 2.661150 13.303922

C 18.839514 1.342494 15.109537

H 18.885256 1.044903 16.150365

C 18.264218 0.749870 14.033338

H 17.710141 -0.175970 13.938714

C 22.285929 3.247662 14.230980

H 21.984837 2.197182 14.322751

H 23.141649 3.420858 14.899270

C 21.786727 3.932767 11.941903

C 23.963713 3.426663 12.345176

H 24.818241 3.121396 12.937150

C 23.858812 3.776269 11.038566

H 24.604171 3.846245 10.255746

C 21.440522 5.468794 14.792251

H 21.589567 5.694020 15.857997

H 22.358005 5.731009 14.251582

C 19.589134 5.824155 13.247725

C 19.898277 7.479154 14.769996

H 20.366423 7.983043 15.607171

C 18.844677 7.829981 13.990128

H 18.199843 8.699963 14.011095

C 18.215308 1.200945 11.595331

C 16.989674 1.578683 11.036934

C 16.732899 1.203559 9.714604

H 15.784486 1.495335 9.261744

C 17.655874 0.469180 8.963527

C 18.860818 0.094606 9.569289

H 19.589081 -0.485278 9.001072

C 19.164134 0.452168 10.884653

C 16.003538 2.384583 11.825680

H 15.792402 1.928405 12.800373

H 15.061134 2.492291 11.280112

H 16.401317 3.387992 12.016865

C 17.378684 0.111561 7.531172

H 16.306686 0.146456 7.309446

H 17.752770 -0.890217 7.290565

H 17.880618 0.816594 6.854018

C 20.467685 0.069542 11.522106

H 21.028138 0.963414 11.827803

H 21.087203 -0.506993 10.828571

H 20.309534 -0.532711 12.426085

C 22.040309 4.642690 9.590869

C 21.609983 3.792330 8.567479

C 21.139123 4.377711 7.388014

H 20.794486 3.729028 6.581785

C 21.098035 5.764860 7.218726

C 21.555352 6.578101 8.262652

H 21.538359 7.662285 8.144841

C 22.031262 6.038703 9.459294

C 21.628025 2.305476 8.750318

H 20.948086 2.015132 9.559189

H 22.626835 1.940375 9.019583

H 21.306950 1.794323 7.837660

C 20.556100 6.375433 5.957806

H 20.488304 5.636480 5.152416

H 21.184228 7.206292 5.615359

H 19.548540 6.779821 6.125939

C 22.505715 6.912892 10.582582

H 21.903734 6.743324 11.485611

H 22.434753 7.970665 10.311935

H 23.548061 6.693214 10.847312

C 17.564820 6.728521 12.168398

C 17.667420 7.307246 10.898168

C 16.569866 7.195344 10.040629

H 16.635427 7.635420 9.044681

C 15.398530 6.533084 10.423742

C 15.332030 5.987541 11.710172

H 14.422997 5.476391 12.029320

C 16.405808 6.069830 12.600312

C 18.929121 7.990009 10.466165

H 19.741476 7.259698 10.374348

H 19.253161 8.746056 11.191423

H 18.798616 8.474463 9.493710

C 14.251540 6.382451 9.465688

H 14.200826 7.224326 8.766408

H 13.295061 6.308425 9.994815

H 14.367369 5.466998 8.868778

C 16.342686 5.450351 13.965145

H 17.076643 4.637259 14.054395

H 15.348015 5.038792 14.161600

H 16.578212 6.179842 14.750309

100

**I'∙PF_6_ (PBE, GP)**

C -0.959750 4.091907 -2.866573

C -2.271401 3.819362 -3.144340

N -2.753006 3.094671 -2.064902

C -1.777180 2.842941 -1.134995

N -0.675388 3.502859 -1.630420

C -4.021796 -1.521390 -1.291317

C -2.794706 -2.114866 -1.300398

C -4.099869 2.556635 -1.943656

N -3.837599 -0.270167 -0.720762

C -2.523398 -0.036262 -0.395847

C -0.285887 3.674596 3.390913

C -4.871417 0.722793 -0.500787

N -1.886895 -1.202492 -0.754726

C -0.482485 -1.491792 -0.649966

C -2.995294 2.210862 1.811190

C -4.820719 3.102630 2.898615

C -3.894650 2.769423 3.847567

N -4.290079 2.056818 -0.590128

C -4.787469 3.040167 0.354641

Fe -1.949715 1.640736 0.339173

N -4.263095 2.748983 1.673998

C 0.056522 -1.756161 0.630910

N -2.793458 2.224828 3.179683

C 1.428804 -2.035270 0.730499

C 2.257164 -2.094675 -0.404641

C 1.669044 -1.887774 -1.663186

C 0.302706 -1.580978 -1.825357

C -0.816799 -1.747693 1.854369

C 3.737203 -2.358412 -0.268974

C -0.245264 -1.324323 -3.203663

C 1.828347 4.962880 0.697827

C 0.623260 4.773552 -0.001415

C 0.551185 3.700108 -0.914267

C 1.637276 2.829411 -1.139731

C 2.819150 3.058922 -0.414821

C 2.934619 4.113676 0.509959

C -0.576176 5.655930 0.231814

C 1.496113 1.670476 -2.084019

C 4.203158 4.304837 1.305853

C 0.576908 1.669129 4.680331

C -0.470739 2.312160 3.998109

C -1.690696 1.615485 3.866753

C -1.880454 0.323577 4.405935

C -0.805779 -0.268514 5.090952

C 0.434169 0.381670 5.229225

C 1.594955 -0.302781 5.908535

C -3.182867 -0.410652 4.207892

N -0.545119 1.426791 0.863869

H -0.201854 4.634878 -3.430076

H -2.875453 3.997494 -4.032692

H -4.968460 -1.838672 -1.723695

H -2.470971 -3.079853 -1.685934

H -4.242637 1.737249 -2.658882

H -4.823937 3.359147 -2.195211

H 0.607896 4.174707 3.797900

H -0.154530 3.575600 2.298216

H -1.163238 4.323506 3.558936

H -5.300578 0.607865 0.513319

H -5.663575 0.553440 -1.256232

H -5.808483 3.554680 2.991270

H -3.916130 2.859836 4.933255

H -5.897445 3.082951 0.416215

H -4.422019 4.028673 0.014545

H 1.855771 -2.225526 1.724731

H 2.293745 -1.955055 -2.564282

H -1.748241 -2.319238 1.690369

H -0.287975 -2.175590 2.721470

H -1.105284 -0.713483 2.107461

H 3.946086 -3.113159 0.509875

H 4.176073 -2.712418 -1.217374

H 4.275986 -1.435709 0.021277

H 0.491759 -1.617977 -3.968804

H -1.192621 -1.844147 -3.417223

H -0.491410 -0.260082 -3.354038

H 1.899804 5.792156 1.414724

H 3.670958 2.383432 -0.572053

H -0.349333 6.455265 0.956173

H -0.925533 6.125333 -0.706294

H -1.421423 5.056996 0.622587

H 1.149742 1.985423 -3.084321

H 2.445405 1.122935 -2.195578

H 0.744069 0.963688 -1.692582

H 5.099865 4.092618 0.697611

H 4.288112 5.333418 1.696168

H 4.230230 3.618117 2.173864

H 1.536640 2.193852 4.780229

H -0.936929 -1.276486 5.507245

H 1.253996 -1.033774 6.661664

H 2.208150 -0.854030 5.169679

H 2.260410 0.422538 6.407719

H -4.016057 0.063127 4.759509

H -3.468199 -0.418620 3.139575

H -3.100742 -1.455040 4.549971

P -3.494515 0.117072 -4.581626

F -2.140702 0.176029 -5.479732

F -3.274552 -1.481232 -4.284034

F -4.417754 -0.212530 -5.869309

F -3.742279 1.742549 -4.796008

F -2.596013 0.483563 -3.227149

F -4.856773 0.088358 -3.595675

100

**I'∙PF_6_ (PBE, SMD=MeCN)**

C -1.311664 4.522428 -2.615388

C -2.629752 4.238567 -2.845076

N -2.989384 3.278680 -1.911830

C -1.940477 2.924720 -1.098520

N -0.905040 3.715957 -1.548403

C -4.013685 -1.572188 -1.510188

C -2.780160 -2.160674 -1.470518

C -4.244991 2.546580 -1.881637

N -3.873741 -0.336015 -0.896847

C -2.584094 -0.103845 -0.487120

C -0.382825 3.688379 3.245550

C -4.940139 0.597642 -0.569581

N -1.914771 -1.260433 -0.837592

C -0.542299 -1.561292 -0.538424

C -3.062606 2.097747 1.810629

C -4.881099 2.901255 2.961174

C -3.914132 2.601270 3.879755

N -4.384491 1.947205 -0.559487

C -4.962030 2.849703 0.430300

Fe -2.050527 1.575710 0.264138

N -4.346255 2.585347 1.720055

C -0.190738 -1.824630 0.807114

N -2.813230 2.108141 3.171011

C 1.162704 -2.059397 1.109755

C 2.156654 -2.069056 0.111905

C 1.753816 -1.883461 -1.224377

C 0.412059 -1.639139 -1.580161

C -1.235871 -1.854695 1.885327

C 3.610252 -2.257808 0.465907

C 0.022851 -1.494925 -3.026206

C 1.995269 4.716072 0.556469

C 0.711227 4.722863 -0.012620

C 0.414943 3.745299 -0.993870

C 1.375194 2.813514 -1.438062

C 2.648217 2.844838 -0.830869

C 2.974431 3.775081 0.171093

C -0.325947 5.734053 0.402882

C 1.086296 1.812879 -2.526070

C 4.332150 3.773435 0.828528

C 0.652916 1.758787 4.519835

C -0.462740 2.327565 3.877513

C -1.656426 1.572805 3.831614

C -1.767728 0.312396 4.464798

C -0.626309 -0.207939 5.102842

C 0.597672 0.488906 5.126319

C 1.822198 -0.123367 5.758845

C -3.064551 -0.456234 4.458955

N -0.620183 1.367755 0.741754

H -0.628044 5.219506 -3.100709

H -3.327278 4.618487 -3.591155

H -4.961336 -1.917816 -1.923965

H -2.435515 -3.130008 -1.830653

H -4.223146 1.747680 -2.636593

H -5.070823 3.242628 -2.123041

H 0.528112 4.221603 3.563530

H -0.358452 3.589845 2.146578

H -1.261501 4.308908 3.496753

H -5.329661 0.379562 0.440993

H -5.758322 0.470487 -1.303207

H -5.885330 3.310413 3.079808

H -3.905905 2.683759 4.966910

H -6.059481 2.742446 0.530504

H -4.736244 3.884305 0.116450

H 1.443515 -2.247365 2.155403

H 2.505664 -1.926017 -2.024383

H -2.123889 -2.436081 1.578123

H -0.831880 -2.291460 2.812805

H -1.580385 -0.830253 2.107932

H 3.730472 -2.861016 1.382808

H 4.169117 -2.746347 -0.351337

H 4.096730 -1.280500 0.653747

H 0.914077 -1.339887 -3.657229

H -0.487367 -2.405974 -3.391354

H -0.679399 -0.666263 -3.189987

H 2.236019 5.459226 1.328919

H 3.403327 2.114231 -1.152696

H 0.009850 6.305023 1.284275

H -0.530906 6.455297 -0.411042

H -1.289893 5.251567 0.646863

H 0.028199 1.786056 -2.828646

H 1.685205 2.040070 -3.428532

H 1.375122 0.800243 -2.197887

H 4.997399 3.014458 0.382846

H 4.825491 4.758933 0.737200

H 4.248772 3.558949 1.910802

H 1.590171 2.331066 4.551884

H -0.695658 -1.192639 5.584731

H 1.566179 -0.710509 6.658478

H 2.320719 -0.815635 5.052275

H 2.561561 0.645863 6.041138

H -3.789870 -0.025992 5.175303

H -3.549632 -0.437883 3.466842

H -2.898391 -1.507800 4.745740

P -2.895684 0.557605 -4.831764

F -1.436211 0.891397 -5.495531

F -2.518588 -1.037190 -4.765995

F -3.558474 0.355215 -6.310420

F -3.291140 2.154961 -4.864706

F -2.232866 0.774373 -3.324205

F -4.354907 0.230482 -4.119349

93

**I (Hopt)**

Fe 11.942907 7.753715 4.060466

N 9.919958 6.825482 3.375434

N 10.618710 8.078622 1.518727

N 12.580627 8.906986 1.299730

N 11.229479 4.983899 3.978955

N 12.825121 5.083959 5.406271

N 9.376096 8.135763 5.249190

N 10.631771 9.699459 6.020095

N 13.219495 8.428040 4.563234

C 9.436023 7.598380 2.219857

H 8.801627 7.007780 1.530918

H 8.848146 8.456284 2.592941

C 11.741016 8.377958 2.215232

C 10.749351 8.409282 0.186717

H 9.956182 8.251612 -0.546277

C 11.985068 8.937969 0.047056

H 12.510831 9.318656 -0.829554

C 10.223997 5.430574 3.023801

H 9.343169 4.759846 3.067684

H 10.633869 5.412188 1.996885

C 12.046895 5.860459 4.614288

C 11.480199 3.679360 4.353353

H 10.939789 2.824777 3.943214

C 12.484317 3.742888 5.253120

H 13.003618 2.958245 5.804885

C 9.012580 6.918738 4.535308

H 7.943456 6.954849 4.250061

H 9.176474 6.036923 5.180975

C 10.631042 8.633406 5.193571

C 8.584888 8.877964 6.115066

H 7.539265 8.638793 6.314659

C 9.375913 9.858688 6.601653

H 9.168555 10.669230 7.300943

C 13.935942 9.308468 1.563114

C 14.170825 10.644051 1.911041

C 15.491782 11.004442 2.162241

H 15.710808 12.042733 2.444385

C 16.526045 10.093695 2.055394

C 16.249263 8.781807 1.691230

H 17.069361 8.058348 1.597079

C 14.939765 8.358128 1.438001

C 13.043398 11.635150 1.993200

H 13.408121 12.616622 2.332954

H 12.269541 11.299876 2.703661

H 12.554610 11.781922 1.012009

C 17.951653 10.539452 2.301309

H 18.363428 11.028311 1.398398

H 18.611320 9.690415 2.544644

H 18.009597 11.273512 3.122837

C 14.626409 6.940703 1.049287

H 13.916230 6.473261 1.758629

H 15.538404 6.323168 1.035817

H 14.171363 6.878526 0.043234

C 13.744044 5.557625 6.414027

C 15.012700 6.005478 6.050960

C 15.855088 6.448455 7.077263

H 16.858598 6.806954 6.813340

C 15.468198 6.424742 8.413852

C 14.194630 5.958038 8.728712

H 13.868765 5.939914 9.776998

C 13.311541 5.518826 7.746361

C 15.488985 6.026640 4.620693

H 14.950377 5.305250 3.984111

H 16.563806 5.785542 4.568164

H 15.356860 7.030219 4.173877

C 16.397683 6.889229 9.509167

H 16.795159 6.025420 10.074094

H 15.872225 7.532807 10.236212

H 17.258805 7.447866 9.107996

C 11.930595 5.051383 8.115983

H 11.153816 5.532528 7.491567

H 11.706840 5.288086 9.168230

H 11.809784 3.958438 7.999362

C 11.688828 10.676105 6.108584

C 12.776334 10.452559 6.955192

C 13.785338 11.415176 6.957027

H 14.658130 11.257515 7.604334

C 13.731158 12.545810 6.151125

C 12.608007 12.745283 5.351486

H 12.540224 13.645053 4.726164

C 11.567058 11.820791 5.316243

C 12.874951 9.224300 7.817253

H 11.899214 8.931401 8.242830

H 13.573722 9.387064 8.653256

H 13.253446 8.360067 7.240349

C 14.869687 13.541790 6.125887

H 14.695837 14.355424 6.855393

H 14.970881 14.013337 5.133773

H 15.829780 13.067382 6.388474

C 10.362284 12.051285 4.443224

H 9.461454 12.289619 5.039043

H 10.115097 11.162921 3.831164

H 10.527878 12.898235 3.758625

66

**I (Hopt, trunc)**

Fe 0.000000 0.000000 0.000000

N -0.149689 -0.005589 -2.323958

N 0.323143 2.231840 -1.798328

N 0.957505 2.902385 0.133315

N 1.502000 -1.590626 -1.844282

N 1.600240 -2.675120 0.001663

N -2.261508 -0.724051 -1.583939

N -3.002160 -0.407229 0.407847

N 0.000000 0.000000 1.528781

C -0.356237 1.382868 -2.767172

H 0.033461 1.582941 -3.784527

H -1.439973 1.598191 -2.760251

C 0.392498 1.852360 -0.500067

C 0.838824 3.497224 -1.981437

H 0.874615 3.993983 -2.952823

C 1.234594 3.925098 -0.762293

H 1.706608 4.856737 -0.447025

C 1.126779 -0.557790 -2.800992

H 1.069689 -0.994455 -3.818267

H 1.879502 0.252366 -2.809370

C 1.047815 -1.575912 -0.566135

C 2.326124 -2.670745 -2.087218

H 2.806963 -2.856201 -3.049205

C 2.386780 -3.354134 -0.924816

H 2.918045 -4.269728 -0.660247

C -1.290505 -0.879427 -2.659076

H -1.761011 -0.627445 -3.629602

H -0.935806 -1.924974 -2.704976

C -1.876352 -0.382103 -0.334799

C -3.625938 -0.974244 -1.632496

H -4.144169 -1.277521 -2.543676

C -4.090088 -0.778009 -0.379356

H -5.097900 -0.851481 0.031143

C 1.302963 2.931118 1.528755

C 0.364567 3.450734 2.428429

C 0.724704 3.464667 3.773048

H 0.013545 3.863383 4.507801

C 1.956660 3.005637 4.199845

C 2.864251 2.513201 3.270294

H 3.848785 2.163146 3.605855

C 2.553787 2.463053 1.906653

C 1.285598 -3.199843 1.309338

C 1.865146 -2.641223 2.446864

C 1.519675 -3.199140 3.683198

H 1.966335 -2.775634 4.591996

C 0.655286 -4.284640 3.789223

C 0.109719 -4.815880 2.623433

H -0.568436 -5.676817 2.687741

C 0.408329 -4.290932 1.369229

C -3.118421 0.105620 1.750419

C -2.793211 -0.706808 2.838341

C -2.883258 -0.132879 4.106110

H -2.635778 -0.748675 4.980448

C -3.267378 1.189126 4.294543

C -3.619552 1.949510 3.181685

H -3.962768 2.983305 3.318725

C -3.555915 1.425163 1.893076

H -0.607560 3.815649 2.081425

H 2.221986 3.039133 5.262823

H 3.267314 2.085694 1.165748

H -2.479332 -1.745834 2.695279

H -3.830985 2.023043 1.016656

H -3.326420 1.614316 5.302698

H -0.022970 -4.716226 0.455725

H 2.561586 -1.799575 2.372776

H 0.414974 -4.715466 4.767520

93

**I (PBE)**

Fe 11.952859 7.706661 4.022546

N 9.871664 6.728653 3.356432

N 10.445354 8.229079 1.617899

N 12.419113 9.097399 1.385612

N 11.300266 4.934208 3.847033

N 12.905388 5.082900 5.296201

N 9.423233 7.943567 5.324109

N 10.670941 9.577229 6.012670

N 13.223294 8.388699 4.502539

C 9.325507 7.580156 2.291072

H 8.731475 7.013530 1.551081

H 8.679993 8.349235 2.749131

C 11.614310 8.453498 2.284237

C 10.497159 8.725622 0.328438

H 9.664192 8.638139 -0.370602

C 11.748700 9.278813 0.181947

H 12.221065 9.760867 -0.674756

C 10.270997 5.384589 2.918350

H 9.436025 4.659008 2.921473

H 10.682246 5.456755 1.895980

C 12.088109 5.839092 4.506922

C 11.619311 3.635667 4.196201

H 11.110443 2.768565 3.772971

C 12.636097 3.729766 5.116058

H 13.181791 2.959078 5.661746

C 9.065255 6.738735 4.588657

H 7.978178 6.740215 4.389155

H 9.314070 5.844503 5.185913

C 10.665337 8.488521 5.193154

C 8.649509 8.666612 6.223323

H 7.623062 8.390546 6.467488

C 9.439072 9.698140 6.661041

H 9.237315 10.509579 7.360793

C 13.790233 9.445263 1.650311

C 14.116756 10.788601 1.932888

C 15.446309 11.067125 2.300583

H 15.717232 12.102993 2.545489

C 16.429578 10.060634 2.364460

C 16.068971 8.746463 2.003840

H 16.832298 7.957061 2.006883

C 14.752774 8.410066 1.640776

C 13.093539 11.885771 1.808753

H 13.444195 12.807579 2.298291

H 12.127106 11.598921 2.254025

H 12.900524 12.119087 0.744507

C 17.829946 10.376844 2.826092

H 18.570354 9.698873 2.368192

H 17.909382 10.255436 3.923769

H 18.109474 11.418238 2.591854

C 14.381249 7.004108 1.245326

H 13.695719 6.541738 1.981025

H 15.277014 6.366903 1.174196

H 13.859610 6.985751 0.271230

C 13.805939 5.584335 6.303678

C 15.113922 5.972662 5.946961

C 15.952819 6.438080 6.978524

H 16.974017 6.750951 6.722736

C 15.527262 6.501988 8.318159

C 14.218180 6.081170 8.627036

H 13.866895 6.128352 9.666330

C 13.331179 5.628950 7.635976

C 15.601708 5.918271 4.523620

H 15.168502 5.070832 3.966061

H 16.700626 5.838703 4.489372

H 15.319283 6.841272 3.985197

C 16.432134 7.045881 9.395083

H 16.286154 6.515484 10.351967

H 16.210310 8.115042 9.579453

H 17.494653 6.973571 9.107863

C 11.914183 5.239462 7.972840

H 11.188101 5.812548 7.365820

H 11.700258 5.431351 9.036376

H 11.722675 4.168993 7.773770

C 11.735057 10.544625 6.070183

C 12.830656 10.320094 6.929538

C 13.886360 11.249797 6.892891

H 14.755724 11.084194 7.543336

C 13.858695 12.375098 6.046999

C 12.718136 12.583488 5.245058

H 12.668287 13.470400 4.600051

C 11.642106 11.679009 5.233109

C 12.878223 9.114011 7.825130

H 11.946832 8.999517 8.408300

H 13.725441 9.173145 8.526861

H 13.001029 8.190772 7.232308

C 15.032177 13.319545 5.970156

H 14.703789 14.363029 5.822505

H 15.677200 13.057342 5.108824

H 15.656149 13.269625 6.878473

C 10.433447 11.900982 4.359321

H 9.513561 12.012744 4.962308

H 10.264249 11.044581 3.679157

H 10.552811 12.809596 3.748039

93

**I (PBE, CPCM=MeCN)**

Fe 11.955141 7.718315 4.025013

N 9.878022 6.733061 3.356509

N 10.455897 8.224218 1.611708

N 12.430327 9.090965 1.379607

N 11.307606 4.943752 3.861390

N 12.905245 5.103232 5.317685

N 9.420245 7.957366 5.315945

N 10.661942 9.597720 5.999304

N 13.222644 8.404668 4.506452

C 9.334360 7.578641 2.285216

H 8.742667 7.007887 1.546582

H 8.687277 8.349853 2.737445

C 11.622625 8.453641 2.280319

C 10.511849 8.711394 0.318892

H 9.681073 8.618859 -0.382098

C 11.763819 9.263598 0.172507

H 12.239001 9.739604 -0.686018

C 10.281312 5.387664 2.926282

H 9.447625 4.660618 2.929509

H 10.696589 5.455668 1.905249

C 12.090758 5.853556 4.520081

C 11.627006 3.647721 4.219449

H 11.121578 2.777457 3.798593

C 12.639036 3.748624 5.143814

H 13.183098 2.981896 5.696659

C 9.067492 6.747691 4.586002

H 7.981065 6.745382 4.382980

H 9.316730 5.857342 5.188836

C 10.661650 8.504235 5.186240

C 8.641672 8.684086 6.207977

H 7.614904 8.407266 6.449932

C 9.427464 9.719854 6.642491

H 9.221495 10.535120 7.336545

C 13.801014 9.440823 1.644269

C 14.126118 10.785430 1.920103

C 15.454851 11.066261 2.290711

H 15.723377 12.102311 2.537432

C 16.438973 10.061684 2.359323

C 16.077827 8.743300 2.013078

H 16.839111 7.952070 2.032442

C 14.763275 8.404614 1.648012

C 13.102364 11.881460 1.791483

H 13.451120 12.804634 2.279812

H 12.134730 11.595128 2.234373

H 12.912382 12.112495 0.726177

C 17.856087 10.391636 2.756964

H 18.494010 10.520530 1.861608

H 18.303095 9.581613 3.359135

H 17.904434 11.329576 3.335690

C 14.391011 6.994532 1.268223

H 13.702802 6.541197 2.007035

H 15.286072 6.355381 1.206419

H 13.871930 6.966212 0.292996

C 13.798505 5.611818 6.328057

C 15.109200 5.997104 5.977981

C 15.941121 6.468645 7.012315

H 16.964336 6.779077 6.761703

C 15.506111 6.541620 8.348515

C 14.194543 6.123960 8.650844

H 13.835989 6.178303 9.687297

C 13.314254 5.665672 7.656505

C 15.606179 5.933009 4.558219

H 15.184861 5.075317 4.007116

H 16.706004 5.864091 4.531318

H 15.317639 6.847329 4.008389

C 16.403989 7.091797 9.428101

H 16.246984 6.571602 10.388807

H 16.185836 8.163829 9.599806

H 17.468641 7.011672 9.151008

C 11.894549 5.279573 7.985796

H 11.173283 5.852846 7.373161

H 11.674417 5.474391 9.047543

H 11.702041 4.209067 7.787963

C 11.723634 10.567900 6.054865

C 12.815112 10.352759 6.921823

C 13.868464 11.285067 6.883908

H 14.734744 11.126674 7.540220

C 13.842437 12.403885 6.029363

C 12.705911 12.602913 5.219358

H 12.657355 13.484631 4.567177

C 11.632439 11.695379 5.208382

C 12.860876 9.153582 7.826729

H 11.928511 9.043780 8.409305

H 13.706955 9.217649 8.529410

H 12.984019 8.225679 7.241302

C 15.013936 13.350743 5.951785

H 14.683970 14.392161 5.793506

H 15.665037 13.083015 5.096691

H 15.632410 13.309894 6.864310

C 10.428427 11.906277 4.325472

H 9.504380 12.018967 4.921872

H 10.267139 11.043800 3.651103

H 10.548071 12.810602 3.707914

93

**I (TPSSh)**

Fe 11.972830 7.711631 4.008876

N 9.932326 6.738949 3.365071

N 10.459231 8.231560 1.613617

N 12.412618 9.110528 1.364791

N 11.335104 4.940295 3.870927

N 12.901146 5.094180 5.343346

N 9.454225 7.952752 5.316289

N 10.674678 9.603282 5.976008

N 13.234593 8.389084 4.477811

C 9.354177 7.574523 2.301143

H 8.769157 6.993917 1.580221

H 8.713593 8.331927 2.762269

C 11.622696 8.470630 2.264917

C 10.496824 8.723150 0.322414

H 9.663768 8.628802 -0.361832

C 11.735261 9.281239 0.165330

H 12.195122 9.762259 -0.687645

C 10.324134 5.392461 2.923341

H 9.489340 4.682416 2.918824

H 10.750517 5.467158 1.917953

C 12.108929 5.840301 4.535770

C 11.637713 3.644234 4.240343

H 11.133402 2.784146 3.819841

C 12.629436 3.741936 5.173961

H 13.157282 2.982402 5.735251

C 9.116377 6.735186 4.593030

H 8.041500 6.721218 4.383985

H 9.380064 5.858946 5.192544

C 10.682182 8.507592 5.180782

C 8.670726 8.680031 6.201089

H 7.655584 8.396762 6.445755

C 9.442969 9.722056 6.619679

H 9.234650 10.535910 7.301032

C 13.779339 9.471500 1.634971

C 14.090996 10.813316 1.906307

C 15.408689 11.104464 2.285736

H 15.668746 12.136408 2.519440

C 16.391059 10.106448 2.377094

C 16.045164 8.789679 2.032833

H 16.805626 8.009964 2.060611

C 14.740670 8.444942 1.656193

C 13.058815 11.900558 1.763266

H 13.398687 12.821072 2.244638

H 12.102173 11.605921 2.204186

H 12.876248 12.118277 0.702455

C 17.782986 10.437856 2.855525

H 18.061420 11.463850 2.591446

H 18.523602 9.750272 2.433117

H 17.836558 10.351908 3.949872

C 14.378212 7.030441 1.280783

H 13.704240 6.579097 2.021946

H 15.274525 6.407042 1.218888

H 13.860226 6.999067 0.314524

C 13.796992 5.609563 6.347634

C 15.090920 6.014323 5.984688

C 15.923107 6.499304 7.005557

H 16.930491 6.824438 6.746866

C 15.501581 6.561471 8.340901

C 14.205288 6.123956 8.657049

H 13.859447 6.171677 9.689115

C 13.326491 5.654970 7.674149

C 15.576146 5.952368 4.560011

H 15.147876 5.101636 4.019925

H 16.667374 5.876088 4.529633

H 15.289716 6.864266 4.022456

C 16.403437 7.124241 9.411590

H 16.256054 6.608084 10.366707

H 16.179821 8.187968 9.574340

H 17.457683 7.045650 9.126057

C 11.913468 5.253478 8.016055

H 11.191026 5.816739 7.410863

H 11.706873 5.450568 9.071820

H 11.736780 4.187435 7.825638

C 11.740986 10.568870 6.032727

C 12.837495 10.330050 6.875879

C 13.891339 11.253460 6.845624

H 14.757676 11.079118 7.483380

C 13.858020 12.386313 6.018462

C 12.719882 12.604919 5.225694

H 12.669024 13.491569 4.595109

C 11.646584 11.704611 5.210125

C 12.891501 9.108763 7.750800

H 11.961006 8.975234 8.315302

H 13.723867 9.172840 8.456754

H 13.035850 8.208887 7.142166

C 15.030941 13.333751 5.956820

H 14.703986 14.359200 5.752451

H 15.713468 13.037404 5.148050

H 15.600660 13.324190 6.892199

C 10.434040 11.937001 4.343745

H 9.529943 12.064914 4.952505

H 10.255387 11.084289 3.675352

H 10.564326 12.835231 3.733677

93

**I (TPSSh, CPCM=MeCN)**

Fe 11.972840 7.706043 4.006628

N 9.930881 6.739744 3.360364

N 10.465711 8.228991 1.608669

N 12.423167 9.099213 1.361116

N 11.326785 4.937118 3.870065

N 12.887795 5.086890 5.348172

N 9.453375 7.955320 5.310517

N 10.677348 9.602871 5.971089

N 13.236138 8.379864 4.476520

C 9.357194 7.576916 2.295289

H 8.770710 6.998201 1.574010

H 8.719052 8.337096 2.755271

C 11.629224 8.464507 2.261309

C 10.507378 8.717637 0.316494

H 9.674941 8.625449 -0.368799

C 11.748326 9.270301 0.160252

H 12.211566 9.747796 -0.692877

C 10.319946 5.392150 2.919516

H 9.483140 4.684492 2.912466

H 10.749413 5.465774 1.915344

C 12.101395 5.835024 4.536933

C 11.623229 3.640331 4.241956

H 11.117385 2.781673 3.820368

C 12.611807 3.735405 5.179152

H 13.134771 2.974608 5.743291

C 9.112972 6.738606 4.587037

H 8.038387 6.727783 4.376329

H 9.373052 5.861693 5.187139

C 10.682947 8.506896 5.176281

C 8.670765 8.684843 6.194253

H 7.654604 8.404289 6.437805

C 9.445259 9.724951 6.613422

H 9.238324 10.539590 7.294257

C 13.791225 9.456056 1.630482

C 14.106209 10.796888 1.901676

C 15.424883 11.084736 2.281141

H 15.686051 12.115037 2.520818

C 16.405602 10.085229 2.369061

C 16.053983 8.767315 2.033926

H 16.810074 7.983732 2.072106

C 14.748841 8.425770 1.657520

C 13.075514 11.886164 1.763869

H 13.419366 12.805711 2.244234

H 12.120124 11.593250 2.208661

H 12.888754 12.104716 0.703970

C 17.817728 10.425043 2.778922

H 18.437451 10.625711 1.894095

H 18.280955 9.595711 3.324708

H 17.840814 11.319441 3.410729

C 14.380214 7.009997 1.293230

H 13.707053 6.566023 2.039586

H 15.274118 6.383008 1.232738

H 13.859090 6.973928 0.328846

C 13.780817 5.599672 6.356311

C 15.078712 5.997215 5.999716

C 15.908176 6.478674 7.024476

H 16.918685 6.797941 6.770749

C 15.479977 6.544644 8.357493

C 14.179548 6.114853 8.667233

H 13.828501 6.165808 9.697383

C 13.303503 5.649303 7.680259

C 15.571102 5.931137 4.577650

H 15.141544 5.081796 4.036376

H 16.662097 5.849550 4.553066

H 15.291899 6.843667 4.037319

C 16.379305 7.103323 9.432436

H 16.225610 6.587328 10.386655

H 16.159079 8.167828 9.594727

H 17.434470 7.020716 9.151524

C 11.886672 5.255481 8.015191

H 11.170288 5.821247 7.405190

H 11.675263 5.455380 9.069480

H 11.705744 4.189977 7.825660

C 11.744816 10.567238 6.027308

C 12.840729 10.328611 6.871035

C 13.894854 11.251974 6.840761

H 14.760915 11.077821 7.478913

C 13.862246 12.384337 6.013161

C 12.724706 12.602461 5.219157

H 12.674531 13.488486 4.587613

C 11.651440 11.702393 5.203479

C 12.894421 9.107774 7.746663

H 11.963469 8.974200 8.310400

H 13.726153 9.172497 8.453316

H 13.039453 8.207515 7.138722

C 15.034503 13.332613 5.951581

H 14.704046 14.363298 5.781474

H 15.698918 13.058185 5.120324

H 15.623841 13.298206 6.874035

C 10.439948 11.934061 4.335404

H 9.535308 12.063973 4.942940

H 10.261277 11.080212 3.668467

H 10.571660 12.830994 3.723714

93

**I (PBE0)**

Fe 11.967381 7.839821 4.131086

N 9.880699 6.789456 3.336006

N 10.578483 8.227039 1.606173

N 12.582911 8.959909 1.380916

N 11.328969 5.039566 3.903189

N 12.908405 5.201918 5.348249

N 9.316702 8.033042 5.239559

N 10.443247 9.702261 5.974912

N 13.121612 8.526513 4.740741

C 9.421732 7.647618 2.257519

H 8.818660 7.116673 1.506685

H 8.803404 8.442402 2.691778

C 11.732500 8.434562 2.281387

C 10.687683 8.641884 0.296587

H 9.877063 8.573748 -0.419732

C 11.961224 9.106812 0.158475

H 12.479491 9.510593 -0.703357

C 10.333511 5.467813 2.940940

H 9.526352 4.720549 2.907296

H 10.777995 5.538695 1.939785

C 12.095814 5.937161 4.569681

C 11.660580 3.747426 4.252330

H 11.175429 2.873927 3.832481

C 12.661174 3.855565 5.169552

H 13.212994 3.095873 5.710411

C 9.038280 6.811301 4.520876

H 7.964206 6.767906 4.288429

H 9.288096 5.941476 5.140372

C 10.537898 8.613826 5.191473

C 8.457821 8.740853 6.060548

H 7.431844 8.443640 6.242647

C 9.176035 9.795507 6.525059

H 8.901373 10.606270 7.188987

C 13.986409 9.176744 1.604097

C 14.434619 10.434864 2.018876

C 15.808989 10.590371 2.211876

H 16.183742 11.561761 2.533663

C 16.713540 9.546776 1.996811

C 16.212826 8.307811 1.580020

H 16.904904 7.484855 1.402182

C 14.850418 8.095784 1.373891

C 13.484609 11.561692 2.287267

H 14.005017 12.523239 2.254941

H 13.046927 11.459946 3.288091

H 12.662839 11.600518 1.563552

C 18.187465 9.758785 2.169431

H 18.632837 10.118248 1.232025

H 18.700977 8.829919 2.437955

H 18.400142 10.507674 2.939131

C 14.330236 6.759167 0.929200

H 13.659765 6.319105 1.681156

H 15.152851 6.056423 0.770418

H 13.770107 6.828842 -0.011907

C 13.856016 5.726941 6.292272

C 15.130440 6.097132 5.847038

C 16.020090 6.593398 6.802351

H 17.019022 6.887918 6.481520

C 15.674997 6.708525 8.151777

C 14.388600 6.320582 8.544775

H 14.101672 6.407100 9.592679

C 13.455883 5.828634 7.632757

C 15.528767 5.994233 4.404949

H 15.127442 5.093833 3.925927

H 16.617857 5.967161 4.307075

H 15.174258 6.864870 3.838135

C 16.667487 7.202749 9.161080

H 17.188380 6.356786 9.629276

H 16.180771 7.766958 9.963699

H 17.428345 7.841046 8.701046

C 12.068832 5.448922 8.064893

H 11.308712 6.001643 7.495410

H 11.918807 5.671467 9.124884

H 11.870004 4.378723 7.924548

C 11.460180 10.698301 6.156712

C 12.426023 10.516507 7.153737

C 13.387538 11.517290 7.301526

H 14.152937 11.396844 8.067958

C 13.389750 12.669084 6.508993

C 12.395410 12.807561 5.533709

H 12.379899 13.702205 4.911195

C 11.417464 11.832795 5.334372

C 12.445571 9.287563 8.010767

H 11.448370 9.030672 8.387735

H 13.104144 9.427479 8.872352

H 12.823894 8.424468 7.448998

C 14.410063 13.746285 6.724053

H 14.059183 14.458197 7.483193

H 14.597150 14.314618 5.807293

H 15.360954 13.335158 7.078329

C 10.368695 11.984443 4.270133

H 9.357126 12.028493 4.693467

H 10.391806 11.142927 3.563480

H 10.524778 12.904279 3.699510

93

**I (PBE0, CPCM=MeCN)**

Fe 11.994563 7.698055 3.994238

N 9.931579 6.743743 3.356518

N 10.491588 8.205738 1.598231

N 12.434735 9.078904 1.343405

N 11.310117 4.942190 3.889094

N 12.861178 5.074053 5.362068

N 9.487749 7.972402 5.301671

N 10.719536 9.597667 5.968088

N 13.253987 8.351220 4.434617

C 9.385264 7.564845 2.281875

H 8.800128 6.981561 1.560094

H 8.736844 8.332687 2.717863

C 11.648333 8.449606 2.240626

C 10.528424 8.683878 0.308741

H 9.695209 8.584799 -0.376946

C 11.762364 9.240117 0.148061

H 12.219847 9.718413 -0.709605

C 10.312407 5.397954 2.942985

H 9.469995 4.693777 2.940338

H 10.738122 5.447828 1.934020

C 12.096956 5.822926 4.544607

C 11.574606 3.649230 4.274513

H 11.048934 2.795027 3.864615

C 12.559824 3.733221 5.209614

H 13.063472 2.965665 5.784934

C 9.125213 6.773405 4.575470

H 8.047808 6.785533 4.369596

H 9.354092 5.888763 5.179615

C 10.712736 8.513670 5.169774

C 8.717640 8.694729 6.190662

H 7.698874 8.420986 6.437625

C 9.498871 9.722839 6.614355

H 9.296873 10.533717 7.303537

C 13.783336 9.470537 1.617764

C 14.054024 10.815240 1.900354

C 15.355249 11.142198 2.289877

H 15.582895 12.180500 2.533397

C 16.363022 10.176703 2.382949

C 16.059027 8.857150 2.029183

H 16.842524 8.099378 2.059987

C 14.772569 8.476995 1.640703

C 12.995482 11.867475 1.764164

H 13.312786 12.800966 2.236741

H 12.050810 11.553099 2.218532

H 12.790906 12.076511 0.705588

C 17.732599 10.545464 2.873818

H 18.498649 9.877577 2.465359

H 17.777241 10.466734 3.969059

H 17.988496 11.577590 2.610475

C 14.457718 7.059201 1.264984

H 13.815712 6.576171 2.014200

H 15.374139 6.467409 1.183560

H 13.924371 7.009210 0.307882

C 13.760343 5.570679 6.360123

C 15.067356 5.923895 6.006572

C 15.907378 6.382897 7.027297

H 16.929599 6.665830 6.774112

C 15.480330 6.473415 8.354161

C 14.169136 6.091947 8.661514

H 13.817397 6.163274 9.691086

C 13.284078 5.648085 7.679385

C 15.562147 5.837405 4.593836

H 15.070186 5.037664 4.030574

H 16.642688 5.663236 4.574613

H 15.369734 6.778676 4.063031

C 16.390458 7.005118 9.422840

H 16.215445 6.504813 10.381817

H 16.210373 8.078268 9.577288

H 17.443964 6.882999 9.150019

C 11.860907 5.307869 8.012269

H 11.163942 5.904729 7.408393

H 11.655163 5.507942 9.067828

H 11.634356 4.252151 7.816733

C 11.778272 10.559794 6.018145

C 12.887606 10.323682 6.838132

C 13.932197 11.250897 6.795538

H 14.810832 11.077233 7.417724

C 13.880359 12.384870 5.979270

C 12.730329 12.602551 5.211979

H 12.663626 13.494252 4.588582

C 11.664832 11.700239 5.210493

C 12.967233 9.104658 7.701974

H 12.039866 8.943058 8.264668

H 13.796253 9.179859 8.411587

H 13.133445 8.209048 7.092401

C 15.041817 13.332606 5.900806

H 14.707253 14.364416 5.745735

H 15.689871 13.068251 5.053272

H 15.653646 13.294642 6.808517

C 10.440100 11.937447 4.376069

H 9.550240 12.076941 5.003163

H 10.233788 11.085394 3.714232

H 10.560142 12.831936 3.758058

107

**I ∙2PF_6_ (PBE, GP)**

C -0.829155 4.122896 -1.949232

C -2.166965 4.149320 -2.212711

N -2.750668 3.267351 -1.317125

C -1.829271 2.717302 -0.478109

N -0.629054 3.258288 -0.868653

C -5.067160 -1.065284 -1.007225

C -3.999717 -1.921074 -1.046757

C -4.187773 3.069639 -1.179551

N -4.577508 0.135860 -0.526813

C -3.246961 0.057928 -0.221901

C -0.477833 4.973627 3.428547

C -5.391416 1.256036 -0.082428

N -2.896452 -1.217982 -0.568090

C -1.592757 -1.812360 -0.459600

C -2.923780 2.658295 2.293660

C -4.397250 3.827081 3.546833

C -3.291631 3.585067 4.318482

N -4.523058 2.421083 0.097999

C -5.008757 3.332421 1.153056

Fe -2.435272 1.484942 0.845252

N -4.144290 3.248051 2.319960

C -1.193703 -2.366501 0.775532

N -2.393897 2.868876 3.532964

C 0.091833 -2.939610 0.830316

C 0.933928 -3.007715 -0.293688

C 0.465064 -2.488019 -1.516253

C -0.792071 -1.872648 -1.621773

C -2.117474 -2.410058 1.962159

C 2.321283 -3.591645 -0.189448

C -1.269096 -1.273326 -2.920826

C 2.406069 4.352656 0.998794

C 1.155228 4.407063 0.363306

C 0.647798 3.201784 -0.195148

C 1.402545 2.009443 -0.187150

C 2.644527 2.016180 0.477249

C 3.163184 3.169764 1.082362

C 0.436529 5.726894 0.257613

C 0.949192 0.759174 -0.883109

C 4.503563 3.165436 1.774387

C 1.242054 3.254241 4.161846

C -0.085238 3.575388 3.827517

C -1.048438 2.546927 3.927360

C -0.741364 1.254559 4.409233

C 0.604393 0.991661 4.720297

C 1.610296 1.965631 4.588654

C 3.050462 1.624451 4.882083

C -1.805126 0.215410 4.621487

N -1.124305 0.895139 1.348589

H -0.002088 4.666574 -2.400874

H -2.748217 4.755006 -2.906037

H -6.118856 -1.218377 -1.246721

H -3.921833 -2.962963 -1.355300

H -4.542196 2.426738 -2.005645

H -4.650205 4.069877 -1.247428

H -1.042117 5.472569 4.238892

H 0.415926 5.585047 3.229662

H -1.132046 5.015029 2.542419

H -5.847690 0.997727 0.886021

H -6.190692 1.434731 -0.831240

H -5.317739 4.366197 3.761783

H -3.062505 3.852505 5.348796

H -6.025207 3.048126 1.470220

H -4.996522 4.372125 0.791249

H 0.435656 -3.358105 1.785475

H 1.106479 -2.539384 -2.406274

H -2.787517 -3.288918 1.898480

H -1.548561 -2.501262 2.900929

H -2.775128 -1.533288 2.047059

H 2.405587 -4.291392 0.659004

H 2.607704 -4.127789 -1.110837

H 3.069603 -2.791176 -0.031767

H -0.467455 -1.281566 -3.676771

H -2.131717 -1.826696 -3.336630

H -1.600462 -0.227963 -2.781060

H 2.801160 5.277269 1.441191

H 3.230304 1.087135 0.496803

H 0.751541 6.409998 1.061898

H 0.671481 6.234028 -0.697275

H -0.655755 5.638616 0.302025

H 0.230459 0.971370 -1.690555

H 1.808744 0.219418 -1.315158

H 0.450837 0.077338 -0.173090

H 4.967248 2.164802 1.758473

H 5.204191 3.871558 1.291769

H 4.408580 3.477289 2.830436

H 2.006843 4.037755 4.081819

H 0.868727 -0.010323 5.084369

H 3.135040 0.879017 5.691810

H 3.537866 1.193310 3.986744

H 3.629900 2.517223 5.174123

H -2.534788 0.528821 5.388136

H -2.406877 0.041780 3.718636

H -1.359877 -0.741038 4.939448

P -5.449626 -0.209989 3.315594

F -6.728704 -0.636563 4.195741

F -6.013635 -1.026398 2.000514

F -4.589915 -1.511175 3.787539

F -4.825908 0.690919 4.524711

F -6.246867 1.151273 2.762832

F -4.123122 0.247161 2.346920

P -3.363748 6.613903 0.560147

F -4.045558 8.063411 0.364592

F -4.483224 5.932877 -0.492391

F -4.382256 6.259550 1.806614

F -2.249456 7.195400 1.594633

F -2.365108 6.855472 -0.715963

F -2.693207 5.083102 0.745650

107

**I∙2PF_6_ (PBE, SMD=MeCN)**

C -1.196896 4.737241 -2.680594

C -2.537876 4.530469 -2.880614

N -2.932033 3.628774 -1.914876

C -1.885956 3.234241 -1.128911

N -0.812869 3.943996 -1.603738

C -4.865668 -1.212747 -0.807233

C -3.786748 -2.036415 -0.591895

C -4.238873 2.998288 -1.817808

N -4.435348 0.067330 -0.530084

C -3.126665 0.084904 -0.157309

C 0.154163 3.644858 2.542411

C -5.259538 1.264218 -0.448407

N -2.731789 -1.225467 -0.191980

C -1.432286 -1.689397 0.208814

C -2.799124 2.436097 1.750032

C -4.310884 3.512174 3.059899

C -3.305932 3.060274 3.870297

N -4.376996 2.439148 -0.469547

C -4.700834 3.445382 0.553309

Fe -2.161110 1.731483 0.081500

N -3.971310 3.127328 1.772799

C -1.265217 -2.143731 1.534918

N -2.384784 2.395645 3.055759

C 0.038975 -2.484304 1.941358

C 1.139752 -2.394165 1.066357

C 0.906566 -2.006002 -0.268948

C -0.376704 -1.657399 -0.728290

C -2.442125 -2.297151 2.460264

C 2.537050 -2.686758 1.550964

C -0.637272 -1.283989 -2.159137

C 2.112544 5.309109 0.243411

C 0.817260 5.192194 -0.285194

C 0.507624 4.027470 -1.032607

C 1.458480 3.011744 -1.268833

C 2.735868 3.170928 -0.691698

C 3.084085 4.303001 0.064518

C -0.217711 6.256892 -0.027389

C 1.166620 1.791256 -2.098442

C 4.443364 4.424518 0.704907

C 1.216787 1.639012 3.661873

C 0.058034 2.324808 3.250658

C -1.197295 1.751703 3.554444

C -1.313518 0.604302 4.376832

C -0.123568 -0.038420 4.766688

C 1.149013 0.441161 4.396943

C 2.396676 -0.294781 4.815702

C -2.643286 0.111751 4.879036

N -0.771851 1.259184 0.481925

H -0.484641 5.379033 -3.200022

H -3.224037 4.926345 -3.629195

H -5.886123 -1.434915 -1.119205

H -3.675608 -3.116224 -0.695313

H -4.286909 2.171528 -2.541158

H -5.017211 3.746256 -2.056671

H -0.498032 4.402874 3.013923

H 1.188978 4.020699 2.552951

H -0.160924 3.561229 1.489859

H -5.815433 1.241892 0.499497

H -5.974773 1.267045 -1.289420

H -5.228460 4.056313 3.284012

H -3.162248 3.139158 4.947694

H -5.779061 3.473657 0.787041

H -4.384331 4.436034 0.185077

H 0.196280 -2.826481 2.972917

H 1.745895 -1.973391 -0.976748

H -3.049121 -3.177551 2.172985

H -2.105788 -2.452902 3.497350

H -3.126033 -1.433592 2.436541

H 2.535706 -3.402632 2.391066

H 3.170581 -3.093808 0.743555

H 3.024632 -1.759607 1.910436

H 0.282812 -1.351927 -2.761960

H -1.393875 -1.946735 -2.615425

H -1.035231 -0.261824 -2.263742

H 2.360547 6.201032 0.834328

H 3.483662 2.383256 -0.856918

H 0.120818 6.941350 0.767785

H -0.416364 6.864189 -0.930383

H -1.183833 5.817987 0.280992

H 0.245517 1.880742 -2.691829

H 2.007305 1.582943 -2.783994

H 1.056372 0.905980 -1.446518

H 5.191732 3.793728 0.195320

H 4.801975 5.468893 0.702251

H 4.403609 4.099561 1.763085

H 2.197105 2.064745 3.407474

H -0.199776 -0.943996 5.383759

H 2.523839 -0.271322 5.914679

H 2.347798 -1.359800 4.524124

H 3.299834 0.146201 4.361093

H -2.984758 0.729939 5.731408

H -3.431955 0.165711 4.118442

H -2.566726 -0.926530 5.240539

P -2.769784 0.892458 -4.683408

F -1.886917 1.519481 -5.905753

F -1.875560 -0.479183 -4.720393

F -3.837015 0.245597 -5.734636

F -3.665974 2.277926 -4.623187

F -1.708738 1.552138 -3.587734

F -3.646669 0.288235 -3.412444

P -6.155986 -0.024746 2.998356

F -7.633015 -0.336551 3.614758

F -6.581264 -0.745154 1.580694

F -5.578704 -1.450097 3.562860

F -5.712347 0.723557 4.387182

F -6.693883 1.425436 2.401265

F -4.657009 0.301641 2.352147

94

**1 (Hopt)**

Fe 8.339095 9.856711 4.991475

F 8.442237 11.717851 4.875958

N 6.625813 10.191433 3.416570

N 8.712749 10.512354 2.260867

N 10.681630 10.106487 2.995353

N 6.554021 7.859492 3.928114

N 8.013061 6.795486 5.078428

N 5.748797 10.907611 5.473791

N 6.503239 10.486271 7.437028

N 9.375216 9.374108 5.991129

C 7.264594 10.678904 2.182387

H 6.889839 10.136415 1.295991

H 7.047611 11.751650 2.039825

C 9.377074 10.013708 3.317100

C 9.578081 10.910991 1.273275

H 9.243504 11.342281 0.328588

C 10.804322 10.650956 1.723637

H 11.780678 10.820864 1.271037

C 5.870082 8.949254 3.236574

H 4.854893 9.056472 3.660607

H 5.767118 8.683764 2.169089

C 7.676923 8.034950 4.654719

C 6.186254 6.526086 3.892707

H 5.302240 6.174961 3.357837

C 7.093188 5.860405 4.613095

H 7.203052 4.797735 4.831934

C 5.812921 11.223420 4.053458

H 6.326592 12.191953 3.939348

H 4.786835 11.289737 3.642211

C 6.784364 10.340078 6.125582

C 4.813651 11.406272 6.370550

H 3.901376 11.910530 6.048196

C 5.283652 11.142477 7.595491

H 4.879206 11.354664 8.585297

C 11.826717 9.630972 3.756157

C 12.181748 10.264769 4.941437

C 13.249467 9.724236 5.662827

H 13.533400 10.195137 6.612827

C 13.964324 8.622451 5.200230

C 13.645021 8.093728 3.964386

H 14.238372 7.265623 3.556156

C 12.586955 8.597911 3.207594

C 11.530414 11.526234 5.441790

H 12.246755 12.365307 5.363221

H 11.270754 11.434799 6.510555

H 10.618690 11.815321 4.900910

C 15.068880 8.015099 6.045467

H 15.802348 8.780415 6.355178

H 15.611154 7.223541 5.503725

H 14.651823 7.575660 6.969374

C 12.355037 8.048907 1.820834

H 12.955123 8.610114 1.079062

H 11.307138 8.090079 1.487674

H 12.693064 7.001930 1.753446

C 9.344881 6.375981 5.577650

C 10.271771 6.061921 4.594514

C 11.522211 5.586015 5.015703

H 12.278563 5.352857 4.256692

C 11.804193 5.382300 6.351309

C 10.826309 5.638243 7.299158

H 11.031939 5.452046 8.360943

C 9.564463 6.139678 6.940525

C 9.953119 6.203693 3.133889

H 9.077387 5.593440 2.845577

H 10.796604 5.871033 2.513360

H 9.721172 7.250419 2.865208

C 13.150542 4.859268 6.780844

H 13.482514 5.318864 7.727134

H 13.923008 5.036915 6.015266

H 13.105934 3.767167 6.954109

C 8.492143 6.403014 7.953149

H 8.267249 7.481751 8.016828

H 8.802095 6.073368 8.957216

H 7.549896 5.883367 7.702051

C 7.321042 10.145617 8.583715

C 6.917513 9.084699 9.399925

C 7.643280 8.849787 10.556282

H 7.342279 8.015341 11.203488

C 8.734539 9.625051 10.914113

C 9.100502 10.682319 10.089397

H 9.951429 11.317623 10.366310

C 8.396532 10.978875 8.925365

C 5.719383 8.236927 9.060111

H 4.774933 8.729064 9.360336

H 5.757193 7.272598 9.591871

H 5.644900 8.027245 7.978293

C 9.491200 9.341457 12.195327

H 9.034909 9.891586 13.039956

H 10.543423 9.663388 12.127238

H 9.466369 8.269935 12.454294

C 8.748144 12.206668 8.125113

H 8.062125 13.037766 8.380835

H 8.684334 12.065612 7.033499

H 9.765586 12.551341 8.369870

67

**1 (Hopt, trunc)**

Fe 0.000000 0.000000 0.000000

F 0.000000 1.769522 -0.597172

N -0.019085 -0.425332 -2.312237

N 2.206864 0.298671 -1.751057

N 3.022233 0.598707 0.204914

N -0.250548 -2.568353 -1.283325

N -0.029194 -2.971820 0.807691

N -2.182044 0.320807 -1.783941

N -3.085703 0.448567 0.157218

N 0.000000 0.000000 1.518475

C 1.278145 0.025784 -2.843797

H 1.725043 -0.732975 -3.511602

H 1.149923 0.953807 -3.428023

C 1.916820 0.185109 -0.443937

C 3.482087 0.769948 -1.937463

H 3.919211 0.934732 -2.923725

C 3.995724 0.948849 -0.721619

H 4.975353 1.310183 -0.406007

C -0.291177 -1.844911 -2.551615

H -1.292886 -1.972748 -3.002170

H 0.447183 -2.293991 -3.240721

C -0.045804 -1.964601 -0.094537

C -0.360949 -3.939453 -1.133795

H -0.531081 -4.612943 -1.975741

C -0.226930 -4.193479 0.170866

H -0.225900 -5.133167 0.724484

C -1.119521 0.414757 -2.775607

H -0.772232 1.460567 -2.809189

H -1.511837 0.118359 -3.768463

C -1.919278 0.185849 -0.467869

C -3.511693 0.662649 -1.990151

H -3.931714 0.826030 -2.984036

C -4.075932 0.741304 -0.779197

H -5.095648 0.977344 -0.473070

C 3.270678 0.599365 1.638252

C 2.583993 1.486412 2.459377

C 2.814882 1.397156 3.834632

H 2.269057 2.072389 4.505677

C 3.726943 0.490193 4.368030

C 4.466137 -0.295227 3.504603

H 5.236420 -0.968031 3.903272

C 4.270819 -0.243072 2.124181

C 0.532610 -2.905419 2.178428

C 1.904998 -3.088838 2.263467

C 2.474262 -3.111424 3.545227

H 3.558531 -3.247929 3.638004

C 1.697280 -3.012924 4.681654

C 0.321443 -2.904650 4.557062

H -0.304293 -2.859479 5.457330

C -0.303651 -2.851420 3.300578

C -3.352347 0.542709 1.578398

C -4.136319 -0.447734 2.177576

C -4.480663 -0.278757 3.508720

H -5.102789 -1.044105 3.991498

C -4.075409 0.824528 4.242511

C -3.310119 1.799350 3.613268

H -3.000723 2.691119 4.173389

C -2.950582 1.693687 2.272343

H -1.390025 -2.763020 3.198375

H 2.158267 -3.053737 5.675279

H 2.519511 -3.195788 1.363237

H 1.875605 2.209411 2.039966

H 3.888886 0.439329 5.450656

H 4.844506 -0.884994 1.445654

H -2.377183 2.479053 1.766595

H -4.367497 0.935638 5.292742

H -4.473878 -1.320336 1.606166

94

**1 (PBE)**

Fe 8.420279 9.813132 5.027965

F 8.538585 11.649567 4.889112

N 6.491931 10.202467 3.358679

N 8.636064 10.380413 2.208188

N 10.639172 10.121716 3.021412

N 6.459244 7.897384 4.014216

N 8.061166 6.801157 4.982345

N 5.868487 11.099640 5.459707

N 6.585078 10.563358 7.434750

N 9.515669 9.297188 5.919811

C 7.168052 10.437237 2.089338

H 6.850432 9.676835 1.353172

H 6.905458 11.428798 1.677332

C 9.317126 9.973089 3.312822

C 9.520724 10.785562 1.212488

H 9.191215 11.148661 0.237964

C 10.779285 10.618498 1.727090

H 11.759608 10.809653 1.290233

C 5.672366 9.003899 3.466567

H 4.819689 9.206367 4.138853

H 5.264550 8.683234 2.488803

C 7.654509 8.063038 4.647704

C 6.085066 6.558017 3.985223

H 5.153428 6.212587 3.535071

C 7.094581 5.870869 4.601639

H 7.220839 4.805105 4.793020

C 5.921142 11.373583 4.027718

H 6.590406 12.231383 3.849078

H 4.896640 11.616086 3.680390

C 6.853392 10.408065 6.113775

C 4.988488 11.686622 6.361453

H 4.133239 12.287590 6.049386

C 5.441646 11.346134 7.606509

H 5.061366 11.586526 8.599610

C 11.751152 9.747260 3.868222

C 12.141128 10.585662 4.943643

C 13.188451 10.128709 5.764432

H 13.501030 10.759984 6.606399

C 13.873824 8.925810 5.517441

C 13.510734 8.179778 4.379212

H 14.072716 7.270227 4.131560

C 12.460514 8.569876 3.532711

C 11.587655 11.971672 5.143179

H 12.177175 12.691614 4.543306

H 11.683386 12.282320 6.196202

H 10.533578 12.073620 4.848617

C 14.971954 8.459777 6.435884

H 15.563169 9.306196 6.824336

H 15.659176 7.760468 5.931844

H 14.545838 7.933193 7.311056

C 12.170701 7.793828 2.274268

H 12.695358 8.241106 1.408648

H 11.098011 7.766409 2.024719

H 12.532120 6.757446 2.360922

C 9.383372 6.403356 5.410928

C 10.211329 5.789618 4.433824

C 11.482678 5.365566 4.840165

H 12.133395 4.875512 4.104106

C 11.947046 5.531512 6.164972

C 11.082964 6.121693 7.098681

H 11.410317 6.231908 8.140563

C 9.779975 6.539074 6.759884

C 9.731235 5.544002 3.024185

H 9.018915 4.699816 2.979122

H 10.570643 5.283842 2.361863

H 9.215922 6.422808 2.596302

C 13.308585 5.029213 6.563392

H 13.627618 5.432114 7.538129

H 14.073496 5.288570 5.811120

H 13.304202 3.926016 6.645972

C 8.857029 7.040486 7.832110

H 9.247052 7.962675 8.296491

H 8.764922 6.291593 8.638443

H 7.845974 7.252193 7.455175

C 7.330388 10.053580 8.562104

C 6.759150 8.993159 9.303779

C 7.447072 8.560401 10.447949

H 7.024548 7.736146 11.036996

C 8.667236 9.141995 10.851201

C 9.194499 10.189890 10.073143

H 10.137591 10.660880 10.379037

C 8.536842 10.688557 8.931742

C 5.471187 8.333871 8.875426

H 4.589809 8.965558 9.092790

H 5.323277 7.380164 9.406482

H 5.456271 8.125142 7.789511

C 9.367835 8.672412 12.098868

H 8.934243 9.163224 12.990802

H 10.442803 8.916037 12.082317

H 9.256070 7.584603 12.245252

C 9.071333 11.901852 8.213576

H 8.594411 12.818465 8.610922

H 8.893943 11.891086 7.126111

H 10.154927 12.005800 8.385104

94

**1 (PBE, CPCM=MeCN)**

Fe 8.368086 9.865116 5.011531

F 8.527795 11.704010 4.859588

N 6.562158 10.188081 3.431925

N 8.644168 10.357830 2.217626

N 10.641226 10.120699 3.042828

N 6.460282 7.904754 4.090367

N 8.076804 6.824547 5.042990

N 5.867037 11.177212 5.439141

N 6.562579 10.624676 7.412983

N 9.438636 9.390864 5.959915

C 7.179819 10.379138 2.113768

H 6.867592 9.571117 1.431455

H 6.879605 11.342746 1.667950

C 9.316302 9.977308 3.329068

C 9.527120 10.737416 1.214862

H 9.188636 11.072787 0.233673

C 10.786056 10.584632 1.734879

H 11.769020 10.754426 1.294909

C 5.687180 9.013578 3.547127

H 4.849147 9.237882 4.228685

H 5.268046 8.708420 2.572230

C 7.659475 8.079885 4.697811

C 6.084801 6.569495 4.080641

H 5.144021 6.229766 3.645994

C 7.103860 5.888028 4.687489

H 7.240477 4.824075 4.881766

C 5.927024 11.386901 3.998039

H 6.561125 12.259356 3.776711

H 4.904117 11.554944 3.612071

C 6.832535 10.472805 6.091774

C 4.982840 11.763046 6.331655

H 4.131567 12.361266 6.004408

C 5.422644 11.415693 7.581280

H 5.032901 11.647263 8.572898

C 11.744537 9.751162 3.897125

C 12.124466 10.588904 4.974681

C 13.151078 10.128229 5.820607

H 13.444071 10.755956 6.672866

C 13.824890 8.913852 5.594580

C 13.481761 8.167758 4.451063

H 14.028368 7.242947 4.225897

C 12.450626 8.566026 3.582510

C 11.560298 11.971866 5.165180

H 12.079765 12.677749 4.488190

H 11.728392 12.319089 6.197794

H 10.484239 12.034235 4.943148

C 14.881154 8.426391 6.552976

H 15.430506 9.266748 7.011103

H 15.603571 7.757777 6.054941

H 14.416647 7.850884 7.376871

C 12.165549 7.782522 2.328592

H 12.676557 8.243622 1.461862

H 11.091210 7.749078 2.088652

H 12.536767 6.749815 2.420281

C 9.404353 6.418062 5.437016

C 10.202215 5.797502 4.438638

C 11.473746 5.342113 4.813116

H 12.103532 4.861216 4.053135

C 11.965084 5.483607 6.129426

C 11.131939 6.080035 7.088257

H 11.482967 6.174553 8.124650

C 9.831989 6.531244 6.776651

C 9.689688 5.582251 3.036485

H 8.927533 4.781956 3.009825

H 10.508673 5.280850 2.365370

H 9.214610 6.490005 2.623324

C 13.332030 4.962989 6.491592

H 13.639298 5.292114 7.497981

H 14.092879 5.301530 5.766103

H 13.344133 3.856788 6.476283

C 8.943865 7.062334 7.863816

H 9.284019 8.055268 8.206832

H 8.969601 6.394469 8.742162

H 7.898451 7.161639 7.536668

C 7.312343 10.103360 8.525871

C 6.764222 9.017460 9.244666

C 7.469987 8.563170 10.371279

H 7.065127 7.715682 10.939980

C 8.693683 9.140552 10.765456

C 9.198075 10.220695 10.016233

H 10.143919 10.690048 10.317704

C 8.517340 10.739854 8.897620

C 5.486565 8.352826 8.799361

H 4.608222 9.006190 8.955969

H 5.317103 7.419420 9.359622

H 5.516931 8.110802 7.720702

C 9.464600 8.583192 11.935107

H 8.789258 8.194344 12.716504

H 10.126751 9.342721 12.384227

H 10.103950 7.740532 11.607479

C 9.029108 11.967287 8.187212

H 8.472350 12.862299 8.526277

H 8.912936 11.916530 7.091745

H 10.094491 12.130947 8.417262

94

**1 (TPSSh)**

Fe 8.360899 9.837607 5.000518

F 8.485808 11.647780 4.854471

N 6.561307 10.203369 3.443442

N 8.639104 10.369076 2.218307

N 10.626587 10.103670 3.032983

N 6.469333 7.898173 4.009058

N 8.078231 6.799183 4.931599

N 5.863182 11.125334 5.481868

N 6.581778 10.564695 7.436419

N 9.423582 9.368076 5.932144

C 7.178178 10.434233 2.131123

H 6.834116 9.674018 1.422085

H 6.903038 11.419640 1.740298

C 9.312288 9.966437 3.319286

C 9.525183 10.761867 1.220944

H 9.200119 11.117001 0.251680

C 10.773719 10.591235 1.737679

H 11.749741 10.771173 1.308022

C 5.688080 9.028945 3.529952

H 4.881013 9.237890 4.239490

H 5.244880 8.767156 2.561763

C 7.660654 8.055190 4.633687

C 6.103835 6.559662 3.946754

H 5.177826 6.223102 3.499178

C 7.118701 5.871359 4.535474

H 7.255282 4.811640 4.702375

C 5.935706 11.386852 4.052495

H 6.582365 12.248238 3.871604

H 4.923811 11.576732 3.669844

C 6.832115 10.413435 6.119689

C 5.004581 11.726478 6.391718

H 4.166617 12.341854 6.091241

C 5.458663 11.369303 7.623442

H 5.096971 11.608941 8.614230

C 11.732584 9.739221 3.893159

C 12.109062 10.588848 4.954124

C 13.150307 10.150214 5.783075

H 13.453099 10.787859 6.612606

C 13.840217 8.952241 5.551995

C 13.489042 8.192397 4.426240

H 14.051893 7.289865 4.193643

C 12.443771 8.566898 3.575706

C 11.541723 11.973408 5.130126

H 12.091173 12.673142 4.485071

H 11.675241 12.313172 6.160922

H 10.483029 12.042819 4.874802

C 14.939256 8.504279 6.480081

H 15.525382 9.355036 6.842966

H 15.618487 7.801295 5.988288

H 14.513388 8.000294 7.357999

C 12.161033 7.778917 2.322956

H 12.671935 8.229629 1.461381

H 11.093889 7.737655 2.086846

H 12.536059 6.756943 2.418067

C 9.395566 6.402043 5.378193

C 10.227055 5.769272 4.427091

C 11.488439 5.351736 4.853776

H 12.141616 4.850215 4.140665

C 11.935663 5.548403 6.173732

C 11.066310 6.156923 7.082762

H 11.380810 6.289903 8.116933

C 9.772568 6.565223 6.720558

C 9.762916 5.493427 3.016763

H 9.075126 4.638960 2.983788

H 10.610130 5.242883 2.374197

H 9.239447 6.351782 2.578116

C 13.294804 5.058432 6.600194

H 13.600184 5.500324 7.552900

H 14.056662 5.290006 5.847928

H 13.283596 3.967793 6.725508

C 8.831144 7.079863 7.771656

H 9.239006 7.967565 8.266683

H 8.679343 6.316338 8.543597

H 7.852955 7.337449 7.360303

C 7.339071 10.039359 8.548899

C 6.764023 8.995555 9.298602

C 7.465035 8.547156 10.421993

H 7.041402 7.738144 11.015343

C 8.702950 9.099291 10.793578

C 9.233946 10.132372 10.008532

H 10.187468 10.577473 10.289062

C 8.561974 10.644114 8.887755

C 5.451639 8.366314 8.899193

H 4.599089 9.010680 9.150005

H 5.306686 7.416317 9.420756

H 5.408844 8.173822 7.819439

C 9.424308 8.610650 12.023203

H 9.019575 9.100813 12.918717

H 10.494006 8.836838 11.977576

H 9.299204 7.530977 12.157918

C 9.109781 11.844280 8.156926

H 8.682966 12.764814 8.578231

H 8.889026 11.840891 7.085894

H 10.194498 11.900925 8.284678

94

**1 (TPSSh, CPCM=MeCN)**

Fe 8.348950 9.881738 5.014536

F 8.500228 11.696519 4.850857

N 6.593086 10.197153 3.470001

N 8.653797 10.349978 2.235030

N 10.639168 10.098645 3.055437

N 6.469604 7.914568 4.092725

N 8.085007 6.830711 5.016756

N 5.859281 11.180205 5.453293

N 6.542026 10.612502 7.416099

N 9.389948 9.430200 5.982300

C 7.191404 10.389623 2.139222

H 6.866561 9.593083 1.465442

H 6.900607 11.354653 1.715642

C 9.320621 9.970445 3.341577

C 9.538004 10.715575 1.230375

H 9.202929 11.046104 0.255990

C 10.788128 10.555619 1.747586

H 11.765495 10.714122 1.312362

C 5.698243 9.033301 3.574004

H 4.883681 9.260211 4.267241

H 5.274341 8.751823 2.605507

C 7.663541 8.081624 4.695863

C 6.099815 6.580097 4.059659

H 5.165920 6.248420 3.625642

C 7.117794 5.897051 4.648507

H 7.259512 4.839776 4.826636

C 5.935404 11.396346 4.014425

H 6.560229 12.265511 3.803754

H 4.925320 11.541725 3.614756

C 6.814823 10.472752 6.101843

C 4.973197 11.759281 6.345860

H 4.131436 12.356820 6.021714

C 5.404483 11.402330 7.587097

H 5.016470 11.624825 8.571957

C 11.739649 9.728744 3.913710

C 12.108182 10.559403 4.991206

C 13.135498 10.107406 5.831955

H 13.419158 10.728383 6.681347

C 13.820421 8.907020 5.596599

C 13.484245 8.164224 4.455611

H 14.034727 7.253008 4.226363

C 12.450958 8.556098 3.595345

C 11.529718 11.935745 5.190616

H 12.056403 12.646511 4.538033

H 11.674636 12.262023 6.224888

H 10.466147 11.988915 4.950145

C 14.888808 8.429732 6.547945

H 15.439260 9.272304 6.980942

H 15.598115 7.762430 6.047306

H 14.435307 7.870115 7.377404

C 12.171062 7.775560 2.337963

H 12.682687 8.239378 1.483475

H 11.104330 7.744011 2.101666

H 12.542272 6.751658 2.431586

C 9.409540 6.425958 5.422218

C 10.210244 5.802746 4.438225

C 11.474954 5.352645 4.822791

H 12.106489 4.871186 4.077121

C 11.952972 5.506503 6.136711

C 11.117050 6.108750 7.081729

H 11.458680 6.213550 8.111209

C 9.823462 6.552873 6.756587

C 9.707036 5.577697 3.033490

H 8.954972 4.778933 3.010951

H 10.528722 5.277201 2.378500

H 9.237975 6.477899 2.618810

C 13.316717 4.987678 6.516856

H 13.627074 5.365253 7.496008

H 14.069069 5.277709 5.774604

H 13.309060 3.890282 6.561769

C 8.921808 7.090222 7.830557

H 9.269085 8.068141 8.183264

H 8.919317 6.415318 8.693659

H 7.893962 7.202436 7.479129

C 7.305016 10.093503 8.520755

C 6.770149 9.014167 9.246691

C 7.491992 8.561146 10.356559

H 7.099904 7.720979 10.928580

C 8.719386 9.136129 10.724946

C 9.210422 10.210845 9.970016

H 10.155139 10.675177 10.250811

C 8.510622 10.727055 8.868241

C 5.482900 8.351731 8.824474

H 4.617202 9.000520 9.010102

H 5.333820 7.420629 9.378561

H 5.493872 8.121904 7.751505

C 9.508638 8.578791 11.883148

H 8.849794 8.295804 12.711849

H 10.244587 9.302394 12.248644

H 10.051332 7.675573 11.571399

C 9.009006 11.955388 8.149319

H 8.456413 12.840152 8.495095

H 8.878038 11.895965 7.064492

H 10.069646 12.115969 8.363384

94

**1 (PBE0)**

Fe 8.370243 9.838314 5.018761

F 8.491066 11.638239 4.878924

N 6.561870 10.207330 3.433530

N 8.653701 10.362506 2.246202

N 10.630899 10.095560 3.059038

N 6.488119 7.911609 4.012347

N 8.078358 6.809279 4.940606

N 5.867010 11.097786 5.479011

N 6.581737 10.550581 7.427769

N 9.400359 9.374398 5.950746

C 7.202892 10.456102 2.151869

H 6.853378 9.728301 1.409801

H 6.951645 11.456599 1.779218

C 9.322804 9.965390 3.342483

C 9.536506 10.746769 1.253509

H 9.215633 11.100954 0.280954

C 10.780660 10.575685 1.770073

H 11.757864 10.752270 1.338027

C 5.717607 9.027917 3.506422

H 4.879994 9.224473 4.185544

H 5.301536 8.751102 2.528585

C 7.670326 8.060164 4.639234

C 6.115147 6.583442 3.953307

H 5.189040 6.248680 3.501226

C 7.119308 5.892050 4.547353

H 7.246869 4.829981 4.715732

C 5.937855 11.366624 4.059521

H 6.574559 12.239262 3.888284

H 4.923593 11.562456 3.681387

C 6.834333 10.402257 6.119131

C 5.003339 11.687371 6.380105

H 4.158595 12.295334 6.078586

C 5.456069 11.338533 7.610542

H 5.086614 11.577801 8.600160

C 11.731295 9.739859 3.915737

C 12.113286 10.597661 4.962264

C 13.145769 10.163133 5.795601

H 13.452769 10.808680 6.618519

C 13.822159 8.958208 5.585136

C 13.468648 8.189934 4.473076

H 14.022328 7.276963 4.257041

C 12.433253 8.562490 3.616423

C 11.551850 11.978674 5.126651

H 12.103538 12.675553 4.480304

H 11.683868 12.328220 6.154883

H 10.493377 12.056843 4.871682

C 14.907658 8.516646 6.519839

H 15.585638 9.342377 6.763074

H 15.501750 7.702062 6.094754

H 14.478729 8.161455 7.466239

C 12.152384 7.765860 2.377773

H 12.669811 8.201630 1.512195

H 11.087068 7.724100 2.133212

H 12.521177 6.742536 2.483615

C 9.387728 6.394127 5.369939

C 10.197866 5.763459 4.405037

C 11.454834 5.324358 4.809248

H 12.092829 4.822459 4.081699

C 11.920607 5.499075 6.119232

C 11.070418 6.101946 7.044411

H 11.399656 6.218376 8.076937

C 9.783072 6.531121 6.705401

C 9.720107 5.513833 3.002035

H 9.028287 4.662995 2.958368

H 10.557893 5.268642 2.344565

H 9.197055 6.379111 2.576315

C 13.273531 4.997283 6.523856

H 13.677446 5.562758 7.369231

H 13.989802 5.049968 5.697125

H 13.213830 3.944760 6.831949

C 8.874224 7.056437 7.770135

H 9.271736 7.974186 8.218445

H 8.776632 6.322251 8.578323

H 7.870065 7.269598 7.396783

C 7.342225 10.045536 8.535653

C 6.797712 8.984606 9.275749

C 7.501715 8.555781 10.399327

H 7.098166 7.732935 10.989077

C 8.714485 9.142785 10.781091

C 9.216249 10.192125 10.006303

H 10.152972 10.666965 10.297414

C 8.538900 10.685875 8.887045

C 5.511426 8.323730 8.870686

H 4.639082 8.944964 9.112326

H 5.383203 7.372302 9.394508

H 5.476617 8.123461 7.791995

C 9.436945 8.678998 12.009936

H 9.041398 9.189957 12.898079

H 10.507843 8.898928 11.957178

H 9.309629 7.603512 12.172256

C 9.050332 11.902522 8.171519

H 8.576756 12.805448 8.581335

H 8.859355 11.897750 7.094550

H 10.128187 12.009877 8.325500

94

**1 (PBE0, CPCM=MeCN)**

Fe 8.364162 9.868964 5.030965

F 8.524392 11.675149 4.888682

N 6.596489 10.191529 3.468194

N 8.661134 10.342311 2.260314

N 10.639776 10.096099 3.070329

N 6.488226 7.919024 4.095369

N 8.087174 6.830214 5.019454

N 5.879189 11.156787 5.455744

N 6.553305 10.598802 7.412842

N 9.380842 9.412624 5.986858

C 7.208449 10.390990 2.160494

H 6.882852 9.608168 1.468445

H 6.926659 11.360827 1.736960

C 9.329282 9.965381 3.358264

C 9.536077 10.710484 1.259229

H 9.198717 11.043766 0.284899

C 10.784352 10.553862 1.770982

H 11.760294 10.717545 1.330774

C 5.724043 9.027684 3.567322

H 4.891745 9.244094 4.245056

H 5.308611 8.737090 2.595833

C 7.674221 8.076648 4.699519

C 6.112336 6.594394 4.061154

H 5.177904 6.265338 3.622549

C 7.121476 5.907248 4.651422

H 7.254454 4.846875 4.826635

C 5.950950 11.374489 4.026211

H 6.571655 12.249254 3.817764

H 4.938246 11.532428 3.632716

C 6.827255 10.456327 6.106758

C 4.995190 11.735860 6.338654

H 4.152775 12.333670 6.011702

C 5.421939 11.384449 7.578592

H 5.028413 11.611557 8.561867

C 11.742178 9.737280 3.916508

C 12.129275 10.581649 4.970397

C 13.162694 10.140334 5.802019

H 13.461602 10.774103 6.637533

C 13.836277 8.936167 5.579748

C 13.483465 8.179339 4.458924

H 14.027556 7.261241 4.237676

C 12.444595 8.561202 3.608506

C 11.557584 11.955125 5.156297

H 12.054155 12.652483 4.466622

H 11.742074 12.311125 6.174687

H 10.484995 12.005491 4.957342

C 14.905911 8.466838 6.521564

H 15.428445 9.310180 6.986217

H 15.640280 7.834846 6.010533

H 14.464632 7.867447 7.329914

C 12.149028 7.768947 2.371262

H 12.643779 8.223142 1.501673

H 11.079240 7.727823 2.149390

H 12.524870 6.746814 2.467832

C 9.401420 6.416237 5.420577

C 10.197018 5.797089 4.434947

C 11.458424 5.345196 4.811634

H 12.087808 4.866726 4.060958

C 11.940752 5.492594 6.119556

C 11.109057 6.085387 7.067441

H 11.453845 6.184166 8.097314

C 9.819127 6.531516 6.750122

C 9.692870 5.579165 3.037270

H 8.947803 4.773895 3.008916

H 10.512651 5.290431 2.374288

H 9.213616 6.476076 2.626156

C 13.303151 4.983389 6.486910

H 13.610070 5.337518 7.476203

H 14.054466 5.302709 5.755084

H 13.313513 3.885339 6.502020

C 8.931030 7.064830 7.827627

H 9.284080 8.038307 8.187588

H 8.929449 6.386117 8.688083

H 7.899488 7.185811 7.489371

C 7.303493 10.086816 8.518256

C 6.775416 8.999945 9.229070

C 7.482376 8.555385 10.346471

H 7.092010 7.708293 10.910914

C 8.689256 9.146130 10.737792

C 9.174523 10.228531 9.998003

H 10.105639 10.708863 10.299584

C 8.488568 10.736269 8.890038

C 5.509231 8.326718 8.787573

H 4.632878 8.968212 8.947586

H 5.352270 7.398615 9.344850

H 5.540646 8.086445 7.716840

C 9.461418 8.603833 11.904429

H 10.146797 9.353141 12.314255

H 10.062782 7.738030 11.594088

H 8.791734 8.265997 12.703194

C 8.975442 11.969438 8.186692

H 8.359504 12.833773 8.472006

H 8.924884 11.891268 7.095962

H 10.009529 12.187644 8.469785

108

**1∙2PF_6_ (PBE, GP)**

C -1.295200 5.320491 -1.912547

C -2.627399 5.107894 -2.122687

N -2.948357 3.947105 -1.438173

C -1.866802 3.458937 -0.775544

N -0.833212 4.286914 -1.095231

C -4.806432 -1.240864 -0.633305

C -3.778332 -2.081234 -0.313179

C -4.224365 3.239557 -1.530756

N -4.352309 0.043034 -0.386665

C -3.091862 0.025149 0.112611

C -0.072654 4.191769 2.953874

C -5.215100 1.229803 -0.443420

N -2.724167 -1.289312 0.145992

C -1.525537 -1.860772 0.709848

C -2.732954 2.363608 1.782540

C -4.159660 3.289269 3.266823

C -3.058218 2.874194 3.960635

N -4.463177 2.471250 -0.315558

C -4.816293 3.295230 0.839539

Fe -2.042902 1.688485 0.076091

N -3.931306 2.965778 1.944272

C -1.663274 -2.633729 1.891539

N -2.188745 2.305743 3.032367

C -0.493607 -3.176040 2.451945

C 0.777637 -2.980645 1.881266

C 0.855420 -2.259041 0.679791

C -0.281674 -1.713858 0.053978

C -3.001942 -2.913820 2.524207

C 2.018489 -3.522886 2.545078

C -0.142041 -1.108730 -1.318007

C 2.498985 4.792574 0.515921

C 1.175128 5.063762 0.115591

C 0.549070 4.114478 -0.723955

C 1.263573 3.029654 -1.293182

C 2.572744 2.800802 -0.839334

C 3.197101 3.648554 0.094407

C 0.502452 6.341176 0.543175

C 0.703451 2.234053 -2.443417

C 4.577225 3.337481 0.617043

C 1.505382 2.207868 3.235109

C 0.204588 2.728207 3.138560

C -0.870171 1.823733 3.308531

C -0.690812 0.502588 3.774529

C 0.631847 0.036299 3.865167

C 1.734982 0.853755 3.547310

C 3.133668 0.290424 3.544625

C -1.863566 -0.308526 4.248319

F -2.155333 1.236227 -1.737466

N -0.692237 1.340289 0.639586

H -0.642791 6.129458 -2.234249

H -3.375315 5.716548 -2.626809

H -5.824648 -1.448483 -0.957766

H -3.701047 -3.166081 -0.350165

H -4.172292 2.534059 -2.377330

H -5.007211 4.001260 -1.716088

H -0.558881 4.605834 3.857455

H 0.858726 4.749359 2.786872

H -0.774297 4.404216 2.130999

H -5.919575 1.140292 0.402385

H -5.779413 1.188789 -1.393595

H -5.077192 3.771591 3.593371

H -2.818997 2.911409 5.021726

H -5.847130 3.103937 1.176624

H -4.690160 4.359921 0.588971

H -0.585013 -3.769619 3.371661

H 1.829786 -2.133314 0.188546

H -3.507189 -3.760413 2.021699

H -2.881767 -3.203531 3.580636

H -3.698297 -2.062772 2.494311

H 1.804432 -4.436269 3.126032

H 2.804198 -3.758464 1.806799

H 2.444004 -2.779646 3.246678

H 0.798125 -0.537835 -1.393183

H -0.094220 -1.916425 -2.073722

H -0.970756 -0.440924 -1.595527

H 2.993379 5.506402 1.188652

H 3.129564 1.948997 -1.252186

H 1.006289 6.774054 1.422715

H 0.557332 7.098840 -0.261198

H -0.564632 6.223438 0.777032

H -0.301994 1.824263 -2.255435

H 0.616136 2.885780 -3.334334

H 1.378045 1.404079 -2.708127

H 5.230861 2.932028 -0.175144

H 5.063931 4.230808 1.043435

H 4.526292 2.572686 1.416456

H 2.355643 2.881145 3.066860

H 0.801009 -0.996171 4.195837

H 3.889305 1.069600 3.744567

H 3.255365 -0.509280 4.295857

H 3.366696 -0.150379 2.555647

H -2.206776 0.068543 5.231145

H -2.741421 -0.236742 3.586926

H -1.585371 -1.364033 4.379450

P -5.991300 -0.029501 3.071993

F -7.471506 -0.193317 3.693476

F -6.477880 -0.712637 1.644033

F -5.529450 -1.491898 3.629608

F -5.436584 0.706682 4.415519

F -6.405495 1.460484 2.435828

F -4.463175 0.157271 2.388857

P -3.431681 6.729621 1.138906

F -4.305444 8.086108 1.190126

F -4.365427 6.158944 -0.135593

F -4.467103 5.977786 2.177122

F -2.481598 7.198134 2.370099

F -2.398146 7.383151 0.045516

F -2.553522 5.294941 1.055888

108

**1∙2PF_6_ (PBE, SMD=MeCN)**

C -1.292729 5.326533 -1.959960

C -2.634474 5.133672 -2.144563

N -2.964981 3.986579 -1.440824

C -1.878226 3.480848 -0.801189

N -0.835086 4.292176 -1.139465

C -4.791364 -1.242227 -0.679902

C -3.758920 -2.078683 -0.355091

C -4.242717 3.276999 -1.548800

N -4.353894 0.043163 -0.409005

C -3.093950 0.031636 0.088561

C -0.074245 4.166858 2.878013

C -5.210774 1.232459 -0.531129

N -2.715162 -1.281776 0.118433

C -1.512079 -1.841314 0.686143

C -2.729489 2.345059 1.763945

C -4.178184 3.221743 3.259872

C -3.067821 2.821640 3.950959

N -4.459816 2.470537 -0.349429

C -4.829171 3.257354 0.823591

Fe -2.047637 1.700819 0.043622

N -3.942229 2.920025 1.930976

C -1.642824 -2.600560 1.876414

N -2.186343 2.280637 3.016261

C -0.468354 -3.112029 2.458581

C 0.803841 -2.900875 1.894804

C 0.878711 -2.198250 0.678663

C -0.263817 -1.684183 0.035727

C -2.980979 -2.889732 2.504054

C 2.050366 -3.422231 2.562708

C -0.124660 -1.093210 -1.341420

C 2.521875 4.732674 0.446383

C 1.201372 5.026195 0.050474

C 0.551098 4.095339 -0.791400

C 1.248063 3.006973 -1.378182

C 2.557694 2.753055 -0.933478

C 3.199889 3.580448 0.008991

C 0.553697 6.314462 0.481050

C 0.674485 2.233047 -2.534697

C 4.572910 3.238951 0.526896

C 1.512776 2.215307 3.285595

C 0.208663 2.715853 3.135882

C -0.865037 1.809795 3.310713

C -0.681197 0.507640 3.826608

C 0.644449 0.056211 3.967341

C 1.750096 0.874490 3.653804

C 3.154398 0.333700 3.720562

C -1.849317 -0.306735 4.309320

F -2.162304 1.251695 -1.768785

N -0.687668 1.357860 0.591661

H -0.621267 6.102601 -2.326953

H -3.376041 5.710077 -2.697706

H -5.791762 -1.448740 -1.060360

H -3.670209 -3.163656 -0.406632

H -4.196598 2.600861 -2.416675

H -5.034738 4.031933 -1.706013

H -0.667074 4.599719 3.706339

H 0.859745 4.739302 2.788200

H -0.679252 4.321177 1.971438

H -5.982297 1.142108 0.250396

H -5.695590 1.196444 -1.521018

H -5.105987 3.682211 3.592854

H -2.821248 2.862427 5.011786

H -5.860672 3.051702 1.149291

H -4.715248 4.329078 0.600918

H -0.554394 -3.681157 3.393768

H 1.855780 -2.056302 0.196636

H -3.478844 -3.735147 1.991553

H -2.859295 -3.180843 3.560493

H -3.673455 -2.036792 2.461805

H 1.825622 -3.873412 3.543869

H 2.545276 -4.190823 1.939541

H 2.787928 -2.613508 2.716408

H 0.820977 -0.532987 -1.430434

H -0.088026 -1.909266 -2.089479

H -0.953462 -0.426978 -1.621314

H 3.028056 5.428876 1.128446

H 3.098465 1.896682 -1.358339

H 1.060589 6.729139 1.368211

H 0.634832 7.070055 -0.323971

H -0.516757 6.209051 0.703433

H -0.346228 1.860712 -2.353523

H 0.618664 2.890615 -3.424421

H 1.321852 1.379299 -2.792107

H 5.224098 2.846555 -0.274457

H 5.065516 4.114196 0.983756

H 4.509746 2.450963 1.302892

H 2.361428 2.893399 3.125161

H 0.817244 -0.960986 4.342302

H 3.882310 1.121697 3.981023

H 3.242004 -0.485213 4.455201

H 3.454687 -0.076024 2.736268

H -2.172384 0.066290 5.301313

H -2.732380 -0.234102 3.655945

H -1.569804 -1.363737 4.431294

P -6.097559 -0.091941 3.091346

F -7.589079 -0.266769 3.728621

F -6.638180 -0.644845 1.634686

F -5.709128 -1.623732 3.528877

F -5.525960 0.480441 4.513490

F -6.474421 1.447214 2.617827

F -4.581920 0.091143 2.432045

P -3.414456 6.773295 1.367400

F -4.250596 8.159085 1.573480

F -4.365382 6.377160 0.069414

F -4.465945 5.979439 2.354478

F -2.455747 7.119038 2.647625

F -2.370075 7.535912 0.357303

F -2.575761 5.355840 1.150690

94

**2 (PBE)**

C -2.049852 1.391337 4.313783

C -2.707489 0.204556 4.489847

N -2.295530 -0.622172 3.451684

C -1.402675 0.020366 2.638262

N -1.253228 1.261094 3.171559

C -2.950549 -3.986163 -0.908289

C -2.774564 -3.222452 -2.031269

C -2.870448 -1.924147 3.114452

N -2.270693 -3.334944 0.118500

C -1.672973 -2.205984 -0.344464

C 2.356436 1.229272 1.018756

C -2.275687 -3.764870 1.527126

N -1.997954 -2.121573 -1.665128

C -1.743256 -1.001054 -2.542273

C 0.570170 -1.778429 1.489311

C 1.817143 -3.105259 2.856306

C 2.665913 -2.501560 1.971354

N -1.834377 -2.696669 2.421170

C -0.678824 -3.005609 3.255134

Fe -1.034659 -0.820052 0.868118

N 0.543156 -2.653908 2.533694

C -0.572091 -0.988859 -3.336786

N 1.889195 -1.703494 1.128109

C -0.355573 0.121608 -4.202127

C -1.277161 1.171787 -4.325516

C -2.456795 1.085279 -3.554092

C -2.733027 0.000235 -2.673136

C 0.424470 -2.095120 -3.326231

C -1.057023 2.314138 -5.276295

C -4.078586 -0.083249 -2.018187

C 1.444283 3.835815 3.096296

C 0.694553 2.702830 3.496748

C -0.418721 2.341978 2.706408

C -0.793636 3.076121 1.550367

C 0.017285 4.185538 1.187501

C 1.120490 4.598840 1.949442

C 1.094884 1.938354 4.729397

C -2.018123 2.776966 0.736343

C 1.918784 5.819919 1.590564

C 3.369636 0.817478 -1.242802

C 2.718820 0.298851 -0.093339

C 2.466641 -1.093203 -0.045775

C 2.906932 -1.964105 -1.073426

C 3.580699 -1.389892 -2.179941

C 3.819715 -0.000002 -2.290580

C 4.571025 0.559334 -3.465222

C 2.707958 -3.454232 -1.009542

F -2.825092 -0.412753 0.750103

N -0.276981 0.118810 -0.025269

H -2.075262 2.314638 4.894034

H -3.420424 -0.109730 5.254558

H -3.489871 -4.924295 -0.763329

H -3.131433 -3.362694 -3.052834

H -3.718968 -1.779798 2.424551

H -3.225524 -2.405286 4.047317

H 3.213569 1.878355 1.276575

H 1.541225 1.911527 0.706570

H 2.030348 0.706187 1.929489

H -1.602291 -4.633225 1.635849

H -3.299850 -4.105714 1.768150

H 2.011905 -3.805856 3.670537

H 3.748476 -2.573215 1.858269

H -0.624720 -4.075087 3.534264

H -0.732389 -2.416879 4.187609

H 0.560098 0.134464 -4.807627

H -3.217361 1.871406 -3.652338

H 0.086876 -2.991328 -2.784450

H 0.703432 -2.383452 -4.355856

H 1.365902 -1.739953 -2.850071

H -0.000385 2.406217 -5.573985

H -1.647554 2.159623 -6.199584

H -1.388767 3.273602 -4.844306

H -4.507875 0.921358 -1.870892

H -4.782711 -0.627379 -2.680292

H -4.051295 -0.599188 -1.048022

H 2.308006 4.129972 3.707376

H -0.262949 4.760037 0.294961

H 2.174961 2.042702 4.923088

H 0.578717 2.327869 5.628347

H 0.853782 0.863935 4.658129

H -2.568187 1.880180 1.055799

H -2.710281 3.640470 0.772865

H -1.748878 2.649936 -0.330389

H 1.715695 6.160015 0.562717

H 1.663535 6.654466 2.271429

H 3.003252 5.645577 1.699924

H 3.557738 1.898013 -1.287646

H 3.944867 -2.058916 -2.971191

H 5.657213 0.385799 -3.339911

H 4.281246 0.066256 -4.409008

H 4.423029 1.645782 -3.571903

H 3.505101 -3.938536 -0.413584

H 1.744898 -3.729344 -0.545956

H 2.754875 -3.904683 -2.014069

67

**2 (PBE, trunc)**

Fe 0.000000 0.000000 0.000000

F 0.000000 1.728700 -0.618580

N 0.029165 -0.495258 -2.519219

N 2.243522 0.236334 -1.800534

N 2.956412 0.692986 0.204621

N -0.182144 -2.585572 -1.362910

N 0.197721 -2.932098 0.744096

N -2.099556 0.379725 -1.953548

N -3.093510 0.381061 -0.027217

N 0.000000 0.000000 1.501349

C 1.392560 -0.178998 -2.932703

H 1.862099 -1.059053 -3.408506

H 1.402113 0.640049 -3.674680

C 1.890965 0.189720 -0.488341

C 3.517360 0.774666 -1.950211

H 4.001979 0.906302 -2.920377

C 3.964019 1.062144 -0.688989

H 4.907924 1.494979 -0.351000

C -0.393745 -1.887778 -2.635146

H -1.467020 -1.928267 -2.893283

H 0.158860 -2.433700 -3.423469

C 0.027056 -1.942167 -0.183347

C -0.183858 -3.966321 -1.189747

H -0.349802 -4.671160 -2.007606

C 0.053595 -4.183695 0.140356

H 0.151843 -5.113527 0.704305

C -1.004664 0.467253 -2.916197

H -0.573776 1.481808 -2.877042

H -1.409685 0.277532 -3.930125

C -1.887877 0.173576 -0.619453

C -3.425482 0.712635 -2.204789

H -3.809207 0.930820 -3.204065

C -4.053309 0.712337 -0.989612

H -5.089843 0.925471 -0.720595

C 3.111430 0.761266 1.640258

C 2.403524 1.731969 2.394196

C 2.573863 1.732487 3.807308

H 2.019533 2.480661 4.389213

C 3.460367 0.870421 4.469362

C 4.202932 -0.022305 3.668813

H 4.960203 -0.662150 4.140929

C 4.071950 -0.079663 2.250916

C 0.716298 -2.761791 2.079890

C 2.088302 -3.017602 2.278747

C 2.593527 -2.880575 3.596003

H 3.652264 -3.109255 3.773234

C 1.768953 -2.537825 4.696532

C 0.403052 -2.329814 4.449101

H -0.265438 -2.096448 5.287542

C -0.159608 -2.470994 3.159636

C -3.417061 0.370937 1.378733

C -4.199002 -0.715146 1.881592

C -4.631407 -0.649444 3.219595

H -5.249832 -1.466228 3.613913

C -4.320333 0.443438 4.046963

C -3.538989 1.497133 3.496658

H -3.316045 2.370202 4.124282

C -3.090531 1.500178 2.157588

H 1.738966 2.450879 1.901677

H 3.598072 0.912826 5.554972

H 4.681176 -0.772212 1.658942

H -1.236603 -2.360193 2.993518

H 2.737854 -3.316709 1.448734

H 2.180071 -2.467880 5.709397

H -4.468104 -1.558506 1.235012

H -4.680105 0.495383 5.080408

H -2.540989 2.347938 1.734178

94

**2 (PBE, CPCM=MeCN)**

C -2.101806 1.236763 4.615631

C -2.688391 0.010155 4.777814

N -2.370837 -0.722913 3.644638

C -1.601988 0.006986 2.790482

N -1.436000 1.214618 3.387763

C -2.783843 -4.040729 -0.888204

C -2.518544 -3.322168 -2.023230

C -2.871179 -2.052759 3.306489

N -2.252322 -3.317615 0.171083

C -1.671995 -2.176594 -0.271984

C 2.056743 1.610588 0.812194

C -2.329429 -3.748049 1.582109

N -1.827200 -2.175027 -1.630110

C -1.261498 -1.213868 -2.539591

C 0.424823 -1.539211 1.501917

C 1.857960 -2.773560 2.743248

C 2.599308 -2.067501 1.834658

N -1.848533 -2.714538 2.491067

C -0.618800 -2.999485 3.226340

Fe -1.262475 -0.724292 0.974254

N 0.531827 -2.442541 2.506374

C -0.230706 -1.632380 -3.412188

N 1.703507 -1.325005 1.062269

C 0.386189 -0.645734 -4.239246

C -0.023204 0.704293 -4.243994

C -1.107379 1.048313 -3.429101

C -1.773782 0.104658 -2.594667

C 0.141516 -3.071588 -3.601013

C 0.646903 1.733403 -5.112007

C -3.043541 0.527193 -1.928598

C 1.237789 3.785074 3.080214

C 0.540676 2.642392 3.524184

C -0.685059 2.335735 2.891169

C -1.221878 3.121294 1.847592

C -0.456406 4.231258 1.416246

C 0.765170 4.583126 2.020773

C 1.103598 1.767921 4.612288

C -2.580591 2.863978 1.256512

C 1.571269 5.750571 1.514812

C 2.808407 1.169512 -1.542991

C 2.309292 0.667557 -0.311151

C 2.096803 -0.730309 -0.190610

C 2.391265 -1.617390 -1.247489

C 2.878460 -1.060063 -2.458632

C 3.125130 0.330945 -2.613193

C 3.781820 0.844197 -3.862800

C 2.278796 -3.104323 -1.085441

F -3.095923 -0.546401 0.913571

N -0.639237 0.352338 0.134352

H -2.085380 2.118628 5.256825

H -3.289158 -0.401542 5.589901

H -3.294662 -4.994009 -0.744969

H -2.755554 -3.522102 -3.068326

H -3.791150 -1.957666 2.708771

H -3.090021 -2.593388 4.246553

H 2.875563 2.346892 0.897732

H 1.139546 2.200592 0.610823

H 1.922559 1.097793 1.775226

H -1.722399 -4.662710 1.687483

H -3.380791 -4.005696 1.794675

H 2.152073 -3.476534 3.523915

H 3.674069 -2.035461 1.652208

H -0.448333 -4.083464 3.349010

H -0.667775 -2.539569 4.227800

H 1.171665 -0.973716 -4.931694

H -1.496196 2.074675 -3.449001

H 0.060331 -3.664195 -2.676585

H -0.550716 -3.529564 -4.335496

H 1.157395 -3.169285 -4.014444

H 1.278014 1.266323 -5.884831

H -0.100625 2.379141 -5.604314

H 1.290360 2.392031 -4.499536

H -2.957773 1.559663 -1.547403

H -3.846643 0.544083 -2.693528

H -3.363997 -0.132182 -1.110336

H 2.192293 4.036066 3.560699

H -0.847814 4.844967 0.594414

H 2.157188 2.022315 4.808451

H 0.545493 1.886886 5.559779

H 1.044174 0.698563 4.337749

H -2.837012 1.793741 1.191390

H -3.357427 3.341304 1.885791

H -2.655874 3.306091 0.249578

H 0.930073 6.507975 1.033501

H 2.141446 6.230002 2.328493

H 2.303843 5.408138 0.758364

H 2.981775 2.249772 -1.628343

H 3.135060 -1.743531 -3.278045

H 4.863677 0.611537 -3.831898

H 3.376489 0.355408 -4.764938

H 3.674776 1.936003 -3.962399

H 3.052333 -3.477463 -0.387291

H 1.302712 -3.399932 -0.659237

H 2.426079 -3.620823 -2.044514

94

**2 (TPSSh)**

C -2.064479 1.410622 4.274154

C -2.732474 0.237425 4.440980

N -2.319166 -0.592046 3.407593

C -1.415537 0.040930 2.609177

N -1.259078 1.271843 3.142630

C -2.906767 -4.016972 -0.899119

C -2.745425 -3.265623 -2.022537

C -2.887862 -1.897650 3.072215

N -2.233176 -3.349487 0.120205

C -1.653446 -2.223746 -0.353588

C 2.334270 1.207745 1.016727

C -2.235784 -3.762358 1.533544

N -1.982018 -2.154983 -1.666345

C -1.747629 -1.022421 -2.531746

C 0.575574 -1.766518 1.485818

C 1.806512 -3.077575 2.867947

C 2.655369 -2.504168 1.974749

N -1.829368 -2.663846 2.406982

C -0.687071 -2.943063 3.268502

Fe -1.034279 -0.802876 0.838427

N 0.538676 -2.617125 2.541520

C -0.578549 -0.983363 -3.318553

N 1.885998 -1.713874 1.122527

C -0.362720 0.149151 -4.148875

C -1.284178 1.194958 -4.240798

C -2.461947 1.082294 -3.479910

C -2.734502 -0.028008 -2.632201

C 0.421920 -2.085179 -3.330478

C -1.064176 2.368636 -5.155991

C -4.080515 -0.119207 -1.977619

C 1.464112 3.804342 3.069605

C 0.699139 2.688324 3.468254

C -0.410321 2.341696 2.677590

C -0.769180 3.068124 1.522030

C 0.053685 4.162137 1.158709

C 1.155085 4.560951 1.921963

C 1.079527 1.918636 4.705374

C -1.993515 2.774928 0.702449

C 1.970490 5.772202 1.558518

C 3.330383 0.807468 -1.252087

C 2.696721 0.283027 -0.100993

C 2.463517 -1.104863 -0.051867

C 2.902831 -1.969312 -1.074079

C 3.562206 -1.392390 -2.182351

C 3.779920 -0.004886 -2.296291

C 4.514376 0.563617 -3.480011

C 2.712630 -3.461460 -1.007671

F -2.800104 -0.413340 0.695043

N -0.279433 0.125336 -0.038430

H -2.084092 2.328065 4.847664

H -3.449986 -0.070664 5.191363

H -3.428786 -4.952948 -0.742740

H -3.101570 -3.413707 -3.033882

H -3.715200 -1.764413 2.369801

H -3.247535 -2.368693 3.996560

H 3.201969 1.816139 1.302186

H 1.552464 1.910988 0.696403

H 1.976837 0.676929 1.901078

H -1.538493 -4.596631 1.657480

H -3.244354 -4.119391 1.770544

H 1.990570 -3.760150 3.688327

H 3.728106 -2.587258 1.858771

H -0.636097 -3.993564 3.578959

H -0.750828 -2.319787 4.165690

H 0.547408 0.181332 -4.745974

H -3.219747 1.861621 -3.555245

H 0.080136 -2.984441 -2.813835

H 0.699639 -2.345179 -4.358937

H 1.349900 -1.737119 -2.841656

H -0.013687 2.462809 -5.444643

H -1.652984 2.243025 -6.074419

H -1.387751 3.304648 -4.688641

H -4.361660 0.844305 -1.538935

H -4.836918 -0.350539 -2.741979

H -4.124371 -0.879570 -1.198655

H 2.322140 4.087398 3.678070

H -0.212211 4.731687 0.269259

H 2.150040 2.018340 4.907024

H 0.552128 2.305288 5.588325

H 0.837102 0.853712 4.618013

H -2.538921 1.887748 1.029254

H -2.675467 3.635411 0.739615

H -1.717832 2.639316 -0.352601

H 1.795030 6.082062 0.524571

H 1.698952 6.614039 2.209438

H 3.041609 5.589855 1.697244

H 3.502357 1.881773 -1.299075

H 3.924411 -2.053712 -2.968817

H 5.597125 0.438194 -3.344131

H 4.244245 0.045360 -4.406166

H 4.317698 1.632588 -3.601607

H 3.509997 -3.933234 -0.417738

H 1.756884 -3.731471 -0.544846

H 2.755895 -3.903632 -2.007000

67

**2 (TPSSh, trunc)**

C -2.063640 1.410527 4.273039

C -2.731828 0.237466 4.440051

N -2.318941 -0.592105 3.406576

C -1.415385 0.040678 2.607925

N -1.258548 1.271569 3.141318

C -2.908294 -4.016987 -0.899932

C -2.747082 -3.265689 -2.023402

C -2.887981 -1.897601 3.071361

N -2.234317 -3.349619 0.119214

C -1.654481 -2.224001 -0.354743

C -2.236656 -3.762465 1.532560

N -1.983365 -2.155195 -1.667419

C -1.748965 -1.022695 -2.532898

C 0.575087 -1.767186 1.484102

C 1.806107 -3.078465 2.865948

C 2.654857 -2.505242 1.972529

N -1.829805 -2.664019 2.405879

C -0.687349 -2.943449 3.267120

Fe -1.034735 -0.803234 0.837094

N 0.538282 -2.617768 2.539828

C -0.580073 -0.983883 -3.319996

N 1.885431 -1.714809 1.120485

C -0.364225 0.148574 -4.150391

C -1.285497 1.194563 -4.242103

C -2.463100 1.082146 -3.480920

C -2.735665 -0.028087 -2.633124

C 1.465128 3.803524 3.067573

C 0.700032 2.687665 3.466431

C -0.409694 2.341245 2.676049

C -0.768695 3.067724 1.520565

C 0.054298 4.161567 1.157021

C 1.155967 4.560175 1.919995

C 3.329729 0.806205 -1.254531

C 2.696248 0.281909 -0.103271

C 2.462780 -1.105933 -0.054063

C 2.901668 -1.970487 -1.076370

C 3.560883 -1.393715 -2.184815

C 3.778845 -0.006257 -2.298833

F -2.800518 -0.413349 0.694142

N -0.279922 0.124812 -0.039966

H -2.081852 2.326855 4.849674

H -3.447796 -0.069156 5.192965

H -3.427695 -4.955353 -0.746579

H -3.100803 -3.417737 -3.035618

H -3.715649 -1.762376 2.369582

H -3.247723 -2.368246 3.995794

H -1.539417 -4.597000 1.654733

H -3.245234 -4.119457 1.769414

H 1.992256 -3.760644 3.686558

H 3.727302 -2.593855 1.851929

H -0.636285 -3.993783 3.577977

H -0.749798 -2.320785 4.164894

H 0.541094 0.174872 -4.754742

H -3.221915 1.859434 -3.558638

H 2.314225 4.095709 3.683740

H -0.216293 4.735746 0.272325

H 3.510371 1.879052 -1.297188

H 3.926285 -2.055279 -2.968773

H 1.750753 5.426319 1.644195

H -1.657188 2.808719 0.950690

H 0.948349 2.128370 4.364962

H 2.402564 0.927309 0.719950

H 4.299421 0.410614 -3.156480

H 2.752694 -3.045157 -1.007484

H -3.650975 -0.082301 -2.050174

H 0.151078 -1.786008 -3.269113

H -1.115750 2.048274 -4.891842

94

**2 (TPSSh, CPCM=MeCN)**

C -2.173332 1.410216 4.219385

C -2.818256 0.223570 4.391484

N -2.401814 -0.598808 3.357589

C -1.513846 0.046688 2.559480

N -1.375006 1.281893 3.082179

C -2.748573 -4.109683 -0.909078

C -2.583420 -3.369797 -2.041343

C -2.917381 -1.934427 3.045305

N -2.141528 -3.403333 0.122843

C -1.594865 -2.267097 -0.347414

C 2.266404 1.297389 0.864094

C -2.170361 -3.793787 1.546502

N -1.878158 -2.225656 -1.673474

C -1.627001 -1.062610 -2.481148

C 0.556111 -1.686933 1.517235

C 1.811333 -2.909588 2.946553

C 2.654652 -2.344029 2.043485

N -1.821135 -2.663752 2.404262

C -0.689086 -2.882990 3.296811

Fe -1.071049 -0.790605 0.814323

N 0.536088 -2.499678 2.595217

C -0.421914 -0.974006 -3.210722

N 1.868244 -1.607508 1.159354

C -0.095298 0.263265 -3.835685

C -0.947843 1.363973 -3.776911

C -2.193794 1.185329 -3.149059

C -2.572216 -0.031323 -2.512729

C 0.505391 -2.116239 -3.352807

C -0.563168 2.695956 -4.361979

C -3.968868 -0.164056 -1.985841

C 1.391448 3.742226 2.889534

C 0.611736 2.665901 3.329991

C -0.539974 2.344532 2.585956

C -0.923108 3.054794 1.434085

C -0.088627 4.112968 1.024376

C 1.061016 4.474701 1.736275

C 1.012809 1.861100 4.540190

C -2.169257 2.743037 0.648276

C 1.950773 5.594192 1.257844

C 3.117509 0.790988 -1.445271

C 2.581168 0.318779 -0.215614

C 2.406697 -1.066458 -0.057544

C 2.837455 -1.981779 -1.034622

C 3.428088 -1.458639 -2.213912

C 3.550572 -0.074011 -2.446950

C 4.121783 0.433329 -3.743294

C 2.683600 -3.466645 -0.868984

F -2.847733 -0.463733 0.622671

N -0.333513 0.157369 -0.056344

H -2.195875 2.327841 4.791681

H -3.518605 -0.107698 5.146941

H -3.235602 -5.062193 -0.745181

H -2.895620 -3.542726 -3.062627

H -3.745673 -1.851444 2.337959

H -3.255911 -2.399956 3.978027

H 3.142703 1.929226 1.058448

H 1.462229 1.974396 0.544431

H 1.967739 0.808952 1.792255

H -1.449273 -4.601072 1.697187

H -3.175244 -4.168808 1.761414

H 1.994566 -3.560615 3.791273

H 3.728955 -2.402374 1.930352

H -0.593987 -3.929866 3.600823

H -0.797589 -2.262695 4.190438

H 0.851765 0.334129 -4.367228

H -2.910524 2.005447 -3.142001

H 0.104791 -3.061208 -2.982000

H 0.844626 -2.218826 -4.389815

H 1.425645 -1.882994 -2.775074

H 0.287434 2.597004 -5.042654

H -1.404012 3.146578 -4.900317

H -0.277873 3.385834 -3.556343

H -4.240781 0.718345 -1.397814

H -4.656975 -0.212706 -2.842040

H -4.105748 -1.054322 -1.372703

H 2.288312 4.001361 3.450880

H -0.363908 4.673505 0.131561

H 2.035606 2.108852 4.837956

H 0.353273 2.062456 5.393934

H 0.959255 0.785224 4.331869

H -2.842785 2.066942 1.179344

H -2.706050 3.668857 0.413133

H -1.909489 2.268348 -0.308551

H 1.413615 6.266526 0.581166

H 2.341261 6.176490 2.099831

H 2.812443 5.185249 0.712315

H 3.217978 1.866865 -1.579047

H 3.786642 -2.162738 -2.963485

H 5.078022 -0.054834 -3.964317

H 3.437517 0.205007 -4.570857

H 4.274246 1.515833 -3.710079

H 3.467627 -3.865857 -0.211617

H 1.715428 -3.718732 -0.421690

H 2.771487 -3.968051 -1.836541

94

**2 (PBE0)**

C -2.064495 1.414035 4.229431

C -2.726869 0.242258 4.398432

N -2.309296 -0.584963 3.375057

C -1.411431 0.047711 2.583050

N -1.259617 1.273705 3.108362

C -2.839058 -4.024355 -0.898092

C -2.677491 -3.281669 -2.022203

C -2.870979 -1.886422 3.054073

N -2.189737 -3.343691 0.118195

C -1.624813 -2.220439 -0.355612

C 2.381884 1.206918 1.017476

C -2.228729 -3.742812 1.527428

N -1.937251 -2.164357 -1.666128

C -1.734299 -1.030174 -2.521073

C 0.574053 -1.754368 1.489370

C 1.791239 -3.047577 2.876704

C 2.641941 -2.476651 1.989093

N -1.827301 -2.666422 2.407674

C -0.688379 -2.936820 3.254619

Fe -1.020717 -0.793426 0.823561

N 0.531208 -2.595905 2.541298

C -0.576917 -0.958012 -3.318870

N 1.878587 -1.697996 1.134340

C -0.387707 0.187395 -4.136271

C -1.322252 1.215511 -4.207560

C -2.486059 1.070403 -3.438955

C -2.733267 -0.055283 -2.604180

C 0.438979 -2.029511 -3.354128

C -1.133412 2.398931 -5.102771

C -4.062985 -0.156725 -1.939382

C 1.461863 3.782995 3.049876

C 0.696448 2.674684 3.439811

C -0.413083 2.337161 2.649398

C -0.768693 3.068443 1.502985

C 0.054086 4.155424 1.148593

C 1.155152 4.543738 1.912085

C 1.068755 1.900537 4.668720

C -1.989112 2.785240 0.683665

C 1.964464 5.752663 1.558552

C 3.359346 0.779425 -1.241844

C 2.722385 0.273699 -0.090156

C 2.456688 -1.104024 -0.037290

C 2.863444 -1.975461 -1.060725

C 3.525819 -1.417859 -2.171333

C 3.780179 -0.040791 -2.285214

C 4.522002 0.506155 -3.464122

C 2.636350 -3.454363 -0.990783

F -2.774848 -0.404895 0.659416

N -0.255041 0.111418 -0.031010

H -2.091864 2.334044 4.801352

H -3.446688 -0.062718 5.149913

H -3.354468 -4.965911 -0.742847

H -3.027118 -3.443388 -3.035440

H -3.702055 -1.760115 2.352225

H -3.242101 -2.342734 3.983472

H 3.252396 1.821305 1.280698

H 1.595315 1.909419 0.705320

H 2.040080 0.693507 1.918494

H -1.558505 -4.598466 1.667220

H -3.249625 -4.084713 1.741249

H 1.975855 -3.726456 3.701968

H 3.717361 -2.558259 1.882978

H -0.624900 -3.989671 3.560611

H -0.755231 -2.329767 4.164448

H 0.517803 0.241679 -4.740482

H -3.259063 1.838033 -3.495538

H 0.123772 -2.951985 -2.861445

H 0.736851 -2.254083 -4.385893

H 1.360665 -1.671048 -2.851983

H -0.087535 2.523812 -5.397073

H -1.722121 2.274262 -6.021766

H -1.475724 3.323252 -4.625282

H -4.270151 0.748763 -1.356517

H -4.847764 -0.219914 -2.707132

H -4.152883 -1.014296 -1.273528

H 2.320683 4.062286 3.660551

H -0.210777 4.736094 0.264818

H 2.133099 2.017879 4.893485

H 0.521338 2.262591 5.549996

H 0.853259 0.830368 4.569780

H -2.567850 1.926792 1.030657

H -2.647060 3.664435 0.681632

H -1.711900 2.602820 -0.364694

H 1.838397 6.036477 0.509579

H 1.646644 6.608367 2.169682

H 3.030789 5.598812 1.755217

H 3.559435 1.849763 -1.290131

H 3.870582 -2.088731 -2.959011

H 5.604131 0.387886 -3.315152

H 4.268407 -0.026114 -4.386988

H 4.328083 1.573158 -3.608328

H 3.411236 -3.946136 -0.386913

H 1.668063 -3.703329 -0.541325

H 2.684499 -3.906867 -1.985628

67

**2 (PBE0, trunc)**

C -2.063985 1.414256 4.228110

C -2.726460 0.242569 4.397343

N -2.309165 -0.584796 3.373971

C -1.411373 0.047701 2.581741

N -1.259326 1.273728 3.106908

C -2.840089 -4.024536 -0.898754

C -2.678642 -3.281976 -2.022965

C -2.871052 -1.886223 3.053213

N -2.190507 -3.343848 0.117350

C -1.625543 -2.220705 -0.356666

C -2.229288 -3.742830 1.526628

N -1.938213 -2.164713 -1.667131

C -1.735289 -1.030634 -2.522221

C 0.573710 -1.754704 1.487872

C 1.791003 -3.047918 2.875108

C 2.641608 -2.477172 1.987288

N -1.827579 -2.666402 2.406699

C -0.688534 -2.936847 3.253463

Fe -1.021073 -0.793647 0.822261

N 0.530962 -2.596136 2.539888

C -0.578043 -0.958678 -3.320235

N 1.878186 -1.698512 1.132600

C -0.388854 0.186630 -4.137780

C -1.323296 1.214844 -4.208997

C -2.486980 1.069940 -3.440167

C -2.734162 -0.055639 -2.605240

C 1.462424 3.782708 3.047689

C 0.696956 2.674520 3.437868

C -0.412756 2.337046 2.647689

C -0.768493 3.068258 1.501271

C 0.054344 4.155113 1.146626

C 1.155592 4.543377 1.909881

C 3.358791 0.778516 -1.244089

C 2.721983 0.272971 -0.092238

C 2.456141 -1.104717 -0.039192

C 2.862613 -1.976297 -1.062618

C 3.524849 -1.418875 -2.173399

C 3.779343 -0.041847 -2.287458

F -2.775190 -0.404935 0.658397

N -0.255451 0.111030 -0.032535

H -2.089717 2.332317 4.804333

H -3.445613 -0.060515 5.150633

H -3.353865 -4.967448 -0.745328

H -3.026249 -3.447151 -3.036619

H -3.702580 -1.758478 2.352095

H -3.241937 -2.341901 3.982992

H -1.559146 -4.598674 1.665708

H -3.250150 -4.084732 1.740479

H 1.976983 -3.729127 3.698389

H 3.716284 -2.566513 1.875138

H -0.625091 -3.989806 3.559120

H -0.753878 -2.331217 4.164380

H 0.516311 0.240079 -4.742300

H -3.256237 1.841261 -3.490953

H 2.313637 4.070069 3.664992

H -0.212366 4.738763 0.265714

H 3.564683 1.847531 -1.290548

H 3.868512 -2.090013 -2.960811

H -3.647471 -0.134529 -2.019545

H 0.157923 -1.760225 -3.304204

H -1.173019 2.082234 -4.847208

H 2.449706 0.926851 0.733625

H 2.692492 -3.048584 -0.988985

H 4.306019 0.361737 -3.149053

H -1.658753 2.813228 0.930014

H 1.752802 5.410522 1.638129

H 0.940165 2.109031 4.335401

94

**2 (PBE0, CPCM=MeCN)**

C -2.181987 1.420184 4.150891

C -2.835751 0.240270 4.303370

N -2.402127 -0.575755 3.281763

C -1.498172 0.065441 2.510983

N -1.363181 1.290786 3.038665

C -2.667989 -4.102532 -0.904146

C -2.512848 -3.372147 -2.038006

C -2.900700 -1.909482 2.978732

N -2.065354 -3.388472 0.114874

C -1.529469 -2.258521 -0.360987

C 2.419868 1.276395 0.943546

C -2.109546 -3.770689 1.530245

N -1.817058 -2.227368 -1.680408

C -1.630312 -1.067943 -2.492513

C 0.581831 -1.675018 1.541348

C 1.798670 -2.888059 2.989570

C 2.655848 -2.335319 2.096333

N -1.799159 -2.650055 2.391332

C -0.690565 -2.849508 3.297473

Fe -1.011078 -0.777656 0.788873

N 0.538517 -2.474442 2.619207

C -0.446355 -0.928608 -3.249975

N 1.890785 -1.602948 1.204251

C -0.190244 0.312097 -3.907772

C -1.097609 1.357571 -3.874494

C -2.319076 1.122694 -3.220940

C -2.623798 -0.097187 -2.542914

C 0.525200 -2.011900 -3.387965

C -0.817953 2.681383 -4.511226

C -3.995404 -0.266501 -1.993273

C 1.413072 3.722726 2.923584

C 0.631767 2.640735 3.328099

C -0.517248 2.349308 2.575631

C -0.896433 3.096307 1.451555

C -0.063786 4.157846 1.078237

C 1.086245 4.489473 1.798896

C 1.019949 1.803703 4.511696

C -2.141588 2.803988 0.666789

C 1.971197 5.618893 1.359336

C 3.239280 0.748425 -1.362903

C 2.693130 0.296173 -0.142952

C 2.443366 -1.074428 -0.003681

C 2.800129 -1.999503 -0.998012

C 3.391313 -1.499995 -2.173142

C 3.594163 -0.124398 -2.383074

C 4.153037 0.366911 -3.682310

C 2.552256 -3.466156 -0.838058

F -2.765190 -0.423201 0.525755

N -0.231512 0.130925 -0.050696

H -2.214077 2.335940 4.728593

H -3.555188 -0.088381 5.044023

H -3.151921 -5.057056 -0.732932

H -2.832794 -3.553862 -3.057105

H -3.713530 -1.841963 2.249324

H -3.275413 -2.354973 3.909807

H 3.304894 1.899995 1.118336

H 1.610275 1.958154 0.651101

H 2.140818 0.795308 1.882784

H -1.381007 -4.570706 1.694969

H -3.110215 -4.172710 1.725606

H 1.968441 -3.536770 3.840781

H 3.732466 -2.405730 1.998855

H -0.595615 -3.891753 3.623357

H -0.815128 -2.224197 4.187496

H 0.751443 0.417747 -4.445998

H -3.080970 1.902972 -3.217941

H 0.164462 -2.988344 -3.058368

H 0.933940 -2.057915 -4.403748

H 1.407275 -1.748757 -2.752652

H 0.133875 2.670191 -5.049513

H -1.617751 2.953673 -5.210036

H -0.774312 3.465277 -3.744200

H -4.254715 0.587233 -1.357166

H -4.706297 -0.270594 -2.832280

H -4.118488 -1.183381 -1.417373

H 2.310159 3.963675 3.494450

H -0.339787 4.754426 0.208157

H 2.032531 2.054151 4.841386

H 0.342215 1.961387 5.360558

H 0.989864 0.733644 4.268905

H -2.911905 2.319119 1.273811

H -2.554010 3.729156 0.251577

H -1.928200 2.134583 -0.177776

H 1.410249 6.368593 0.791195

H 2.446772 6.109677 2.215538

H 2.773851 5.243598 0.709422

H 3.407990 1.818227 -1.485857

H 3.696919 -2.210029 -2.941986

H 5.027143 -0.221157 -3.984842

H 3.401515 0.264546 -4.477313

H 4.439455 1.421172 -3.620691

H 3.257941 -3.911049 -0.124385

H 1.540680 -3.659843 -0.459492

H 2.672414 -3.983139 -1.794159

101

**2∙PF_6_ (PBE, SMD=MeCN)**

C -1.357300 4.580223 -2.507028

C -2.611848 4.146548 -2.857037

N -2.939181 3.143467 -1.968676

C -1.927045 2.937653 -1.075514

N -0.950221 3.818114 -1.413433

C -4.395797 -1.891346 -0.900040

C -3.217772 -2.584771 -0.744072

C -4.115038 2.273424 -2.051798

N -4.146831 -0.590805 -0.492399

C -2.865229 -0.464960 -0.069181

C 0.308171 3.127919 2.636822

C -5.101343 0.526864 -0.564592

N -2.283349 -1.688345 -0.245006

C -0.861964 -1.899787 -0.162568

C -2.864615 2.044402 1.543449

C -4.409510 3.237849 2.690037

C -3.414198 2.888900 3.569672

N -4.391149 1.796324 -0.697143

C -4.813007 2.842718 0.225803

Fe -1.964237 1.262027 -0.008605

N -4.052187 2.703167 1.468686

C -0.291388 -2.134033 1.118980

N -2.480319 2.148130 2.856030

C 1.116362 -2.171437 1.227400

C 1.940171 -2.011039 0.105131

C 1.321851 -1.870147 -1.162409

C -0.083032 -1.836234 -1.334965

C -1.167207 -2.344509 2.307296

C 3.437902 -1.988971 0.221161

C -0.653178 -1.762177 -2.722822

C 1.783804 5.327759 0.611536

C 0.512433 5.132215 0.036313

C 0.329993 3.991627 -0.778155

C 1.367277 3.064855 -1.033447

C 2.614256 3.299669 -0.402844

C 2.844666 4.418306 0.413445

C -0.610413 6.097451 0.304352

C 1.207518 1.871393 -1.930418

C 4.171598 4.632408 1.092166

C 0.934297 1.147113 4.052026

C -0.073952 1.895519 3.392889

C -1.405855 1.437638 3.497125

C -1.755013 0.320572 4.298050

C -0.713417 -0.363326 4.958108

C 0.640174 0.022234 4.833839

C 1.721161 -0.765719 5.521984

C -3.180974 -0.127034 4.457919

F -1.707610 0.681524 -1.723490

N -0.673225 0.999502 0.724863

H -0.721655 5.361748 -2.924485

H -3.280390 4.448947 -3.662348

H -5.377486 -2.214525 -1.249541

H -2.967133 -3.628313 -0.938791

H -3.879214 1.414780 -2.701989

H -4.946571 2.857981 -2.485562

H 0.926403 3.789286 3.271285

H 0.932809 2.859971 1.765537

H -0.559616 3.700094 2.278126

H -5.696623 0.545092 0.362442

H -5.773592 0.328937 -1.416903

H -5.329239 3.807382 2.831509

H -3.297403 3.085149 4.636061

H -5.887018 2.788496 0.474839

H -4.601215 3.830588 -0.215352

H 1.564196 -2.333869 2.215812

H 1.953495 -1.775411 -2.055655

H -2.005727 -3.029418 2.084754

H -0.596503 -2.732924 3.164203

H -1.626847 -1.384059 2.611583

H 3.769597 -2.209973 1.248918

H 3.903223 -2.719097 -0.466133

H 3.831773 -0.993650 -0.061304

H -0.091031 -1.049201 -3.347387

H -0.563328 -2.753205 -3.209322

H -1.706142 -1.455033 -2.753005

H 1.940338 6.208571 1.248054

H 3.428845 2.585428 -0.582197

H -0.361240 6.753961 1.153635

H -0.807912 6.742525 -0.572324

H -1.554213 5.571154 0.534561

H 0.352386 1.944772 -2.619302

H 2.130061 1.712476 -2.515619

H 1.063331 0.950609 -1.327538

H 4.954000 3.980427 0.669054

H 4.502817 5.682649 1.006893

H 4.093263 4.407085 2.173228

H 1.975696 1.479117 3.949104

H -0.970596 -1.238073 5.569389

H 1.545782 -0.811120 6.612629

H 1.735241 -1.810568 5.159060

H 2.717047 -0.326268 5.347496

H -3.723019 0.526495 5.168815

H -3.737647 -0.091485 3.505296

H -3.222697 -1.154193 4.854704

P -2.374103 0.984005 -5.267170

F -1.682398 1.863598 -6.459038

F -1.285114 -0.223909 -5.490395

F -3.389748 0.293574 -6.346856

F -3.473391 2.186937 -4.997055

F -1.362653 1.683599 -4.171557

F -3.090190 0.096001 -4.056615

108

**2∙2PF_6_ (PBE, SMD=MeCN)**

C -1.251528 5.062731 -2.312391

C -2.593973 4.863547 -2.497491

N -2.955960 3.836001 -1.646307

C -1.882397 3.406790 -0.921726

N -0.821877 4.157359 -1.342677

C -4.861348 -1.279739 -0.682772

C -3.805156 -2.104146 -0.382384

C -4.235694 3.124417 -1.728512

N -4.431463 0.012587 -0.447541

C -3.158605 0.011128 0.014972

C 0.161391 3.738029 2.487808

C -5.283582 1.205390 -0.554107

N -2.757242 -1.290848 0.037792

C -1.476634 -1.787959 0.475708

C -2.789687 2.355314 1.685028

C -4.309463 3.236194 3.095700

C -3.217552 2.821758 3.851564

N -4.489310 2.431132 -0.468444

C -4.827270 3.315927 0.639075

Fe -2.096807 1.657433 0.031396

N -4.013953 2.950597 1.798921

C -1.390065 -2.319212 1.781340

N -2.300264 2.295726 2.983073

C -0.125227 -2.756269 2.221493

C 1.013964 -2.706058 1.392188

C 0.857909 -2.247280 0.070173

C -0.382876 -1.807254 -0.427427

C -2.613371 -2.478551 2.640298

C 2.361124 -3.160024 1.889518

C -0.549570 -1.485811 -1.886992

C 2.361015 5.360108 0.184961

C 1.037893 5.361608 -0.291050

C 0.540073 4.159743 -0.860764

C 1.363341 3.021332 -1.032488

C 2.673028 3.074896 -0.510244

C 3.189891 4.222936 0.110483

C 0.230424 6.628520 -0.193450

C 0.956051 1.793598 -1.802336

C 4.577853 4.239254 0.695358

C 1.361491 1.662157 3.275980

C 0.156730 2.331550 2.993708

C -1.061291 1.686180 3.367762

C -1.082795 0.532146 4.187445

C 0.154594 -0.081778 4.447939

C 1.383268 0.446610 3.978997

C 2.678923 -0.237584 4.309546

C -2.342098 0.014545 4.823745

F -2.111473 1.192076 -1.778381

N -0.789737 1.186474 0.635788

H -0.564724 5.769722 -2.777324

H -3.311433 5.352856 -3.157784

H -5.874153 -1.504043 -1.018311

H -3.707489 -3.189521 -0.418313

H -4.165791 2.371248 -2.529101

H -5.019653 3.862542 -1.976793

H -0.096720 4.429078 3.313667

H 1.157121 4.013227 2.111389

H -0.587638 3.935989 1.708257

H -5.994865 1.172781 0.285935

H -5.837252 1.142853 -1.505893

H -5.230631 3.733404 3.397787

H -3.033759 2.894330 4.923503

H -5.885771 3.245556 0.936839

H -4.585671 4.360545 0.384578

H -0.034710 -3.161405 3.237606

H 1.720516 -2.252080 -0.609405

H -3.244913 -3.303994 2.259267

H -2.333951 -2.728687 3.675286

H -3.246505 -1.579301 2.648544

H 2.303273 -3.555857 2.916980

H 2.779640 -3.948996 1.237561

H 3.086780 -2.325538 1.882554

H 0.425623 -1.280213 -2.357661

H -0.992031 -2.356958 -2.409144

H -1.216189 -0.627030 -2.057801

H 2.747683 6.278834 0.645872

H 3.312538 2.189335 -0.624895

H 0.511113 7.192435 0.709469

H 0.430685 7.278582 -1.067128

H -0.852039 6.454759 -0.144415

H -0.058671 1.847502 -2.220195

H 1.662638 1.630190 -2.637091

H 1.016370 0.893914 -1.163061

H 5.206441 3.436986 0.272862

H 5.079226 5.208045 0.523248

H 4.538764 4.087795 1.791657

H 2.305716 2.131764 2.973270

H 0.163389 -1.001419 5.046617

H 3.024066 0.067920 5.317145

H 2.564267 -1.334558 4.330200

H 3.474702 0.031063 3.594775

H -2.585969 0.624956 5.714815

H -3.215533 0.055484 4.159531

H -2.203288 -1.020849 5.170706

P -6.042196 0.012678 3.086045

F -7.514373 -0.185801 3.749230

F -6.506682 -0.808238 1.741643

F -5.504447 -1.373326 3.769388

F -5.540761 0.869733 4.394774

F -6.539133 1.426345 2.371773

F -4.538245 0.230310 2.399073

P -2.799975 6.466474 2.161364

F -3.285827 7.736582 3.058379

F -4.371675 6.044625 1.880376

F -2.786432 5.506953 3.502001

F -1.227411 6.834370 2.440396

F -2.800719 7.389537 0.806829

F -2.327494 5.162631 1.236555

115

**2∙3PF_6_ (PBE, GP)**

C -1.080877 4.742393 -2.382526

C -2.413433 4.541356 -2.623392

N -2.847168 3.640940 -1.672529

C -1.820355 3.266377 -0.854013

N -0.726245 3.945750 -1.298675

C -4.918990 -1.255199 -0.689262

C -3.863036 -2.091593 -0.440973

C -4.131024 2.947351 -1.744777

N -4.492276 0.021526 -0.367426

C -3.229643 -0.006553 0.111475

C 0.010882 3.817277 2.662404

C -5.314569 1.226360 -0.435712

N -2.822711 -1.299726 0.035016

C -1.470422 -1.736621 0.252106

C -2.864860 2.393717 1.792840

C -4.420671 3.389424 3.079240

C -3.386970 2.956371 3.909391

N -4.472066 2.417003 -0.430670

C -4.785386 3.396726 0.594936

Fe -2.093349 1.604554 0.222352

N -4.068094 3.043712 1.818515

C -1.106290 -2.110058 1.566013

N -2.447076 2.366879 3.115183

C 0.246260 -2.403259 1.800453

C 1.201221 -2.364665 0.764835

C 0.765618 -2.081148 -0.542379

C -0.575841 -1.770208 -0.843808

C -2.152875 -2.265282 2.637095

C 2.661443 -2.613548 1.051123

C -0.999429 -1.546245 -2.274099

C 2.382445 5.161840 0.349143

C 1.087621 5.170950 -0.199431

C 0.607237 3.956488 -0.751141

C 1.415179 2.799766 -0.858080

C 2.692020 2.850926 -0.266228

C 3.188647 4.008953 0.356289

C 0.291571 6.449674 -0.199971

C 1.001066 1.571805 -1.626296

C 4.545243 4.016140 1.016573

C 1.198644 1.759974 3.526777

C 0.000181 2.430794 3.224466

C -1.215863 1.777407 3.568093

C -1.250095 0.613518 4.377047

C -0.016271 0.004172 4.657232

C 1.212205 0.529063 4.202669

C 2.500584 -0.208147 4.456056

C -2.521409 0.084360 4.982475

F -1.900411 0.977894 -1.459941

N -0.841600 1.154143 0.947194

H -0.347100 5.363391 -2.892839

H -3.056177 4.879953 -3.433967

H -5.931960 -1.461093 -1.031389

H -3.752898 -3.168724 -0.557087

H -4.026868 2.104413 -2.454713

H -4.881025 3.663579 -2.133582

H -0.432625 4.540341 3.369729

H 1.036291 4.135051 2.426046

H -0.597662 3.923922 1.753511

H -5.976093 1.235963 0.447201

H -5.922648 1.161859 -1.356886

H -5.330529 3.935873 3.316195

H -3.262365 3.069147 4.984531

H -5.859834 3.435236 0.844769

H -4.436608 4.399674 0.297668

H 0.558165 -2.681121 2.815816

H 1.490994 -2.102378 -1.366880

H -2.719274 -3.203554 2.480496

H -1.688794 -2.328255 3.632753

H -2.902422 -1.457713 2.643275

H 2.801010 -3.235186 1.952186

H 3.163616 -3.114256 0.205580

H 3.191911 -1.656696 1.222852

H -0.434810 -0.732520 -2.756718

H -0.809411 -2.462425 -2.863174

H -2.056191 -1.271428 -2.393727

H 2.762817 6.089983 0.795948

H 3.329761 1.958917 -0.331524

H 0.469373 7.017567 0.725070

H 0.593089 7.088229 -1.052644

H -0.793908 6.293443 -0.257037

H 0.173499 1.753536 -2.328588

H 1.864531 1.182897 -2.193551

H 0.680973 0.762999 -0.942734

H 5.217765 3.262320 0.572946

H 5.031478 5.003564 0.937090

H 4.457564 3.784695 2.096004

H 2.144779 2.232514 3.233817

H -0.016611 -0.913299 5.260578

H 2.519189 -0.663128 5.461779

H 2.620772 -1.030549 3.724999

H 3.376626 0.454131 4.356777

H -2.836783 0.718314 5.832675

H -3.368600 0.061004 4.284779

H -2.371930 -0.930603 5.381903

P -2.654686 1.379051 -4.806859

F -1.869370 2.116834 -6.018627

F -1.711594 0.051945 -4.912526

F -3.746975 0.755028 -5.827532

F -3.612735 2.725440 -4.581653

F -1.572656 2.039801 -3.727616

F -3.476492 0.660194 -3.525179

P -6.001141 0.159272 3.122340

F -7.465276 -0.006784 3.769589

F -6.436050 -0.676888 1.779244

F -5.445099 -1.191997 3.837343

F -5.495605 1.075352 4.398332

F -6.476336 1.573046 2.354631

F -4.474130 0.368001 2.441150

P -2.774734 6.427345 2.064886

F -3.254045 7.690654 2.943653

F -4.350932 5.986464 1.787152

F -2.771915 5.460800 3.421966

F -1.197632 6.753420 2.348427

F -2.758571 7.299471 0.690878

F -2.314435 5.082089 1.159536

115

**2∙3PF_6_ (PBE, SMD=MeCN)**

C -1.127813 4.829513 -2.349938

C -2.468707 4.640844 -2.564513

N -2.878557 3.690704 -1.653473

C -1.836425 3.289922 -0.866580

N -0.748148 3.987864 -1.308704

C -4.915647 -1.256608 -0.688697

C -3.854479 -2.092011 -0.433097

C -4.173380 3.008700 -1.725268

N -4.497235 0.021679 -0.369163

C -3.231712 -0.000957 0.108970

C 0.047873 3.747625 2.593383

C -5.325963 1.227312 -0.451170

N -2.819698 -1.296782 0.042066

C -1.474879 -1.733665 0.304551

C -2.859480 2.386688 1.787229

C -4.418616 3.329068 3.110917

C -3.372855 2.899157 3.923576

N -4.487375 2.423981 -0.425140

C -4.813228 3.379240 0.624014

Fe -2.115804 1.611952 0.194110

N -4.071158 3.016950 1.833925

C -1.150977 -2.112652 1.628716

N -2.428100 2.341619 3.106664

C 0.194747 -2.401137 1.908192

C 1.188938 -2.345773 0.907160

C 0.795990 -2.061880 -0.414692

C -0.538879 -1.767717 -0.757221

C -2.228427 -2.273580 2.665515

C 2.637563 -2.580060 1.245587

C -0.921355 -1.579556 -2.203596

C 2.403698 5.186893 0.279340

C 1.100164 5.200455 -0.246597

C 0.597443 3.988362 -0.787016

C 1.399445 2.827238 -0.897078

C 2.688888 2.870699 -0.330207

C 3.205840 4.028886 0.275960

C 0.314611 6.485477 -0.234420

C 0.970888 1.591693 -1.643509

C 4.570715 4.031604 0.914026

C 1.219838 1.677582 3.442249

C 0.024268 2.359402 3.151256

C -1.199863 1.737427 3.536484

C -1.234107 0.594026 4.375008

C -0.006208 -0.032377 4.640461

C 1.226052 0.465088 4.152175

C 2.503934 -0.272145 4.435994

C -2.500463 0.093058 5.010308

F -1.994156 1.030844 -1.537851

N -0.858245 1.114700 0.883969

H -0.410503 5.485352 -2.842913

H -3.147351 5.070996 -3.300791

H -5.925127 -1.465531 -1.043951

H -3.745374 -3.171843 -0.538284

H -4.088073 2.197589 -2.468138

H -4.925843 3.747301 -2.056439

H -0.270884 4.471686 3.366111

H 1.061844 4.015286 2.263108

H -0.648027 3.899990 1.756650

H -6.000301 1.233458 0.419458

H -5.922842 1.161993 -1.376800

H -5.341198 3.851042 3.363642

H -3.238305 2.984374 5.001972

H -5.885437 3.391685 0.879956

H -4.493513 4.392395 0.332561

H 0.472940 -2.684286 2.931614

H 1.550117 -2.073222 -1.213180

H -2.811862 -3.192702 2.461473

H -1.792875 -2.374006 3.671239

H -2.953108 -1.444360 2.667993

H 2.750157 -3.268177 2.101029

H 3.194658 -2.989691 0.385328

H 3.126610 -1.626753 1.526651

H -0.214112 -0.913014 -2.724480

H -0.880195 -2.557734 -2.720246

H -1.926990 -1.160922 -2.342097

H 2.797096 6.113131 0.719178

H 3.316495 1.971714 -0.398040

H 0.582848 7.089983 0.645575

H 0.545740 7.085646 -1.135545

H -0.771870 6.330046 -0.203200

H 0.099721 1.757385 -2.292888

H 1.809021 1.223661 -2.261600

H 0.718322 0.772157 -0.944724

H 5.223807 3.254887 0.480341

H 5.067850 5.011544 0.806469

H 4.492010 3.826152 1.999554

H 2.169648 2.128021 3.127651

H -0.009490 -0.943323 5.253001

H 2.675538 -0.360275 5.525317

H 2.454055 -1.303368 4.040230

H 3.374618 0.233032 3.986646

H -2.762331 0.727613 5.878729

H -3.361655 0.112101 4.330388

H -2.365575 -0.933518 5.385460

P -2.570595 1.438067 -4.980703

F -1.757728 2.164958 -6.200178

F -1.617893 0.106018 -5.100274

F -3.629484 0.803258 -6.054429

F -3.530644 2.770576 -4.807235

F -1.509197 2.088217 -3.900762

F -3.410022 0.706170 -3.750552

P -6.054267 0.025726 3.162556

F -7.529891 -0.208717 3.807553

F -6.482136 -0.807492 1.814365

F -5.489383 -1.345210 3.854130

F -5.594650 0.896809 4.477653

F -6.575418 1.427907 2.442322

F -4.546714 0.278275 2.497211

P -2.751704 6.500172 2.097919

F -3.206347 7.774390 3.006582

F -4.333260 6.114290 1.823716

F -2.749978 5.531056 3.432440

F -1.170259 6.834724 2.372817

F -2.736390 7.432098 0.750599

F -2.313821 5.193314 1.161114

115

**2∙3PF_6_ (TPSSh, SMD=MeCN)**

C -1.142065 4.890460 -2.403635

C -2.463804 4.669466 -2.628390

N -2.861192 3.716191 -1.709385

C -1.822923 3.343175 -0.920116

N -0.757615 4.062947 -1.349394

C -4.877484 -1.253440 -0.688253

C -3.824038 -2.079563 -0.430897

C -4.143076 3.013006 -1.780738

N -4.456349 0.031155 -0.386703

C -3.192514 0.011911 0.072451

C 0.159508 3.527580 2.452129

C -5.281955 1.238155 -0.492379

N -2.784481 -1.276229 0.023960

C -1.437875 -1.693359 0.308532

C -2.806180 2.365135 1.734052

C -4.377555 3.289352 3.044502

C -3.337304 2.831076 3.870422

N -4.431826 2.427484 -0.474158

C -4.745919 3.391050 0.565591

Fe -2.060298 1.618581 0.121033

N -4.013643 3.005238 1.779708

C -1.126761 -2.043170 1.635560

N -2.396851 2.289803 3.068725

C 0.216420 -2.290964 1.940780

C 1.220940 -2.209621 0.960386

C 0.845874 -1.950879 -0.366102

C -0.487782 -1.705566 -0.730486

C -2.218112 -2.203917 2.658708

C 2.672435 -2.386974 1.325613

C -0.861837 -1.548970 -2.182628

C 2.356010 5.256510 0.289074

C 1.063571 5.267239 -0.248984

C 0.583618 4.069302 -0.821555

C 1.392526 2.925133 -0.953389

C 2.671100 2.964604 -0.372907

C 3.164663 4.108596 0.266564

C 0.257614 6.540537 -0.227711

C 0.981130 1.706789 -1.739264

C 4.519390 4.106033 0.927675

C 1.208979 1.478917 3.450699

C 0.048671 2.183427 3.103125

C -1.194443 1.649400 3.523257

C -1.285826 0.553274 4.410579

C -0.091348 -0.092691 4.739597

C 1.160567 0.330700 4.248974

C 2.411252 -0.412046 4.628621

C -2.580192 0.103192 5.030128

F -1.965546 1.078806 -1.595544

N -0.799896 1.136762 0.762400

H -0.436487 5.554905 -2.882887

H -3.147173 5.080907 -3.358710

H -5.879885 -1.460387 -1.037847

H -3.713176 -3.151544 -0.522337

H -4.052215 2.203569 -2.508743

H -4.904741 3.733553 -2.097408

H 0.124885 4.306525 3.225144

H 1.112605 3.610975 1.928135

H -0.651096 3.753769 1.759248

H -5.958828 1.262314 0.362716

H -5.857257 1.166942 -1.419307

H -5.293188 3.802908 3.303318

H -3.228241 2.906476 4.944210

H -5.807524 3.424829 0.820784

H -4.394286 4.384189 0.277862

H 0.483390 -2.550135 2.964127

H 1.608218 -1.943490 -1.144905

H -2.808765 -3.103637 2.435729

H -1.794239 -2.321659 3.658676

H -2.916643 -1.362654 2.660482

H 2.788636 -3.019511 2.212217

H 3.239774 -2.830203 0.499436

H 3.127773 -1.412245 1.550760

H -0.149112 -0.906252 -2.708089

H -0.832974 -2.534533 -2.668098

H -1.857754 -1.128857 -2.320346

H 2.733118 6.168013 0.752050

H 3.302206 2.079424 -0.451163

H 0.561928 7.164439 0.615961

H 0.429120 7.112792 -1.149903

H -0.813336 6.356168 -0.136893

H 0.086686 1.872631 -2.341938

H 1.801649 1.410304 -2.403870

H 0.788649 0.855099 -1.074546

H 5.189072 3.373781 0.463180

H 4.989688 5.094635 0.877849

H 4.424168 3.840244 1.990162

H 2.170130 1.863193 3.112986

H -0.138869 -0.959294 5.397357

H 2.603776 -0.309833 5.705396

H 2.306066 -1.483854 4.422081

H 3.282088 -0.031004 4.086685

H -2.816588 0.730302 5.900565

H -3.421898 0.165434 4.341285

H -2.491371 -0.927603 5.383404

P -2.765548 1.245888 -5.064196

F -1.952147 1.821170 -6.349402

F -1.861050 -0.110009 -5.076519

F -3.848688 0.547811 -6.056219

F -3.677411 2.601392 -5.013232

F -1.684686 1.954839 -4.070385

F -3.598292 0.659516 -3.772650

P -6.079740 0.158926 3.126550

F -7.549431 -0.027680 3.771196

F -6.529497 -0.663029 1.796900

F -5.559847 -1.209429 3.824929

F -5.599812 1.021989 4.427616

F -6.557759 1.559414 2.408422

F -4.581445 0.366780 2.467176

P -2.638258 6.347677 2.326832

F -3.012750 7.518337 3.378756

F -4.221094 5.947240 2.192351

F -2.481216 5.280225 3.561240

F -1.058606 6.699101 2.461373

F -2.787401 7.374840 1.079173

F -2.281455 5.141236 1.259772

115

**2∙3PF_6_ (PBE0, SMD=MeCN)**

C -1.104055 5.192538 -2.340811

C -2.420860 4.972428 -2.575595

N -2.802945 3.960887 -1.723282

C -1.763052 3.556740 -0.967488

N -0.710490 4.309890 -1.346396

C -4.691922 -1.152738 -0.793148

C -3.647093 -1.966909 -0.495621

C -4.065366 3.244130 -1.846292

N -4.272452 0.133115 -0.541931

C -3.012431 0.132077 -0.090637

C 0.298514 3.501107 2.455061

C -5.110920 1.311450 -0.743292

N -2.611083 -1.153056 -0.070553

C -1.324084 -1.630746 0.338516

C -2.725276 2.395389 1.613906

C -4.313154 3.261029 2.926417

C -3.246530 2.816494 3.744868

N -4.344262 2.536491 -0.612042

C -4.708537 3.393061 0.484945

Fe -1.942693 1.777849 -0.066951

N -3.949590 3.001695 1.671466

C -1.191479 -2.123628 1.644083

N -2.317326 2.291805 2.937597

C 0.079375 -2.533839 2.056507

C 1.184695 -2.479559 1.201157

C 0.985717 -2.059553 -0.118473

C -0.266564 -1.651905 -0.586397

C -2.384320 -2.281554 2.535512

C 2.553023 -2.861781 1.682126

C -0.478633 -1.354287 -2.038978

C 2.369481 5.434292 0.370087

C 1.079720 5.452893 -0.160681

C 0.626099 4.297000 -0.825600

C 1.462470 3.191684 -1.046124

C 2.738992 3.220326 -0.470219

C 3.206125 4.318133 0.252785

C 0.244719 6.692470 -0.041216

C 1.096821 2.025298 -1.916454

C 4.556111 4.297785 0.905555

C 1.251717 1.435326 3.489986

C 0.125166 2.159129 3.087433

C -1.135022 1.632289 3.419312

C -1.287179 0.522407 4.275637

C -0.125423 -0.143532 4.661794

C 1.149158 0.273910 4.256551

C 2.366598 -0.483179 4.690333

C -2.608462 0.089539 4.832880

F -1.904900 1.355603 -1.818821

N -0.650901 1.401075 0.506362

H -0.410493 5.899183 -2.778933

H -3.113964 5.437852 -3.266349

H -5.688911 -1.374110 -1.152738

H -3.541156 -3.043697 -0.543910

H -3.964696 2.512679 -2.651139

H -4.843306 3.975542 -2.097993

H 0.296979 4.275015 3.233412

H 1.255328 3.552215 1.931189

H -0.493799 3.776973 1.760484

H -5.903417 1.286819 0.008026

H -5.549392 1.226778 -1.740714

H -5.247303 3.735025 3.204237

H -3.143312 2.874384 4.822511

H -5.768637 3.337737 0.748928

H -4.438897 4.431136 0.265660

H 0.204498 -2.909786 3.072023

H 1.824886 -2.064380 -0.815023

H -2.970798 -3.158237 2.225636

H -2.073937 -2.445876 3.570637

H -3.061720 -1.424240 2.498867

H 2.502958 -3.513483 2.561207

H 3.123322 -3.374737 0.898881

H 3.124271 -1.966637 1.964872

H 0.473276 -1.130471 -2.531502

H -0.906936 -2.235045 -2.538675

H -1.163865 -0.522835 -2.209150

H 2.724878 6.316057 0.904168

H 3.391590 2.358885 -0.617376

H 0.551161 7.274416 0.831112

H 0.373918 7.326001 -0.929606

H -0.819153 6.475184 0.064677

H 0.088746 2.086482 -2.329361

H 1.803811 1.961041 -2.753881

H 1.180802 1.082736 -1.361387

H 5.243278 3.619205 0.388004

H 5.004455 5.297446 0.932516

H 4.472666 3.950068 1.945187

H 2.235899 1.815290 3.215325

H -0.222198 -1.019180 5.303668

H 2.558417 -0.322156 5.760213

H 2.232505 -1.562048 4.548886

H 3.256303 -0.164155 4.137793

H -2.875517 0.712398 5.697821

H -3.424382 0.158394 4.114252

H -2.551834 -0.942972 5.187827

P -3.636933 0.504859 -4.893167

F -3.052630 0.649867 -6.394142

F -2.225445 -0.121187 -4.398628

F -4.184738 -0.981353 -5.222807

F -5.048861 1.139830 -5.367680

F -3.091927 2.001400 -4.542541

F -4.228152 0.369028 -3.376193

P -6.084407 0.189189 2.884571

F -7.557181 0.034595 3.509667

F -6.508878 -0.679925 1.589283

F -5.586753 -1.142755 3.647828

F -5.621962 1.102115 4.151823

F -6.540015 1.554501 2.108543

F -4.578872 0.367168 2.251625

P -2.495248 6.281723 2.619038

F -2.777170 7.375073 3.764772

F -4.082469 5.916604 2.556746

F -2.293630 5.129163 3.762590

F -0.912152 6.591344 2.684367

F -2.696228 7.389456 1.463269

F -2.230744 5.148792 1.461604

94

**a**

C -1.211512 0.533084 -4.910455

C -2.489361 0.070402 -4.749012

N -2.624897 -0.193424 -3.390421

C -1.473378 0.083302 -2.713931

N -0.598134 0.531267 -3.652743

C 0.771447 0.944612 -3.478309

C -3.676856 -0.960838 -2.753692

C 1.073255 2.322025 -3.617359

C 2.424932 2.697772 -3.535128

C 3.457848 1.753399 -3.327538

C 3.100967 0.395532 -3.194342

C 1.768536 -0.048695 -3.296352

C 1.450064 -1.519729 -3.321965

C 4.897431 2.184197 -3.307404

C -0.001546 3.355789 -3.844369

C -2.350805 -3.120942 2.287633

C -3.523875 -3.007798 1.593620

N -3.288206 -2.064080 0.599339

C -2.007673 -1.612195 0.646707

N -1.427923 -2.245634 1.702449

C -0.110560 -2.069204 2.273471

C -4.211829 -1.724074 -0.478393

N -3.654871 -0.633990 -1.308050

C -0.041648 -1.528582 3.579618

C 1.194458 -1.591985 4.257348

C 2.333538 -2.214955 3.694180

C 2.238894 -2.668395 2.368748

C 1.027205 -2.639686 1.634156

C 0.955877 -3.353734 0.314578

C 3.572020 -2.447415 4.513947

C -1.251675 -0.962359 4.277142

C -2.440394 2.678106 1.504568

C -3.586635 2.442940 0.798254

N -3.312084 1.384419 -0.070930

C -2.032163 0.975423 0.048494

N -1.479487 1.747292 1.053939

C -0.195624 1.463350 1.536939

C -4.192591 0.718017 -1.028842

C 0.258549 1.746795 2.825112

C 1.586315 1.335205 3.145997

C 2.507105 0.705395 2.262392

C 2.031608 0.354252 1.014284

C 0.728382 0.869208 0.498096

C 1.166498 2.115514 -0.399158

C 3.925366 0.449391 2.682438

C -0.547900 2.430195 3.901621

Fe -1.372317 -0.528342 -0.838642

F -1.332262 -2.186335 -1.677829

N 0.138710 -0.114358 -0.380884

H -0.678686 0.859265 -5.804732

H -3.288937 -0.092186 -5.474191

H -4.651781 -0.735882 -3.227975

H -3.449354 -2.034646 -2.856860

H 2.685415 3.758681 -3.643368

H 3.892961 -0.353930 -3.066371

H 2.308879 -2.110296 -2.965050

H 0.563398 -1.792048 -2.726330

H 1.252043 -1.845232 -4.361581

H 5.025857 3.178627 -2.847587

H 5.541042 1.459540 -2.782699

H 5.279506 2.263731 -4.343860

H -0.912843 3.153775 -3.252373

H 0.360647 4.364340 -3.588567

H -0.312101 3.392011 -4.905412

H -2.086928 -3.750038 3.138716

H -4.480200 -3.518629 1.721026

H -5.188987 -1.443177 -0.043851

H -4.360732 -2.618630 -1.107137

H 1.243093 -1.221189 5.290088

H 3.109873 -3.147401 1.902001

H 1.883806 -3.200419 -0.261695

H 0.887793 -4.444346 0.499189

H 0.101234 -3.069182 -0.316614

H 3.704526 -1.690171 5.304862

H 3.492811 -3.425268 5.027695

H 4.484319 -2.488080 3.896552

H -1.846517 -1.758275 4.762853

H -0.955748 -0.268239 5.079221

H -1.930751 -0.436006 3.583600

H -2.224521 3.452229 2.237025

H -4.550529 2.955487 0.831913

H -5.217088 0.682130 -0.615030

H -4.215239 1.297591 -1.969001

H 1.935264 1.558389 4.163840

H 2.649651 -0.181808 0.283354

H 1.888000 1.781229 -1.157773

H 1.626114 2.880790 0.247240

H 0.266041 2.517965 -0.889491

H 4.006686 0.237591 3.759966

H 4.375325 -0.383866 2.120050

H 4.535586 1.351270 2.483231

H -1.595423 2.089996 3.934886

H -0.104480 2.237475 4.891271

H -0.544612 3.528765 3.769383

94

**b (d)**

C 4.744061 0.452208 1.265138

C 4.607934 0.416367 2.629747

N 3.238538 0.359614 2.873086

C 2.531958 0.383642 1.713081

N 3.454272 0.424703 0.719267

C 3.156383 0.443721 -0.686875

C 2.514897 0.445626 4.131538

C 2.800266 1.684052 -1.273202

C 2.536382 1.682238 -2.654481

C 2.610365 0.499891 -3.434615

C 2.950476 -0.717053 -2.787019

C 3.244126 -0.769220 -1.415888

C 3.668186 -2.060035 -0.764629

C 2.402956 0.538240 -4.920803

C 2.715145 2.940884 -0.452155

C -3.238774 0.391857 3.577492

C -2.399206 0.213888 4.645664

N -1.113355 0.177404 4.113774

C -1.137427 0.345711 2.761888

N -2.444796 0.471055 2.425213

C -2.997960 0.791224 1.131810

C 0.151528 0.266237 4.825902

N 1.220529 -0.264474 3.941152

C -3.167320 2.163429 0.818201

C -3.870656 2.466022 -0.359622

C -4.374468 1.461131 -1.218554

C -4.132944 0.111219 -0.884289

C -3.472172 -0.254378 0.301914

C -3.346740 -1.699582 0.707892

C -5.169473 1.829175 -2.440117

C -2.633754 3.242570 1.722683

C 0.601393 -3.660702 0.967702

C 1.013176 -3.639258 2.272123

N 0.980896 -2.309098 2.678558

C 0.579359 -1.504710 1.669371

N 0.311997 -2.331882 0.596051

C -0.151275 -1.808365 -0.631767

C 1.353032 -1.745117 3.976951

C -0.130198 -0.377186 -0.765763

C -0.406467 0.263376 -2.100474

C -1.088416 -0.591928 -3.090860

C -1.179036 -1.962238 -2.849053

C -0.733154 -2.603536 -1.665066

C -0.982834 -4.080592 -1.565439

C -1.633541 0.002665 -4.339699

C -0.799591 1.743230 -2.046585

Fe 0.588154 0.342689 1.858599

F 0.752428 2.064575 2.502429

N 0.189614 0.444468 0.225692

H 5.636710 0.500960 0.639397

H 5.360272 0.437704 3.420545

H 3.120769 0.012430 4.949094

H 2.291378 1.502680 4.350380

H 2.285185 2.631119 -3.145192

H 3.019715 -1.636487 -3.382736

H 4.771212 -2.128437 -0.708395

H 3.329192 -2.929583 -1.350711

H 3.291154 -2.161632 0.267826

H 3.386735 0.563245 -5.430992

H 1.855438 1.437420 -5.247399

H 1.888130 -0.364712 -5.293055

H 1.982989 2.842419 0.372124

H 2.424511 3.801207 -1.074652

H 3.689562 3.183437 0.010706

H -4.325762 0.474497 3.529997

H -2.611897 0.121209 5.712357

H 0.086782 -0.281230 5.784541

H 0.374996 1.326929 5.028977

H -4.039929 3.520487 -0.613452

H -4.521554 -0.680656 -1.538187

H -4.120349 -1.968835 1.452460

H -2.370767 -1.924965 1.172085

H -3.495293 -2.367226 -0.156394

H -6.209149 2.082447 -2.154367

H -5.229914 1.000226 -3.164432

H -4.761536 2.722591 -2.943997

H -3.180990 3.268584 2.683931

H -2.745645 4.235346 1.260122

H -1.564339 3.087391 1.959136

H 0.527997 -4.508829 0.295050

H 1.324096 -4.461008 2.920960

H 0.698452 -2.173521 4.757244

H 2.395299 -2.031272 4.206516

H -1.662126 -2.591950 -3.606491

H -1.419228 -4.371510 -0.594241

H -1.675759 -4.405292 -2.356872

H -0.051310 -4.662455 -1.710055

H -1.959997 -0.762884 -5.060009

H -0.900721 0.669205 -4.831322

H -2.502004 0.648924 -4.096183

H -0.149544 2.284097 -1.343360

H -0.705606 2.213208 -3.037079

H -1.839024 1.846317 -1.700264

H 0.661673 0.256929 -2.538795

94

**b (q)**

C 4.730267 0.076834 1.185434

C 4.622380 0.033487 2.555609

N 3.271651 0.183814 2.830798

C 2.547741 0.339912 1.683408

N 3.446666 0.253277 0.671257

C 3.175087 0.395556 -0.740505

C 2.618333 0.355015 4.120390

C 3.084820 1.707147 -1.266451

C 2.911910 1.824344 -2.657729

C 2.838330 0.694466 -3.507045

C 2.911567 -0.591013 -2.920434

C 3.095628 -0.769444 -1.537862

C 3.252001 -2.151002 -0.954304

C 2.731288 0.854806 -4.997318

C 3.184397 2.920854 -0.380517

C -3.190444 0.397460 3.782338

C -2.295727 0.411866 4.827552

N -1.038207 0.465880 4.244963

C -1.128981 0.516386 2.882129

N -2.451953 0.439351 2.602668

C -3.026488 0.350586 1.280053

C 0.266963 0.540472 4.882607

N 1.226143 -0.130485 3.967090

C -3.149369 1.530206 0.512451

C -3.758878 1.405819 -0.753466

C -4.241898 0.175595 -1.242932

C -4.090472 -0.967872 -0.432118

C -3.496844 -0.908484 0.842939

C -3.420915 -2.137586 1.714708

C -4.952778 0.093018 -2.566479

C -2.688924 2.863510 1.038360

C 0.111832 -3.289666 0.868483

C 0.547439 -3.382864 2.165885

N 0.698031 -2.079317 2.623380

C 0.382846 -1.197675 1.649318

N -0.009572 -1.923405 0.558487

C -0.406554 -1.297563 -0.658426

C 1.107620 -1.614825 3.953562

C -0.164542 0.129279 -0.738025

C -0.279623 0.832537 -2.075997

C -1.069382 0.099018 -3.104524

C -1.403576 -1.225660 -2.889326

C -1.094486 -1.955476 -1.700466

C -1.568988 -3.380498 -1.649724

C -1.424050 0.789559 -4.374901

C -0.504062 2.347719 -1.985031

Fe 0.614995 0.654805 1.952727

F 0.916310 2.286111 2.601374

N 0.180908 0.857983 0.288910

H 5.606257 0.008107 0.537675

H 5.387669 -0.075609 3.327066

H 3.179192 -0.189180 4.903218

H 2.581496 1.427986 4.377201

H 2.857718 2.828442 -3.097557

H 2.856287 -1.477152 -3.566014

H 4.318444 -2.443869 -0.913199

H 2.746130 -2.904187 -1.580530

H 2.859542 -2.227500 0.074108

H 3.743537 0.847229 -5.447630

H 2.268955 1.814546 -5.282042

H 2.173835 0.026622 -5.466638

H 2.396357 2.929717 0.395203

H 3.095224 3.847211 -0.968663

H 4.157118 2.959420 0.143974

H -4.281811 0.373162 3.780570

H -2.460988 0.406841 5.907068

H 0.224835 0.067892 5.881811

H 0.576403 1.594682 4.986599

H -3.908951 2.313841 -1.352475

H -4.482272 -1.930658 -0.786171

H -4.220956 -2.136986 2.478677

H -2.462594 -2.219795 2.258795

H -3.563962 -3.055618 1.122075

H -6.046831 0.168414 -2.415668

H -4.772283 -0.868865 -3.076346

H -4.668999 0.915349 -3.243781

H -3.177458 3.107038 1.999627

H -2.932891 3.673917 0.333914

H -1.597755 2.882361 1.216464

H -0.081781 -4.087912 0.158465

H 0.762172 -4.265495 2.772238

H 0.352823 -1.940719 4.691899

H 2.072478 -2.083723 4.217097

H -1.958902 -1.759454 -3.669988

H -1.989801 -3.656039 -0.669135

H -2.349216 -3.545947 -2.408940

H -0.752091 -4.089847 -1.889076

H -1.892776 0.108131 -5.101252

H -0.532801 1.248850 -4.844622

H -2.120571 1.628339 -4.178546

H 0.171181 2.791542 -1.237758

H -0.306743 2.832742 -2.952888

H -1.542062 2.562391 -1.685385

H 0.772700 0.712223 -2.481961

94

**c (d)**

C 4.849959 -0.324965 1.374793

C 4.679757 -0.697377 2.679941

N 3.312049 -0.611731 2.931702

C 2.625922 -0.196253 1.833256

N 3.574647 -0.019709 0.869978

C 3.378130 0.676406 -0.372203

C 2.669191 -0.615717 4.245566

C 3.397270 2.069624 -0.342640

C 3.349579 2.798028 -1.637118

C 3.221652 2.023887 -2.901551

C 3.133746 0.642037 -2.825327

C 3.211783 -0.053218 -1.587233

C 3.147712 -1.542644 -1.576128

C 3.232784 2.752768 -4.198688

C 3.478888 2.837528 0.923530

C -2.998424 0.657603 3.869172

C -2.137040 0.459981 4.918400

N -0.884139 0.253616 4.345179

C -0.948572 0.336208 2.987083

N -2.247965 0.565846 2.692378

C -2.771992 0.717839 1.359198

C 0.411584 0.087038 4.976547

N 1.214808 -0.785363 4.073952

C -2.703431 2.000616 0.765765

C -3.255576 2.141070 -0.519460

C -3.820184 1.054017 -1.215991

C -3.831348 -0.210583 -0.588487

C -3.339284 -0.402289 0.714608

C -3.469759 -1.741943 1.395243

C -4.445322 1.239461 -2.572649

C -2.077132 3.162262 1.491498

C -0.374696 -3.737109 0.995939

C -0.020559 -3.868984 2.308389

N 0.389454 -2.608949 2.734743

C 0.291694 -1.702458 1.733128

N -0.176498 -2.387905 0.634976

C -0.421216 -1.744090 -0.604892

C 0.755867 -2.195689 4.085874

C -0.194325 -0.306458 -0.633216

C -0.409934 0.456275 -1.840410

C -0.831669 -0.217084 -3.002910

C -1.029302 -1.607907 -2.930172

C -0.843807 -2.407071 -1.775636

C -1.113611 -3.886510 -1.922570

C -1.067445 0.522512 -4.291192

C -0.171420 1.933588 -1.854065

Fe 0.713032 0.087507 2.010653

F 1.230897 1.734203 2.756891

N 0.240264 0.374558 0.439173

H 5.753007 -0.254392 0.766299

H 5.409993 -1.010508 3.428799

H 3.116220 -1.408604 4.873134

H 2.855713 0.360176 4.725461

H 4.271266 3.427879 -1.695839

H 3.021623 0.054036 -3.744826

H 4.171520 -1.955416 -1.699322

H 2.553975 -1.924150 -2.422791

H 2.755384 -1.939719 -0.626616

H 4.278610 3.029220 -4.453041

H 2.674432 3.705061 -4.156062

H 2.855704 2.135458 -5.029516

H 2.647456 2.542026 1.610350

H 3.452623 3.925687 0.760043

H 4.410816 2.591173 1.471213

H -4.071324 0.854770 3.853561

H -2.311979 0.460598 5.995711

H 0.288431 -0.344104 5.988095

H 0.920897 1.062846 5.038230

H -3.260096 3.136729 -0.982577

H -4.275097 -1.065268 -1.116111

H -4.424633 -1.808273 1.950294

H -2.663891 -1.936945 2.123213

H -3.479113 -2.561159 0.657249

H -5.543792 1.336056 -2.477563

H -4.261893 0.373114 -3.231664

H -4.087834 2.154626 -3.073226

H -2.631203 3.409476 2.416109

H -2.075928 4.066293 0.862624

H -1.035168 2.941702 1.791609

H -0.750158 -4.492521 0.315458

H -0.031708 -4.749291 2.953856

H -0.129469 -2.290557 4.740792

H 1.542766 -2.867450 4.474120

H -1.357415 -2.124841 -3.841449

H -1.967511 -4.222512 -1.306317

H -1.376828 -4.115467 -2.966747

H -0.235526 -4.508844 -1.674528

H -1.369891 -0.158705 -5.101862

H -0.160983 1.061522 -4.624211

H -1.860892 1.283701 -4.178229

H 0.199828 2.297246 -0.884132

H 0.536635 2.202871 -2.659210

H -1.109335 2.470822 -2.082177

H 2.566835 3.587766 -1.588244

94

**c (q)**

C 4.825510 -0.431216 1.556882

C 4.640219 -0.637752 2.901488

N 3.295629 -0.384929 3.145130

C 2.644828 -0.011662 2.008940

N 3.583752 -0.059040 1.028768

C 3.349813 0.306385 -0.344220

C 2.593044 -0.332322 4.427097

C 3.308187 1.653928 -0.668747

C 3.133479 2.021807 -2.098156

C 2.963953 0.942260 -3.108218

C 2.975614 -0.376900 -2.681553

C 3.182116 -0.727979 -1.321863

C 3.303366 -2.167981 -0.942578

C 2.844871 1.316183 -4.543387

C 3.449112 2.738012 0.346493

C -3.167857 0.299192 3.845616

C -2.334985 0.103012 4.924653

N -1.046649 0.120309 4.411755

C -1.057697 0.346118 3.061301

N -2.364368 0.428537 2.717084

C -2.865356 0.611980 1.372874

C 0.227237 0.020690 5.108032

N 1.170121 -0.643059 4.168210

C -3.011669 1.930377 0.888413

C -3.557567 2.077959 -0.399969

C -3.918131 0.972830 -1.196255

C -3.733226 -0.320956 -0.666014

C -3.237436 -0.531023 0.632353

C -3.179823 -1.919174 1.220099

C -4.537965 1.162450 -2.555054

C -2.607030 3.122180 1.716608

C 0.103514 -3.321027 0.644067

C 0.387417 -3.594076 1.959134

N 0.591442 -2.368602 2.573765

C 0.434547 -1.358435 1.683329

N 0.138209 -1.926316 0.475912

C -0.093860 -1.145621 -0.700022

C 0.885371 -2.090791 3.981487

C -0.022471 0.296233 -0.523839

C -0.305278 1.178638 -1.632485

C -0.636976 0.621444 -2.880514

C -0.669257 -0.783563 -3.012079

C -0.422875 -1.693294 -1.962855

C -0.560264 -3.162458 -2.289583

C -0.990247 1.493237 -4.054612

C -0.282467 2.663100 -1.411368

Fe 0.726029 0.419808 2.248510

F 1.175911 1.942988 3.079442

N 0.309342 0.872045 0.633398

H 5.726022 -0.507038 0.944753

H 5.351606 -0.925885 3.678429

H 3.065140 -1.036369 5.137435

H 2.654589 0.691855 4.833528

H 3.997078 2.665773 -2.394162

H 2.847565 -1.183175 -3.413829

H 4.374197 -2.460713 -0.942599

H 2.803302 -2.821342 -1.674627

H 2.921777 -2.380996 0.069338

H 3.857311 1.513819 -4.956091

H 2.273400 2.249413 -4.689406

H 2.403229 0.509953 -5.150284

H 2.605392 2.707456 1.065450

H 3.479065 3.737326 -0.114373

H 4.368988 2.605461 0.946874

H -4.256233 0.360604 3.788451

H -2.561649 -0.028044 5.984758

H 0.096622 -0.537659 6.053933

H 0.619276 1.029656 5.322911

H -3.724097 3.092882 -0.785059

H -4.025123 -1.192350 -1.266797

H -4.088986 -2.131872 1.814048

H -2.318666 -2.066408 1.894776

H -3.137806 -2.685415 0.428692

H -5.636508 1.043864 -2.494447

H -4.178984 0.410536 -3.279161

H -4.347305 2.169579 -2.960799

H -3.159608 3.164242 2.673192

H -2.812220 4.062794 1.181994

H -1.529136 3.098113 1.964393

H -0.127057 -4.015918 -0.155567

H 0.444527 -4.551579 2.480769

H 0.011865 -2.380833 4.592865

H 1.746532 -2.705557 4.300241

H -0.931673 -1.204061 -3.990823

H -1.382380 -3.641685 -1.726798

H -0.802435 -3.285993 -3.356423

H 0.365419 -3.738401 -2.108308

H -1.191028 0.896973 -4.958496

H -0.188854 2.215445 -4.295616

H -1.892630 2.095518 -3.842048

H 0.292123 2.937461 -0.511993

H 0.105013 3.209566 -2.288561

H -1.312016 3.036298 -1.253097

H 2.293259 2.747780 -2.179963

94

**d**

C 4.721541 -0.318520 1.093861

C 4.586440 -0.421357 2.449306

N 3.237392 -0.206449 2.722169

C 2.524843 0.044956 1.593301

N 3.443602 -0.027282 0.579640

C 3.215709 0.365222 -0.767284

C 2.632158 0.005587 4.033270

C 2.601833 1.693599 -0.973674

C 2.488546 2.122584 -2.368532

C 2.888042 1.338472 -3.449154

C 3.414255 0.057939 -3.140465

C 3.589964 -0.462596 -1.826106

C 4.153786 -1.847752 -1.649920

C 2.789840 1.810112 -4.872526

C 3.065657 2.823147 -0.000360

C -3.085733 1.006689 3.738512

C -2.176174 0.968174 4.763489

N -0.941456 0.742182 4.161792

C -1.059281 0.660355 2.805878

N -2.380453 0.803513 2.548155

C -2.998690 0.746658 1.247364

C 0.376494 0.689849 4.761295

N 1.182129 -0.249208 3.931281

C -3.151344 1.957842 0.530623

C -3.827184 1.893493 -0.701434

C -4.303766 0.678723 -1.233041

C -4.095584 -0.503443 -0.489731

C -3.477884 -0.493399 0.774309

C -3.411314 -1.753948 1.600327

C -5.068811 0.646000 -2.528798

C -2.637372 3.261755 1.082036

C -0.321456 -3.616126 1.270495

C 0.069050 -3.579535 2.579209

N 0.415899 -2.258726 2.850141

C 0.241696 -1.480321 1.756639

N -0.206603 -2.308005 0.754869

C -0.542653 -1.817336 -0.532792

C 0.793977 -1.669433 4.130732

C -0.443848 -0.373008 -0.717953

C -0.845523 0.243452 -1.965826

C -1.271257 -0.582807 -3.023236

C -1.279891 -1.974584 -2.818482

C -0.933983 -2.632881 -1.612730

C -1.010778 -4.141847 -1.615249

C -1.704443 -0.002993 -4.341263

C -0.825076 1.730344 -2.131831

Fe 0.599308 0.344038 1.818814

F 1.093888 2.047031 2.372727

N 0.053244 0.432025 0.232253

H 5.607456 -0.387298 0.461957

H 5.333749 -0.612530 3.222026

H 3.126064 -0.645030 4.778259

H 2.782140 1.059268 4.323294

H 2.056761 3.116886 -2.546027

H 3.707369 -0.592114 -3.978264

H 5.257566 -1.842186 -1.726474

H 3.791343 -2.516508 -2.448481

H 3.885625 -2.289299 -0.677355

H 3.797996 2.012934 -5.280418

H 2.205569 2.739938 -4.958368

H 2.333595 1.043028 -5.522625

H 2.757656 2.591712 1.031262

H 2.580547 3.771089 -0.282259

H 4.157249 2.956049 -0.073219

H -4.166573 1.155172 3.747023

H -2.306833 1.089527 5.840321

H 0.301960 0.366700 5.816793

H 0.852016 1.682331 4.701400

H -3.999312 2.826442 -1.254590

H -4.471236 -1.456260 -0.885784

H -4.344127 -1.884739 2.180821

H -2.581647 -1.752061 2.326916

H -3.314351 -2.645960 0.959086

H -6.156665 0.691778 -2.328754

H -4.891729 -0.286706 -3.091325

H -4.830873 1.507683 -3.174761

H -3.208418 3.573999 1.976554

H -2.733003 4.070640 0.340897

H -1.577044 3.191737 1.388377

H -0.677371 -4.461757 0.693151

H 0.118827 -4.380128 3.319735

H -0.069284 -1.722610 4.818843

H 1.622829 -2.250874 4.573710

H -1.591592 -2.610763 -3.657184

H -1.840956 -4.521316 -0.990924

H -1.203891 -4.503942 -2.636916

H -0.073037 -4.617962 -1.280244

H -1.984572 -0.789311 -5.059407

H -0.906668 0.608358 -4.801979

H -2.577281 0.663205 -4.214602

H -0.494196 2.256032 -1.224157

H -0.197315 2.025459 -2.992412

H -1.842837 2.093081 -2.363893

H 1.510052 1.522784 -0.644553

94

**e (Hopt)**

Fe 14.810755 7.613681 8.380735

F 15.215307 5.904319 8.848904

N 15.990839 8.254211 9.536990

N 13.256398 7.121723 6.894772

N 15.246642 6.885149 5.663971

N 13.414572 9.558201 6.986053

N 12.823110 7.938415 10.730798

N 14.842745 10.566601 8.310928

N 17.120598 7.752752 6.320087

N 12.087410 6.987594 8.928305

C 13.701411 8.541680 11.700748

C 13.187441 7.576095 9.476644

C 14.387019 9.340693 7.899583

C 18.939683 7.391804 7.913696

C 18.165498 8.301936 7.149234

C 15.861166 7.469751 6.731989

C 16.419293 9.486296 9.887291

C 15.884607 10.702555 9.275147

C 14.481909 7.672347 12.508008

C 13.929144 6.304883 5.852386

H 14.028146 5.272726 6.232402

H 13.373846 6.287086 4.895693

C 17.462278 9.555810 10.896617

C 17.300628 7.327392 5.004545

H 18.252641 7.447803 4.484076

C 13.726572 9.944520 11.825358

C 16.113382 6.773977 4.585545

H 15.837624 6.315734 3.633451

C 18.408791 9.688885 7.120999

C 15.311307 8.269050 13.475489

H 15.898092 7.618942 14.137353

C 14.108113 11.555300 7.629087

H 14.245189 12.620946 7.771528

C 12.221248 6.350186 7.630547

H 11.246900 6.329422 7.106862

H 12.587169 5.316260 7.759045

C 20.005810 7.928844 8.662849

H 20.650352 7.239599 9.224658

C 14.609590 10.482695 12.781082

H 14.657905 11.573470 12.897909

C 20.289888 9.309052 8.679632

C 11.034948 6.950301 9.832673

H 10.063632 6.502886 9.611557

C 15.391730 9.666632 13.632358

C 13.217891 10.919145 6.799801

H 12.480532 11.341665 6.113648

C 11.504558 7.556313 10.974792

H 11.016575 7.743064 11.933258

C 16.423242 11.950623 9.646818

C 18.659691 5.912497 7.895851

H 17.616207 5.680019 8.183706

H 19.332921 5.374076 8.580902

H 18.816144 5.485734 6.887123

C 12.731748 8.425104 6.359072

H 12.886623 8.470542 5.266210

H 11.647761 8.503172 6.557205

C 19.462027 10.169479 7.924872

H 19.671855 11.247298 7.926383

C 17.635633 10.620176 6.222215

H 18.077805 10.640197 5.207892

H 17.671487 11.655427 6.598909

H 16.579430 10.323260 6.103309

C 18.018785 8.301682 11.496922

H 17.255052 7.536427 11.716134

H 18.532403 8.509967 12.446622

H 18.770196 7.857391 10.812739

C 17.986981 10.815961 11.249707

C 17.452262 11.952440 10.620972

H 17.866892 12.929889 10.898546

C 14.401104 6.176242 12.355377

H 15.106094 5.670617 13.033622

H 14.619734 5.846877 11.321136

H 13.390269 5.800631 12.602229

C 12.812856 10.841016 11.029111

H 11.849811 10.979875 11.556223

H 12.574728 10.436275 10.030928

H 13.249545 11.845989 10.907068

C 16.230126 10.278715 14.719157

H 15.619266 10.414126 15.633187

H 16.604413 11.278802 14.442252

H 17.079791 9.635648 15.003453

C 21.478087 9.845752 9.429475

H 21.724055 9.234251 10.313982

H 21.334618 10.893886 9.741079

H 22.373174 9.829117 8.777813

C 19.095491 10.966868 12.254419

H 18.799411 10.581039 13.246516

H 19.381285 12.022740 12.380742

H 19.997344 10.408137 11.947518

C 16.027313 13.300842 9.097345

H 14.994866 13.584877 9.372165

H 16.129441 13.360784 7.999346

H 16.682522 14.079699 9.516438

H 16.461452 7.490311 10.048904

67

**e (Hopt, trunc)**

Fe 0.000000 0.000000 0.000000

F 0.000000 -1.817390 -0.042986

N 0.000000 0.000000 1.771950

N -0.035390 0.317701 -2.182647

N 2.247662 -0.041122 -1.745829

N -0.052191 2.525895 -1.136999

N -3.074698 0.134772 0.327132

N -0.005707 2.763579 1.043183

N 3.085683 0.141673 0.243959

N -2.295604 -0.083895 -1.682723

C -3.155352 0.222846 1.763059

C -1.921117 0.052675 -0.379579

C -0.004825 1.841959 0.028116

C 3.254545 -1.063436 2.364838

C 3.223747 0.178079 1.679407

C 1.911728 0.065646 -0.428324

C 0.022834 0.950549 2.731251

C 0.045601 2.378683 2.415377

C -3.151407 -0.993824 2.495373

C 1.198448 -0.319347 -2.710071

H 1.029471 -1.409263 -2.774074

H 1.482324 0.058153 -3.710845

C 0.044795 0.491147 4.109611

C 4.154042 0.061513 -0.648340

H 5.192880 0.084981 -0.311827

C -3.262546 1.490283 2.368230

C 3.625553 -0.056878 -1.912488

H 4.118815 -0.162529 -2.881499

C 3.379196 1.420435 2.324369

C -3.264708 -0.899544 3.894747

H -3.310486 -1.826057 4.482414

C -0.069023 4.046325 0.466408

H -0.087909 4.967067 1.051283

C -1.266900 -0.355529 -2.670817

H -1.584647 0.000916 -3.669143

H -1.074459 -1.442207 -2.721414

C 3.464478 -1.021574 3.757837

H 3.538523 -1.970061 4.306106

C -3.328093 1.520444 3.774483

H -3.428172 2.492148 4.275921

C 3.617120 0.192498 4.456902

C -3.677832 -0.114713 -1.806995

H -4.199289 -0.244394 -2.758177

C -3.355506 0.341012 4.555869

C -0.095357 3.892044 -0.897558

H -0.141387 4.651676 -1.682068

C -4.169146 0.026321 -0.529913

H -5.197652 0.050776 -0.163136

C 0.130791 3.312029 3.467778

C -0.061857 1.803779 -2.410466

H 0.817828 2.110003 -3.005234

H -0.968723 2.083291 -2.976822

C 3.542822 1.397827 3.724088

H 3.684905 2.350676 4.250947

C 0.132954 1.444145 5.144977

C 0.169525 2.802475 4.789409

H 0.237202 3.541831 5.599847

H -0.011531 -0.969059 2.139863

H -3.103710 -1.961770 1.983292

H -3.316836 2.405872 1.769069

H -3.470117 0.392084 5.644376

H 0.164058 4.392399 3.295720

H 0.165959 1.139356 6.196853

H 0.019296 -0.586121 4.310796

H 3.809204 0.200675 5.535574

H 3.403432 2.358048 1.758103

H 3.164198 -2.011976 1.823312

94

**e (PBE)**

Fe 0.349400 -1.705301 -1.128634

F -0.546342 -1.901888 -2.698348

N 2.099036 -2.494002 0.867070

N 1.310537 -0.517086 1.399345

N -0.348146 -0.094777 -0.888422

N -0.714356 -4.206231 -0.288191

N -2.051560 -2.721636 0.550284

N 1.391781 -3.642066 -1.171882

N 2.730594 -2.308711 -2.566064

N 2.663380 -0.145629 -2.460656

C 0.848000 1.054879 -4.334048

C 0.963056 5.251720 -1.480261

C 3.753213 1.251906 -0.141624

C 1.354686 3.818912 -1.707627

C 2.233675 3.164482 -0.812089

C 2.712000 1.861334 -1.045718

C 2.221296 1.198125 -2.187945

C 1.320625 1.794096 -3.109762

C 0.914832 3.114913 -2.845744

C -1.576481 -1.431977 3.128651

C 2.259415 -3.702381 0.055390

C 1.252272 1.650143 3.589309

C -1.930355 4.270559 0.789340

C -1.925291 2.201147 -1.343062

C -1.091285 3.027131 0.893829

C -1.077082 2.045607 -0.118222

C -0.280719 0.845944 0.077788

C 0.533080 0.672881 1.281238

C 0.507853 1.671537 2.275194

C -0.304235 2.807708 2.036570

C 1.260413 -1.514879 0.460640

C 2.710641 -2.144632 2.062870

C 2.222098 -0.906456 2.399295

C 0.330702 -4.681824 -1.176537

C -3.874289 -1.708311 -1.418648

C -4.952176 2.087522 1.774050

C -4.130988 0.878461 1.418726

C -3.179252 0.358979 2.323456

C -2.475309 -0.836590 2.074479

C -2.711983 -1.472095 0.840128

C -3.629278 -0.979921 -0.123662

C -4.331232 0.197907 0.200956

C -0.924466 -2.862345 -0.188690

C -1.712132 -4.919721 0.361252

C -2.557440 -3.974772 0.893933

C 2.184567 -3.647159 -2.429888

C 2.006032 -1.265522 -2.073427

C 3.828813 -1.860184 -3.287068

C 3.784208 -0.486842 -3.216387

H -0.964657 0.162117 -1.676199

H 0.405093 0.070727 -4.087747

H 1.683505 0.861520 -5.033068

H 0.098009 1.641656 -4.887333

H 0.016371 5.510635 -1.982867

H 1.736872 5.923922 -1.900020

H 0.886298 5.495921 -0.407002

H 3.660110 1.634633 0.888166

H 4.769409 1.523468 -0.485361

H 3.710525 0.150192 -0.112528

H 2.591859 3.706152 0.073345

H 0.247312 3.613065 -3.560684

H -0.741045 -2.015276 2.705418

H -1.162407 -0.648023 3.783751

H -2.146270 -2.118749 3.783223

H 1.989014 -4.583900 0.663782

H 3.319195 -3.799260 -0.240884

H 2.348604 1.702628 3.457239

H 1.013106 0.762032 4.200619

H 0.972842 2.529082 4.190175

H -1.817728 4.906505 1.681166

H -3.001708 4.023397 0.684923

H -1.653827 4.878862 -0.090549

H -1.446795 1.836266 -2.267504

H -2.176531 3.257194 -1.519429

H -2.883500 1.658312 -1.211721

H -0.320280 3.575730 2.820304

H 3.434053 -2.774156 2.585738

H 2.472898 -0.301108 3.262555

H -0.064933 -4.766333 -2.204091

H 0.698481 -5.671657 -0.846805

H -4.297257 -2.714686 -1.239192

H -4.594581 -1.163987 -2.048969

H -2.944383 -1.851815 -2.002473

H -4.425833 2.757482 2.474273

H -5.885693 1.772364 2.279382

H -5.258236 2.661109 0.882857

H -3.013160 0.877543 3.277059

H -5.081930 0.572216 -0.507955

H -1.755084 -6.010548 0.388241

H -3.473023 -4.086921 1.477726

H 1.497434 -3.856241 -3.268591

H 2.995648 -4.399436 -2.424173

H 4.534619 -2.524771 -3.789787

H 4.449108 0.269335 -3.637957

94

**e (PBE, CPCM=MeCN)**

Fe 14.833820 7.468031 8.416320

F 15.141742 5.727572 8.881158

N 16.026499 8.017610 9.591889

N 13.294300 7.078678 6.921133

N 15.255544 6.710382 5.709452

N 13.582861 9.500517 7.041271

N 12.793795 8.009613 10.694761

N 15.026281 10.412328 8.423602

N 17.100008 7.673305 6.300927

N 12.061469 7.038120 8.906849

C 13.659813 8.622278 11.665558

C 13.183362 7.545138 9.480041

C 14.519360 9.217822 7.965022

C 18.942006 7.648948 7.913589

C 18.091022 8.387828 7.061136

C 15.874615 7.312769 6.758360

C 16.509046 9.210352 9.995294

C 16.047271 10.469778 9.408540

C 14.413827 7.770675 12.507371

C 13.913044 6.197474 5.897514

H 13.952392 5.169519 6.294739

H 13.359365 6.208106 4.942540

C 17.532560 9.192491 11.023553

C 17.252756 7.269088 4.977602

H 18.175953 7.450981 4.425873

C 13.711430 10.029324 11.744648

C 16.078123 6.660398 4.599858

H 15.770640 6.207135 3.656420

C 18.179458 9.787996 6.904928

C 15.265814 8.381143 13.444054

H 15.861020 7.743000 14.109476

C 14.353550 11.447640 7.751811

H 14.533334 12.502256 7.924003

C 12.171437 6.397674 7.611417

H 11.222099 6.483288 7.055308

H 12.430198 5.333848 7.740242

C 19.904576 8.371197 8.641807

H 20.586877 7.821084 9.303115

C 14.601708 10.583797 12.681694

H 14.682933 11.676881 12.746252

C 20.016303 9.772024 8.545061

C 10.968432 7.155411 9.745083

H 9.974273 6.798169 9.472770

C 15.379866 9.781786 13.542144

C 13.454337 10.869540 6.888318

H 12.748826 11.331440 6.195628

C 11.435135 7.771097 10.883191

H 10.927063 8.060859 11.803820

C 16.645068 11.675189 9.825702

C 18.830605 6.149792 8.022623

H 17.822277 5.833056 8.348007

H 19.563087 5.757849 8.745845

H 19.015317 5.664888 7.046086

C 12.886259 8.413896 6.350220

H 13.141567 8.456007 5.277532

H 11.798327 8.555466 6.460927

C 19.141656 10.459270 7.679512

H 19.214165 11.551769 7.595860

C 17.324137 10.544757 5.920666

H 17.833706 10.604591 4.940301

H 17.150971 11.577852 6.264196

H 16.347283 10.067602 5.738246

C 18.020623 7.914415 11.635561

H 17.318897 7.067494 11.585016

H 18.248138 8.070356 12.702281

H 18.964486 7.601382 11.147465

C 18.119663 10.409399 11.409622

C 17.657156 11.593276 10.809904

H 18.122264 12.537410 11.119743

C 14.298501 6.271475 12.405898

H 14.952073 5.782211 13.145195

H 14.579533 5.904205 11.400652

H 13.259946 5.937876 12.587088

C 12.834111 10.922015 10.905171

H 11.923665 11.198915 11.469463

H 12.497144 10.442866 9.972064

H 13.358780 11.858817 10.652543

C 16.273864 10.422027 14.570527

H 15.678006 10.757848 15.440706

H 16.776914 11.315829 14.161786

H 17.038295 9.719517 14.942221

C 21.064002 10.528164 9.319203

H 21.549520 9.890817 10.075913

H 20.627195 11.408007 9.825512

H 21.849001 10.909547 8.639401

C 19.233607 10.456063 12.417072

H 18.916483 10.035715 13.388081

H 19.574127 11.490266 12.581267

H 20.095309 9.854575 12.075853

C 16.331651 13.052506 9.300873

H 15.302387 13.367958 9.549449

H 16.468892 13.119881 8.207200

H 17.014693 13.781148 9.763080

H 16.459334 7.212513 10.074002

94

**e (TPSSh)**

Fe 14.790303 7.560720 8.393737

F 15.135935 5.850596 8.804968

N 15.977355 8.172140 9.582790

N 13.273278 7.120965 6.927270

N 15.246433 6.908534 5.678182

N 13.443882 9.554800 7.045449

N 12.791914 7.909605 10.726263

N 14.881264 10.518278 8.377500

N 17.105472 7.774587 6.340808

N 12.074696 6.956954 8.931207

C 13.668428 8.561632 11.663822

C 13.162464 7.544199 9.485325

C 14.402222 9.316655 7.953175

C 18.933105 7.454597 7.924867

C 18.137278 8.343573 7.173346

C 15.860144 7.470777 6.749846

C 16.428271 9.380302 9.942806

C 15.924046 10.616802 9.342606

C 14.481193 7.746127 12.481211

C 13.933594 6.319941 5.861761

H 14.036073 5.291652 6.222532

H 13.372545 6.329150 4.920071

C 17.474057 9.418828 10.950984

C 17.284877 7.381198 5.017501

H 18.225899 7.524284 4.501329

C 13.672700 9.963251 11.718764

C 16.108928 6.828347 4.594785

H 15.832235 6.391908 3.642838

C 18.344115 9.730475 7.148033

C 15.323777 8.395625 13.392900

H 15.931350 7.792340 14.065673

C 14.171253 11.528593 7.701378

H 14.332195 12.580916 7.853356

C 12.224899 6.331671 7.630625

H 11.269928 6.325804 7.092358

H 12.592449 5.307862 7.751088

C 19.991652 8.010311 8.659476

H 20.654143 7.340460 9.207024

C 14.569679 10.558421 12.615247

H 14.599700 11.645259 12.684235

C 20.245551 9.391057 8.670748

C 11.017197 6.919943 9.828824

H 10.056670 6.473759 9.601658

C 15.384932 9.794992 13.477647

C 13.276626 10.921751 6.869580

H 12.555362 11.358733 6.189443

C 11.474240 7.528296 10.964612

H 10.983678 7.720654 11.910511

C 16.490192 11.838201 9.725641

C 18.690817 5.966703 7.900053

H 17.651482 5.714040 8.147257

H 19.347553 5.456186 8.609161

H 18.900370 5.551502 6.905537

C 12.744081 8.432992 6.412334

H 12.887552 8.490335 5.328564

H 11.674941 8.510555 6.634539

C 19.392518 10.232760 7.934151

H 19.580352 11.305925 7.930800

C 17.548883 10.647265 6.252154

H 18.032894 10.734476 5.270387

H 17.502050 11.656255 6.672517

H 16.528832 10.288975 6.077930

C 18.021842 8.153357 11.536211

H 17.285762 7.351428 11.646284

H 18.437163 8.339228 12.528953

H 18.842199 7.785323 10.901779

C 18.035239 10.654205 11.301588

C 17.523986 11.804755 10.690068

H 17.961906 12.761503 10.969920

C 14.419413 6.242104 12.395968

H 15.171116 5.785668 13.045253

H 14.579427 5.882102 11.370710

H 13.439452 5.869430 12.721059

C 12.713759 10.803811 10.913495

H 11.774701 10.941306 11.465684

H 12.458346 10.347851 9.951099

H 13.124197 11.801740 10.732818

C 16.231534 10.477948 14.515700

H 15.608619 10.747739 15.379838

H 16.665981 11.408825 14.134784

H 17.030310 9.828447 14.886178

C 21.442697 9.953593 9.390271

H 21.745165 9.326533 10.235003

H 21.262528 10.973808 9.744358

H 22.298212 9.998695 8.702542

C 19.166742 10.765758 12.287979

H 18.882360 10.373215 13.270796

H 19.471473 11.807242 12.418154

H 20.039781 10.196603 11.950134

C 16.119789 13.207805 9.203627

H 15.100304 13.498360 9.485840

H 16.222862 13.283128 8.115294

H 16.788909 13.954453 9.637265

H 16.426546 7.403405 10.090844

94

**e (TPSSh, CPCM=MeCN)**

Fe 14.824750 7.430698 8.420343

F 15.096250 5.695336 8.838433

N 16.025460 7.970382 9.618615

N 13.322287 7.069601 6.950256

N 15.269783 6.725499 5.721299

N 13.601203 9.487898 7.074569

N 12.779278 8.013426 10.689006

N 15.039392 10.377897 8.458289

N 17.097971 7.697371 6.308995

N 12.065154 7.031384 8.912570

C 13.649280 8.640164 11.646764

C 13.176733 7.534610 9.492014

C 14.529010 9.200191 7.990576

C 18.926728 7.705233 7.928169

C 18.074998 8.426341 7.073650

C 15.887811 7.318807 6.767012

C 16.511513 9.149033 10.019274

C 16.061442 10.416614 9.439279

C 14.400977 7.805709 12.495617

C 13.935470 6.193984 5.915883

H 13.989520 5.176153 6.310378

H 13.376101 6.210513 4.975880

C 17.538261 9.121820 11.044835

C 17.247098 7.313576 4.980987

H 18.156673 7.515509 4.430946

C 13.709958 10.041599 11.693295

C 16.085109 6.698717 4.606418

H 15.777259 6.256521 3.667998

C 18.146076 9.820017 6.910269

C 15.261752 8.426172 13.409231

H 15.851216 7.804066 14.080726

C 14.371715 11.423077 7.795159

H 14.556430 12.466209 7.978107

C 12.190328 6.381866 7.623201

H 11.259199 6.469393 7.056448

H 12.453326 5.330138 7.759495

C 19.876921 8.438123 8.651940

H 20.558396 7.904606 9.313404

C 14.609580 10.609925 12.604847

H 14.697466 11.694940 12.645619

C 19.975503 9.833938 8.541179

C 10.964164 7.165377 9.736601

H 9.979253 6.814756 9.457236

C 15.384776 9.823019 13.473431

C 13.477865 10.859232 6.931837

H 12.779847 11.323147 6.247013

C 11.418657 7.788083 10.865419

H 10.907215 8.093155 11.768699

C 16.672425 11.604004 9.854989

C 18.834465 6.204124 8.038630

H 17.830537 5.879466 8.339706

H 19.551978 5.831569 8.774789

H 19.050002 5.729178 7.072865

C 12.904925 8.402810 6.378446

H 13.163309 8.445862 5.316694

H 11.827030 8.536984 6.497314

C 19.097443 10.505668 7.676089

H 19.160724 11.589346 7.583578

C 17.282908 10.556395 5.916895

H 17.784583 10.594968 4.940709

H 17.112154 11.586347 6.243393

H 16.314304 10.072227 5.758228

C 18.013144 7.834115 11.648009

H 17.284464 7.020033 11.625813

H 18.280029 7.994398 12.695854

H 18.919303 7.495325 11.126120

C 18.140581 10.321771 11.424339

C 17.687658 11.508215 10.830867

H 18.161521 12.439249 11.136101

C 14.272622 6.304985 12.426317

H 14.929162 5.833432 13.162514

H 14.538861 5.924588 11.431546

H 13.240838 5.990320 12.627864

C 12.821716 10.919104 10.848833

H 11.915919 11.181329 11.411623

H 12.495964 10.428866 9.927006

H 13.333438 11.852592 10.594441

C 16.286478 10.480304 14.484923

H 15.693708 10.850352 15.332518

H 16.803457 11.342806 14.049031

H 17.029239 9.778005 14.875646

C 21.023688 10.604386 9.302172

H 21.523454 9.973340 10.042682

H 20.583073 11.468832 9.813696

H 21.785421 10.991982 8.612879

C 19.264914 10.356250 12.423011

H 18.945501 9.956486 13.392323

H 19.619368 11.379839 12.569028

H 20.104269 9.740958 12.078334

C 16.375979 12.988822 9.337558

H 15.361575 13.312314 9.598986

H 16.504258 13.051852 8.251203

H 17.073065 13.694622 9.794608

H 16.448213 7.170620 10.101294

94

**e (PBE0)**

Fe 14.813205 7.575519 8.388247

F 15.154290 5.874465 8.799408

N 16.000337 8.193258 9.563311

N 13.290267 7.129930 6.928882

N 15.254384 6.900764 5.687844

N 13.456373 9.548711 7.036840

N 12.818818 7.937871 10.723905

N 14.883151 10.518750 8.357472

N 17.112765 7.765520 6.324014

N 12.108181 6.991712 8.933731

C 13.682449 8.563520 11.681503

C 13.186954 7.576338 9.489773

C 14.417726 9.324531 7.931968

C 18.941365 7.459047 7.893872

C 18.146658 8.337397 7.137918

C 15.873812 7.477768 6.737343

C 16.435146 9.399314 9.917152

C 15.924683 10.626324 9.313772

C 14.457576 7.728301 12.506001

C 13.948487 6.322131 5.883882

H 14.051280 5.295589 6.254135

H 13.385784 6.310787 4.941222

C 17.474712 9.446147 10.928879

C 17.283819 7.350356 5.014459

H 18.223614 7.479405 4.489106

C 13.700580 9.959822 11.769132

C 16.106830 6.797557 4.607843

H 15.826404 6.345821 3.662473

C 18.354983 9.720680 7.100894

C 15.277914 8.349096 13.451273

H 15.860394 7.725174 14.129278

C 14.158161 11.518468 7.697941

H 14.300556 12.573882 7.855959

C 12.253843 6.360718 7.645483

H 11.294340 6.344501 7.112137

H 12.613857 5.333637 7.775132

C 19.988997 8.021058 8.629280

H 20.649824 7.357613 9.188327

C 14.571465 10.525528 12.704034

H 14.606520 11.611312 12.798761

C 20.237334 9.398640 8.635459

C 11.053640 6.955541 9.822984

H 10.090957 6.510716 9.595315

C 15.352282 9.741217 13.567350

C 13.269160 10.906290 6.870465

H 12.536879 11.339840 6.197576

C 11.507299 7.560701 10.956635

H 11.012522 7.752421 11.902291

C 16.486620 11.844632 9.686068

C 18.703484 5.977275 7.881413

H 17.668704 5.716487 8.140657

H 19.367948 5.469888 8.586265

H 18.906151 5.551077 6.889962

C 12.774294 8.428180 6.405362

H 12.930416 8.481314 5.321408

H 11.698700 8.509092 6.601804

C 19.395130 10.229162 7.884309

H 19.585042 11.302779 7.869635

C 17.562902 10.625375 6.201947

H 18.016954 10.668161 5.202609

H 17.558925 11.650596 6.584898

H 16.526553 10.296937 6.066395

C 18.002217 8.179469 11.507844

H 17.217300 7.462092 11.771593

H 18.569715 8.369486 12.420521

H 18.691060 7.701996 10.793780

C 18.013922 10.681912 11.291288

C 17.505695 11.820366 10.664640

H 17.934557 12.782408 10.944423

C 14.382630 6.233207 12.397362

H 15.106876 5.756268 13.063686

H 14.571128 5.876671 11.375476

H 13.391074 5.862651 12.689068

C 12.779594 10.827259 10.961364

H 11.839395 10.994425 11.504282

H 12.514760 10.388354 9.993605

H 13.218412 11.815234 10.789035

C 16.182627 10.386686 14.632898

H 15.555071 10.618330 15.504734

H 16.614822 11.335250 14.295655

H 16.984561 9.730776 14.986486

C 21.413956 9.966292 9.368486

H 21.711660 9.342883 10.217977

H 21.225486 10.985952 9.720885

H 22.281599 10.018859 8.696311

C 19.113974 10.810669 12.299440

H 18.814263 10.410036 13.274892

H 19.397143 11.856683 12.445097

H 20.010122 10.264534 11.982568

C 16.132634 13.200348 9.140713

H 15.126886 13.523418 9.438550

H 16.212995 13.250424 8.048997

H 16.823110 13.948536 9.537885

H 16.451179 7.426750 10.067743

94

**e (PBE0, CPCM=MeCN)**

Fe 14.846704 7.437612 8.413888

F 15.121059 5.707873 8.819958

N 16.066703 7.979052 9.591978

N 13.337792 7.076713 6.960055

N 15.269706 6.741987 5.721291

N 13.611713 9.480058 7.074305

N 12.810845 8.026669 10.694289

N 15.048795 10.373348 8.439086

N 17.103865 7.696634 6.285441

N 12.107021 7.037142 8.928563

C 13.659035 8.661094 11.656931

C 13.205886 7.552559 9.503764

C 14.540181 9.205335 7.981145

C 18.954062 7.663105 7.862680

C 18.094461 8.403067 7.038918

C 15.899502 7.334846 6.749049

C 16.538525 9.158813 9.981729

C 16.066906 10.419054 9.417078

C 14.404679 7.840241 12.516347

C 13.946129 6.212194 5.930004

H 14.007673 5.190609 6.318198

H 13.376836 6.219933 4.993763

C 17.569984 9.136644 10.999831

C 17.237791 7.306567 4.965328

H 18.144116 7.497985 4.403233

C 13.694523 10.059675 11.715981

C 16.070924 6.701497 4.605163

H 15.752540 6.257736 3.669241

C 18.168977 9.795934 6.909948

C 15.231455 8.469972 13.448639

H 15.819762 7.854251 14.129046

C 14.380554 11.409745 7.777268

H 14.565178 12.456217 7.948009

C 12.228666 6.390119 7.647335

H 11.289162 6.461485 7.088672

H 12.495600 5.337888 7.784062

C 19.901014 8.374686 8.602615

H 20.582097 7.824221 9.251940

C 14.557434 10.637250 12.650780

H 14.621756 11.724642 12.700348

C 19.993845 9.769293 8.535059

C 11.011264 7.160804 9.749388

H 10.027173 6.800666 9.473390

C 15.328290 9.863825 13.524597

C 13.485517 10.842836 6.924294

H 12.783686 11.303886 6.239063

C 11.459994 7.788967 10.872171

H 10.943718 8.091715 11.775378

C 16.648438 11.607005 9.851924

C 18.863857 6.166825 7.932909

H 17.869095 5.830219 8.252764

H 19.600208 5.770322 8.637882

H 19.052918 5.714937 6.950531

C 12.913425 8.397657 6.399318

H 13.146242 8.441559 5.329916

H 11.835005 8.531380 6.530215

C 19.120525 10.459361 7.687517

H 19.182894 11.546130 7.623112

C 17.308059 10.556840 5.944896

H 17.809370 10.628071 4.970135

H 17.134871 11.577535 6.299740

H 16.339136 10.080017 5.765523

C 18.032394 7.827455 11.544604

H 17.205089 7.255107 11.983237

H 18.777632 7.968214 12.327781

H 18.493770 7.211503 10.760865

C 18.116714 10.338263 11.430350

C 17.643203 11.518866 10.846493

H 18.083227 12.458362 11.178953

C 14.308061 6.344719 12.438707

H 14.950072 5.879664 13.192245

H 14.609571 5.966608 11.452881

H 13.278003 6.006057 12.609450

C 12.819770 10.925942 10.858772

H 11.904778 11.191558 11.405255

H 12.504849 10.435959 9.932620

H 13.330934 11.860965 10.606283

C 16.205412 10.525539 14.544781

H 15.600152 10.905214 15.378983

H 16.734901 11.383190 14.113567

H 16.941420 9.827674 14.956431

C 21.026944 10.516273 9.324442

H 21.492967 9.877818 10.081193

H 20.588307 11.389264 9.823093

H 21.819299 10.889572 8.662079

C 19.195821 10.400821 12.467428

H 18.873896 9.933334 13.404934

H 19.469322 11.438335 12.678275

H 20.094809 9.871987 12.128616

C 16.335715 12.986094 9.351407

H 15.311759 13.291088 9.598697

H 16.482621 13.071054 8.268597

H 17.009178 13.702131 9.828192

H 16.495089 7.179932 10.065843

94

**f**

Fe 0.372794 -1.651287 -1.046705

F -0.501588 -1.877224 -3.155148

N 1.952431 -2.598399 1.038416

N 1.246567 -0.581382 1.541705

N -0.292871 -0.170598 -0.714771

N -0.799414 -4.213367 -0.594207

N -2.176866 -2.773681 0.262191

N 1.456173 -3.696260 -1.073402

N 2.884408 -2.233440 -2.293894

N 2.669264 -0.076826 -2.353222

C 0.639212 0.827988 -4.267801

C 0.800262 5.349020 -2.008958

C 3.670921 1.582179 -0.208460

C 1.227035 3.907137 -2.057206

C 2.142602 3.393117 -1.113360

C 2.634362 2.079520 -1.184982

C 2.140076 1.258846 -2.224362

C 1.189257 1.708460 -3.168527

C 0.760611 3.044647 -3.068352

C -1.541169 -1.867579 2.954507

C 2.045333 -3.850203 0.289499

C 1.455114 1.763873 3.574393

C -1.851878 4.252644 0.750179

C -1.943664 2.066661 -1.240833

C -1.034059 3.004714 0.924680

C -1.088837 1.961322 -0.021484

C -0.281488 0.786314 0.213358

C 0.545002 0.648445 1.407810

C 0.609405 1.711281 2.324568

C -0.190339 2.849427 2.038463

C 1.199157 -1.567351 0.587657

C 2.481320 -2.299480 2.292383

C 2.042963 -1.044008 2.609748

C 0.362891 -4.673343 -1.333973

C -4.002543 -1.396985 -1.493394

C -4.674520 2.028007 2.192426

C -3.973856 0.808327 1.659093

C -3.034642 0.112771 2.452272

C -2.437128 -1.086480 2.025826

C -2.764201 -1.541630 0.730322

C -3.674937 -0.869944 -0.120297

C -4.276348 0.297449 0.380782

C -1.003479 -2.870652 -0.410243

C -1.852029 -4.955812 -0.070280

C -2.720551 -4.039999 0.471793

C 2.461085 -3.620804 -2.161276

C 2.037818 -1.210490 -1.955755

C 4.031557 -1.750077 -2.911905

C 3.893911 -0.383964 -2.944375

H -0.432960 0.595482 -4.081600

H 1.226668 -0.095936 -4.418717

H 0.642610 1.349602 -5.241169

H -0.187194 5.509066 -2.473626

H 1.519784 5.976085 -2.569974

H 0.779739 5.739032 -0.977377

H 3.710170 2.226504 0.684642

H 4.683994 1.598366 -0.652679

H 3.480839 0.544049 0.117859

H 2.513121 4.050265 -0.315766

H 0.055379 3.429467 -3.817206

H -0.779809 -2.463314 2.422495

H -1.027525 -1.198536 3.664605

H -2.133615 -2.576045 3.564049

H 1.494121 -4.636018 0.837206

H 3.104796 -4.156787 0.223129

H 2.525582 1.586379 3.368699

H 1.118490 1.043338 4.342560

H 1.382092 2.761692 4.033822

H -1.676372 4.971922 1.565244

H -2.931903 4.018292 0.732826

H -1.623401 4.758249 -0.205418

H -1.925545 1.147936 -1.844567

H -1.614023 2.913448 -1.869609

H -2.990181 2.276276 -0.958551

H -0.141917 3.677448 2.757722

H 3.114951 -2.985929 2.857573

H 2.234266 -0.464327 3.506035

H 0.153462 -4.679560 -2.418049

H 0.626011 -5.701551 -1.020215

H -4.524584 -2.370672 -1.441900

H -4.666096 -0.704372 -2.034318

H -3.094525 -1.552183 -2.105496

H -4.023177 2.623901 2.854333

H -5.550003 1.726316 2.799367

H -5.058442 2.674550 1.385705

H -2.795986 0.493494 3.454113

H -5.016178 0.815200 -0.243657

H -1.909899 -6.044489 -0.131228

H -3.671625 -4.180105 0.988642

H 1.989279 -3.957666 -3.101401

H 3.340420 -4.262836 -1.967425

H 4.831063 -2.393611 -3.284922

H 4.552326 0.389217 -3.344450

H -0.242328 -1.085078 -3.612771

98

**3 (Hopt)**

Fe 14.932354 10.199741 3.527760

N 14.203299 10.203349 5.574142

N 14.002731 7.944159 4.966508

N 15.219169 7.104882 3.420332

N 16.618101 10.195482 5.704921

N 17.871573 10.190311 3.932716

N 15.798445 10.196041 2.088764

N 13.029992 10.205589 2.833339

C 13.369396 8.981436 5.754767

H 13.290237 8.668741 6.812408

H 12.354941 9.192002 5.374749

C 14.755371 8.266748 3.897146

C 13.962877 6.570381 5.151951

H 13.396820 6.093334 5.954461

C 14.731651 6.041221 4.191979

C 15.395657 10.199969 6.492346

H 15.378054 11.095254 7.139416

C 16.585936 10.194798 4.369083

C 17.932316 10.191423 6.153547

H 18.205526 10.191167 7.210583

C 18.713198 10.188212 5.065880

C 16.194193 6.969874 2.363664

C 15.760204 6.977590 1.045105

C 16.720513 6.853950 0.052128

H 16.401881 6.826342 -0.997863

C 18.076839 6.731814 0.357812

C 18.460089 6.722236 1.693087

C 17.539538 6.832176 2.728746

C 14.289909 7.110408 0.697681

H 13.903741 8.109154 0.973133

H 13.675866 6.359423 1.226133

H 14.129630 6.968543 -0.382443

C 19.100052 6.603014 -0.737102

H 18.654525 6.716974 -1.738025

H 19.589720 5.612002 -0.704263

H 19.905864 7.351551 -0.622654

C 17.963205 6.763265 4.179749

H 19.023353 7.041996 4.296770

H 17.866343 5.733090 4.571171

H 17.359095 7.412231 4.836681

C 18.183865 10.188463 2.545542

C 17.062914 10.191619 1.645594

C 17.244374 10.190179 0.219207

C 18.540428 10.185621 -0.278521

C 19.597127 10.182681 0.628641

H 20.616806 10.179292 0.220428

C 19.479651 10.183894 2.026285

C 16.054826 10.193555 -0.689670

C 18.802174 10.183882 -1.764160

C 20.750483 10.180195 2.841822

C 11.991143 10.208722 2.351897

C 10.688123 10.210968 1.715593

N 14.017597 12.463090 4.963837

N 15.239526 13.292517 3.416675

C 13.377455 11.430935 5.753320

H 13.300415 11.745391 6.810594

H 12.361593 11.226742 5.373544

C 14.768095 12.134292 3.894860

C 13.986785 13.837318 5.147655

H 13.423813 14.318985 5.949577

C 14.759020 14.360274 4.187062

H 15.020021 15.394342 3.956334

H 15.372294 9.305573 7.140482

C 16.215413 13.419858 2.359851

C 15.781377 13.413438 1.041301

C 16.742475 13.529583 0.048182

H 16.423810 13.553313 -1.001786

C 18.099576 13.643153 0.353728

C 18.482887 13.651789 1.688991

H 19.547128 13.760593 1.939192

C 17.561636 13.549132 2.724776

C 14.310239 13.289887 0.694028

H 13.907184 12.307614 1.001836

H 13.706739 14.067164 1.196370

H 14.154389 13.399127 -0.390452

C 19.123611 13.763924 -0.741335

H 18.675720 13.661705 -1.742475

H 19.627096 14.747767 -0.703331

H 19.919238 13.003735 -0.631678

C 17.985753 13.616969 4.175699

H 19.043993 13.331266 4.293172

H 17.895854 14.648291 4.565795

H 17.377205 12.973019 4.833472

H 14.985911 5.005186 3.962502

H 19.523609 6.606512 1.943314

H 16.121717 11.024531 -1.414122

H 15.109640 10.282807 -0.135291

H 16.024637 9.264546 -1.287992

H 18.338177 9.308362 -2.252966

H 19.880170 10.164502 -1.987968

H 18.372973 11.080587 -2.247080

H 21.625267 10.177474 2.173025

H 20.839745 9.281790 3.478613

H 20.845078 11.078381 3.478151

H 19.795579 10.184588 5.004264

H 10.534041 11.156179 1.164223

H 9.887048 10.108501 2.469726

H 10.611679 9.366733 1.006471

98

**3 (PBE)**

Fe 14.976939 10.215827 3.592373

N 14.161016 10.218085 5.676529

N 14.090182 7.940614 5.049070

N 15.287854 7.131603 3.437767

N 16.577084 10.235037 5.872951

N 17.890947 10.216904 4.122920

N 15.901102 10.225319 2.197435

N 13.115376 10.190350 2.818316

C 13.376173 8.959917 5.792488

H 13.239250 8.639174 6.842274

H 12.380279 9.133229 5.348406

C 14.812666 8.295450 3.943559

C 14.085785 6.559313 5.229420

H 13.554531 6.059794 6.041722

C 14.845444 6.051717 4.207409

C 15.324354 10.264091 6.616799

H 15.281447 11.188561 7.220672

C 16.577717 10.228010 4.520294

C 17.880241 10.231084 6.365756

H 18.123318 10.237342 7.429929

C 18.703510 10.219461 5.274532

C 16.162072 6.998868 2.295669

C 15.584880 6.928019 1.007026

C 16.455562 6.742626 -0.080993

H 16.026740 6.645585 -1.087116

C 17.851771 6.647384 0.084209

C 18.375595 6.740078 1.390708

C 17.552063 6.887938 2.520466

C 14.095231 7.036572 0.802505

H 13.743065 8.064373 1.009933

H 13.534851 6.355621 1.467874

H 13.821986 6.787400 -0.234850

C 18.758728 6.390137 -1.088641

H 18.287587 6.661979 -2.047569

H 19.007424 5.313250 -1.148157

H 19.717435 6.929022 -0.995232

C 18.148090 6.858571 3.906385

H 19.172088 7.267398 3.902882

H 18.226642 5.818623 4.275774

H 17.552823 7.416965 4.648247

C 18.268798 10.203901 2.753507

C 17.170371 10.216249 1.794370

C 17.413746 10.211875 0.368829

C 18.748340 10.181275 -0.082822

C 19.773850 10.167622 0.877753

H 20.809987 10.144470 0.515585

C 19.593081 10.179861 2.286018

C 16.268037 10.212838 -0.588778

C 19.067241 10.162866 -1.550715

C 20.842341 10.167326 3.134745

C 12.103469 10.173416 2.232081

C 10.863173 10.150384 1.485741

N 14.000684 12.477617 5.014990

N 15.220484 13.299464 3.426518

C 13.315617 11.440693 5.760844

H 13.144423 11.770045 6.803021

H 12.338351 11.214421 5.300205

C 14.762169 12.130008 3.934464

C 13.953686 13.860862 5.176529

H 13.387490 14.355945 5.967674

C 14.727386 14.376504 4.168628

H 14.976339 15.409052 3.918090

H 15.289317 9.401885 7.307019

C 16.156028 13.420662 2.334044

C 15.658929 13.461823 1.011956

C 16.598873 13.595650 -0.024743

H 16.236226 13.661027 -1.058794

C 17.983452 13.673190 0.224656

C 18.424935 13.623774 1.564726

H 19.499459 13.704735 1.775762

C 17.530993 13.523266 2.643396

C 14.181187 13.387842 0.725299

H 13.745186 12.441909 1.096101

H 13.629985 14.214188 1.210534

H 13.986324 13.454125 -0.356562

C 18.971459 13.860004 -0.894097

H 18.526690 13.657629 -1.881936

H 19.338389 14.903825 -0.912011

H 19.860432 13.217766 -0.763112

C 18.031916 13.592580 4.064835

H 19.082113 13.264652 4.133718

H 18.004954 14.633961 4.437922

H 17.432023 12.987892 4.766356

H 15.114566 5.023329 3.960771

H 19.460237 6.651827 1.537495

H 16.382372 11.015336 -1.338410

H 15.302933 10.339827 -0.076770

H 16.243783 9.259305 -1.149193

H 18.611222 9.288825 -2.049668

H 20.152905 10.127948 -1.731608

H 18.668221 11.059918 -2.058101

H 21.733794 10.145344 2.489149

H 20.904079 9.276382 3.785487

H 20.933964 11.070249 3.765145

H 19.787179 10.213518 5.242253

H 10.731654 11.097640 0.931417

H 10.002893 10.019088 2.167369

H 10.869741 9.311503 0.765686

98

**3 (PBE, CPCM=MeCN)**

Fe 14.933287 10.204423 3.548907

N 14.148905 10.200213 5.619753

N 13.991656 7.942032 4.985355

N 15.232297 7.121163 3.418198

N 16.565556 10.184886 5.792597

N 17.855697 10.186233 4.023482

N 15.824870 10.209251 2.138639

N 13.091699 10.215309 2.812446

C 13.310958 8.976989 5.737600

H 13.179125 8.659792 6.786777

H 12.322058 9.183588 5.296201

C 14.741943 8.288693 3.904642

C 13.982934 6.567443 5.178341

H 13.432769 6.090056 5.990123

C 14.768779 6.046963 4.182502

C 15.321193 10.178132 6.554120

H 15.297276 11.062027 7.213799

C 16.547345 10.190747 4.447043

C 17.873552 10.176362 6.264109

H 18.119213 10.170253 7.326774

C 18.685364 10.177321 5.163985

C 16.183900 6.998431 2.345332

C 15.717622 6.997734 1.013049

C 16.678190 6.863948 -0.006294

H 16.340008 6.848993 -1.050793

C 18.054395 6.763661 0.273534

C 18.468347 6.775268 1.620405

C 17.551116 6.869696 2.680491

C 14.254658 7.162360 0.693674

H 13.932202 8.206008 0.866702

H 13.620048 6.519537 1.328907

H 14.056689 6.919548 -0.362471

C 19.071641 6.630324 -0.830051

H 18.611514 6.752985 -1.824210

H 19.559137 5.638192 -0.797205

H 19.872526 7.384043 -0.719370

C 18.026362 6.795885 4.109938

H 19.055090 7.182691 4.200138

H 18.037333 5.745556 4.458179

H 17.381915 7.352441 4.810204

C 18.201809 10.188720 2.649683

C 17.088059 10.202138 1.711635

C 17.300227 10.204079 0.283977

C 18.621994 10.183140 -0.193393

C 19.669868 10.173369 0.743415

H 20.698304 10.160552 0.359929

C 19.516408 10.176613 2.151673

C 16.134635 10.213338 -0.651645

C 18.904523 10.170386 -1.669529

C 20.774683 10.164531 2.982112

C 12.039086 10.219968 2.309076

C 10.739887 10.225325 1.679376

N 14.029955 12.468368 5.008934

N 15.262424 13.283075 3.432184

C 13.337554 11.438512 5.757827

H 13.221830 11.747366 6.811236

H 12.340272 11.256613 5.324595

C 14.763892 12.118777 3.918300

C 14.040671 13.841762 5.209882

H 13.505456 14.321186 6.030414

C 14.821418 14.358419 4.207948

H 15.112263 15.383954 3.978433

H 15.287520 9.269879 7.179422

C 16.190682 13.399569 2.338769

C 15.691054 13.410790 1.018253

C 16.627286 13.528988 -0.024855

H 16.263419 13.546924 -1.060472

C 18.011375 13.610015 0.221107

C 18.458789 13.591133 1.557111

H 19.535376 13.648954 1.765358

C 17.567318 13.506629 2.639993

C 14.217264 13.276177 0.735980

H 13.876424 12.240611 0.920575

H 13.612583 13.934265 1.384710

H 13.997752 13.520562 -0.315523

C 19.002340 13.734469 -0.907149

H 18.522923 13.583110 -1.888223

H 19.472037 14.735897 -0.907363

H 19.818646 12.997790 -0.797048

C 18.081767 13.565746 4.056585

H 19.095774 13.137089 4.120498

H 18.145503 14.615920 4.399396

H 17.433827 13.038117 4.775599

H 15.049970 5.019243 3.950733

H 19.538290 6.701192 1.856138

H 16.226510 11.041843 -1.376368

H 15.178376 10.311686 -0.116755

H 16.114027 9.277943 -1.241276

H 18.442691 9.290344 -2.153286

H 19.987787 10.148161 -1.868174

H 18.478160 11.063444 -2.161844

H 21.652455 10.158099 2.317622

H 20.839385 9.266409 3.622140

H 20.854366 11.059609 3.624629

H 19.768239 10.172861 5.117583

H 10.601707 11.164849 1.115628

H 9.954716 10.146037 2.452205

H 10.656601 9.366683 0.989802

98

**3 (TPSSh)**

Fe 14.953536 10.223217 3.582083

N 14.170452 10.228936 5.632929

N 14.115438 7.948260 5.042594

N 15.310597 7.144746 3.442228

N 16.581393 10.249352 5.829016

N 17.870308 10.225432 4.074217

N 15.859551 10.235443 2.175443

N 13.081105 10.185246 2.843307

C 13.394177 8.967142 5.777084

H 13.275634 8.666470 6.824503

H 12.404551 9.125037 5.337142

C 14.821849 8.299560 3.933911

C 14.132649 6.570207 5.236915

H 13.616314 6.077562 6.051346

C 14.890884 6.066797 4.222783

C 15.331549 10.298580 6.577179

H 15.288299 11.235922 7.141520

C 16.570110 10.243494 4.485225

C 17.889067 10.238229 6.308239

H 18.138959 10.243086 7.361841

C 18.695917 10.222435 5.215107

C 16.190657 7.024189 2.303148

C 15.623699 6.955997 1.018222

C 16.497742 6.779794 -0.061951

H 16.079018 6.683797 -1.063029

C 17.887772 6.691333 0.112544

C 18.403237 6.785824 1.416312

C 17.573606 6.926946 2.535593

C 14.134516 7.061530 0.806722

H 13.789528 8.084154 1.006986

H 13.580652 6.386528 1.469855

H 13.872522 6.808765 -0.224042

C 18.804177 6.436496 -1.055027

H 18.330888 6.691177 -2.008008

H 19.068626 5.371491 -1.097354

H 19.742015 6.995330 -0.961825

C 18.157604 6.903145 3.926933

H 19.175086 7.305855 3.925632

H 18.223218 5.872486 4.299528

H 17.558229 7.470299 4.647064

C 18.226638 10.210267 2.700679

C 17.115866 10.225020 1.759589

C 17.341108 10.220585 0.334260

C 18.661693 10.179100 -0.133689

C 19.695652 10.164770 0.810078

H 20.718804 10.136549 0.437607

C 19.535415 10.182885 2.217609

C 16.181782 10.229516 -0.607998

C 18.962384 10.158263 -1.607052

C 20.799096 10.174735 3.046239

C 12.068645 10.166379 2.276843

C 10.809766 10.139689 1.552103

N 13.979239 12.482042 4.986233

N 15.197662 13.301219 3.410827

C 13.305419 11.438551 5.731070

H 13.143856 11.755372 6.768082

H 12.339948 11.202784 5.273587

C 14.741127 12.138600 3.913626

C 13.927692 13.864397 5.145349

H 13.360435 14.352741 5.927877

C 14.701269 14.377687 4.147068

H 14.949716 15.401492 3.898066

H 15.296167 9.459415 7.279435

C 16.167231 13.402443 2.346632

C 15.719883 13.426726 1.014992

C 16.694712 13.533290 0.014495

H 16.374141 13.588279 -1.024809

C 18.064363 13.599319 0.314544

C 18.456876 13.561804 1.664768

H 19.515350 13.633573 1.912416

C 17.524828 13.486938 2.704716

C 14.252234 13.349664 0.679627

H 13.824546 12.396692 1.016718

H 13.687196 14.156082 1.162899

H 14.096830 13.434289 -0.398983

C 19.097751 13.761608 -0.767934

H 18.669558 13.624737 -1.764861

H 19.531011 14.769452 -0.730023

H 19.925088 13.053640 -0.635656

C 17.967962 13.560413 4.145205

H 19.008461 13.239529 4.250963

H 17.914396 14.593504 4.513062

H 17.344976 12.948840 4.808003

H 15.173420 5.049143 3.985398

H 19.478959 6.704230 1.568437

H 16.307618 11.011469 -1.364648

H 15.236831 10.385561 -0.085168

H 16.132918 9.272139 -1.142936

H 18.488458 9.298591 -2.094666

H 20.038080 10.104983 -1.793189

H 18.577082 11.059944 -2.098192

H 21.669779 10.157152 2.386407

H 20.869078 9.288483 3.688233

H 20.890380 11.072638 3.669134

H 19.770588 10.209515 5.168883

H 10.650972 11.098789 1.045358

H 9.980325 9.959684 2.246221

H 10.830935 9.334920 0.807658

98

**3 (TPSSh, CPCM=MeCN)**

Fe 14.919468 10.207086 3.545198

N 14.165051 10.205991 5.585774

N 14.014689 7.944774 4.979449

N 15.241068 7.128497 3.413958

N 16.576841 10.197761 5.757886

N 17.843203 10.191303 3.984966

N 15.793888 10.211850 2.126489

N 13.066966 10.208016 2.836959

C 13.332929 8.980468 5.729132

H 13.221090 8.678240 6.774516

H 12.349093 9.176048 5.295566

C 14.749875 8.288586 3.896221

C 14.015338 6.572281 5.177784

H 13.477610 6.100069 5.989304

C 14.792735 6.055415 4.184583

C 15.334975 10.203341 6.525496

H 15.310059 11.096711 7.155188

C 16.547540 10.199856 4.421205

C 17.888757 10.188644 6.216719

H 18.141362 10.186049 7.268764

C 18.684963 10.184524 5.115380

C 16.200710 7.020553 2.347271

C 15.748157 7.022317 1.017769

C 16.715186 6.902533 0.009951

H 16.390466 6.887969 -1.029765

C 18.083898 6.810992 0.303387

C 18.485715 6.826088 1.648340

C 17.559337 6.908839 2.694292

C 14.284946 7.169977 0.688523

H 13.948496 8.196722 0.879582

H 13.666104 6.504068 1.301187

H 14.106154 6.945233 -0.366745

C 19.110897 6.673446 -0.791469

H 18.660283 6.806192 -1.779586

H 19.577393 5.680208 -0.757534

H 19.912537 7.412142 -0.668751

C 18.018043 6.836880 4.129565

H 19.035524 7.227841 4.226746

H 18.027912 5.793385 4.472079

H 17.363422 7.390007 4.810455

C 18.169176 10.189868 2.608053

C 17.045095 10.203536 1.687949

C 17.239155 10.204614 0.261596

C 18.547231 10.179528 -0.230865

C 19.603216 10.168529 0.689570

H 20.618537 10.152886 0.296150

C 19.469103 10.174721 2.095630

C 16.059389 10.218027 -0.657433

C 18.812591 10.166924 -1.711363

C 20.740431 10.162032 2.906927

C 12.018887 10.205030 2.345474

C 10.710436 10.204417 1.724581

N 14.020414 12.468929 4.985303

N 15.245999 13.282272 3.417412

C 13.336413 11.433482 5.733447

H 13.226597 11.733195 6.779755

H 12.351528 11.241849 5.300515

C 14.753598 12.123556 3.901655

C 14.024036 13.841660 5.182805

H 13.488434 14.315448 5.994813

C 14.801129 14.356648 4.188154

H 15.088557 15.374092 3.959291

H 15.303992 9.312140 7.157974

C 16.201867 13.380856 2.346857

C 15.741466 13.387134 1.019976

C 16.704561 13.483874 0.006527

H 16.374214 13.500674 -1.031179

C 18.076607 13.549432 0.292575

C 18.485908 13.530062 1.635395

H 19.548252 13.573978 1.872781

C 17.563882 13.466220 2.686613

C 14.272355 13.279513 0.701869

H 13.888689 12.286969 0.967820

H 13.686549 14.017051 1.263606

H 14.100705 13.436207 -0.366721

C 19.099537 13.666947 -0.808136

H 18.641832 13.535651 -1.793196

H 19.579097 14.654220 -0.781753

H 19.892163 12.918567 -0.685772

C 18.030453 13.529842 4.119626

H 19.044066 13.127781 4.210918

H 18.052766 14.572308 4.464657

H 17.372737 12.982032 4.801998

H 15.078442 5.037355 3.956333

H 19.545671 6.762454 1.891994

H 16.147521 11.042084 -1.374897

H 15.121669 10.320147 -0.108360

H 16.029266 9.287518 -1.238498

H 18.341944 9.296089 -2.183891

H 19.886421 10.136296 -1.915428

H 18.392136 11.060284 -2.189390

H 21.597735 10.152085 2.229384

H 20.808223 9.270178 3.540956

H 20.827055 11.053964 3.538499

H 19.758754 10.178016 5.055522

H 10.560313 11.150092 1.192543

H 9.944375 10.092591 2.499900

H 10.643469 9.367651 1.020757

98

**3 (PBE0)**

Fe 14.944015 10.201547 3.545140

N 14.172973 10.194620 5.590339

N 14.009952 7.952715 4.954610

N 15.230675 7.127824 3.402787

N 16.569304 10.178995 5.771182

N 17.847609 10.187529 4.021396

N 15.823945 10.204555 2.140855

N 13.088278 10.222477 2.823664

C 13.337219 8.984714 5.703290

H 13.203050 8.667254 6.743525

H 12.355705 9.190321 5.265219

C 14.757782 8.286393 3.886223

C 13.984755 6.586799 5.143928

H 13.432780 6.115989 5.948671

C 14.760053 6.063478 4.157525

C 15.329107 10.162017 6.523081

H 15.304544 11.030185 7.189981

C 16.557156 10.185915 4.442116

C 17.867881 10.175876 6.242927

H 18.111618 10.169820 7.298624

C 18.673427 10.181312 5.153232

C 16.182716 7.007268 2.341842

C 15.729376 7.001948 1.016941

C 16.689767 6.873122 0.010076

H 16.361226 6.854625 -1.029310

C 18.054865 6.781692 0.297968

C 18.458570 6.801943 1.637447

C 17.538761 6.894211 2.682416

C 14.273893 7.157475 0.691077

H 13.943585 8.187732 0.877977

H 13.647144 6.500087 1.305234

H 14.088524 6.931096 -0.362948

C 19.074474 6.646478 -0.793776

H 18.621385 6.762814 -1.783271

H 19.557146 5.661318 -0.753212

H 19.866820 7.397479 -0.683289

C 18.001759 6.837542 4.109252

H 19.020131 7.229073 4.200516

H 18.014734 5.798553 4.465535

H 17.352830 7.396393 4.791403

C 18.187329 10.191257 2.654582

C 17.075266 10.198848 1.724684

C 17.288461 10.197786 0.303546

C 18.599197 10.181212 -0.172180

C 19.638129 10.180072 0.759438

H 20.659335 10.172294 0.378173

C 19.488350 10.185965 2.161081

C 16.131212 10.202001 -0.630453

C 18.882382 10.164667 -1.641478

C 20.744727 10.183197 2.982456

C 12.050375 10.237679 2.322130

C 10.753346 10.256035 1.695676

N 14.065848 12.451452 4.989716

N 15.277843 13.270211 3.427798

C 13.375297 11.426115 5.731906

H 13.262696 11.731054 6.778233

H 12.382483 11.255951 5.304353

C 14.791727 12.115440 3.907287

C 14.069104 13.815438 5.193505

H 13.537714 14.287832 6.011082

C 14.838824 14.334948 4.200666

H 15.123001 15.356095 3.977102

H 15.296055 9.251987 7.131279

C 16.196413 13.385696 2.337425

C 15.696607 13.397717 1.028791

C 16.621583 13.513382 -0.011678

H 16.256419 13.532484 -1.038637

C 17.996861 13.591920 0.228153

C 18.447356 13.568764 1.552386

H 19.517409 13.622372 1.755023

C 17.564594 13.483793 2.629792

C 14.228382 13.265265 0.752971

H 13.894266 12.233615 0.923321

H 13.630972 13.911078 1.407191

H 14.006791 13.522176 -0.286997

C 18.978076 13.719660 -0.899097

H 18.496966 13.564275 -1.869930

H 19.435079 14.718001 -0.902719

H 19.792746 12.992796 -0.792376

C 18.082859 13.529725 4.038107

H 19.085312 13.092736 4.093086

H 18.157245 14.569664 4.383731

H 17.436270 13.005666 4.749195

H 15.029887 5.039685 3.928308

H 19.520072 6.737243 1.878426

H 16.221164 11.031149 -1.342547

H 15.179912 10.287657 -0.102086

H 16.124880 9.276081 -1.220173

H 18.429277 9.285970 -2.117478

H 19.958739 10.145601 -1.835186

H 18.458124 11.049348 -2.132552

H 21.612389 10.183365 2.317736

H 20.818718 9.290667 3.615278

H 20.820053 11.073679 3.617903

H 19.749182 10.181565 5.109751

H 10.601091 11.220248 1.197855

H 9.981025 10.111997 2.459930

H 10.692369 9.446648 0.959434

98

**3 (PBE0, CPCM=MeCN)**

Fe 14.974764 10.206929 3.580470

N 14.184846 10.208472 5.642200

N 14.080882 7.950656 5.012100

N 15.270111 7.130951 3.429513

N 16.583393 10.212104 5.829575

N 17.873693 10.203180 4.089916

N 15.877865 10.212860 2.184691

N 13.090137 10.201836 2.838277

C 13.385375 8.973622 5.751216

H 13.240887 8.654315 6.791743

H 12.400654 9.159674 5.308411

C 14.809614 8.287835 3.923327

C 14.055613 6.578770 5.193107

H 13.515198 6.089977 5.996069

C 14.811606 6.063719 4.187987

C 15.339593 10.221496 6.572625

H 15.304815 11.118190 7.203052

C 16.580603 10.209128 4.494240

C 17.880700 10.209309 6.313311

H 18.128853 10.211723 7.368877

C 18.690496 10.203865 5.227084

C 16.169778 7.001070 2.318695

C 15.639802 6.952156 1.022422

C 16.538747 6.783460 -0.032518

H 16.147180 6.702950 -1.046787

C 17.918519 6.684684 0.176736

C 18.397469 6.754027 1.491034

C 17.541714 6.886961 2.585046

C 14.164862 7.072959 0.774813

H 13.815085 8.089433 0.998952

H 13.586791 6.378592 1.396488

H 13.926880 6.855754 -0.270196

C 18.861151 6.450823 -0.964020

H 18.406543 6.692052 -1.929802

H 19.153815 5.392908 -1.001894

H 19.784914 7.030079 -0.852966

C 18.082549 6.841333 3.985380

H 19.123529 7.178052 4.014673

H 18.076727 5.812411 4.369642

H 17.501973 7.447916 4.689163

C 18.234637 10.196774 2.724872

C 17.129509 10.207431 1.781141

C 17.362766 10.207625 0.359126

C 18.682494 10.184689 -0.101331

C 19.705165 10.173457 0.846667

H 20.731349 10.157147 0.478600

C 19.540317 10.180337 2.251003

C 16.219353 10.212830 -0.588342

C 18.991677 10.173785 -1.564902

C 20.795055 10.167962 3.079467

C 12.091382 10.204068 2.257315

C 10.851062 10.207806 1.516529

N 14.053405 12.460617 5.000997

N 15.247847 13.280515 3.422210

C 13.368097 11.433154 5.743436

H 13.216816 11.755506 6.782078

H 12.387273 11.231869 5.298833

C 14.794155 12.123252 3.921042

C 14.013081 13.833714 5.171281

H 13.461655 14.323157 5.966327

C 14.772550 14.348863 4.168524

H 15.022202 15.375180 3.924403

H 15.306147 9.343325 7.228680

C 16.169905 13.399891 2.329319

C 15.669395 13.433549 1.021211

C 16.594240 13.571983 -0.015507

H 16.227495 13.635416 -1.039990

C 17.969683 13.660044 0.224355

C 18.418212 13.611439 1.550774

H 19.485919 13.699929 1.753338

C 17.536464 13.504972 2.626156

C 14.198083 13.333996 0.745117

H 13.804141 12.358523 1.058827

H 13.631163 14.106706 1.279136

H 13.993292 13.456278 -0.322057

C 18.943831 13.857225 -0.896448

H 18.492704 13.660083 -1.873661

H 19.302203 14.895174 -0.909911

H 19.828017 13.219591 -0.779318

C 18.043028 13.566712 4.038577

H 19.088154 13.247000 4.096101

H 18.011169 14.597308 4.417045

H 17.454944 12.954542 4.731510

H 15.068334 5.038164 3.948113

H 19.468707 6.658988 1.670782

H 16.331224 11.020567 -1.320856

H 15.262917 10.327545 -0.076039

H 16.202610 9.273050 -1.155995

H 18.537386 9.307553 -2.060919

H 20.068952 10.138763 -1.748795

H 18.596167 11.069308 -2.059824

H 21.670817 10.151679 2.425707

H 20.867315 9.279032 3.717764

H 20.892020 11.063053 3.705826

H 19.766744 10.201075 5.190410

H 10.718537 11.172186 1.011230

H 10.004214 10.042579 2.193756

H 10.863619 9.407216 0.766772

94

**[TS**-2a**]**

C -1.228144 0.395769 -4.861944

C -2.485150 -0.087137 -4.630259

N -2.570865 -0.290634 -3.258087

C -1.404352 0.047229 -2.629189

N -0.576669 0.469368 -3.625751

C 0.792783 0.912934 -3.550184

C -3.661693 -0.982142 -2.583310

C 1.061887 2.281337 -3.798916

C 2.409374 2.671766 -3.840999

C 3.473920 1.748218 -3.649607

C 3.149757 0.398536 -3.398601

C 1.821619 -0.061018 -3.373562

C 1.517871 -1.529566 -3.283843

C 4.896301 2.202082 -3.760823

C -0.040910 3.288243 -4.007154

C -2.328846 -3.247616 2.185330

C -3.512337 -3.021576 1.538685

N -3.277657 -1.975121 0.653837

C -1.983692 -1.559393 0.726387

N -1.396893 -2.336351 1.682515

C -0.076120 -2.228859 2.261723

C -4.309303 -1.409848 -0.231919

N -3.746930 -0.487390 -1.208231

C 0.001354 -1.742075 3.588867

C 1.251554 -1.814105 4.251488

C 2.396846 -2.382138 3.647750

C 2.281225 -2.804892 2.315104

C 1.052521 -2.778342 1.602591

C 0.986658 -3.439938 0.259535

C 3.661330 -2.601940 4.430874

C -1.210961 -1.257079 4.335945

C -2.274427 2.982376 1.298023

C -3.412928 2.743039 0.577104

N -3.178728 1.583493 -0.160365

C -1.926989 1.108689 0.054864

N -1.370910 1.960101 0.982569

C -0.116859 1.676878 1.589913

C -4.115361 0.917793 -1.075755

C 0.061735 1.685196 2.978847

C 1.337857 1.284374 3.464410

C 2.424816 0.890257 2.634078

C 2.200097 0.850490 1.265750

C 0.955773 1.307729 0.672378

C 1.162541 2.191089 -0.552301

C 3.781190 0.627326 3.221338

C -0.985058 2.134141 3.963635

Fe -1.192904 -0.485206 -0.703044

F -1.247650 -2.143383 -1.523886

N 0.249473 -0.153358 -0.213291

H -0.732975 0.687685 -5.789018

H -3.306443 -0.301306 -5.316818

H -4.592298 -0.821794 -3.163467

H -3.433147 -2.060899 -2.543750

H 2.648576 3.726150 -4.030955

H 3.960438 -0.330747 -3.275431

H 2.392123 -2.088742 -2.914443

H 0.645719 -1.766428 -2.652447

H 1.292501 -1.925997 -4.293996

H 5.056190 3.179724 -3.272175

H 5.605692 1.467667 -3.347733

H 5.161283 2.349372 -4.827936

H -0.911608 3.096743 -3.354861

H 0.318565 4.311190 -3.812012

H -0.410039 3.275831 -5.049744

H -2.064424 -3.975364 2.953525

H -4.482501 -3.512527 1.636260

H -5.053536 -0.887257 0.395778

H -4.826912 -2.251206 -0.727774

H 1.305968 -1.483526 5.297312

H 3.152529 -3.250626 1.817065

H 1.824050 -3.105081 -0.378576

H 1.120708 -4.532869 0.382790

H 0.045463 -3.269880 -0.281896

H 3.756097 -1.908393 5.282407

H 3.659773 -3.625024 4.854144

H 4.562707 -2.521673 3.801000

H -1.785937 -2.106024 4.752374

H -0.923512 -0.632186 5.195472

H -1.904204 -0.686687 3.694406

H -2.026475 3.802664 1.970578

H -4.348181 3.303767 0.523379

H -5.139553 1.057639 -0.680878

H -4.062118 1.402932 -2.066341

H 1.497776 1.314931 4.550209

H 3.009132 0.572464 0.578247

H 1.852414 1.700351 -1.253565

H 1.620537 3.143464 -0.226169

H 0.216799 2.416489 -1.067022

H 3.720702 0.275601 4.262803

H 4.351339 -0.105624 2.628410

H 4.367152 1.566435 3.234554

H -2.008213 1.848582 3.669424

H -0.784726 1.718465 4.963059

H -0.966367 3.234444 4.081030

94

**[TS**-ab**]**

C -1.137294 0.425461 -4.782850

C -2.389300 -0.115391 -4.660899

N -2.562265 -0.361636 -3.303471

C -1.456958 0.000853 -2.589244

N -0.580312 0.485919 -3.501168

C 0.760585 0.969482 -3.273559

C -3.615052 -1.131270 -2.671055

C 0.975104 2.369571 -3.265446

C 2.306496 2.818769 -3.160677

C 3.396757 1.922397 -3.097167

C 3.122376 0.538062 -3.100230

C 1.813369 0.026143 -3.201975

C 1.560651 -1.455297 -3.298257

C 4.812037 2.432987 -3.075648

C -0.168967 3.344808 -3.386564

C -2.441244 -3.315358 2.245375

C -3.631984 -3.130022 1.593636

N -3.392283 -2.151860 0.632954

C -2.100775 -1.740428 0.674461

N -1.504034 -2.456395 1.663341

C -0.131166 -2.396900 2.100431

C -4.336102 -1.671831 -0.369658

N -3.673497 -0.688417 -1.257223

C 0.167467 -1.643260 3.262230

C 1.479341 -1.723555 3.763822

C 2.464529 -2.544739 3.167793

C 2.121504 -3.253805 1.996330

C 0.826239 -3.224056 1.451800

C 0.453113 -4.109357 0.296032

C 3.816711 -2.722874 3.799950

C -0.888225 -0.847701 3.988424

C -2.491370 2.894426 1.223031

C -3.612936 2.597138 0.501287

N -3.344482 1.410872 -0.176892

C -2.088140 0.975055 0.076288

N -1.554584 1.869190 0.978053

C -0.279029 1.674121 1.546862

C -4.182216 0.696653 -1.132454

C 0.177334 2.289321 2.721564

C 1.516113 2.021935 3.145031

C 2.396660 1.138753 2.518016

C 1.903310 0.441012 1.381286

C 0.589447 0.724830 0.829741

C 1.790863 1.728755 -0.150338

C 3.795272 0.908841 3.002571

C -0.651936 3.183341 3.607586

Fe -1.401289 -0.587063 -0.695945

F -1.255497 -2.219161 -1.585907

N 0.078470 -0.230333 -0.050002

H -0.591298 0.775879 -5.660207

H -3.149393 -0.338587 -5.411973

H -4.572350 -0.977851 -3.206343

H -3.341933 -2.200136 -2.677670

H 2.499215 3.899593 -3.162759

H 3.959411 -0.171770 -3.065173

H 2.453238 -2.025281 -2.995575

H 0.705192 -1.787444 -2.684771

H 1.338741 -1.741429 -4.344406

H 4.896843 3.399252 -2.550803

H 5.507613 1.712261 -2.615122

H 5.165363 2.601645 -4.111134

H -1.030463 3.060205 -2.754569

H 0.145324 4.362761 -3.106689

H -0.540485 3.399929 -4.427130

H -2.174800 -3.984258 3.065212

H -4.603489 -3.607916 1.733367

H -5.206285 -1.215947 0.135730

H -4.700620 -2.531984 -0.959417

H 1.728805 -1.170306 4.678776

H 2.869804 -3.905689 1.527016

H 1.351703 -4.496670 -0.209331

H -0.114851 -4.988460 0.658055

H -0.177644 -3.590913 -0.444505

H 4.030271 -1.958695 4.564512

H 3.864330 -3.706439 4.306811

H 4.629083 -2.725991 3.051869

H -1.476729 -1.497638 4.663644

H -0.431554 -0.069811 4.621411

H -1.606069 -0.366264 3.301562

H -2.287965 3.756831 1.850951

H -4.556936 3.139164 0.413980

H -5.234265 0.727384 -0.794652

H -4.118078 1.192595 -2.117115

H 1.856940 2.530692 4.056408

H 2.435824 -0.422713 0.963787

H 2.731062 1.273365 -0.495549

H 1.911059 2.685513 0.371727

H 1.114896 1.770647 -1.021923

H 3.885326 1.066400 4.089741

H 4.152848 -0.103587 2.756520

H 4.485414 1.626486 2.515295

H -1.679875 2.807453 3.743110

H -0.191959 3.253439 4.605940

H -0.706867 4.218365 3.219860

94

**[TS**-bc**]**

C 4.763961 0.288837 1.248118

C 4.647652 0.274397 2.617629

N 3.287178 0.360447 2.883451

C 2.563066 0.457727 1.733936

N 3.472315 0.384575 0.728799

C 3.117530 0.305385 -0.664802

C 2.602026 0.440093 4.167690

C 2.723122 1.471633 -1.334618

C 2.228573 1.300978 -2.677571

C 2.425538 0.060377 -3.395434

C 2.863018 -1.051890 -2.669239

C 3.187647 -0.965622 -1.298850

C 3.633339 -2.192242 -0.553321

C 2.136657 -0.009505 -4.859489

C 2.778620 2.829790 -0.700969

C -3.196099 0.219009 3.758881

C -2.312085 0.171835 4.812919

N -1.051926 0.307879 4.251317

C -1.131008 0.463993 2.894412

N -2.449246 0.380778 2.595882

C -3.022399 0.446047 1.270499

C 0.239401 0.411414 4.911525

N 1.249112 -0.138898 3.970902

C -3.270068 1.719864 0.710815

C -3.901300 1.750490 -0.547333

C -4.257190 0.576297 -1.240494

C -3.969272 -0.666832 -0.638854

C -3.378727 -0.762604 0.633361

C -3.205902 -2.103776 1.302915

C -4.986966 0.639925 -2.555595

C -2.894347 2.985150 1.436180

C 0.269220 -3.216009 0.739668

C 0.688049 -3.342730 2.040122

N 0.814391 -2.052509 2.536977

C 0.489954 -1.147216 1.585549

N 0.143542 -1.843746 0.459582

C -0.267839 -1.188619 -0.743994

C 1.235833 -1.625388 3.876795

C -0.232149 0.262718 -0.703756

C -0.506731 1.045280 -1.928244

C -1.061588 0.339991 -3.052960

C -1.100707 -1.057484 -3.019378

C -0.714572 -1.851257 -1.907131

C -0.836957 -3.344768 -2.073410

C -1.567174 1.093063 -4.244243

C -0.728840 2.538453 -1.775171

Fe 0.609078 0.692733 1.996942

F 0.880175 2.309479 2.701869

N 0.095757 0.955074 0.369081

H 5.648875 0.247416 0.610379

H 5.412958 0.224528 3.395143

H 3.189400 -0.091772 4.939454

H 2.482475 1.497569 4.459218

H 2.091928 2.215287 -3.277065

H 2.975473 -2.016624 -3.179716

H 4.738256 -2.236373 -0.493878

H 3.310250 -3.111056 -1.067355

H 3.257903 -2.215010 0.484252

H 3.004387 0.392320 -5.421914

H 1.273428 0.615137 -5.146411

H 1.971647 -1.041357 -5.207728

H 2.284749 2.861020 0.285402

H 2.321637 3.594925 -1.346897

H 3.834166 3.127435 -0.546106

H -4.286001 0.160368 3.740324

H -2.487466 0.072113 5.886171

H 0.214226 -0.127686 5.877065

H 0.484252 1.472586 5.090296

H -4.152622 2.726878 -0.982928

H -4.257664 -1.591159 -1.156771

H -4.057122 -2.321306 1.975672

H -2.291319 -2.167931 1.918357

H -3.183829 -2.917867 0.559971

H -6.071901 0.490372 -2.395899

H -4.660034 -0.154039 -3.249330

H -4.871894 1.618581 -3.050233

H -3.396987 3.056437 2.418378

H -3.187060 3.874727 0.856973

H -1.805592 3.042560 1.622776

H 0.056846 -3.998493 0.019368

H 0.894177 -4.240913 2.626046

H 0.539093 -2.049584 4.622054

H 2.243404 -2.030310 4.080566

H -1.482936 -1.585411 -3.901495

H -1.500137 -3.799049 -1.315600

H -1.269461 -3.581909 -3.057329

H 0.143291 -3.854654 -2.029447

H -1.814691 0.420836 -5.080366

H -0.844680 1.848504 -4.603500

H -2.481825 1.656598 -3.976919

H -0.138646 2.947708 -0.941571

H -0.481958 3.090307 -2.696161

H -1.793660 2.724643 -1.549472

H 0.872624 1.074523 -2.366132

94

**[TS**-cd**]**

C 4.730209 -0.019240 1.069801

C 4.622175 -0.153236 2.427302

N 3.263728 -0.058174 2.720334

C 2.520549 0.140530 1.600084

N 3.424664 0.158147 0.579848

C 3.122107 0.534491 -0.763853

C 2.654079 0.103449 4.038457

C 2.858474 1.941844 -1.002902

C 2.659953 2.337884 -2.389031

C 2.670123 1.398306 -3.459169

C 2.892739 0.056847 -3.126155

C 3.120852 -0.407341 -1.796336

C 3.366325 -1.868589 -1.553870

C 2.490557 1.862412 -4.874261

C 3.047436 2.977225 0.083566

C -3.108208 0.835740 3.725891

C -2.200476 0.849006 4.753647

N -0.953417 0.680373 4.157371

C -1.064825 0.583252 2.802662

N -2.389965 0.659092 2.538956

C -2.987109 0.566646 1.230683

C 0.365865 0.692646 4.759046

N 1.216681 -0.214346 3.939441

C -3.161593 1.763183 0.494810

C -3.800239 1.660702 -0.753722

C -4.218370 0.423218 -1.283094

C -3.989551 -0.742115 -0.520197

C -3.406500 -0.695603 0.759163

C -3.308356 -1.944810 1.598946

C -4.947050 0.350039 -2.598130

C -2.701696 3.088889 1.042220

C -0.109212 -3.660198 1.276530

C 0.269822 -3.596853 2.587538

N 0.543814 -2.258280 2.855342

C 0.334742 -1.494170 1.757393

N -0.061460 -2.349640 0.755658

C -0.398980 -1.881579 -0.540266

C 0.886688 -1.647644 4.137340

C -0.361209 -0.438167 -0.731139

C -0.741901 0.151667 -1.994492

C -1.106743 -0.693843 -3.058934

C -1.076091 -2.084112 -2.842412

C -0.738647 -2.719463 -1.622903

C -0.770594 -4.230068 -1.610044

C -1.524446 -0.135799 -4.391752

C -0.761834 1.640348 -2.163319

Fe 0.594932 0.344369 1.807610

F 1.011382 2.085769 2.345952

N 0.054033 0.405534 0.227689

H 5.605085 -0.032387 0.417801

H 5.392012 -0.303757 3.186810

H 3.180505 -0.536225 4.770663

H 2.759554 1.157904 4.345096

H 2.463576 3.402505 -2.578499

H 2.893477 -0.688109 -3.933363

H 4.406651 -2.134073 -1.823919

H 2.709978 -2.483260 -2.192706

H 3.212120 -2.152600 -0.501369

H 3.406838 2.375067 -5.227048

H 1.668164 2.593675 -4.966196

H 2.302736 1.021741 -5.560543

H 2.426859 2.758406 0.973800

H 2.796819 3.984603 -0.284366

H 4.110874 2.987876 0.383100

H -4.194951 0.931957 3.731248

H -2.340699 0.971009 5.829171

H 0.301962 0.375991 5.817305

H 0.795645 1.705472 4.691805

H -3.992343 2.580724 -1.322019

H -4.320558 -1.712094 -0.914390

H -4.244093 -2.102449 2.167934

H -2.489870 -1.907964 2.337508

H -3.171184 -2.839037 0.968219

H -6.040690 0.372117 -2.428006

H -4.732326 -0.586470 -3.140969

H -4.713322 1.207158 -3.251824

H -3.283482 3.380632 1.936543

H -2.830040 3.890906 0.298516

H -1.639092 3.060706 1.348052

H -0.413537 -4.526729 0.700876

H 0.357796 -4.390620 3.331839

H 0.020530 -1.735643 4.818245

H 1.734525 -2.194426 4.588663

H -1.347370 -2.736955 -3.682336

H -1.584444 -4.627454 -0.975863

H -0.959463 -4.608869 -2.626425

H 0.182781 -4.675608 -1.275907

H -1.762538 -0.934267 -5.111744

H -0.734873 0.497111 -4.837705

H -2.420629 0.503197 -4.290057

H -0.425229 2.168275 -1.255523

H -0.173083 1.957980 -3.043031

H -1.795073 1.982545 -2.357154

H 1.671817 1.948684 -1.352036

94

**[TS**-de**]**

C 4.813242 -0.184875 1.232019

C 4.663189 -0.278641 2.590136

N 3.303992 -0.103913 2.841802

C 2.610246 0.114103 1.696278

N 3.536377 0.055341 0.699979

C 3.238173 0.337456 -0.665651

C 2.634969 0.079993 4.123344

C 2.589059 1.619499 -0.938018

C 2.335700 1.908413 -2.332404

C 2.595651 0.997896 -3.362771

C 3.160704 -0.245311 -2.994054

C 3.488964 -0.611425 -1.661171

C 4.080878 -1.967740 -1.381610

C 2.335728 1.329469 -4.805461

C 2.884794 2.824514 -0.022942

C -3.109278 0.898879 3.640191

C -2.232221 0.836576 4.690911

N -0.975467 0.660220 4.120111

C -1.046441 0.631700 2.756684

N -2.362726 0.760883 2.465288

C -2.961470 0.759483 1.151838

C 0.319848 0.638004 4.768028

N 1.197567 -0.239411 3.941603

C -3.191124 2.007426 0.525812

C -3.889319 1.996373 -0.699584

C -4.314193 0.799549 -1.311091

C -4.023973 -0.417811 -0.660508

C -3.386032 -0.465583 0.595606

C -3.268547 -1.772542 1.339079

C -5.103629 0.819984 -2.592978

C -2.729536 3.294803 1.154907

C -0.101802 -3.585058 1.152415

C 0.249078 -3.574959 2.473498

N 0.538742 -2.251752 2.793941

C 0.369453 -1.449058 1.718623

N -0.019844 -2.258322 0.679545

C -0.354230 -1.735380 -0.596215

C 0.872906 -1.684453 4.096246

C -0.269924 -0.286941 -0.749106

C -0.718786 0.358874 -1.967125

C -1.149070 -0.444848 -3.040650

C -1.094878 -1.841420 -2.885928

C -0.717961 -2.526372 -1.703433

C -0.737944 -4.036265 -1.757664

C -1.660053 0.162713 -4.317437

C -0.819473 1.845744 -2.046411

Fe 0.686447 0.380805 1.847206

F 1.096016 2.069958 2.462423

N 0.270124 0.502403 0.198177

H 5.707298 -0.242452 0.609822

H 5.405250 -0.440539 3.374456

H 3.112802 -0.553574 4.892830

H 2.720678 1.138004 4.423323

H 1.921060 2.895537 -2.572719

H 3.359469 -0.981910 -3.785586

H 3.692290 -2.716450 -2.091354

H 3.872241 -2.315179 -0.356958

H 5.179570 -1.954952 -1.511905

H 1.832446 0.497222 -5.327897

H 3.290005 1.505198 -5.337208

H 1.727617 2.241325 -4.917566

H 2.599232 2.635601 1.023568

H 2.312669 3.700861 -0.367173

H 3.957784 3.075351 -0.083420

H -4.193059 1.023541 3.619433

H -2.398754 0.910982 5.767114

H 0.220468 0.276032 5.808605

H 0.756671 1.649734 4.760026

H -4.124174 2.957568 -1.176467

H -4.357397 -1.359485 -1.116562

H -4.185641 -1.959748 1.929544

H -2.424366 -1.794342 2.048009

H -3.167004 -2.621351 0.642567

H -6.187621 0.846241 -2.370448

H -4.927224 -0.083059 -3.201392

H -4.885804 1.713093 -3.202464

H -3.276740 3.508098 2.092396

H -2.898526 4.149536 0.481581

H -1.654920 3.261410 1.414548

H -0.409960 -4.425217 0.540518

H 0.306879 -4.396277 3.190524

H 0.005069 -1.798065 4.770949

H 1.723974 -2.237354 4.533014

H -1.395130 -2.461202 -3.740985

H -1.555877 -4.468962 -1.152172

H -0.911784 -4.369712 -2.792433

H 0.216016 -4.487052 -1.433334

H -1.946018 -0.609501 -5.048506

H -0.904809 0.815124 -4.792904

H -2.547654 0.793900 -4.129299

H -0.321533 2.364492 -1.213740

H -0.462468 2.233801 -3.014675

H -1.891697 2.123269 -1.977439

H 1.440826 1.298283 -0.526262

94

**[TS**-ef**]**

Fe 0.414009 -1.718190 -1.034324

F -0.733149 -1.493222 -2.721516

N 2.071534 -2.489448 1.002181

N 1.243583 -0.493362 1.451503

N -0.346294 -0.163865 -0.902283

N -0.719468 -4.235624 -0.376405

N -2.113935 -2.766691 0.395259

N 1.471564 -3.660236 -1.062742

N 2.784876 -2.299641 -2.488758

N 2.622960 -0.137846 -2.490340

C 0.866839 1.149386 -4.401936

C 1.026230 5.288354 -1.471132

C 3.690469 1.192226 -0.157429

C 1.392759 3.850129 -1.705653

C 2.241575 3.165082 -0.810252

C 2.674889 1.850587 -1.053973

C 2.184183 1.209129 -2.217450

C 1.331924 1.843217 -3.148802

C 0.952136 3.169235 -2.862560

C -1.466516 -1.691353 3.009417

C 2.261338 -3.713874 0.219251

C 1.209878 1.805982 3.503673

C -2.027003 4.215621 0.571030

C -1.945953 2.102936 -1.467174

C -1.168283 2.998798 0.752332

C -1.127940 1.974247 -0.222384

C -0.319750 0.799192 0.029212

C 0.474684 0.689743 1.262256

C 0.446480 1.742731 2.202940

C -0.382495 2.851570 1.906814

C 1.253138 -1.518182 0.530349

C 2.600529 -2.115178 2.227659

C 2.087115 -0.871557 2.512080

C 0.422454 -4.713139 -1.135378

C -4.038916 -1.558798 -1.394975

C -4.728698 2.061676 2.095930

C -3.993405 0.839940 1.620555

C -3.019847 0.220525 2.431605

C -2.388014 -0.977718 2.054857

C -2.726011 -1.521270 0.791964

C -3.681281 -0.937659 -0.070129

C -4.302614 0.245004 0.376680

C -0.925665 -2.889649 -0.254316

C -1.776209 -4.958548 0.154931

C -2.659421 -4.022179 0.643416

C 2.338212 -3.664885 -2.272560

C 2.013999 -1.266281 -2.036502

C 3.850045 -1.839812 -3.247621

C 3.743304 -0.466896 -3.246498

H -0.850410 -0.573060 -2.091898

H 0.387287 0.176589 -4.189082

H 1.710697 0.949081 -5.088496

H 0.146178 1.772739 -4.953857

H 0.078244 5.563328 -1.963162

H 1.804353 5.949828 -1.900275

H 0.964502 5.532636 -0.397096

H 3.732531 1.685645 0.826906

H 4.704563 1.272230 -0.594441

H 3.497078 0.115860 -0.007206

H 2.610077 3.686152 0.082899

H 0.303343 3.692696 -3.576759

H -0.682119 -2.276227 2.499594

H -0.984166 -0.982551 3.702127

H -2.036164 -2.404384 3.636337

H 1.937519 -4.580756 0.822824

H 3.335813 -3.834567 -0.007126

H 2.304445 1.766477 3.355845

H 0.921576 1.002339 4.205878

H 0.999036 2.756662 4.017089

H -1.984122 4.880610 1.447666

H -3.083345 3.938625 0.402896

H -1.715794 4.803467 -0.312544

H -1.674111 1.378582 -2.245013

H -1.859150 3.117902 -1.889579

H -3.016401 1.956315 -1.226092

H -0.410224 3.660553 2.648120

H 3.288935 -2.731691 2.810209

H 2.274145 -0.257033 3.385157

H 0.135976 -4.848076 -2.193231

H 0.765028 -5.686713 -0.736212

H -4.564367 -2.523054 -1.261589

H -4.713527 -0.904059 -1.968348

H -3.146179 -1.758533 -2.014798

H -4.094910 2.708464 2.726067

H -5.593282 1.762185 2.720104

H -5.136166 2.655986 1.261101

H -2.779177 0.659274 3.408762

H -5.068466 0.705944 -0.261163

H -1.827406 -6.049425 0.147890

H -3.616238 -4.146708 1.154113

H 1.735031 -3.999372 -3.134886

H 3.209447 -4.338930 -2.169779

H 4.583094 -2.495536 -3.722386

H 4.374174 0.294501 -3.709302
